# Cross-Coupling Reactions with Nickel, Visible Light, and *tert*-Butylamine as a Bifunctional Additive

**DOI:** 10.1021/acscatal.4c07185

**Published:** 2024-12-27

**Authors:** Jonas Düker, Maximilian Philipp, Thomas Lentner, Jamie A. Cadge, João E. A. Lavarda, Ruth M. Gschwind, Matthew S. Sigman, Indrajit Ghosh, Burkhard König

**Affiliations:** † Fakultät für Chemie und Pharmazie, Universität Regensburg, Regensburg 93040, Germany; ‡ Department of Chemistry, University of Utah, 315 1400 E, Salt Lake City 84112, Utah, United States; § Nanotechnology Centre, Centre for Energy and Environmental Technologies, VSB - Technical University of Ostrava, Ostrava-Poruba 708 00, Czech Republic

**Keywords:** AD-HoC, photoredox catalysis, visible light, nickel, cross-coupling, difunctionalization

## Abstract

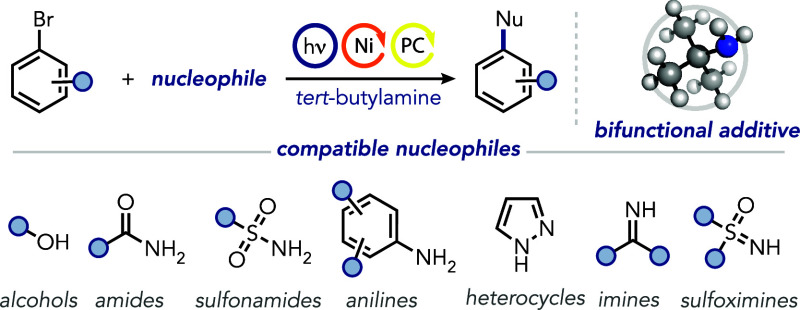

Transition metal catalysis is crucial for the synthesis of complex molecules, with ligands and bases playing a pivotal role in optimizing cross-coupling reactions. Despite advancements in ligand design and base selection, achieving effective synergy between these components remains challenging. We present here a general approach to nickel-catalyzed photoredox reactions employing *tert*-butylamine as a cost-effective bifunctional additive, acting as the base and ligand. This method proves effective for C–O and C–N bond-forming reactions with a diverse array of nucleophiles, including phenols, aliphatic alcohols, anilines, sulfonamides, sulfoximines, and imines. Notably, the protocol demonstrates significant applicability in biomolecule derivatization and facilitates sequential one-pot functionalizations. Spectroscopic investigations revealed the robustness of the dynamic catalytic system, while elucidation of structure–reactivity relationships demonstrated how computed molecular properties of both the nucleophile and electrophile correlated to reaction performance, providing a foundation for effective reaction outcome prediction.

## Introduction

**Figure 1:**
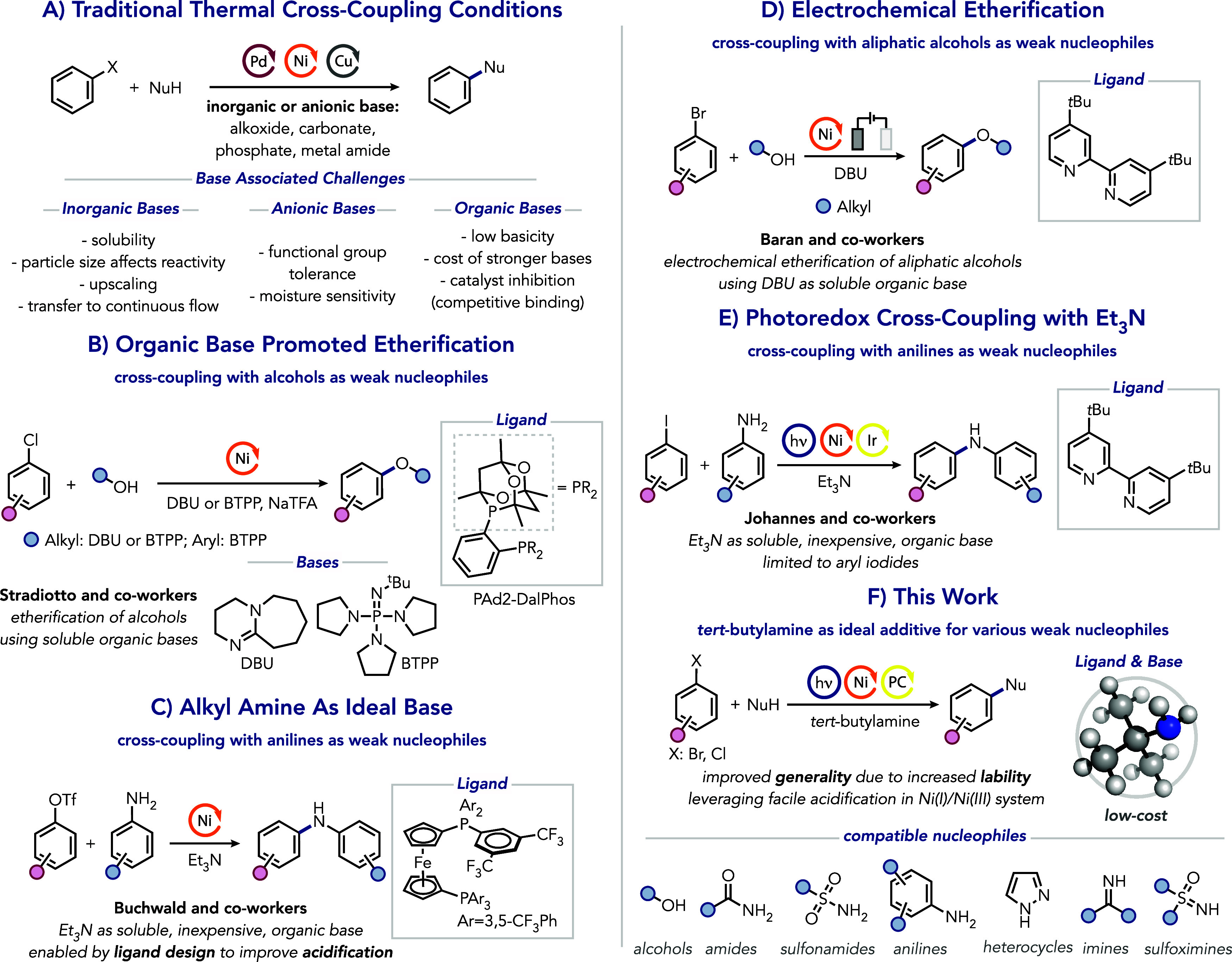
(A) Traditional thermal cross-coupling conditions and base-associated challenges. (B) Nickel-catalyzed etherification using amidine base. (C) Nickel-catalyzed aniline cross-coupling using alkyl amine base. (D) Electrochemical nickel-catalyzed etherification of aliphatic alcohols. (E) Nickel photoredox dual-catalyzed aryl amine cross-coupling using alkyl amine base. (F) *tert*-Butylamine as ligand and base for nickel photoredox cross-coupling of different nucleophile classes.

Transition metal-catalyzed carbon-heteroatom bond formation is fundamental to modern synthetic organic chemistry, playing a pivotal role in the synthesis of pharmaceuticals, agrochemicals, and materials.^1−6^ These transformations are typically performed using palladium,^1,7−13^ copper,^14−19^ or nickel precatalysts,^5,20−32^ with their efficiency reliant on the precise balance of ligands, solvents, and bases for the key catalytic steps to effectively occur. Despite substantial progress, achieving the optimal synergy between ligands and bases remains especially challenging, often requiring case-by-case optimization. While effective ligand design is essential for optimizing oxidative addition, reductive elimination, and active catalyst formation and stability, base selection influences nucleophile binding, deprotonation, and overall catalyst performance.^11,16,29,33,34^ Traditional methods often rely on inorganic bases or strong anionic bases with low nucleophilicity (Figure 1). However, these bases present several challenges, such as insolubility in organic solvents, dependence on particle size and stirring speed on reaction efficiency, the need for elevated temperatures, and clogging during continuous flow up-scaling.^35,36^ Strong anionic bases suffer from moisture sensitivity and limited compatibility with electrophilic functional groups.^37^ In response to these challenges, there has been increasing interest in utilizing organic bases,^38^ such as guanidines,^39−42^ amidines,^39,41,43,44^ and phosphazenes.^40−42,45,46^ Although these bases are more expensive, they offer solubility advantages and, in some cases, improved functional group tolerance. Nonetheless, the field remains dependent on the development of specific ligands and the optimization of conditions for each reaction. For example, Stradiotto and co-workers developed the first thermal nickel-catalyzed protocol for C–O cross-coupling reactions using organic bases in combination with the PAd_2_-DalPhos as a ligand.^22^ The interplay between ligand and base is underscored by the success of coupling aliphatic alcohols with 1,8-diazabicyclo[5.4.0]undec-7-ene (DBU), whereas *tert*-butylimino-tri(pyrrolidino)phosphorane (BTPP) is required for reactions with phenols.

Milder and more cost-effective alkyl amines present a promising alternative but often lack sufficient basicity for effective deprotonation.^47^ To tackle this, Buchwald and co-workers elegantly designed an electron-deficient ligand that allows the deprotonation of nickel-bound anilines with triethylamine for cross-coupling with aryl triflates.^33^ Notably, using the more nucleophilic base, DBU, inhibits the reaction due to its preferential coordination to nickel, demonstrating the additional layer of complexity in achieving the optimal balance between ligands and bases when working with weakly nucleophilic coupling partners.

Similarly, recent advances in catalytic electrochemical and photochemical methods also allowed the use of soluble organic bases.^4,25,48−64^ For instance, Baran and co-workers reported an efficient electrochemical nickel-catalyzed cross-coupling of aliphatic alcohols using a bipyridine ligand and DBU, although this method was ineffective for phenols.^55,56^ Johannes and co-workers used triethylamine in a nickel bipyridine system for C–N cross-coupling reactions with anilines under photoredox conditions, albeit limited to (hetero)aryl iodides as electrophiles.^62^

As such, a general approach that applies to a diverse set of nucleophiles remains elusive. Furthermore, the need to customize ligand and base combinations for specific nucleophile classes is labor-intensive and time-consuming. A more general approach would prove especially beneficial in early stage drug-discovery settings, where practical, time-efficient protocols allow for faster diversification of important building blocks. Thus, the field continues to seek a more generalized solution that can accommodate a wide range of nucleophiles with good catalytic efficiency.

In this context, we recently demonstrated that nickel can engage in various carbon-heteroatom cross-coupling reactions without the need for traditional ligands, relying solely on a photosensitizer and a soluble nitrogen base.^65^ However, different bases were required for different classes of nucleophiles. Especially for weaker nucleophiles, such as aliphatic alcohols and electron-poor phenols, 1,1,3,3-tetramethylguanidine (TMG) was required as a stronger and more expensive organic base. In certain cases, this even led to the undesired formation of the TMG coupled side-product.

An intriguing finding was the efficient cross-coupling of anilines with the assistance of the more nucleophilic cyclohexylamine. We hypothesized that the selectivity toward less nucleophilic anilines might be attributed to their higher acidity combined with a stronger Ni–amido bond formed upon deprotonation.^66^ Intrigued by the bifunctional behavior of primary alkyl amines as both labile ligands and bases, rendering nickel reactive toward oxidative addition and allowing ligand exchange with less nucleophilic coupling partners, we hypothesized that primary alkyl amines could act as a single ideal additive for the cross-coupling of various nucleophiles. Additionally, primary alkyl amines are advantageous due to their cost-effectiveness, availability in diverse substitution patterns, solubility, and mild nature, which allows for broad functional group tolerance. Applying this hypothesis, herein, we report the identification and use of *tert*-butylamine as an ideal additive for photoredox Ni-catalyzed cross-coupling reactions. *tert*-Butylamine effectively combines the roles typically provided by both a ligand and a base, facilitating efficient cross-coupling under mild conditions, and broadening the scope of compatible nucleophiles.

## Results and Discussion

We began our synthetic investigations with phenol as a model nucleophile due to its limited occurrence in nickel photoredox or lack of examples in metal-electrocatalytic protocols. We reasoned in our previous report that alcohols showed low reactivity with cyclohexylamine because their access to the coordination sphere of nickel is hindered by the competitive binding of the more nucleophilic cyclohexylamine.^65^ Given that nucleophile coordination is essential for acidification and subsequent deprotonation with a weak base, we aimed to identify a primary alkyl amine that could enhance nickel’s inherent coordination lability, thus facilitating ligand exchange while maintaining sufficient reactivity toward oxidative addition (Table 1).

**Table 1:**
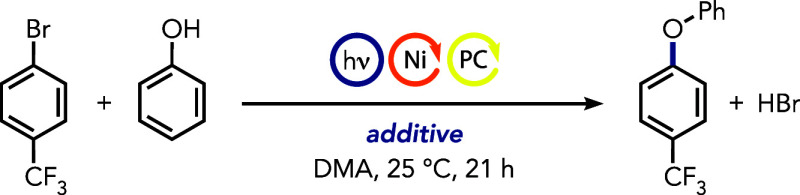

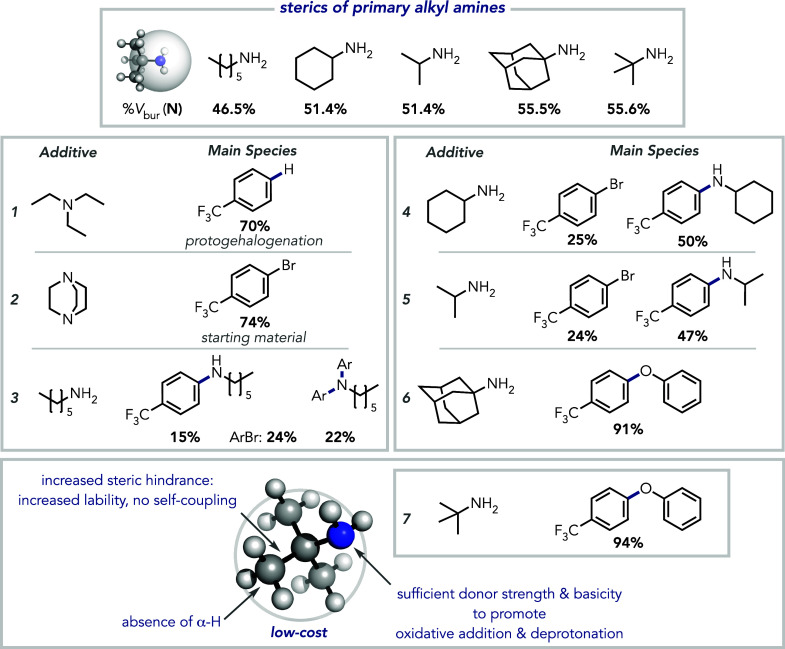
Alkyl Amine Additive Evaluation for the Cross-Coupling Reaction of Phenol^a^

a % *V*_bur_ is a modern steric descriptor capturing the volume percent of a sphere that is occupied by the included atoms.^68,69^ The % *V*_bur_ (N) values correspond to the percent buried volume at nitrogen of the lowest energy conformer with a sphere of 3.0 Å radius. All experiments were performed with NiBr_2_·glyme (5 mol %) and 4CzIPN (0.5 mol %) in DMA (0.4 mL) under nitrogen atmosphere and blue light irradiation ([ArBr] = 0.5 M, [phenol] = 0.75 M, and [additive] = 0.65 M). Yields determined by ^19^F NMR using fluorobenzene as the internal standard.

It is to be noted here that tertiary amines, such as triethylamine and 1,4-diazabicyclo(2.2.2)octane (DABCO) proved less effective under ligand-free photoredox reaction conditions compared to primary amines. For instance, triethylamine led to the formation of the protodehalogenated product, likely due to its role as a sacrificial electron donor in photoredox conditions (Table 1, entry 1).^67^ DABCO, despite being more nucleophilic and expensive, failed to facilitate the desired cross-coupling reaction efficiently (Table 1, entry 2). Hexylamine, a primary amine, underwent self-coupling, yielding mono- and diarylated anilines (Table 1, entry 3). Similarly, cyclohexylamine and isopropylamine exhibited self-coupling to the monoarylation product (Table 1, entries 4 and 5).

A significant improvement was observed with an increase in steric profile: the use of 1-adamantylamine and *tert*-butylamine enabled highly efficient phenol cross-coupling at room temperature, achieving excellent yields of 91% and 94%, respectively. The exceptional performance of *tert*-butylamine in nickel-photoredox cross-coupling reactions is particularly noteworthy (for additional additive evaluation with other nucleophiles see Figures S3 and S4, as well as Tables S1 and S2). The tertiary alkyl group in *tert*-butylamine enhances coordination lability, facilitating efficient ligand exchange and turnover. Additionally, the increased size makes reductive elimination and self-coupling more challenging, while minimizing undesired reductive byproduct formation due to the absence of α-hydrogen atoms. Importantly, the primary amino group in *tert*-butylamine maintains sufficient donor capacity to ensure nickel’s reactivity in the oxidative addition event. Control experiments, in which light, 4CzIPN (as a photocatalyst), or NiBr_2_·glyme was omitted, confirmed the necessity of all components for efficient cross-coupling reactions (Table S3).

**Figure 2:**
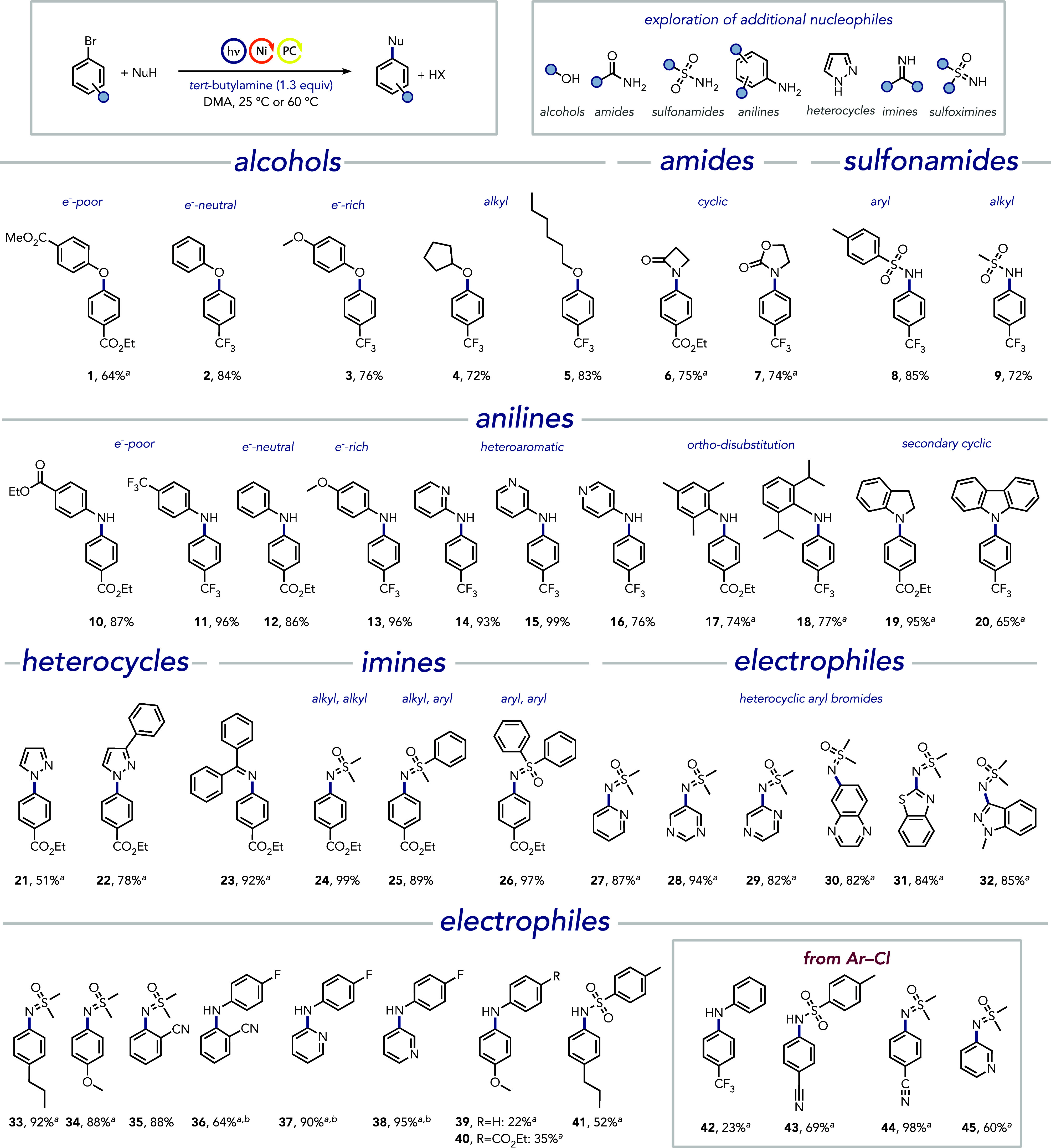
Synthetic examples of C–O and C–N cross-coupling reactions. All experiments were performed with aryl halide (0.2 mmol), NiBr_2_·glyme (5 mol %) and 4CzIPN (0.5 mol %) in DMA (0.4 mL) under nitrogen atmosphere and blue light irradiation ([ArX] = 0.5 M, [nucleophile] = 0.75 M, and [*tert*-butylamine] = 0.65 M) at 25 or 60 °C. ^*a*^Reaction temperature 60 °C. ^*b*^Yield determined by ^19^F NMR using fluorobenzene as the internal standard. See Supporting Information for further details.

Based on our hypothesis, nucleophiles that can compete with *tert*-butylamine for nickel coordination meet the prerequisites for successful cross-coupling. This is further supported by the observation that high *tert*-butylamine concentrations (2.6 equiv) lead to decreased reaction rates (Tables S5 and S6). Thus, nucleophiles of similar or higher nucleophilicity than phenol were thought to be compatible with our optimized reaction condition. Consequently, the scope of C–O cross-coupling reactions was explored using a variety of electronically differentiated phenols (Figure 2). Both electron-rich and electron-poor phenols were effectively cross-coupled, achieving good to excellent yields of the desired products **1**–**3**. Additionally, aliphatic alcohols such as cyclopentanol (**4**) and hexanol (**5**), under the same reaction conditions, were also successfully employed as nucleophiles, providing high isolated yields.

Next, we explored the synthetic versatility of using *tert*-butylamine as an additive in C–N bond-forming reactions with a range of different nitrogen nucleophiles. Nucleophiles with pharmaceutical relevance, such as a lactam (**6**), a carbamate (**7**), or sulfonamides, whether aryl (**8**) or alkyl (**9**), successfully underwent cross-coupling reactions, yielding the desired cross-coupled products in good to excellent yields. Moreover, anilines gave the desired products in good to excellent yields. Notably, electron-poor (**10** and **11**), electron-neutral (**12**), and electron-rich (**13**) anilines led to the efficient formation of the respective products. Heteroaromatic anilines were no exception, giving the desired products **14**–**16** in good to excellent yields. The steric hindrance exerted by alkyl groups in the *ortho*-position was well tolerated (**17** and **18**), even with isopropyl groups at both *ortho*-positions. Only 2,6-di-*tert*-butyl aniline was too sterically hindered to undergo the reaction effectively. The cross-coupling reactions with cyclic secondary anilines, such as 2,3-dihydroindole (**19**) and carbazole (**20**), were also successful, yielding the desired products in 95% and 65% isolated yields, respectively. Moreover, these cross-coupling reactions are compatible when five-membered heterocycles are employed as nucleophiles. For instance, pyrazoles can be employed, yielding the desired products **21** and **22** in good, isolated yields. Not surprisingly, more nucleophilic imines can be efficiently coupled as well. Benzophenone imine, a common ammonia surrogate in cross-coupling reactions, when employed as a nucleophile with ethyl 4-bromobenzoate as the model electrophile, yielded the desired product **23** in an excellent 92% isolated yield. Similarly, sulfoximines, which have recently gained increased attention as promising bioisosteres for sulfones and sulfonamides in drug discovery,^70^ could be efficiently cross-coupled in excellent to near-quantitative yields. Alkyl–alkyl (**24**), alkyl-aryl (**25**), and aryl–aryl (**26**) sulfoximines were all effective as nucleophiles, providing the desired coupling products in good to excellent yields. It is to be noted here that these cross-coupling reactions are also effective in the presence of DABCO, an alternative and more expensive base, which precipitates from DMA upon protonation by the generated mineral acid (e.g., HBr). However, the use of *tert*-butylamine not only allows for faster reactions with lower equivalents (Table S2) but enables precipitate-free reaction conditions, which may facilitate the scale-up of cross-coupling reactions under continuous flow conditions.^71,72^

The cross-coupling reactions are not limited to aryl halides; a range of (hetero)aryl halides covering (hetero)aromatic scaffolds privileged in drug discovery^73^ are equally compatible as electrophiles and did not require any additional optimization as exemplified using dimethylsulfoximine, anilines, and *para*-toluenesulfonamide as nucleophiles. Notably, nitrogen-containing heterocycles often present difficulties in transition metal catalysis due to their propensity to coordinate and deactivate the catalyst.^74,75^ Such complications were not observed, and various heterocycles containing different substituents yielded the desired products **27**–**32** in very good to excellent yields. Importantly, the cross-coupling reactions were also effective when base-sensitive electrophiles (**30** and **32**) were used.^76,77^ Notably, the position of the C–Br bond in the (hetero)aryl core had minimal influence, consistently providing the desired product in good to excellent yields.

Furthermore, the introduction of more electron-donating *para*-substituents on the aryl halides was well tolerated, furnishing the desired products **33** and **34** in 92% and 88% isolated yield, respectively, although requiring longer reaction times at 60 °C. The good yield of sulfoximine with 4-bromoanisole could indicate the involvement of dimethylsulfoximine in the oxidative addition (coordination of dimethylsulfoximine to nickel was observed in the NMR, Figure S6). Increasing the steric hindrance of the aryl bromide with an *ortho*-cyano substituent was well tolerated with dimethylsulfoximine (**35**) but led to a noticeable yield reduction with *para*-fluoroaniline (**36**) as the nucleophile.

When less nucleophilic anilines and sulfonamides were utilized, bromopyridines (**37** and **38**) led to excellent yields, whereas difficult-to-activate, electron-rich electrophiles (**39–41**) furnished the desired products in synthetically useful yields, although diminished. To our delight, electron-poor aryl chlorides and chloro-pyridines were also effective in yielding the desired product in good yields with aniline (**42**), sulfonamide (**43**), and dimethylsulfoximine (**44** and **45**).

**Figure 3:**
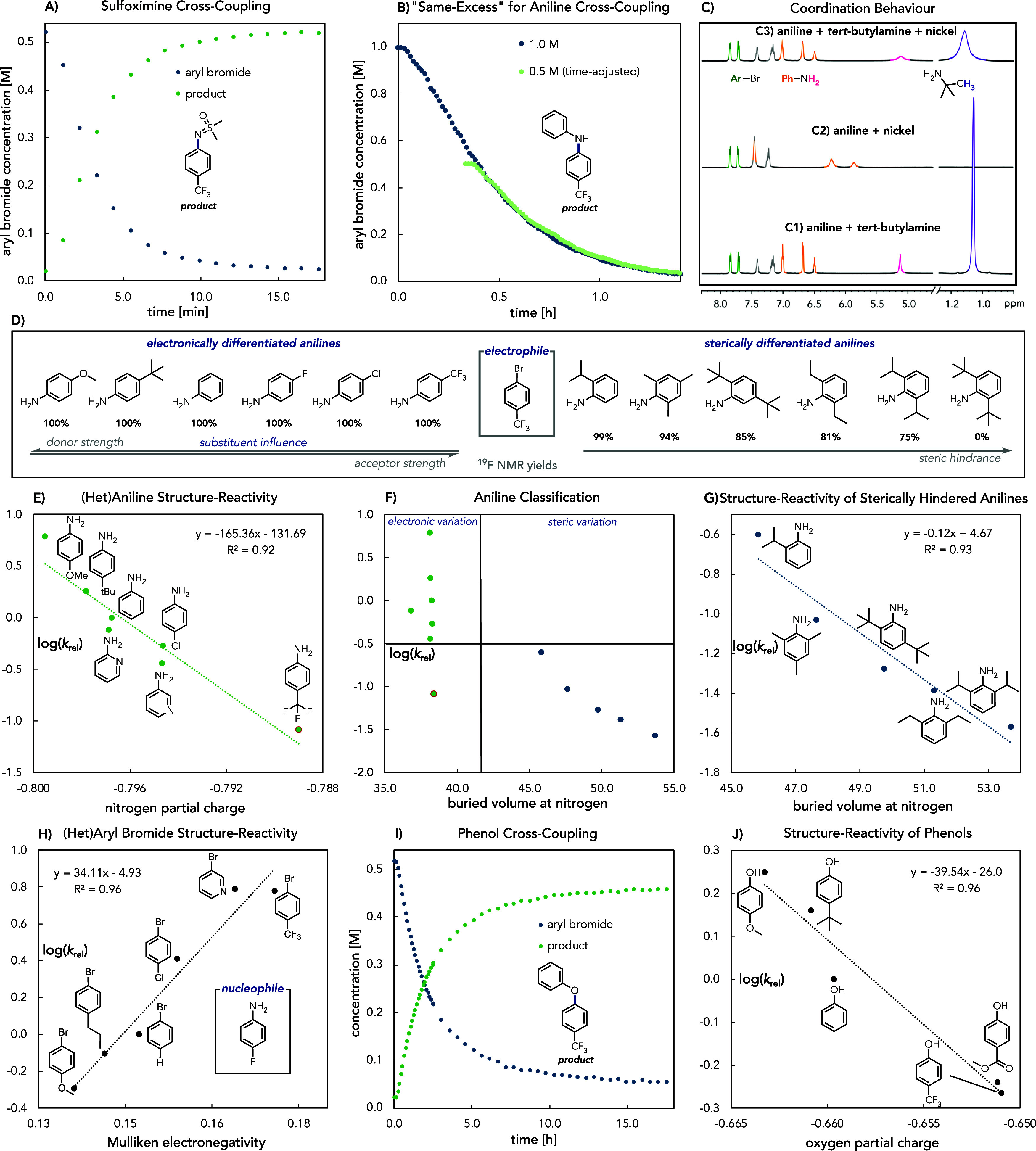
In situ NMR analysis and structure–reactivity relationships. All experiments were performed with NiBr_2_·glyme (5 mol %) and 4CzIPN (0.5 mol %) in DMA under blue light irradiation if not stated otherwise. Reaction kinetics and yields were determined by ^19^F NMR. *k*_rel_ (*k*_X_/*k*_reference_) corresponds to the product distribution in the competition experiments with the unsubsituted aniline, phenol, or bromobenzene as the reference in each reaction. (A) In situ ^1^H NMR kinetics of sulfoximine cross-coupling in NMR tube ([ArBr] = 0.5 M, [sulfoximine] = 0.75 M, and [*tert*-butylamine] = 0.65 M at 60 °C). (B) “Same-Excess” experiments for the aniline cross-coupling, probing the robustness of the catalytic system (blue curve: [ArBr] = 1.0 M, [aniline] = 1.25 M, and [*tert*-butylamine] = 1.07 M at 60 °C; green curve (time-adjusted): [ArBr] = 0.5 M, [aniline] = 0.75 M, and [*tert*-butylamine] = 0.57 M at 60 °C). (C) NMR coordination study with aniline and *tert*-butylamine at 25 °C (C1: [ArBr] = 0.5 M, [aniline] = 0.75 M, and [*tert*-butylamine] = 0.65 M without nickel and 4CzIPN; C2: [ArBr] = 0.5 M, [aniline] = 0.75 M, without *tert*-butylamine; C3: [ArBr] = 0.5 M, [aniline] = 0.75 M, and [*tert*-butylamine] = 0.65 M). (D) ^19^F NMR yields of various substituted anilines ([ArBr] = 0.5 M, [aniline] = 0.75 M, and [*tert*-butylamine] = 0.65 M). (E) (Hetero)Aniline structure–reactivity relationship with nitrogen partial charge ([ArBr] = 0.5 M, [aniline] = 0.75 M for each aniline in a competition experiment, and [*tert*-butylamine] = 0.65 M at 25 °C). (F) Aniline classification based on size as measured by percent buried volume with 3.5 Å radius. (G) Sterically hindered aniline structure–reactivity relationship with buried volume at nitrogen (3.5 Å radius) ([ArBr] = 0.5 M, [aniline] = 0.75 M for each aniline in a competition experiment, and [*tert*-butylamine] = 0.65 M at 25 °C). (H) (Hetero)Aryl bromide structure–reactivity relationship with Mulliken electronegativity ([ArBr] = 0.5 M for each aryl bromide in a competition experiment, [4-fluoroaniline] = 0.5 M, and [*tert*-butylamine] = 0.65 M at 60 °C). (I) In situ NMR kinetics of phenol cross-coupling in NMR tube ([ArBr] = 0.5 M, [phenol] = 0.75 M, and [*tert*-butylamine] = 0.65 M at 60 °C). (J) Phenol structure–reactivity relationship with oxygen partial charge ([ArBr] = 0.5 M, [phenol] = 0.75 M for each phenol in a competition experiment, and [*tert*-butylamine] = 0.65 M at 25 °C). See Supporting Information for further details.

Next, we sought to gain deeper insights into the catalytic system. Intrigued by the efficient sulfoximine cross-coupling with *tert*-butylamine, we performed in situ ^1^H NMR spectroscopic analysis,^78,79^ which revealed that the coupling proceeds to completion within 18 min in the NMR tube without stirring at 60 °C (Figure 3A). Similarly, the aniline cross-coupling could be monitored in the NMR tube (Figure 3B). This allowed us to conveniently conduct “same-excess” experiments pioneered by Blackmond to probe for possible product inhibition or catalyst deactivation (Figure 3B).^80,81^ The experiments were performed by keeping the excess of the nucleophile and base constant while varying the initial aryl bromide concentration. Graphical comparison of the overlay of reaction profiles after reaching the same concentration demonstrates how robust catalysis can be maintained in a labile system involving rapid interconversion of various species, one or more of which exhibit catalytic competence. The accumulation of HBr and product does not interfere with the catalyst, and the active catalytic species can be regenerated without irreversible deactivation which would result in a loss of catalytic performance.

To gain a deeper understanding of the coordination behavior of different species within the catalytic system (Figure 3C), we recorded ^1^H NMR spectra for solutions of aniline and *tert*-butylamine alone (C1), aniline with 5 mol % nickel (C2), and aniline with *tert*-butylamine and 5 mol % nickel (C3). Coordination of aniline with nickel resulted in a noticeable shift of the aromatic hydrogen signals (C2), indicating effective coordination. However, upon the addition of *tert*-butylamine (C3), the aniline signals shifted back to their original positions (C1 for reference), demonstrating the superior coordination ability of *tert*-butylamine.

The preferential coordination of *tert*-butylamine made us question how the nucleophile properties influence reactivity. While anilines of varying electronic nature all lead to quantitative product formation with 4-bromobenzotrifluoride, slightly diminished yields were obtained for sterically more hindered anilines (Figure 3D). Therefore, we performed competition experiments to determine relative reaction rates with aniline as the reference nucleophile. Despite the dynamic nature of the system with various species in equilibrium, a clear trend in relative rates was observed, allowing for the classification of anilines based on their size as defined by the percent buried volume at nitrogen. *Ortho*-substituted anilines with Boltzmann-weighted buried volumes (3.5 Å radius) above 45% exhibited slower rates compared to anilines with varying electronic properties, except for 4-(trifluoromethyl)aniline, which reacted at a rate comparable to 2,4,6-trimethylaniline (Figure 3F).

The assessment of electronically and sterically differentiated anilines revealed significant reactivity differences that align well with computationally derived descriptors describing the electronic and steric properties of the anilines. For electronically varied anilines, a linear correlation with the nitrogen partial charge (determined from a natural population analysis (NPA) calculation) was established, showcasing that more electron-rich anilines exhibit faster reaction rates (Figure 3E). Similarly, a linear correlation with the buried volume at nitrogen was observed for sterically hindered anilines (Figure 3G). These results underscore the crucial role of aniline nucleophilicity on the product selectivity in competition experiments, emphasizing the importance of *tert*-butylamine/nucleophile exchange for product formation.

Additionally, we examined the impact of the aryl bromide properties on the aniline cross-coupling. Consistent with previous nickel-photoredox catalyzed protocols and mechanistic studies,^82−84^ electron-rich aryl halides led to decreased reaction rates in competition experiments (bromobenzene used as the reference), demonstrated by a correlation with the Mulliken electronegativity of the aryl bromides (Figure 3H).

Next, we turned our attention to the phenol coupling partners. Interestingly, the analogous NMR coordination experiment performed with aniline (Figure 3C) indicates that, in the absence of *tert*-butylamine, DMA coordinates preferentially over phenol, consistent with the weak nucleophilicity of this coupling partner (Figure S14). While the reaction still proceeds reliably in the NMR tube, significantly prolonged reaction times were observed when compared to the reaction vial (Figure 3I). For electronically differentiated phenols, a similar correlation between the oxygen partial charge of phenols and their respective reaction rates was identified in competition experiments (Figure 3J). Interestingly, the NPA partial charge on oxygen had a significantly lower impact on reaction rate as compared to the analogous anilines. Performing cross-coupling reactions with electronically differentiated phenols in individual reaction vials still led to the same trend that the more nucleophilic phenols are noticeably faster, emphasizing the nucleophile’s impact on the reaction rate (Figure S17).

These results underscore the importance of both coupling reagents on the reaction rate. The univariate linear correlations with accessible and interpretable molecular features not only provide a foundation for more sophisticated data science models capable of quantitative reaction outcome prediction but moreover enable qualitative reactivity estimation simply by evaluating the most fundamental properties of the reagents.^19,42,85,86^ The practicality of performing these reactions and predicting their outcomes is notable, given the complexity at the microscopic scale.

**Figure 4:**
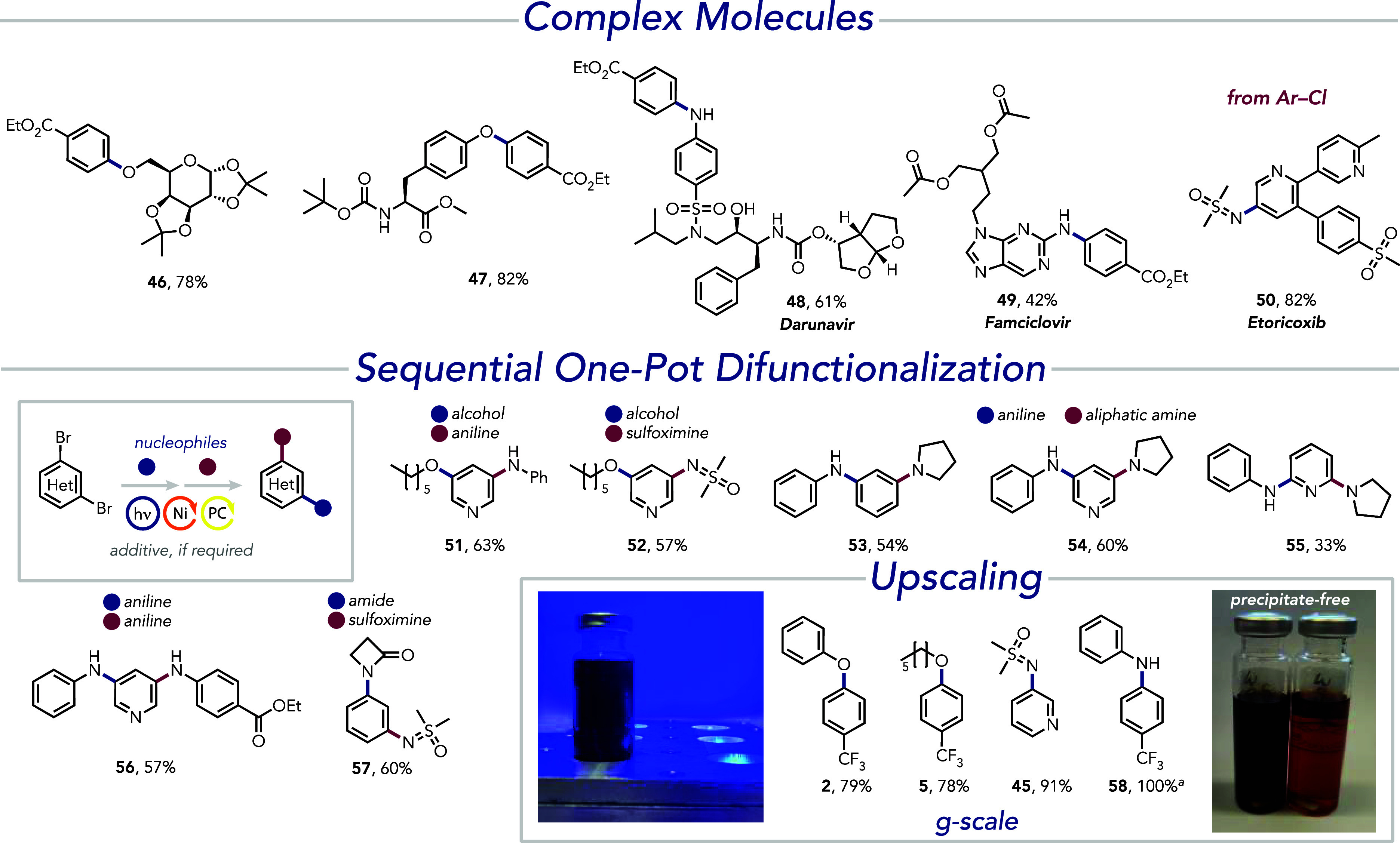
Synthetic applications including the synthesis of complex molecules, sequential one-pot difunctionalization, and upscaling. All experiments were performed with NiBr_2_·glyme (5 mol %) and 4CzIPN (0.5 mol %) in DMA under blue light irradiation at 25 or 60 °C. ^*a*^Yield determined by ^19^F NMR using fluorobenzene as the internal standard. Detailed experimental conditions can be found in the Supporting Information.

The simplicity and versatility of the unified cross-coupling reaction conditions have proven highly efficient for the functionalization of biomolecules, whether employed as electrophiles or nucleophiles (Figure 4). For instance, the use of diacetone-d-galactose and boc-l-tyrosine methyl ester as nucleophiles allows C–O cross-coupling reactions for the functionalization of phenol and aliphatic alcohol, respectively, giving the desired products **46** and **47** in good isolated yields. The use of biomolecules as nucleophiles is demonstrated using darunavir (**48**) and famciclovir (**49**) in C–N cross-coupling reactions. When used as an electrophile, etoricoxib allows for efficient functionalization of the C–Cl bond with sulfoximine (**50**).

The system’s simplicity becomes more apparent when applied to difunctionalization reactions, which involve forming one or two different chemical bonds to rapidly construct molecular complexity. Previously, we reported a nickel photoredox protocol for straightforward introduction of molecular complexity through sequential one-pot bifunctionalization of arenes and heteroarenes with C–S cross-coupling reactions, followed by various C–N cross-coupling reactions.^87^ However, sequential C–N/C–N and C–O/C–N cross-couplings posed challenges, such as the requirement for DABCO, which led to precipitate formation, and the self-coupling of additives like cyclohexylamine and TMG, complicating sequential transformations.

The introduction of *tert*-butylamine has simplified these reactions. To our delight, alcohols, anilines, sulfoximines, and aliphatic amines could be employed in sequential one-pot difunctionalizations using *tert*-butylamine as the sole additive. For example, hexanol and aniline can be installed onto 1,3-dibromopyridine, giving the desired product **51** in a good 63% isolated yield considering a two-step transformation. Employing dimethylsulfoximine as the second nucleophile allowed for isolation of 57% of the respective product **52**. An aniline coupling followed by pyrrolidine was successfully introduced onto 1,3-dibromobenzene, 1,3-dibromopyridine, and 2,6-dibromopyridine, yielding the respective isolated products **53**–**55** in 33%–60%. Additionally, cross-coupling of two electronically different anilines to furnish the product **56** in 57% yield was accomplished, as well as the coupling of a β-lactam, followed by dimethylsulfoximine to yield the product **57** in 60%.

The experimental simplicity of the sequential difunctionalizations is noteworthy. Following the first cross-coupling reaction, the second nucleophile and *tert*-butylamine are added to the reaction mixture, which is then photoirradiated under nitrogen. Lastly, the homogeneous, precipitate-free reaction conditions facilitate convenient scale-up. Figure 4 illustrates the effective performance of the system on a gram-scale, demonstrating the synthesis of **2**, **5**, **45**, and **58** with yields of 78–91%. Additionally, the reactions can be performed with inexpensive nickel hydrate salts and low catalyst loadings (1.25 mol %) as exemplified for the cross-coupling reaction with sulfoximine (Tables S4 and S7).

## Conclusions

In conclusion, we report a general cross-coupling protocol employing *tert*-butylamine as a dual-function additive, marking a significant advancement in nickel-catalyzed photoredox chemistry. This method simplifies reaction conditions with good efficiency, demonstrating broad applicability across various nucleophiles and electrophiles, including biomolecules. *tert*-Butylamine effectively serves as both a ligand and a base, addressing common challenges associated with traditional bases and ligands, such as solubility issues and the need for specific optimization. Additionally, simple and interpretable structure–activity relationships provide a foundation for more sophisticated data science models and enable qualitative estimation of reaction outcomes. The compatibility of various nucleophiles allows sequential one-pot difunctionalizations, showcasing its practicality for constructing molecular complexity and rapid compound library generation. The homogeneous and precipitate-free nature of the system with cost-effective reagents facilitates convenient scaling to gram quantities and promises applicability in larger-scale processes using flow technology.

## Materials and Methods

### Materials

The chemicals were obtained from commercial sources and were used as received unless otherwise specified (see the Supporting Information for more details).

### Methods

A 5 mL crimp top vial was charged with a magnetic stirring bar, (het)aryl halide (0.2 mmol, 1.0 equiv), the respective nucleophile (0.3 mmol, 1.5 equiv) and 0.4 mL of a catalyst stock solution containing 4CzIPN (0.8 mg, 0.001 mmol, 0.005 equiv) and NiBr_2_·glyme (3.2 mg, 0.01 mmol, 0.05 equiv) dissolved in DMA. The reaction mixture was then degassed and refilled with nitrogen two times via a syringe needle before *tert*-butylamine (27.3 μL, 0.26 mmol, 1.3 equiv) was added via syringe. After degassing one more time and refilling with nitrogen, the reaction mixture was photoirradiated through the plane bottom side of the snap vial using a single blue LED (455 (±15) nm). After completion of the reaction, the product was purified by column chromatography (see the Supporting Information for more details).

## Supporting Information Available

Additional experimental details, characterization data of the isolated products, and photographs of the experimental setup can be found in the Supporting Information (PDF)

## Notes

3D molecular structures were visualized by using PyMol.^88^
