# Supplementary material for: Cross-Coupling Reactions with Nickel, Visible Light, and tert-Butylamine as a Bifunctional Additive
Source: ACS Catal. 2024 Dec 27;15(2):817–27. doi: 10.1021/acscatal.4c07185 (PMC11744660; doi:10.1021/acscatal.4c07185)
Supplement: Supplementary file 1 — cs4c07185_si_001.pdf [file cs4c07185_si_001.pdf]

Supporting Information

Cross-coupling reactions with nickel, visible light, and *tert*-butylamine as a bifunctional additive

Jonas Düker[1], Maximilian Philipp[1], Thomas Lentner[1], Jamie Cadge[2], João E. A. Lavarda[1], Ruth M. Gschwind[1], Matthew S. Sigman[2], Indrajit Ghosh[1,3,*], and Burkhard König[1,*]

[1]Fakultät für Chemie und Pharmazie, Universität Regensburg, 93040 Regensburg, Germany.

[2]Department of Chemistry, University of Utah, 315 1400 E, Salt Lake City, Utah 84112, United States

[3]Nanotechnology Centre, Centre for Energy and Environmental Technologies, VŠB–Technical University of Ostrava, 708 00 Ostrava-Poruba, Czech Republic.

The correspondence may be addressed to: indrajit1.ghosh@ur.de; burkhard.koenig@ur.de

Table of Contents

1. *General Information* 2
2. *Additive Evaluation for Different Nucleophiles* 6
3. *Additional Control Experiments* 8
4. *Competitive Coordination Experiments in NMR* 11
5. *NMR Yields of Structurally Diverse Anilines* 18
6. *Structure-Reactivity Relationships with Competition Experiments* 18
7. *Structure-Reactivity with Individual Experiments* 21
8. *Further In-Situ Kinetics* 23
9. *Experimental Procedures* 27
10. *NMR Spectra* 47
11. *Computational Details* 115
12. *References* 145

1. General Information

Reagents and solvents: All reactions were carried out in dry *N,N*-dimethylacetamide (DMA). DMA was dried with 3 Å molecular sieves according to a reported procedure.[1]

Commercially available chemicals were purchased at the highest commercial quality and used without further purification. 4CzIPN was synthesized according to a reported procedure.[2]

Gas chromatography: Gas chromatography with a flame-ionization detector (GC-FID) and gas chromatography coupled to low-resolution mass spectrometry (GC-MS) were performed using a capillary column (length: 30 m; diam.: 0.25 mm; film: 0.25 μM) using He as a carrier gas. GC-MS was performed on a 5975 MSD single quadrupole detector. Standard heating procedure: The initial temperature was set to 40 °C and was held for 3 minutes. Then, the temperature was increased to 280 °C at a rate of 15 °C/min and was held for 5 minutes. Lastly, the temperature was increased to 300 °C at a rate of 25 °C/min.

Flash column chromatography: Flash chromatography was performed either on an automated Biotage® Isolera™ Spektra or Biotage® Selekt system using a prepacked Biotage® Sfär Silica HC Duo 20 μm column.

TLC: Thin-layer chromatography (TLC) was performed on silica gel coated alumina plates (Macherey-Nagel, TLC sheets ALUGRAM Xtra SIL G UV254). Detection of the spots was accomplished utilizing UV light (254 nm).

NMR: The NMR spectra were recorded at room temperature using a Bruker Avance 400 (400 MHz for ^{1}H, 101 MHz for ^{13}C, 376 MHz for ^{19}F) NMR spectrometer. All chemical shifts are reported in δ-scale as parts per million [ppm] (multiplicity, coupling constant *J*, number of protons) relative to the solvent residual peaks as the internal standard.[3] Coupling constants *J* are given in Hertz [Hz]. Abbreviations used for signal multiplicity: ^{1}H-NMR: br = broad, s = singlet, d = doublet, t = triplet, q = quartet, dd = doublet of doublets, dt = doublet of triplets, and m = multiplet.

HRMS: High-resolution mass spectra (HRMS) were obtained from the central analytic mass spectrometry facilities of the Faculty of Chemistry and Pharmacy of the University of Regensburg. The measurements were carried out on either a JEOL AccuTOF GCX or Agilent Q-TOF 6540 UHD.

LEDs: For the photochemical reactions 455 (±15) nm LEDs (OSRAM Oslon SSL 80 LDCQ7P-1U3U (blue, λ_{max} = 455 (±15) nm, I_{max} = 1000 mA, radiant power ~ 500 mW) were used.

The large-scale reactions were performed under highpower LED illumination using 455 (±15) nm LEDs (blue, λ_{max} = 455 (± 15) nm, I_{max} = 1000 mA, radiant power ~ 1.4 W)

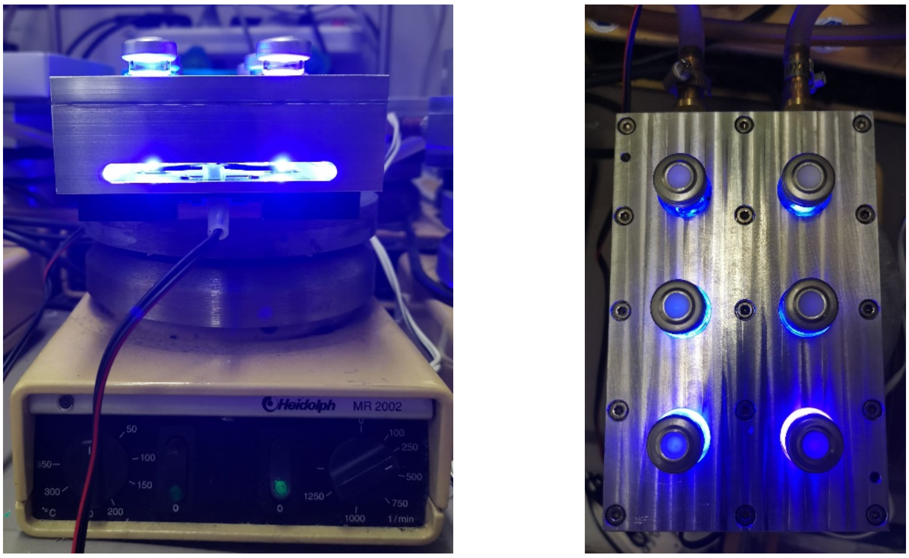

Figure S1: Side and top view of the photochemical reaction setup. The reaction vials (5 mL crimp cap vials) were illuminated from the bottom side with blue LEDs (λ = 455 (± 15) nm). The reaction temperature was maintained at either 25.0 °C or 60.0 °C by a custom-made thermostated aluminium cooling block.

General preparation of the NMR samples

For sample preparation, a Schlenk technique was employed with argon as the inert gas (Figure S2). The reagents were added to a dry NMR tube under argon atmosphere using Gilson direct displacement syringes (MICROMAN E M100E, 10-100 µL, MICROMAN E M100E, 1-10 µL, MICROMAN E M100E, 100-1000 µL). After addition of the electrophile (0.15 mmol, 1.0 equiv) and nucleophile (0.225 mmol, 1.5 equiv), 0.3 mL of a stock solution containing $NiBr_2$·glyme (2.4 mg, 5.0 mol%), and photocatalyst 4CzIPN (0.6 mg, 0.5 mol%) in non-deuterated *N*,*N*-dimethylacetamide (DMA), was added. Next, the NMR tube was evacuated and filled with argon two times, *tert*-butylamine (1.3 equiv) was added, and the NMR tube was evacuated and refilled with argon one more time before the glass fiber was inserted under argon flow and sealed with Parafilm®.

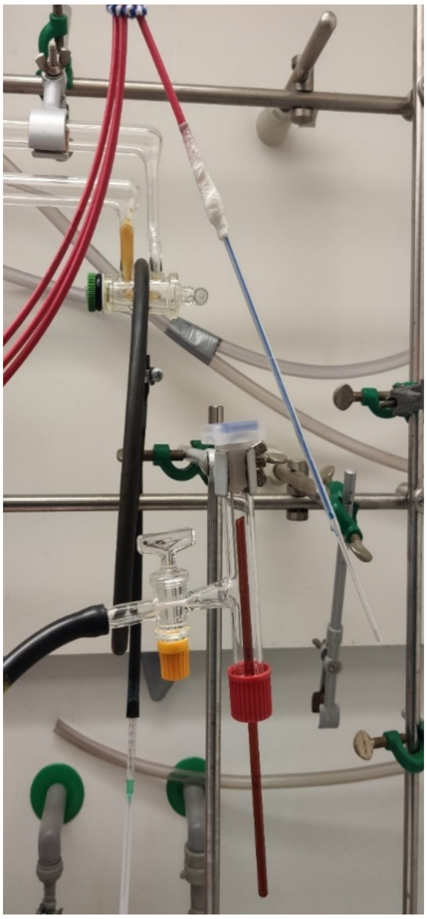

Figure S2: Image depicting the preparation of the NMR sample under an inert gas atmosphere. The NMR tube was evacuated and filled with argon two times after the addition of the starting materials and catalyst stock solution. After addition of *tert*-butylamine, the NMR tube was evacuated and refilled with argon again before the glass fiber was inserted under argon flow.

General setup for the *in-situ* illumination inside the spectrometer

For the illumination within the NMR spectrometer, a setup reported by Gschwind and co-workers was employed.[4] A OSLON® SSL 80 LD CQ7P-2U3U-W5-1-K LED was utilized as the light source with an effective peak wavelength of 451 nm, operating at a current of 1000 mA.

The NMR experiments were conducted at 333 K and 298 K on an AVANCE III-HD 400 MHz spectrometer on a 5 mm PA BBO 400S1 BBF-H-D-05 Z N (^{1}H, ^{19}F, BB) probe head with a z-gradient and Bruker AVANCE III-HD 600 MHz spectrometer with TBI (Triple resonance broadband inverse) 5 mm CPPBBO 1H/19F-BB probe head with Z-gradient and BVT unit. ^{19}F-NMR experiments were used for the *in-situ* illumination kinetics and referenced to fluorobenzene (^{1}H-NMR: 7.49-7.11 ppm (multiplet), ^{19}F-NMR: -113.96 ppm (multiplet)).

The T_1 times of the model electrophile 1-bromo-4-(trifluoromethyl)benzene (1.49 s) and aniline coupling product *N*-phenyl-4-(trifluoromethyl)aniline (0.99 s) were determined via an inversion recovery experiment. To guarantee quantitative results, the relaxation delay (d1) was put to 30 s, which ensures full relaxation of the nuclei (d1 ≥ 5*T_1).
For the experiments, the acquisition time was set to 3 s and the number of scans (NS) to 1. In between each fluorine spectrum one single scan proton spectrum was acquired. Data points were acquired every 50–62 s to achieve complete relaxation.
Processing of the NMR spectra was executed in Topspin 3.2. The spectra were phased and the baseline was corrected. The data was analyzed in Microsoft Excel and Origin 2021.

2. Additive Evaluation for Different Nucleophiles

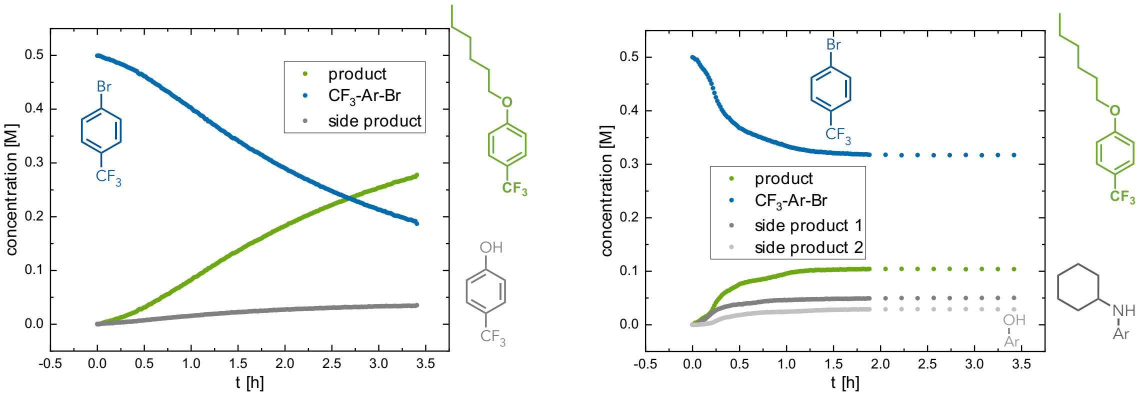

Figure S3: Evaluation of *tert*-butylamine and cyclohexylamine as additives for hexanol cross-coupling. In-situ NMR kinetics run in the NMR tube. ^{19}F-NMR spectra at 298K. 1.5 equiv **1-hexanol** used as the nucleophile and 1-bromo-4-(trifluoromethyl)benzene (1.0 equiv) as the electrophile. Left: 1.3 equiv ***tert*-butylamine**. Right: 1.3 equiv **cyclohexylamine** instead of *tert*-butylamine.
With cyclohexylamine (right), stagnation of reaction progress and self-coupling takes place (right), rendering tert-butylamine (left) the superior additive.

Table S1: Cyclohexylamine additive evaluation. Ni: NiBr₂·glyme (5 mol%), PC: 4CzIPN (0.5 mol%). h**v**: blue LED. Yield determined with ^{19}F-NMR and fluorobenzene as internal standard.

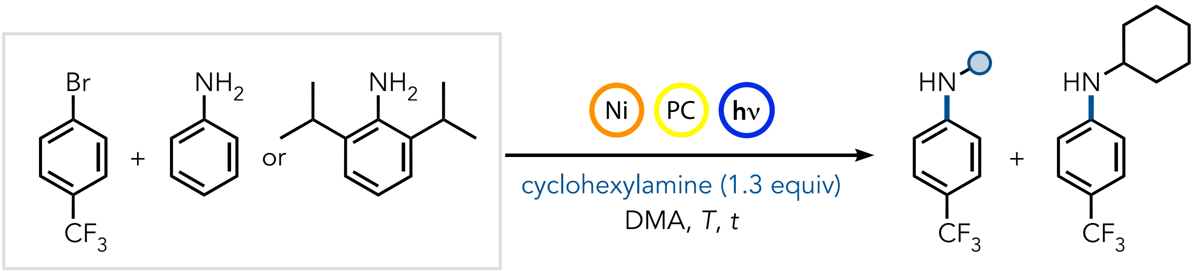

entry	nucleophile (1.5 equiv)	*T* [° C]	time [h]	yield	additive-coupling
1	aniline	25 °C	19 h	93%	7%
2	aniline	60 °C	19 h	93%	7%
3	2,6-diisopropylaniline	25 °C	19 h	68%	21%

Self-coupling of cyclohexylamine diminishes the yield of the aniline cross-coupling, especially when more sterically hindered anilines are introduced.

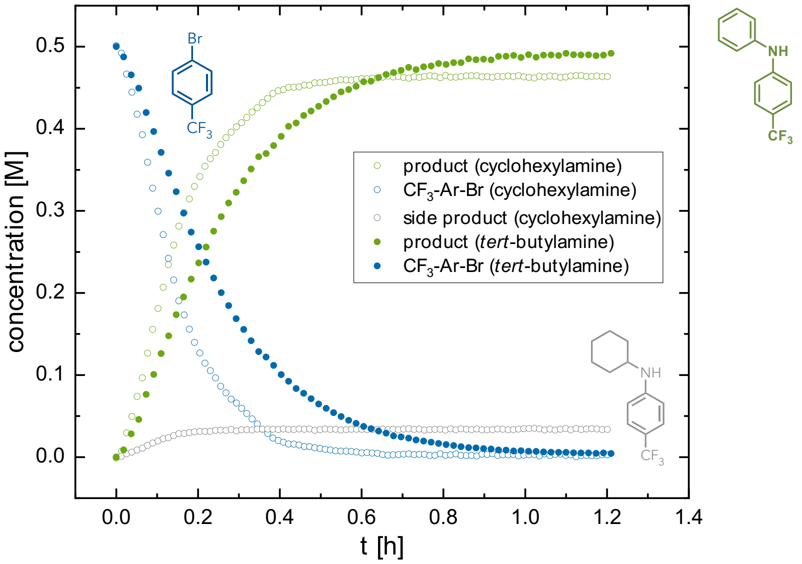

Figure S4: Reaction rate comparison of *tert*-butylamine and cyclohexylamine for aniline cross-coupling. In-situ NMR kinetics run in the NMR tube. ^{19}F-NMR spectra at 333K. 1.5 equiv aniline is used as the nucleophile and 1-bromo-4-(trifluoromethyl)benzene (1.0 equiv) as the electrophile. **Base variation**: 1.3 equiv **cyclohexylamine** and 1.3 equiv ***tert*-butylamine**.
Albeit faster initial conversions, less yield is obtained with cyclohexylamine due to competitive self-coupling.

Table S2: Additive comparison for sulfoximine cross-coupling. Ni: $NiBr_2$·glyme (5 mol%), PC: 4CzIPN (0.5 mol%). h**v**: blue LED. Yield determined with ^{19}F-NMR and fluorobenzene as internal standard.

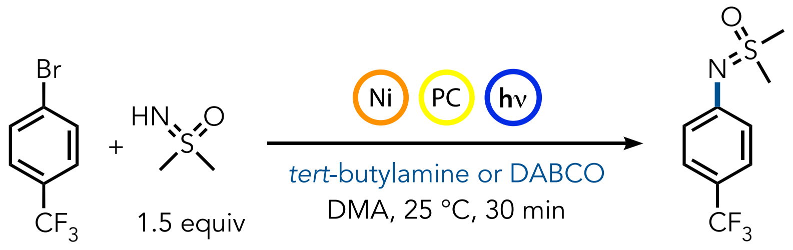

entry	additive	*T* [° C]	time [min]	yield
1	*tert*-butylamine	25	30	100%
2	DABCO (2.2 equiv)	25	30	38%
3	DABCO (1.3 equiv)	25	30	30%
4	none	60	60	10%

3. Additional Control Experiments

Table S3: Control reactions for cross-coupling with phenol. Ni: $NiBr_2$·glyme (5 mol%), PC: 4CzIPN (0.5 mol%). h**v**: blue LED. Yield determined with ^{19}F-NMR and fluorobenzene as internal standard.

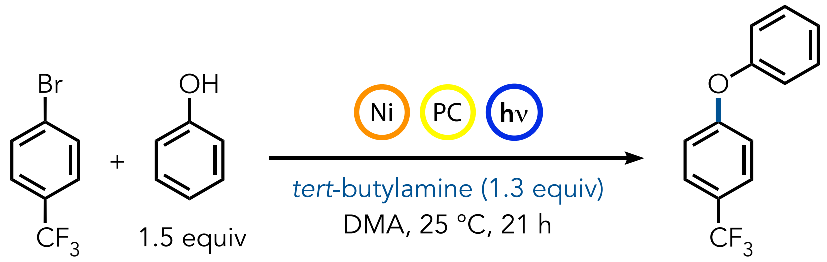

entry	variation from condition	*T* [° C]	time [h]	yield
1	no light	25	21 h	0%
2	no PC	25	21 h	0%
3	no Ni	25	21 h	0%
4	ambient atmosphere (no N_2)	25	17 h	0%

Table S4: Control reactions and catalyst loading variation for sulfoximine cross-coupling. Ni: $NiBr_2$·glyme (5 mol%), PC: 4CzIPN (0.5 mol%). h**v**: blue LED. Yield determined with ^{19}F-NMR and fluorobenzene as internal standard.

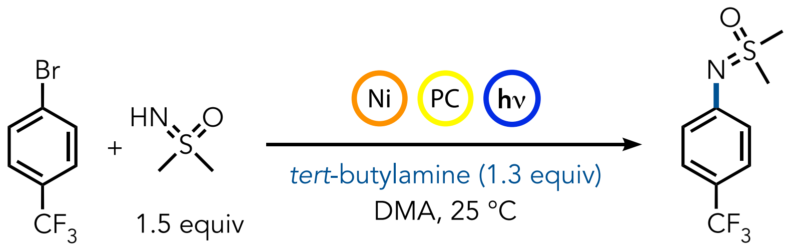

entry	deviation from standard conditions	*T* [° C]	time [h]	yield
1	no photocatalyst	25	1	0%
2	no Ni	25	1	0%
3	no light	25	1	0%
4	2.5 mol% Ni	25	3	95%
5	1.25 mol% Ni	25	3	81%
6	1.0 equiv sulfoximine	25	3	88%
7	ambient atmosphere (no N_2)	25	17	42%

Table S5: Influence of *tert*-butylamine equivalents on phenol cross-coupling. Ni: $NiBr_2$·glyme (5 mol%), PC: 4CzIPN (0.5 mol%). h**v**: blue LED. Yield determined with ^{19}F-NMR and fluorobenzene as internal standard.

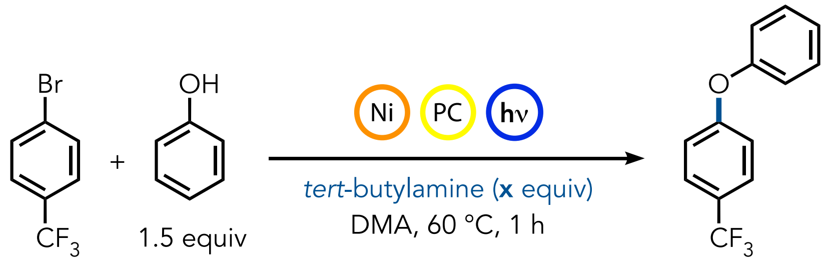

entry	*tert*-butylamine equiv	*T* [° C]	time [h]	yield
1	1.3	60 °C	1 h	54%
2	2.6	60 °C	1 h	41%

Table S6: Influence of *tert*-butylamine equivalents on aniline cross-coupling. Ni: $NiBr_2$·glyme (5 mol%), PC: 4CzIPN (0.5 mol%). h**v**: blue LED. Yield determined with ^{19}F-NMR and fluorobenzene as internal standard.

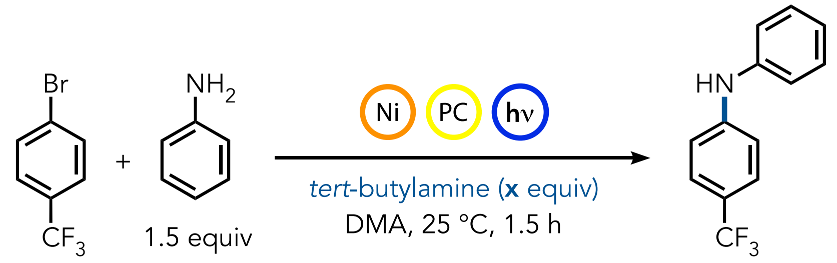

entry	*tert*-butylamine equiv	*T* [° C]	time [h]	yield
1	1.3	25 °C	1.5 h	100%
2	2.6	25 °C	1.5 h	67%

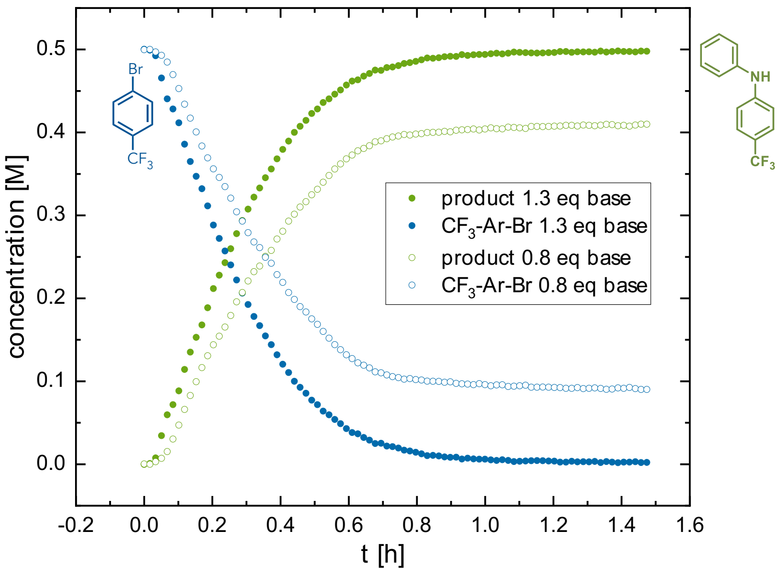

Figure S5: Evaluation of *tert*-butylamine equivalents on reaction performance. In-situ NMR kinetics run in the NMR tube. ^{19}F-NMR spectra at 333K. 1.5 equiv **aniline** is used as the nucleophile and 1-bromo-4-

(trifluoromethyl)benzene (1.0 equiv) as the electrophile. Comparison **0.8 equiv and 1.3 equiv of *tert*-butylamine** as base.
The result showcases that 0.8 equiv of tert-butylamine lead to reduced yields and incomplete conversion.

Table S7: Nickel salt investigation for aniline cross-coupling. PC: 4CzIPN (0.5 mol%). h**v**: blue LED. Yield determined with ^{19}F-NMR and fluorobenzene as internal standard.

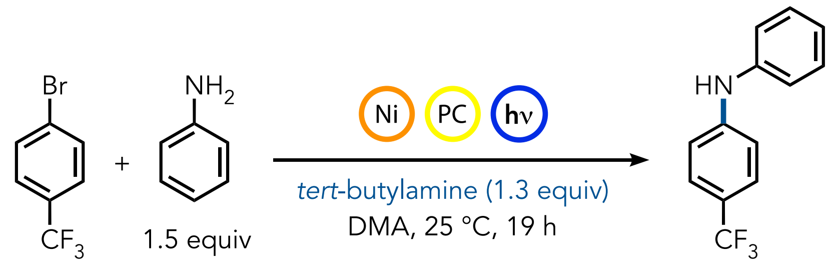

entry	Ni salt	*T* [° C]	time [h]	yield
1	$NiBr_2 \bullet (H_2O)_3$	25	19	99%
2	$NiCl_2 \bullet (H_2O)_6$	25	19	99%
3	$NiCl_2 \bullet$glyme	25	19	99%

4. Competitive Coordination Experiments in NMR

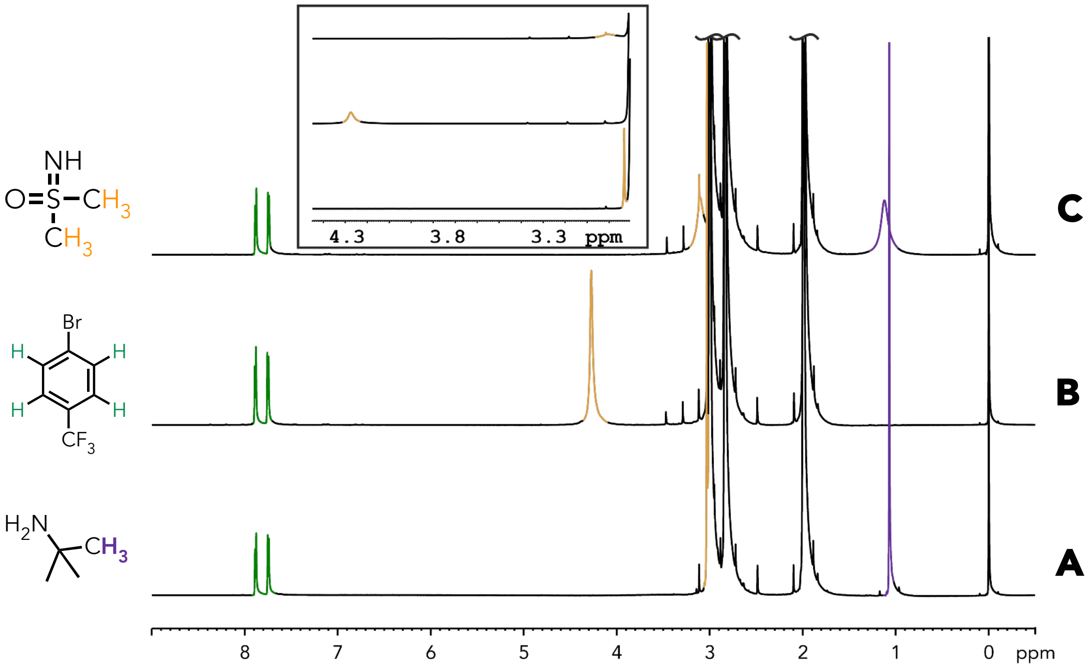

Figure S6: ^{1}H-NMR spectra at 298K to investigate the **coordination** behavior of **(S-methylsulfonimidoyl)methane**. **A**: 0.5 M 1-bromo-4-(trifluoromethyl)benzene, 0.75 M (S-methylsulfonimidoyl)methane and 0.65 M *tert*-butylamine, in DMA (**no catalysts**). **B:** 0.5 M 1-bromo-4-(trifluoromethyl)benzene and 0.75 M (S-methylsulfonimidoyl)methane in DMA with 5 mol% Ni and 0.5 mol% PC (**no *tert*-butylamine**). **C:** 0.5 M 1-bromo-4-(trifluoromethyl)benzene, 0.65 M *tert*-butylamine and 0.75 M (S-methylsulfonimidoyl)methane in DMA with 5 mol% Ni and 0.5 mol% PC (**reaction conditions**).

*The shift of the methyl groups toward higher ppm values indicates the facile coordination of (S-methylsulfonimidoyl)methane to nickel (**B**) in the absence of tert-butylamine.*

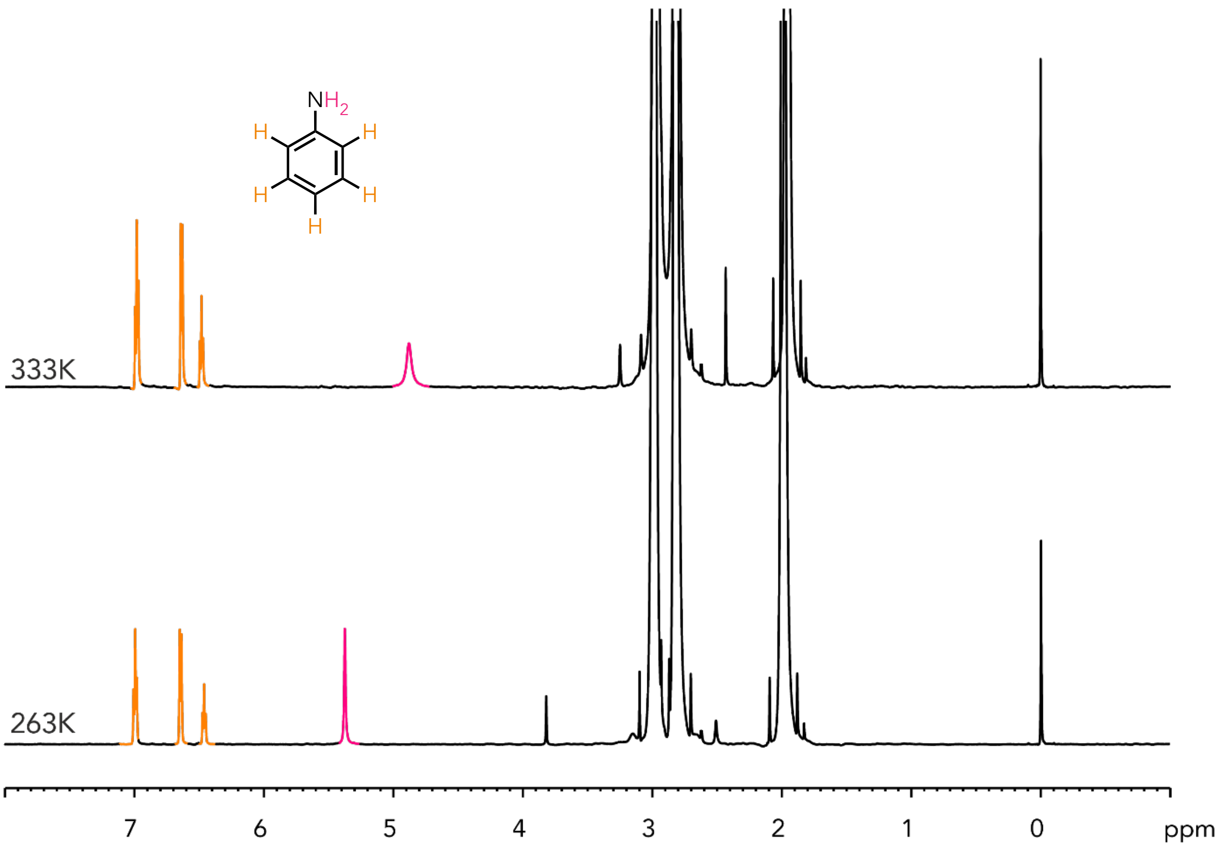

Figure S7: ^{1}H-NMR reference spectra of **aniline** at 263K and 333K to investigate **temperature dependencies**. 0.75 M aniline in DMA (**no catalysts,** with TMS).
The shift and broadening of the nitrogen protons indicates more exchange (with the solvent or other aniline molecules) at higher temperatures.

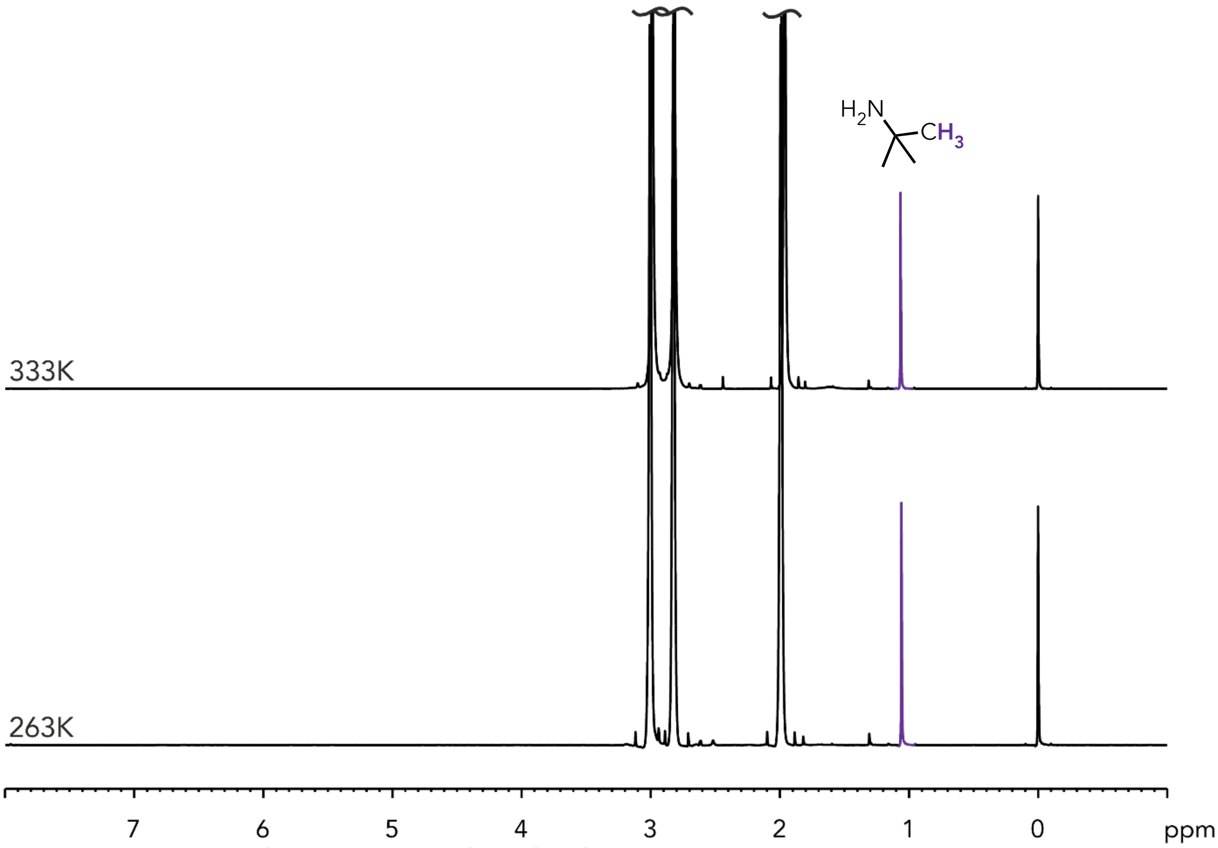

Figure S8: ^{1}H-NMR reference spectra of ***tert*-butylamine** at 263K and 333K to investigate **temperature dependencies**. 0.65 M *tert*-butylamine in DMA (**no catalysts,** with TMS).
No differences between the two spectra can be observed. The tert-butylamine protic hydrogens are either too broad to observe or possibly hidden under the DMA solvent signals.

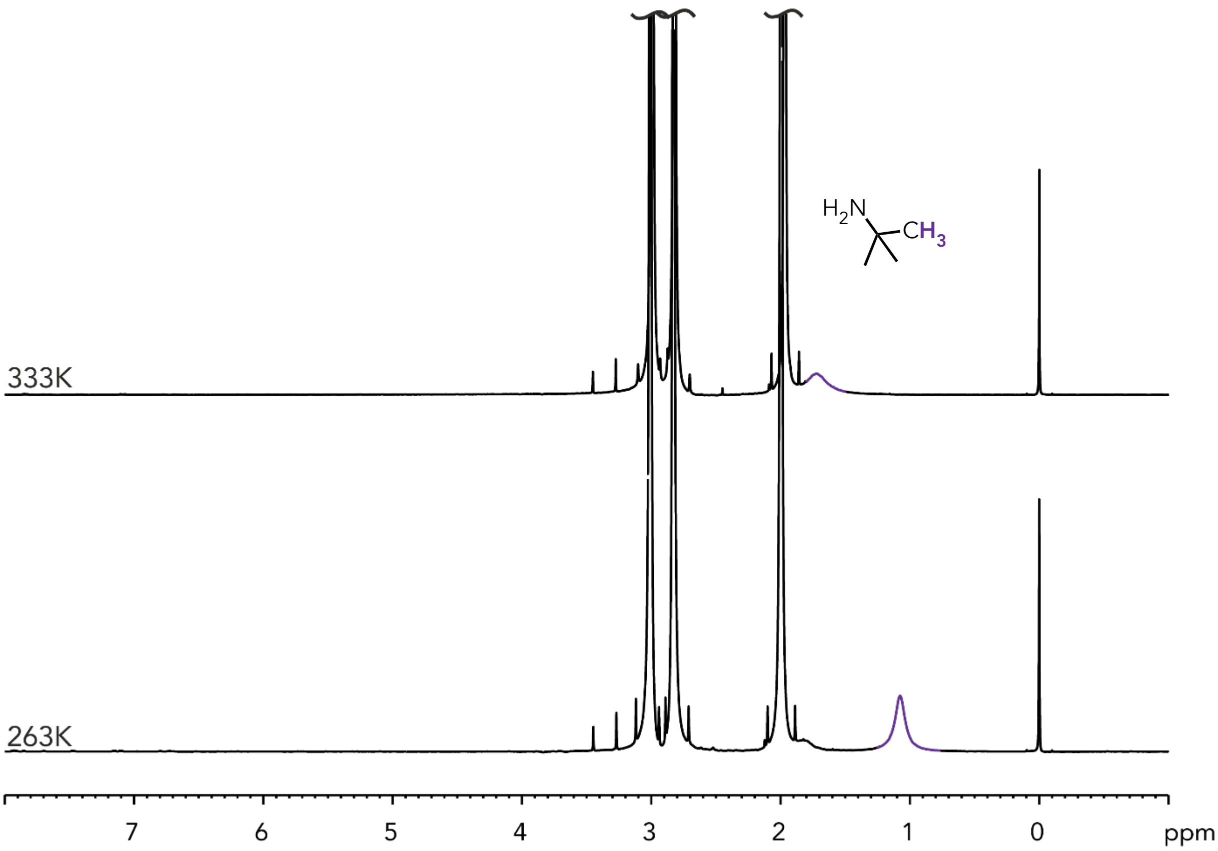

Figure S9: ^{1}H-NMR reference spectra of ***tert*-butylamine** and **nickel** at 263K and 333K to investigate **temperature dependencies** of the *tert*-butylamine coordination to nickel. 0.65 M *tert*-butylamine in DMA with 5.0 mol% Ni and 0.5 mol% PC (with TMS).

The tert-butylamine methyl group protons broaden and shift towards higher ppm values with elevated temperature which suggests coordination of tert-butylamine to nickel and increased exchange rates at higher temperatures.

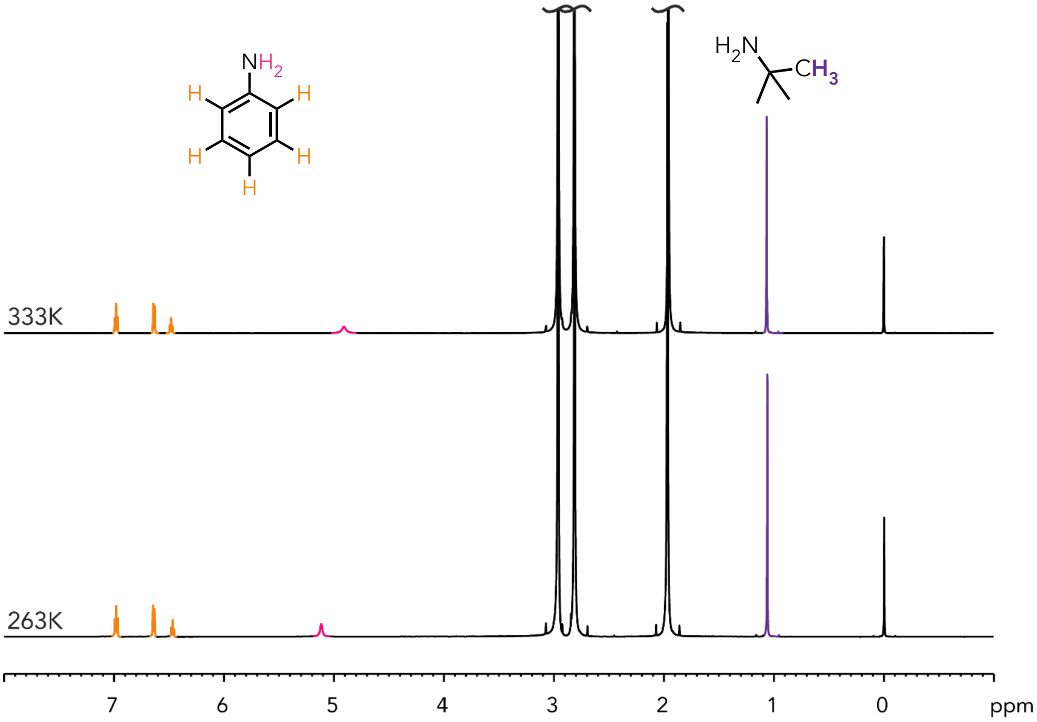

Figure S10: ¹H-NMR reference spectra of **aniline** and ***tert*-butylamine** at 263K and 333K to investigate **temperature dependencies** and **interactions**. 0.75 M aniline and 0.65 M *tert*-butylamine in DMA (**no catalysts,** with TMS).

The shift and broadening of the nitrogen protons is similar as in the solution just containing aniline (Figure S7).

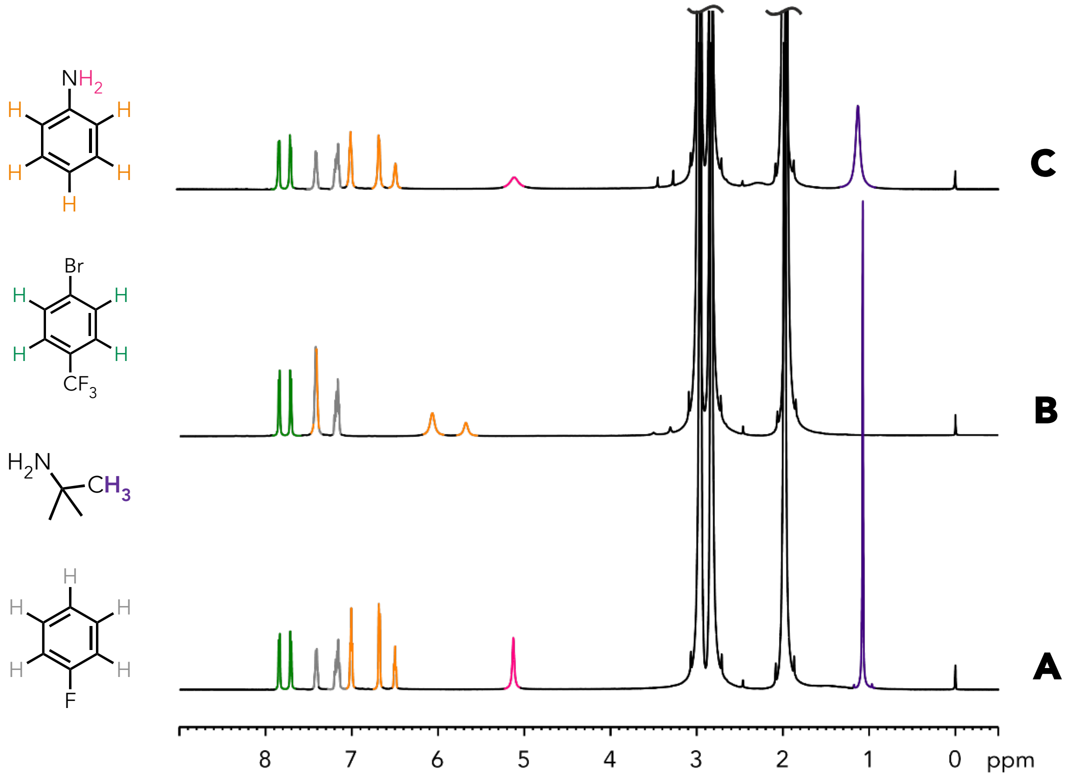

Figure S11: ¹H-NMR spectra at 298K to investigate the **coordination** behavior of **aniline**. **A**: 0.5 M 1-bromo-4-(trifluoromethyl)benzene, 0.75 M **aniline** and 0.65 M ***tert*-butylamine** in DMA (**no catalysts**). **B:** 0.5 M 1-bromo-4-(trifluoromethyl)benzene and 0.75 M aniline in DMA with 5.0 mol% Ni and 0.5 mol% 4CzIPN (**no *tert*-butylamine**). **C:** 0.5 M 1-bromo-4-(trifluoromethyl)benzene, 0.65 M *tert*-butylamine and 0.75 M aniline in DMA with 5.0 mol% Ni and 0.5 mol% 4CzIPN (**reaction conditions**). Fluorobenzene was used as internal reference.

*Labile coordination of aniline to nickel is indicated by the shifted aromatic hydrogen signals of aniline (**B**). However, when both aniline and tert-butylamine are added, tert-butylamine coordinates preferentially as indicated in spectrum **C**.*

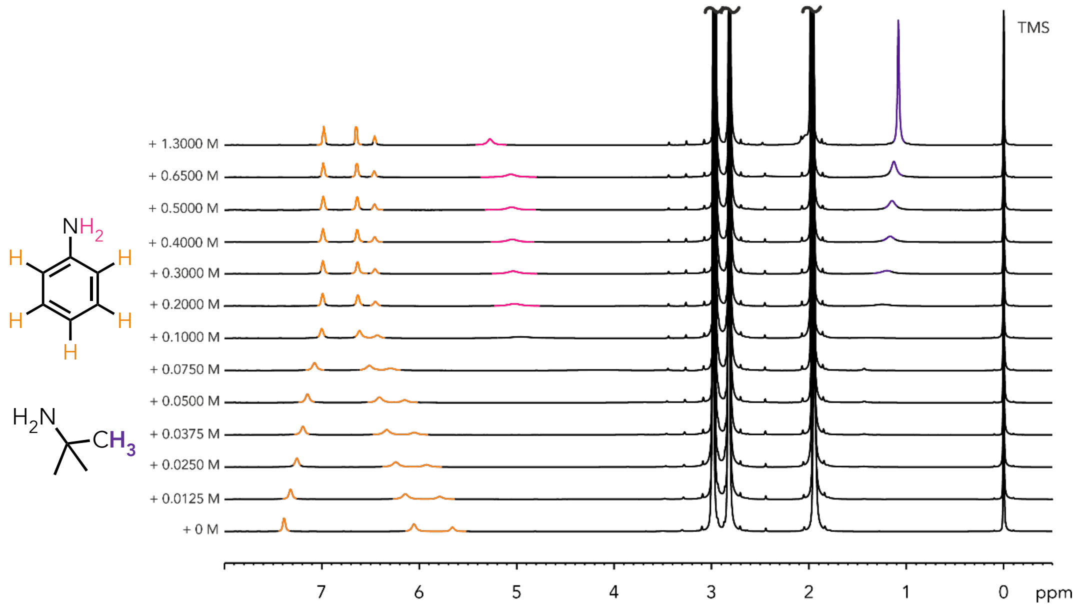

Figure S12: ^{1}H-NMR spectra at 298 K of the **titration** of 0.75 M **aniline** with ***tert*-butylamine** to investigate the coordination behaviour to nickel. The titration was performed under experimental conditions with [nickel] = 0.025 M and [4CzIPN] = 0.0025 M. Titration from 0.0 M to 0.05 M *tert*-butylamine was executed with a 1:1 *tert*-butylamine:DMA stock solution. Any further titration was directly done with *tert*-butylamine.
Addition up to 0.1 M tert-butylamine led to significant chemical shift changes of the aromatic signals of aniline and no visible signal for the added tert-butylamine, which suggests the replacement of the coordinated aniline with tert-butylamine.

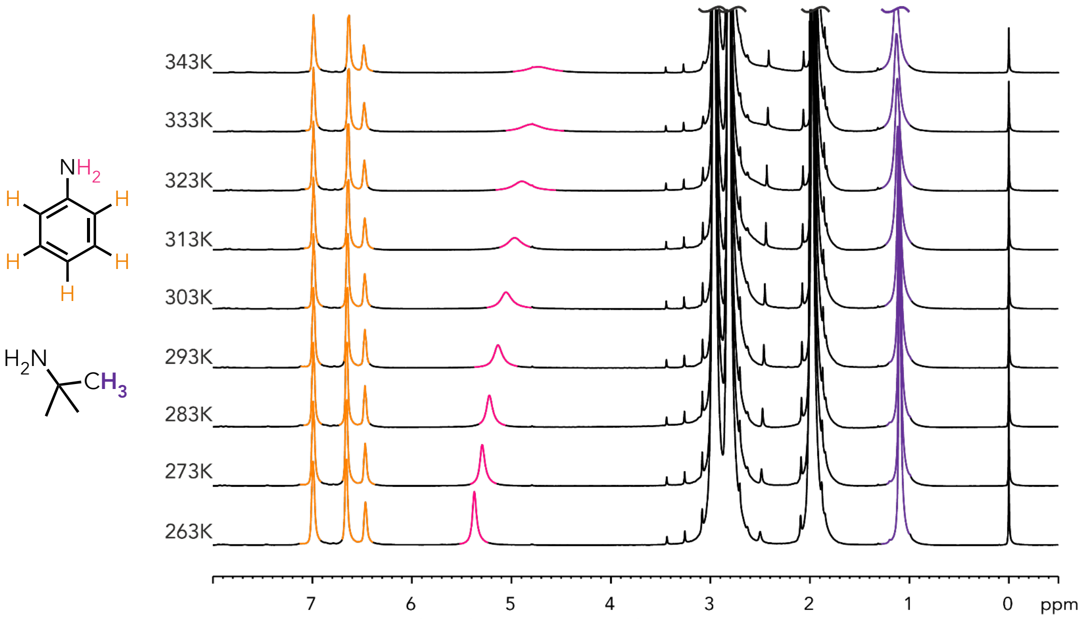

Figure S13: ^{1}H-NMR spectra of ***tert*-butylamine** and **aniline** with nickel at varying temperatures. 0.65 M *tert*-butylamine and 0.75 M aniline in DMA with 5.0 mol% Ni and 0.5 mol% 4CzIPN (**reaction conditions**).
At elevated temperatures, the protic hydrogens of aniline shift to lower ppm values and broaden which is the same behavior as in Figure S7 without nickel.

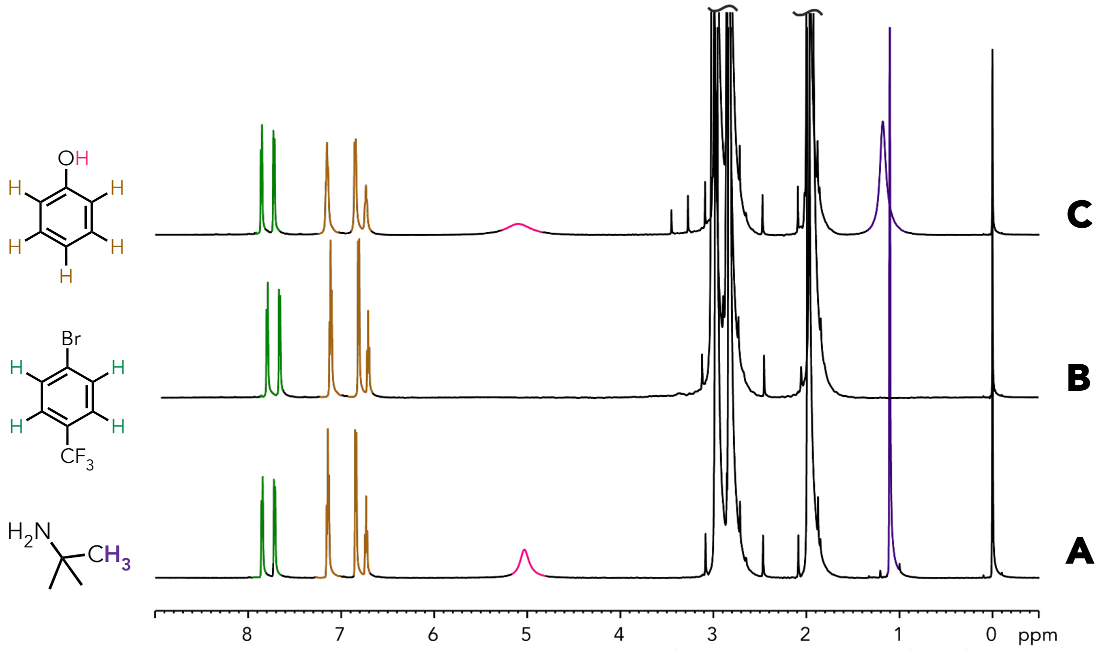

Figure S14: ^{1}H-NMR spectra at 298 K to investigate the **coordination** behavior of **phenol**. **A**: 0.5 M 1-bromo-4-(trifluoromethyl)benzene, 0.75 M phenol and 0.65 M tert-butylamine, in DMA (**no catalysts**). **B:** 0.5 M 1-bromo-4-(trifluoromethyl)benzene and 0.75 M phenol in DMA with 5 mol% Ni and 0.5 mol% PC (**no *tert*-butylamine**). **C:** 0.5 M 1-bromo-4-(trifluoromethyl)benzene, 0.65 M *tert*-butylamine and 0.75 M phenol in DMA with 5 mol% Ni and 0.5 mol% PC (**reaction conditions**).

In contrast to aniline (Figure S11 B), a shift or significant broadening of the aromatic phenol hydrogens cannot be observed, even in the absence of tert-butylamine, indicating a weak coordination tendency toward nickel.

5. NMR Yields of Structurally Diverse Anilines

For the NMR-yields of **Figure 3 (D)**, a 5 mL crimp cap vial was charged with a magnetic stirring bar, 1-bromo-4-(trifluoromethyl)benzene (0.2 mmol, 1.0 equiv), the respective aniline (0.3 mmol, 1.5 equiv) and 0.4 mL of a catalyst stock solution containing 4CzIPN (0.8 mg, 0.001 mmol, 0.005 equiv) and $NiBr_2$•glyme (3.2 mg, 0.01 mmol, 0.05 equiv) dissolved in DMA. The reaction mixture was then degassed and refilled with nitrogen two times via a syringe needle before *tert*-butylamine (27.3 μL, 0.26 mmol, 1.3 equiv) was added via syringe. After degassing one more time and refilling with nitrogen, the reaction mixture was photoirradiated through the plane bottom side of the snap vial using a single blue LED (455 (± 15) nm). Reaction yields were determined with ^{19}F-NMR using fluorobenzene as the internal standard.
Product formation was confirmed with GC-FID and GC-MS analysis.
Note: *Tert*-butylamine is volatile and therefore, it is recommended to not evacuate the reaction mixture to high vacuum for an extended time period.

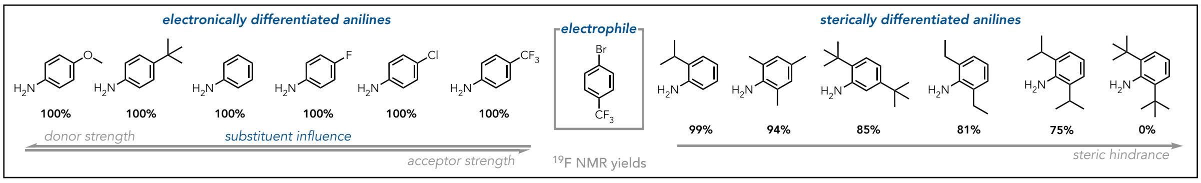

Reaction time and temperature
Electronically differentiated anilines: 25 °C for 13 h.
Sterically differentiated anilines: 60 °C for 17 h.

6. Structure-Reactivity Relationships with Competition Experiments

Experiments for structure-reactivity relationships (Figure 3) were conducted in 5 mL crimp cap vials. The vial was charged with a magnetic stirring bar, (het)aryl halide/s, the respective nucleophile/s and 0.4 mL of a catalyst stock solution containing 4CzIPN (0.8 mg, 0.001 mmol, 0.005 equiv) and $NiBr_2$•glyme (3.2 mg, 0.01 mmol, 0.05 equiv) dissolved in DMA. The reaction mixture was then degassed and refilled with nitrogen two times via a syringe needle before *tert*-butylamine (27.3 μL, 0.26 mmol, 1.3 equiv) was added via syringe. After degassing one more time and refilling with nitrogen, the reaction mixture was photoirradiated through the plane bottom side of the snap vial using a single blue LED (455 (± 15) nm).
Note: *Tert*-butylamine is volatile and therefore, it is recommended to not evacuate the reaction mixture to high vacuum for an extended time period.

The relative rates (k_{rel} ($k_X/k_{reference}$)) correspond to the ratio of products formed in the competition experiment. Relative product formation was determined by ^{19}F-NMR. Product formation was confirmed with GC-FID and GC-MS analysis. The reference product was additionally verified by spiking the reaction mixture with a sample containing the reference product.

Conditions for Anilines (Figure 3, E-G)

Temperature and time: 25 °C for 1 h.

Equivalents: 1.5 equiv (0.75 mmol) of each aniline (aniline as reference in each reaction), 1.0 equiv 1-bromo-4-(trifluoromethyl)benzene (0.5 mmol).

Table S8: Data for aniline plots.

aniline **X**	log(k_{rel})	%Vbur_N1_3.5Å_Boltz	NBO_charge_N1_Boltz
o-*i*Pr aniline	-0.60	45.8	-
2,5-ditertbutylaniline	-1.28	49.8	-
2,4,6-trimethylaniline	-1.03	47.6	-
2,6-diethylaniline	-1.39	51.3	-
2,6-diisopropylaniline	-1.57	53.7	-
p-OMe aniline	0.79	38.1	-0.7995
p-CF3 aniline	-1.09	38.4	-0.7890
p-tertbutyl aniline	0.26	38.2	-0.7978
p-Cl aniline	-0.27	38.3	-0.7946
2-aminopyridine	-0.12	36.8	-0.7969
3-aminopyridine	-0.44	38.2	-0.7947
aniline	0.00	38.3	-0.7968

Conditions for Phenols (Figure 3, J)

Temperature and time: 25 °C for 21 h.

Equivalents: 1.5 equiv (0.75 mmol) of each phenol (phenol as reference in each reaction), 1.0 equiv 1-bromo-4-(trifluoromethyl)benzene (0.5 mmol).

Table S9: Data for phenol plot.

Phenol **X**	log(k_{rel})	NBO_charge_O1_Boltz
p-CF_3 phenol	-0.26	-0.6510
p-*t*Bu phenol	0.16	-0.6609
p-OMe phenol	0.25	-0.6633
p-CO_2Me phenol	-0.24	-0.6512
phenol	0.00	-0.6597

Conditions for Aryl Bromides (Figure 3, H)

Temperature and time: 60 °C for 3 h.

Equivalents: 1.0 equiv (0.5 mmol) of each ArBr (bromobenzene as reference in each reaction), 1.0 equiv (0.5 mmol) 4-fluoroaniline.

Table S10: Data for ArX plot.

Bromobenzene **X**	log(k_{rel})	Mulliken electronegativity_Boltz
p-CF_3 bromobenzene	0.78	0.171
p-propyl bromobenzene	-0.10	0.141

p-Cl bromobenzene	0.41	0.154
3-bromopyridine	0.79	0.164
p-OMe bromobenzene	-0.29	0.136
bromobenzene	0.00	0.147

Two exemplary competition reactions were performed with in-situ NMR monitoring to track reaction progress with two different nucleophile concentrations:

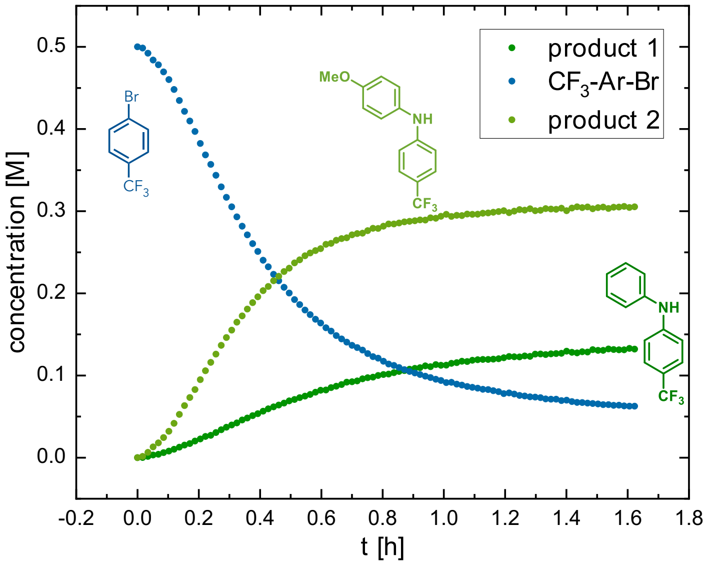

Figure S15: Competition experiments. In-situ NMR kinetics run in the NMR tube. ^{19}F-NMR spectra at 333K. **0.75 equiv** of aniline and **0.75 equiv** 4-anisidine were used as nucleophiles and 1-bromo-4-(trifluoromethyl)benzene (1.0 equiv) as the electrophile.

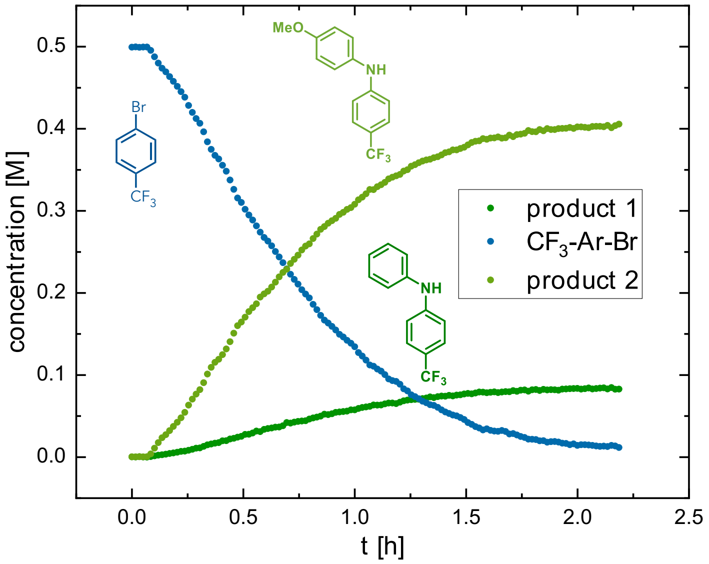

Figure S16: Competition experiments. In-situ NMR kinetics run in the NMR tube. ^{19}F-NMR spectra at 333K. **1.5 equiv** aniline and **1.5 equiv** 4-anisidine were used as nucleophiles and 1-bromo-4-(trifluoromethyl)benzene (1.0 equiv) as the electrophile.

7. Structure-Reactivity with Individual Experiments

For the individual vial experiments with phenols, a 5 mL crimp cap vial was charged with a magnetic stirring bar, 1-bromo-4-(trifluoromethyl)benzene (0.2 mmol, 1.0 equiv), the respective phenol (0.3 mmol, 1.5 equiv) and 0.4 mL of a catalyst stock solution containing 4CzIPN (0.8 mg, 0.001 mmol, 0.005 equiv) and $NiBr_2$•glyme (3.2 mg, 0.01 mmol, 0.05 equiv) dissolved in DMA. The reaction mixture was then degassed and refilled with nitrogen two times via a syringe needle before *tert*-butylamine (27.3 µL, 0.26 mmol, 1.3 equiv) was added via syringe. After degassing one more time and refilling with nitrogen, the reaction mixture was photoirradiated through the plane bottom side of the snap vial using a single blue LED (455 (± 15) nm). The reactions were run in parallel for 2 h at 25 °C. Reaction yields were determined with ^{19}F-NMR using fluorobenzene as the internal standard.

Note: *Tert*-butylamine is volatile and therefore, it is recommended to not evacuate the reaction mixture to high vacuum for an extended time period.

Note: The phenols were dried over P_2O_5 in a desiccator to remove residual water.

k_{rel} corresponds to the ratio of ^{19}F-NMR yields after 2 h with phenol as the reference. Hammett values were obtained from the literature.[5]

Table S11: Data for individual phenol plot.

Phenol **X**	yield after 2 h	log(k_{rel})	NBO_charge_O1_Boltz
p-*t*Bu phenol	79%	0.32	-0.6609
phenol	38%	0.00	-0.6597
p-CO_2Me phenol	18%	-0.32	-0.6512

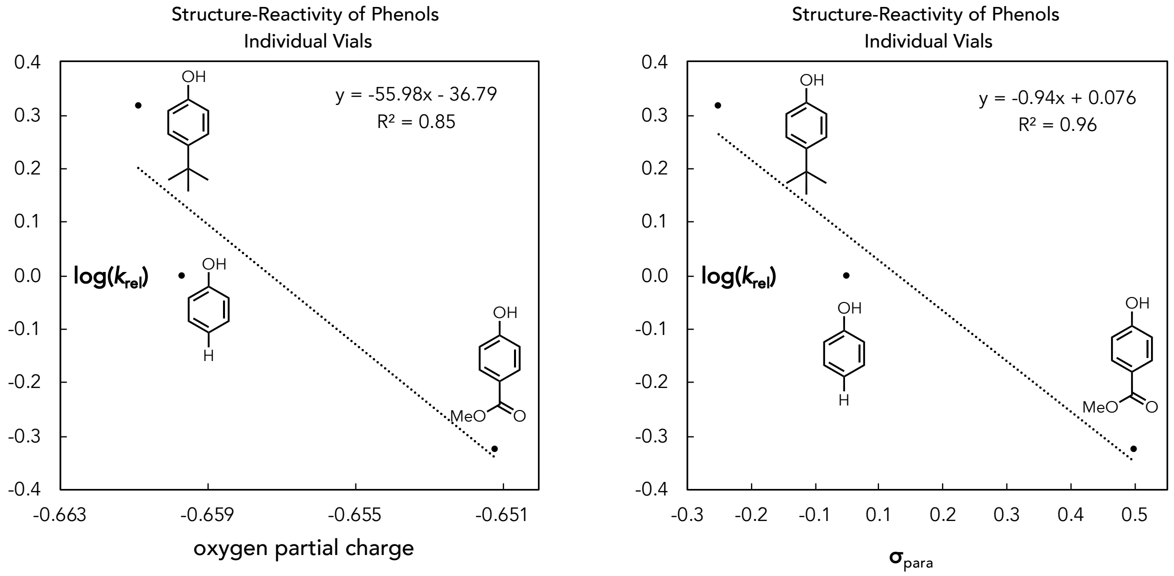

Figure S17: Structure-reactivity relationships of phenols in individual vials.

8. Further In-Situ Kinetics

Additional in-situ NMR kinetics were performed to show the efficiency of reactions with different nucleophiles in the NMR tube:

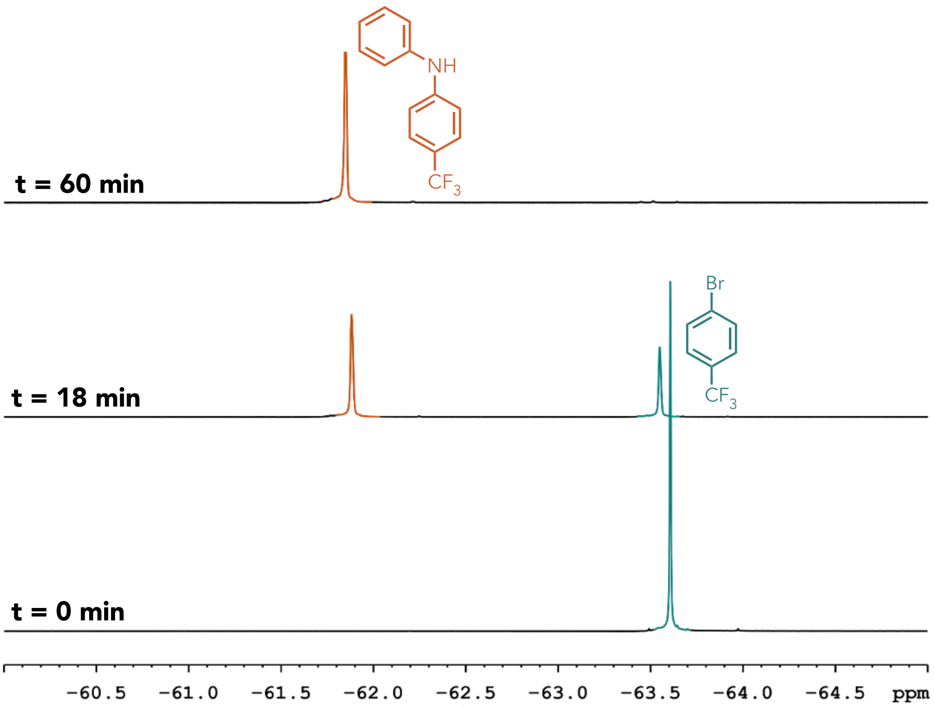

Figure S18: In-situ NMR kinetics run in the NMR tube. ^{19}F-NMR spectra at 333K. Exemplary reaction with **aniline** (1.5 equiv) as a nucleophile and 1-bromo-4-(trifluoromethyl)benzene (1.0 equiv) as the electrophile. Shown is the region of interest where the starting material (blue) declines and the product (orange) forms.

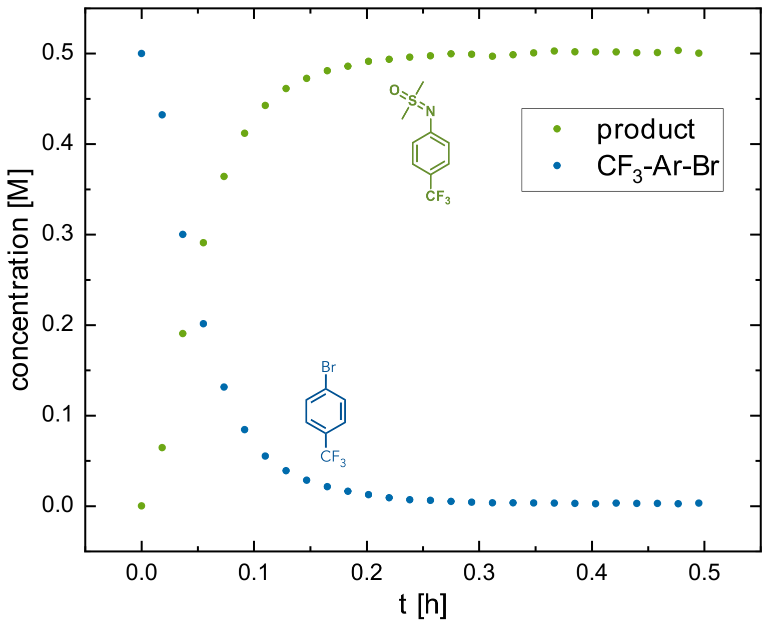

Figure S19: In-situ NMR kinetics run in the NMR tube. ^{19}F-NMR spectra at 333K. 1.5 equiv **(S-methylsulfonimidoyl)methane** as the nucleophile and 1-bromo-4-(trifluoromethyl)benzene (1.0 equiv) as the electrophile.

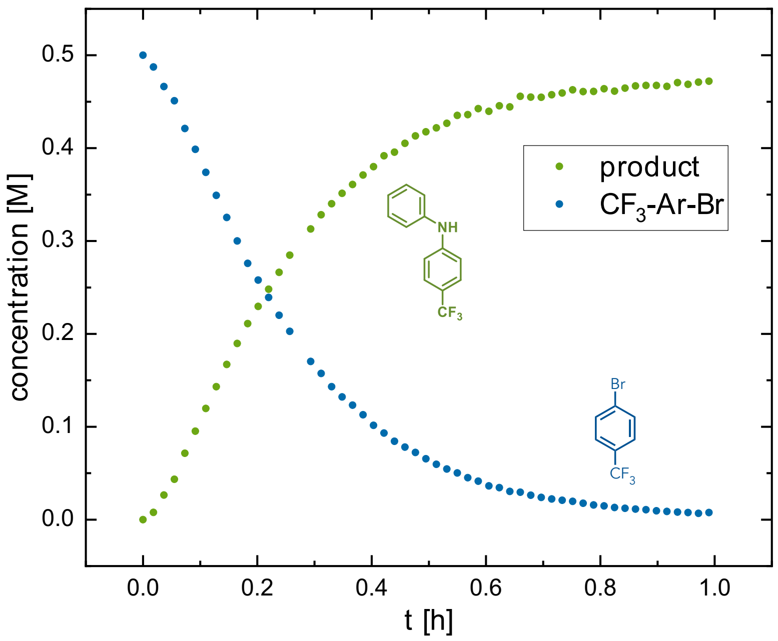

Figure S20: In-situ NMR kinetics run in the NMR tube. ^{19}F-NMR spectra at 333K. 1.5 equiv **aniline** used as nucleophile and 1-bromo-4-(trifluoromethyl)benzene (1.0 equiv) as the electrophile.

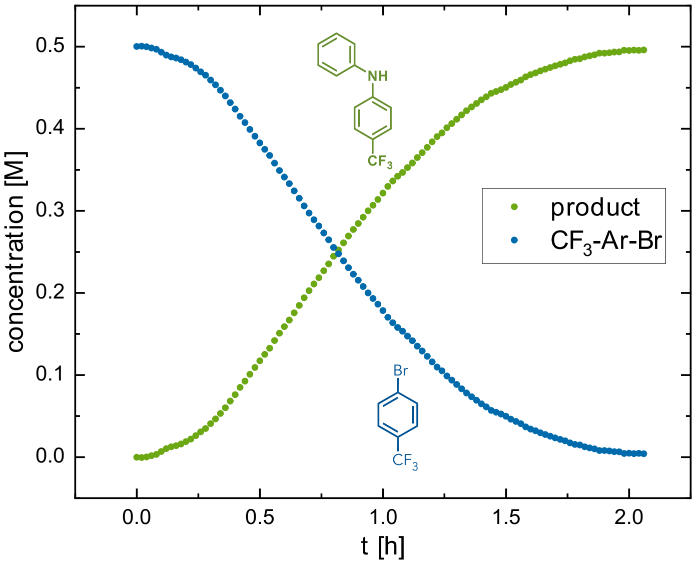

Figure S21: In-situ NMR kinetics run in the NMR tube. ^{19}F-NMR spectra **at 298K**. 1.5 equiv aniline used as nucleophile and 1-bromo-4-(trifluoromethyl)benzene (1.0 equiv) as the electrophile. **Temperature variation**: **298K** instead of **333K**.

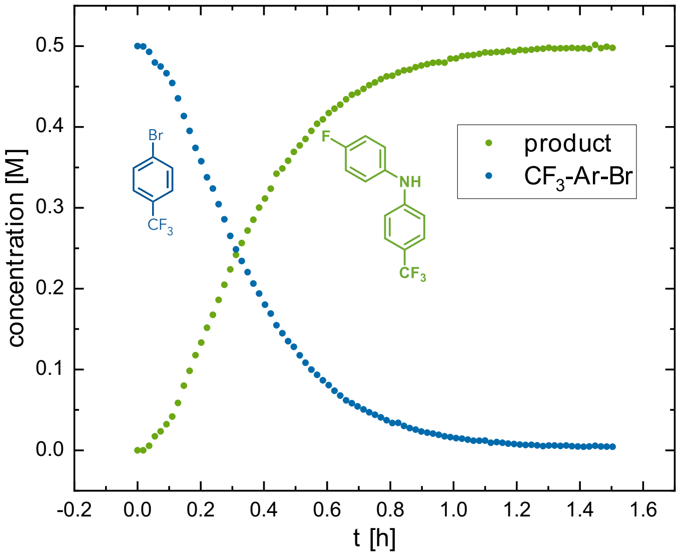

Figure S22: In-situ NMR kinetics run in the NMR tube. ^{19}F-NMR spectra at 333K. 1.5 equiv **4-fluoroaniline** used as nucleophile and 1-bromo-4-(trifluoromethyl)benzene (1.0 equiv) as the electrophile.

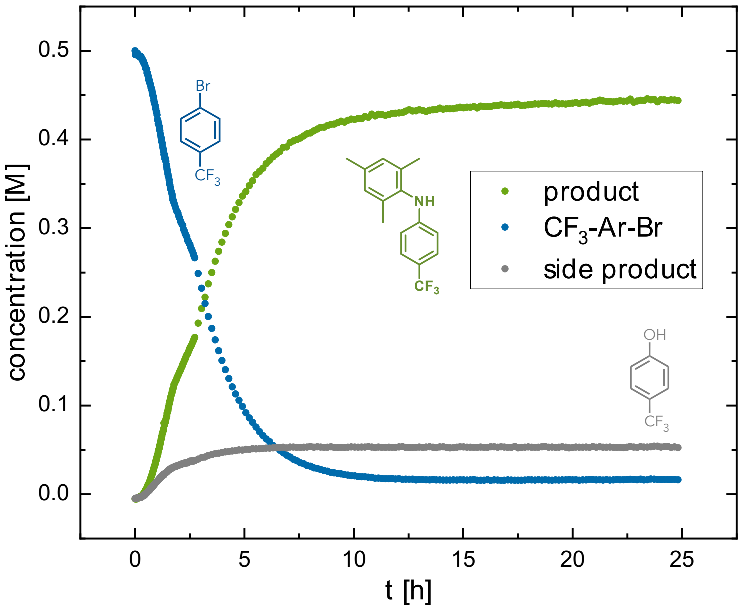

Figure S23: In-situ NMR kinetics run in the NMR tube. ^{19}F-NMR spectra at 333K. 1.5 equiv **2,4,6-trimethylaniline** used as nucleophile and 1-bromo-4-(trifluoromethyl)benzene (1.0 equiv) as the electrophile.

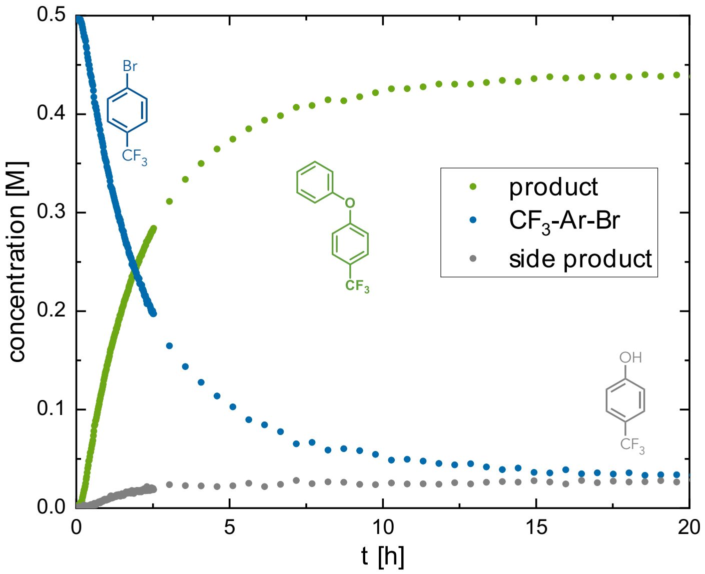

Figure S24: In-situ NMR kinetics run in the NMR tube. ^{19}F-NMR spectra at 333K. 1.5 equiv **phenol** used as nucleophile and 1-bromo-4-(trifluoromethyl)benzene (1.0 equiv) as the electrophile.

9. Experimental Procedures

General procedure: A 5 mL crimp top vial was charged with a magnetic stirring bar, (het)aryl halide (0.2 mmol, 1.0 equiv), the respective nucleophile (0.3 mmol, 1.5 equiv) and 0.4 mL of a catalyst stock solution containing 4CzIPN (0.8 mg, 0.001 mmol, 0.005 equiv) and $NiBr_2$•glyme (3.2 mg, 0.01 mmol, 0.05 equiv) dissolved in DMA. The reaction mixture was then degassed and refilled with nitrogen two times via a syringe needle before *tert*-butylamine (27.3 µL, 0.26 mmol, 1.3 equiv) was added via syringe. After degassing one more time and refilling with nitrogen, the reaction mixture was photoirradiated through the plane bottom side of the snap vial using a single blue LED (455 (± 15) nm).

Note: *Tert*-butylamine is volatile and therefore, it is recommended to not evacuate the reaction mixture to high vacuum for an extended time period.

General difunctionalization procedure:

First step: A 5 mL crimp top vial was charged with a magnetic stirring bar, (het)aryl halide (0.2 mmol, 1.0 equiv), the respective nucleophile (1.05 equiv–1.1 equiv) and 0.4 mL of a catalyst stock solution containing 4CzIPN (0.8 mg, 0.001 mmol, 0.005 equiv) and $NiBr_2$•glyme (3.2 mg, 0.01 mmol, 0.05 equiv) dissolved in DMA. The reaction mixture was then degassed and refilled with nitrogen two times via a syringe needle before *tert*-butylamine (27.3 µL, 0.26 mmol, 1.3 equiv) was added via syringe. After degassing one more time and refilling with nitrogen, the reaction was photoirradiated through the plane bottom side of the snap vial using a single blue LED (455 (± 15) nm).

Second Step: After 2–5 h irradiation at 25 °C or 60 °C, the second nucleophile (1.5 equiv) was added, the reaction mixture was degassed and refilled with nitrogen before *tert*-butylamine (1.3 equiv) was added and the reaction mixture was degassed one more time and refilled with nitrogen before irradiation through the plane bottom side of the snap vial using a single blue LED (455 (± 15) nm).

Note 1: *Tert*-butylamine is volatile and therefore, it is recommended to not evacuate the reaction mixture to high vacuum for an extended time period.

Note 2: When pyrrolidine was used in the second step, the second addition of *tert*-butylamine is not required, but 3.0 equiv of pyrrolidine were used instead of 1.5 equiv.[6]

General workup procedure:

After completion of the reaction, the reaction mixture was transferred to a separating funnel with ethyl acetate (approx. 2 mL). The reaction vial was rinsed two more times with ethyl acetate and one time with water (approx. 2 mL EtOAc and H_2O) and added to the separating funnel as well. Then, approx. 10 mL water and 20 mL EtOAc were added to the separating funnel. After extraction, the organic layer was collected, and the water layer was extracted two more times with ethyl acetate (25 mL) (petrol ether for more volatile biaryl ethers). The combined organic layers were dried over Na_2SO_4, filtered, and concentrated in vacuo. The crude product was purified by flash column chromatography using petrol ether/ethyl acetate and/or ethyl acetate/methanol.

Note 1: For the more volatile biaryl ethers, it is recommended to do the workup and extraction with petrol ether instead of ethyl acetate.

Note 2: For the products of sulfoximine cross-coupling as well as the difunctionalization products, dimethylacetamide was removed in vacuo before the crude product was purified by flash column chromatography using petrol ether/ethyl acetate and/or ethyl acetate/methanol.

Determination of reaction progress

The reaction progress (in this case, mainly substrate consumption and the formation of the desired product) was monitored by GC-FID analysis.

ethyl 4-(4-(methoxycarbonyl)phenoxy)benzoate (1):

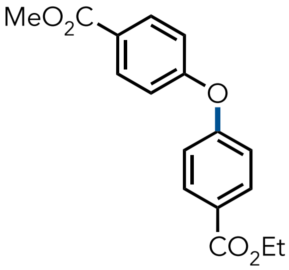

Compound **1** was prepared following **general procedure** using ethyl 4-bromobenzoate (45.8 mg, 0.2 mmol, 1.0 equiv) and methyl 4-hydroxybenzoate (45.6 mg, 0.3 mmol, 1.5 equiv). The reaction mixture was photoirradiated under N_2 at 60 °C for 15 h. After completion, the reaction mixture was subjected to the **general work-up procedure** and purified using flash chromatography (gradient to 5%–10% EtOAc in PE) on silica gel. The title compound was obtained in 64% yield (38.4 mg).

^{1}H NMR (400 MHz, $CDCl_3$) δ 8.12 – 8.01 (m, 4H), 7.12 – 7.01 (m, 4H), 4.38 (q, *J* = 7.1 Hz, 2H), 3.91 (s, 3H), 1.39 (t, *J* = 7.1 Hz, 3H).

^{13}C NMR (101 MHz, $CDCl_3$) δ 166.4, 165.9, 160.2, 160.0, 131.8, 131.8, 126.1, 125.6, 118.7, 118.5, 61.0, 52.1, 14.3.

The spectroscopic data are consistent with the literature.[6]

1-phenoxy-4-(trifluoromethyl)benzene (2)

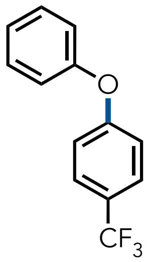

Compound **2** was prepared following **general procedure** using 1-bromo-4-(trifluoromethyl)benzene (45.0 mg, 0.2 mmol, 1.0 equiv) and phenol (28.2 mg, 0.3 mmol, 1.5 equiv). The reaction mixture was photoirradiated under N_2 at 25 °C for 18 h. After completion, the reaction mixture was subjected to the **general work-up procedure** and purified using flash chromatography (100% petroleum ether) on silica gel. The title compound was obtained in 84% yield (40.0 mg).

Note: A nucleophile stock solution in DMA was dried over molecular sieves to reduce the water content of the nucleophile. The reaction works without this precaution, but water coupling can diminish the yield.

^{1}H NMR (400 MHz, $CDCl_3$) δ 7.65 – 7.55 (m, 2H), 7.44 – 7.36 (m, 2H), 7.23 – 7.17 (m, 1H), 7.10 – 7.01 (m, 4H).

^{13}C NMR (101 MHz, $CDCl_3$) δ 160.6, 155.8, 130.2, 127.2 (q, *J* = 3.8 Hz), 125.0 (q, *J* = 32.5 Hz), 124.3 (q, *J* = 271.4 Hz), 124.6, 120.0, 118.0.

^{19}F NMR (377 MHz, $CDCl_3$) δ -62.27.

The spectroscopic data are consistent with the literature.[7]

1-methoxy-4-(4-(trifluoromethyl)phenoxy)benzene (3)

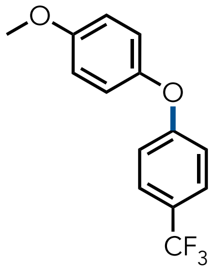

Compound **3** was prepared following **general procedure** using 1-bromo-4-(trifluoromethyl)benzene (45.0 mg, 0.2 mmol, 1.0 equiv) and 4-methoxyphenol (37.2 mg, 0.3 mmol, 1.5 equiv). The reaction mixture was photoirradiated under N_2 at 25 °C for 18 h. After completion, the reaction mixture was subjected to the **general work-up procedure** and purified using flash chromatography (100% petroleum ether) on silica gel. The title compound was obtained in 76% yield (40.8 mg). **Note:** A nucleophile stock solution in DMA was dried over molecular sieves to reduce the water content of the nucleophile. The reaction works without this precaution, but water coupling can diminish the yield.

^{1}H NMR (400 MHz, $CDCl_3$) δ 7.59 – 7.51 (m, 2H), 7.07 – 6.96 (m, 4H), 6.95 – 6.87 (m, 2H), 3.83 (s, 3H).

^{13}C NMR (101 MHz, $CDCl_3$) δ 161.6, 156.8, 148.8, 127.1 (q, *J* = 3.8 Hz), 124.4 (q, *J* = 271.4 Hz), 124.3 (q, *J* = 32.9 Hz), 121.7, 116.9, 115.2, 55.7.

^{19}F NMR (377 MHz, $CDCl_3$) δ -62.18.

The spectroscopic data are consistent with the literature.[8]

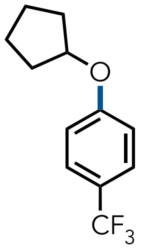

1-(cyclopentyloxy)-4-(trifluoromethyl)benzene (4)

Compound **4** was prepared following **general procedure** using 1-bromo-4-(trifluoromethyl)benzene (45.0 mg, 0.2 mmol, 1.0 equiv) and cyclopentanol (25.8 mg, 0.3 mmol, 1.5 equiv). The reaction mixture was photoirradiated under N_2 at 25 °C for 18 h. After completion, the reaction mixture was subjected to the **general work-up procedure** and purified using flash chromatography (100% petroleum ether) on silica gel. The title compound was obtained in 72% yield (33.2 mg).

Note: A nucleophile stock solution in DMA was dried over molecular sieves to reduce the water content of the nucleophile. The reaction works without this precaution, but water coupling can diminish the yield.

^{1}H NMR (400 MHz, $CDCl_3$) δ 7.52 (d, *J* = 8.7 Hz, 2H), 6.92 (d, *J* = 8.5 Hz, 2H), 4.91 – 4.70 (m, 1H), 2.02 – 1.74 (m, 6H), 1.70 – 1.58 (m, 2H).

^{13}C NMR (101 MHz, $CDCl_3$) δ 160.8, 126.9 (q, *J* = 3.8 Hz), 124.7 (q, *J* = 270.9 Hz), 122.4 (q, *J* = 32.7 Hz), 115.4, 79.7, 32.9, 24.1.

^{19}F NMR (377 MHz, $CDCl_3$) δ -61.93.

The spectroscopic data are consistent with the literature.[9]

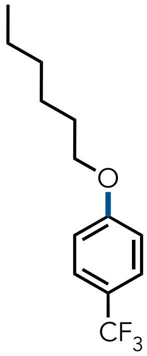

1-(hexyloxy)-4-(trifluoromethyl)benzene (5)

Compound **5** was prepared following **general procedure** using 1-bromo-4-(trifluoromethyl)benzene (45.0 mg, 0.2 mmol, 1.0 equiv) and hexan-1-ol (30.7 mg, 0.3 mmol, 1.5 equiv). The reaction mixture was photoirradiated under N_2 at 25 °C for 18 h. After completion, the reaction mixture was subjected to the **general work-up procedure** and purified using flash chromatography (100% petroleum ether) on silica gel. The title compound was obtained in 83% yield (40.9 mg).

^{1}H NMR (400 MHz, $CDCl_3$) δ 7.49 – 7.41 (m, 2H), 6.91 – 6.83 (m, 2H), 3.91 (t, *J* = 6.6 Hz, 2H), 1.78 – 1.66 (m, 2H), 1.45 – 1.33 (m, 2H), 1.31 – 1.24 (m, 4H), 0.87 – 0.80 (m, 3H).

^{13}C NMR (101 MHz, $CDCl_3$) δ 161.7, 126.9 (q, *J* = 3.7 Hz), 124.6 (q, *J* = 271.2 Hz), 122.7 (q, *J* = 32.5 Hz), 114.5, 68.4, 31.6, 29.2, 25.8, 22.7, 14.1.

^{19}F NMR (376 MHz, $CDCl_3$) δ -61.96.

The spectroscopic data are consistent with the literature.[10]

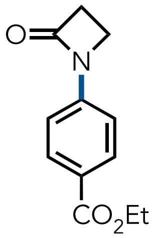

ethyl 4-(2-oxoazetidin-1-yl)benzoate (6)

Compound **6** was prepared following **general procedure** using ethyl 4-bromobenzoate (45.8 mg, 0.2 mmol, 1.0 equiv) and azetidin-2-one (21.3 mg, 0.3 mmol, 1.5 equiv). The reaction mixture was photoirradiated under N_2 at 60 °C for 14 h. After completion, the reaction mixture was subjected to the **general work-up procedure** and purified using flash chromatography (gradient to 30% EtOAc in PE) on silica gel. The title compound was obtained in 75% yield (32.9 mg).

^{1}H NMR (400 MHz, $CDCl_3$) δ 8.04 – 7.96 (m, 2H), 7.40 – 7.32 (m, 2H), 4.34 (q, *J* = 7.1 Hz, 2H), 3.66 (t, *J* = 4.6 Hz, 2H), 3.14 (t, *J* = 4.6 Hz, 2H), 1.37 (t, *J* = 7.1 Hz, 3H).

^{13}C NMR (101 MHz, $CDCl_3$) δ 166.1, 164.8, 142.0, 131.0, 125.6, 115.6, 60.9, 38.3, 36.5, 14.4.

The spectroscopic data are consistent with the literature.[6]

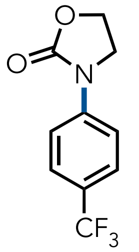

3-(4-(trifluoromethyl)phenyl)oxazolidin-2-one (7)

Compound **7** was prepared following **general procedure** using 1-bromo-4-(trifluoromethyl)benzene (45.0 mg, 0.2 mmol, 1.0 equiv) and oxazolidin-2-one (26.1 mg, 0.3 mmol, 1.5 equiv). The reaction mixture was photoirradiated under N_2 at 60 °C for 14 h. After completion, the reaction mixture was subjected to the **general work-up procedure** and purified using flash chromatography (gradient to 30% EtOAc in PE) on silica gel. The title compound was obtained in 74% yield (34.2 mg).

Note: A nucleophile stock solution in DMA was dried over molecular sieves to reduce the water content of the nucleophile. The reaction works without this precaution, but water coupling can diminish the yield.

^{1}H NMR (400 MHz, $CDCl_3$) δ 7.69 – 7.64 (m, 2H), 7.61 (m, 2H), 4.56 – 4.47 (m, 2H), 4.13 – 4.04 (m, 2H).

^{13}C NMR (101 MHz, $CDCl_3$) δ 155.0, 141.3, 126.3 (q, *J* = 3.8 Hz), 125.8 (q, *J* = 32.8 Hz), 124.1 (q, *J* = 271.3 Hz), 117.7, 61.4, 45.0.

^{19}F NMR (377 MHz, $CDCl_3$) δ -62.65.

The spectroscopic data are consistent with the literature.[11]

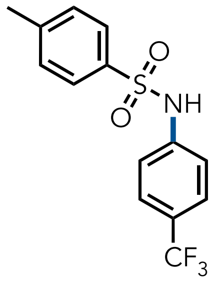

4-methyl-*N*-(4-(trifluoromethyl)phenyl)benzenesulfonamide (8)

Compound **8** was prepared following **general procedure** using 1-bromo-4-(trifluoromethyl)benzene (45.0 mg, 0.2 mmol, 1.0 equiv) and 4-methylbenzenesulfonamide (51.4 mg, 0.3 mmol, 1.5 equiv). The reaction mixture was photoirradiated under N_2 at 25 °C for 14 h. After completion, the reaction mixture was subjected to the **general work-up procedure** and purified using flash chromatography (20% EtOAc in PE) on silica gel. The title compound was obtained in 85% yield (53.6 mg).

Note: A nucleophile stock solution in DMA was dried over molecular sieves to reduce the water content of the nucleophile. The reaction works without this precaution, but water coupling can diminish the yield.

^{1}H NMR (400 MHz, $CDCl_3$) δ 7.80 – 7.74 (m, 2H), 7.73 (s, 1H), 7.52 – 7.43 (m, 2H), 7.26 (d, *J* = 8.1 Hz, 2H), 7.23 – 7.16 (m, 2H), 2.38 (s, 3H).

^{13}C NMR (101 MHz, $CDCl_3$) δ 144.7, 140.1, 135.7, 130.0, 127.3, 126.7 (q, *J* = 3.8 Hz), 126.7 (q, *J* = 33.2 Hz), 124.0 (q, *J* = 271.8 Hz), 119.7, 21.6.

^{19}F NMR (377 MHz, $CDCl_3$) δ -62.77.

The spectroscopic data are consistent with the literature.[12]

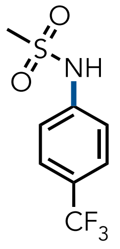

N-(4-(trifluoromethyl)phenyl)methanesulfonamide (**9**)

Compound **9** was prepared following **general procedure** using 1-bromo-4-(trifluoromethyl)benzene (45.0 mg, 0.2 mmol, 1.0 equiv) and methanesulfonamide (28.5 mg, 0.3 mmol, 1.5 equiv). The reaction mixture was photoirradiated under N_2 at 25 °C for 6 h. After completion, the reaction mixture was subjected to the **general work-up procedure** and purified using flash chromatography (gradient to 20% EtOAc in PE) on silica gel. The title compound was obtained in 72% yield (34.4 mg).

Note: A nucleophile stock solution in DMA was dried over molecular sieves to reduce the water content of the nucleophile. The reaction works without this precaution, but water coupling can diminish the yield.

^{1}H NMR (400 MHz, $CDCl_3$) δ′ 7.64 – 7.57 (m, 2H), 7.40 (s, 1H), 7.36 – 7.30 (m, 2H), 3.09 (s, 3H).

^{13}C NMR (101 MHz, $CDCl_3$) δ 140.2, 127.1 (q, *J* = 3.7 Hz), 127.0 (q, *J* = 32.7 Hz), 124.0 (q, *J* = 272.7 Hz), 119.2, 40.0.

^{19}F NMR (377 MHz, $CDCl_3$) δ -62.80.

The spectroscopic data are consistent with the literature.[13]

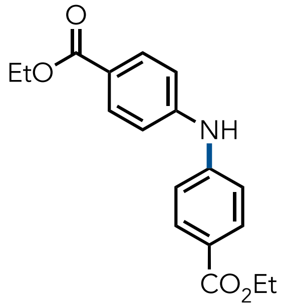

diethyl 4,4'-azanediyldibenzoate (10)

Compound **10** was prepared following **general procedure** using ethyl 4-bromobenzoate (45.8 mg, 0.2 mmol, 1.0 equiv) and ethyl 4-aminobenzoate (49.6 mg, 0.3 mmol, 1.5 equiv). The reaction mixture was photoirradiated under N_2 at 25 °C for 6 h. After completion, the reaction mixture was subjected to the **general work-up procedure** and purified using flash chromatography (gradient to 10% EtOAc in PE) on silica gel. The title compound was obtained in 87% yield (54.5 mg).

^{1}H NMR (400 MHz, $CDCl_3$) δ 8.01 – 7.91 (m, 4H), 7.17 – 7.07 (m, 4H), 6.73 (br s, 1H), 4.34 (q, *J* = 7.1 Hz, 4H), 1.37 (t, *J* = 7.1 Hz, 6H).
^{13}C NMR (101 MHz, $CDCl_3$) δ 166.4, 146.0, 131.4, 123.2, 116.8, 60.7, 14.4.
The spectroscopic data are consistent with the literature.[14]

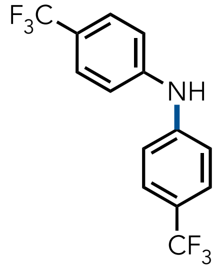

bis(4-(trifluoromethyl)phenyl)amine (11)

Compound **11** was prepared following **general procedure** using 1-bromo-4-(trifluoromethyl)benzene (45.0 mg, 0.2 mmol, 1.0 equiv) and 4-(trifluoromethyl)aniline (48.3 mg, 0.3 mmol, 1.5 equiv). The reaction mixture was photoirradiated under N_2 at 25 °C for 6 h. After completion, the reaction mixture was subjected to the **general work-up procedure** and purified using flash chromatography (gradient to 12% EtOAc in PE) on silica gel. The title compound was obtained in 96% yield (58.6 mg).

^{1}H NMR (400 MHz, $CDCl_3$) δ 7.56 (d, *J* = 8.4 Hz, 4H), 7.17 (d, *J* = 8.4 Hz, 4H), 6.09 (br s, 1H).
^{13}C NMR (101 MHz, $CDCl_3$) δ 145.0, 127.0 (q, *J* = 3.8 Hz), 124.5 (q, *J* = 271.0 Hz), 123.6 (q, *J* = 32.7 Hz), 117.5.
^{19}F NMR (377 MHz, $CDCl_3$) δ -62.25.
The spectroscopic data are consistent with the literature.[15]

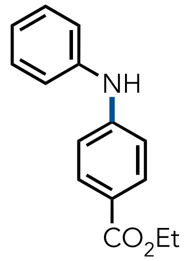

ethyl 4-(phenylamino)benzoate (12)

Compound **12** was prepared following **general procedure** using ethyl 4-bromobenzoate (45.8 mg, 0.2 mmol, 1.0 equiv) and aniline (27.9 mg, 0.3 mmol, 1.5 equiv). The reaction mixture was photoirradiated under N_2 at 25 °C for 6 h. After completion, the reaction mixture was subjected to the **general work-up procedure** and purified using flash chromatography (gradient to 10% EtOAc in PE) on silica gel. The title compound was obtained in 86% yield (41.5 mg).

^{1}H NMR (400 MHz, $CDCl_3$) δ 7.98 – 7.90 (m, 2H), 7.38 – 7.29 (m, 2H), 7.21 – 7.13 (m, 2H), 7.09 – 7.04 (m, 1H), 7.03 – 6.95 (m, 2H), 6.02 (br s, 1H), 4.34 (q, *J* = 7.1 Hz, 2H), 1.38 (t, *J* = 7.1 Hz, 3H).
^{13}C NMR (101 MHz, CDCl3) δ 166.6, 148.0, 141.0, 131.5, 129.6, 123.1, 121.6, 120.4, 114.7, 60.5, 14.5.
The spectroscopic data are consistent with the literature.[6]

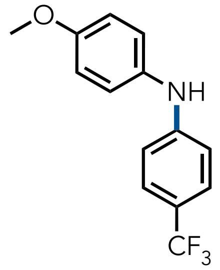

4-methoxy-*N*-(4-(trifluoromethyl)phenyl)aniline (13)

Compound **13** was prepared following **general procedure** using 1-bromo-4-(trifluoromethyl)benzene (45.0 mg, 0.2 mmol, 1.0 equiv) and 4-methoxyaniline (36.9 mg, 0.3 mmol, 1.5 equiv). The reaction mixture was photoirradiated under N_2 at 25 °C for 6 h. After completion, the reaction mixture was subjected to the **general work-up procedure** and purified using flash chromatography (gradient to 10% EtOAc in PE) on silica gel. The title compound was obtained in 96% yield (51.3 mg).

^1H NMR (400 MHz, $CDCl_3$) δ 7.50 – 7.40 (m, 2H), 7.20 – 7.08 (m, 2H), 6.96 – 6.90 (m, 2H), 6.89 – 6.84 (m, 2H), 5.76 (br s, 1H), 3.84 (s, 3H).
^{13}C NMR (101 MHz, $CDCl_3$) δ 156.5, 148.7, 133.8, 126.7 (q, J = 3.8 Hz), 124.3, 124.1 (q, J = 270.7 Hz), 120.4 (q, J = 32.6 Hz), 114.9, 113.8, 55.6.
^{19}F NMR (377 MHz, $CDCl_3$) δ -61.65.
The spectroscopic data are consistent with the literature.[16]

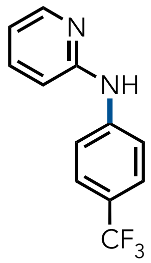

***N*-(4-(trifluoromethyl)phenyl)pyridin-2-amine (14)**

Compound **14** was prepared following **general procedure** using 1-bromo-4-(trifluoromethyl)benzene (45.0 mg, 0.2 mmol, 1.0 equiv) and pyridin-2-amine (28.2 mg, 0.3 mmol, 1.5 equiv). The reaction mixture was photoirradiated under N_2 at 25 °C for 18 h. After completion, the reaction mixture was subjected to the **general work-up procedure** and purified using flash chromatography (gradient to 20% EtOAc in PE) on silica gel. The title compound was obtained in 93% yield (44.3 mg).

^1H NMR (400 MHz, $CDCl_3$) δ 8.30 – 8.25 (m, 1H), 7.59 – 7.53 (m, 3H), 7.50 – 7.44 (m, 2H), 7.17 (br s, 1H), 6.94 – 6.89 (m, 1H), 6.87 – 6.81 (m, 1H).
^{13}C NMR (101 MHz, $CDCl_3$) δ 154.9, 148.3, 144.0, 138.0, 126.6 (q, J = 3.7 Hz), 125.0 (q, J = 271.2 Hz), 123.6 (q, J = 32.7 Hz), 118.3, 116.3, 109.9.
^{19}F NMR (377 MHz, $CDCl_3$) δ -62.23.
The spectroscopic data are consistent with the literature.[17]

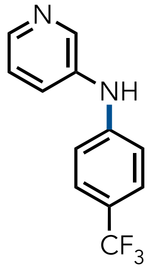

***N*-(4-(trifluoromethyl)phenyl)pyridin-3-amine (15)**

Compound **15** was prepared following **general procedure** using 1-bromo-4-(trifluoromethyl)benzene (45.0 mg, 0.2 mmol, 1.0 equiv) and pyridin-3-amine (28.2 mg, 0.3 mmol, 1.5 equiv). The reaction mixture was photoirradiated under N_2 at 25 °C for 18 h. After completion, the reaction mixture was subjected to the **general work-up procedure** and purified using flash chromatography (gradient to 55% EtOAc in PE) on silica gel. The title compound was obtained in 99% yield (47.2 mg).

^1H NMR (400 MHz, DMSO) δ 8.86 (s, 1H), 8.48 – 8.41 (m, 1H), 8.26 – 8.14 (m, 1H), 7.63 – 7.58 (m, 1H), 7.57 – 7.52 (m, 2H), 7.35 – 7.29 (m, 1H), 7.21 – 7.14 (m, 2H).
^{13}C NMR (101 MHz, DMSO) δ 146.7, 142.4, 141.0, 138.2, 126.6 (q, J = 3.9 Hz), 125.0, 124.8 (q, J = 270.5 Hz), 123.9, 119.3 (q, J = 32.0 Hz), 115.0.
^{19}F NMR (377 MHz, DMSO) δ -59.27.
The spectroscopic data are consistent with the literature.[18]

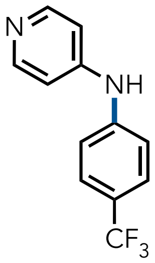

***N*-(4-(trifluoromethyl)phenyl)pyridin-4-amine (16)**

Compound **16** was prepared following **general procedure** using 1-bromo-4-(trifluoromethyl)benzene (45.0 mg, 0.2 mmol, 1.0 equiv) and pyridin-4-amine (28.2 mg, 0.3 mmol, 1.5 equiv). The reaction mixture was photoirradiated under N_2 at 25 °C for 18 h. After completion, the reaction mixture was subjected to the **general work-up procedure** and purified using flash chromatography (gradient to 100% EtOAc in PE) on silica gel. The title compound was obtained in 76% yield (36.2 mg).

^1H NMR (400 MHz, DMSO) δ 9.23 (s, 1H), 8.36 – 8.24 (m, 2H), 7.72 – 7.57 (m, 2H), 7.49 – 7.33 (m, 2H), 7.15 – 6.94 (m, 2H).
^{13}C NMR (101 MHz, DMSO) δ 150.3, 148.6, 144.6, 126.5 (q, J = 3.7 Hz), 124.5 (q, J = 271.0 Hz), 121.4 (q, J = 32.1 Hz), 118.1, 110.5.
^{19}F NMR (377 MHz, DMSO) δ -59.67.
The spectroscopic data are consistent with the literature.[19]

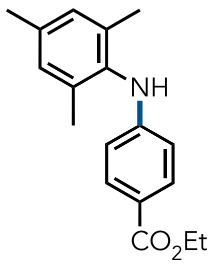

ethyl 4-(mesitylamino)benzoate (17)

Compound **17** was prepared following **general procedure** using ethyl 4-bromobenzoate (45.8 mg, 0.2 mmol, 1.0 equiv) and 2,4,6-trimethylaniline (40.6 mg, 0.3 mmol, 1.5 equiv). The reaction mixture was photoirradiated under N_2 at 60 °C for 12 h. After completion, the reaction mixture was subjected to the **general work-up procedure** and purified using flash chromatography (gradient to 10% EtOAc in PE) on silica gel. The title compound was obtained in 74% yield (41.9 mg).

^{1}H NMR (400 MHz, $CDCl_3$) δ 7.88 – 7.80 (m, 2H), 6.98 – 6.93 (m, 2H), 6.48 – 6.40 (m, 2H), 5.47 (s, 1H), 4.31 (q, *J* = 7.1 Hz, 2H), 2.32 (s, 3H), 2.16 (s, 6H), 1.35 (t, *J* = 7.1 Hz, 3H).
^{13}C NMR (101 MHz, $CDCl_3$) δ 166.9, 150.8, 136.6, 136.5, 134.1, 131.6, 129.4, 119.5, 112.0, 60.3, 21.0, 18.2, 14.5.
HRMS (EI) (m/z): calculated for $C_{18}H_{21}NO_2^+$ [M$^+$] 283.1567; found 283.1563.

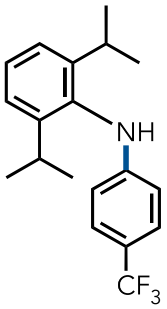

2,6-diisopropyl-*N*-(4-(trifluoromethyl)phenyl)aniline (18)

Compound **18** was prepared following **general procedure** using 1-bromo-4-(trifluoromethyl)benzene (45.0 mg, 0.2 mmol, 1.0 equiv) and 2,6-diisopropylaniline (53.2 mg, 0.3 mmol, 1.5 equiv). The reaction mixture was photoirradiated under N_2 at 60 °C for 18 h. After completion, the reaction mixture was subjected to the **general work-up procedure** and purified using flash chromatography (gradient to 0%–5% EtOAc in PE) on silica gel. The title compound was obtained in 77% yield (49.5 mg).

^{1}H NMR (400 MHz, $CDCl_3$) δ 7.40 – 7.31 (m, 3H), 7.25 – 7.22 (m, 2H), 6.53 – 6.44 (m, 2H), 5.37 (br s, 1H), 3.14 (sept, *J* = 6.9 Hz, 2H), 1.15 (d, *J* = 6.9 Hz, 12H).
^{13}C NMR (101 MHz, $CDCl_3$) δ 150.8, 147.8, 133.9, 128.1, 126.8 (q, *J* = 3.8 Hz), 125.0 (q, *J* = 270.4 Hz), 124.2, 119.4 (q, *J* = 32.8 Hz), 112.2, 28.44, 23.9.
^{19}F NMR (377 MHz, $CDCl_3$) δ -61.49.
The spectroscopic data are consistent with the literature.[20]

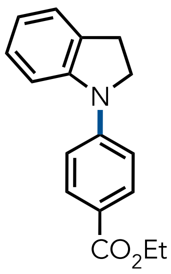

ethyl 4-(indolin-1-yl)benzoate (19)

Compound **19** was prepared following **general procedure** using ethyl 4-bromobenzoate (45.8 mg, 0.2 mmol, 1.0 equiv) and indoline (35.8 mg, 0.3 mmol, 1.5 equiv). The reaction mixture was photoirradiated under N_2 at 60 °C for 14 h. After completion, the reaction mixture was subjected to the **general work-up procedure** and purified using flash chromatography (gradient to 8% EtOAc in PE) on silica gel. The title compound was obtained in 95% yield (50.8 mg).

^{1}H NMR (400 MHz, $CDCl_3$) δ 8.12 – 8.05 (m, 2H), 7.38 – 7.31 (m, 1H), 7.31 – 7.23 (m, 3H), 7.22 – 7.16 (m, 1H), 6.94 – 6.86 (m, 1H), 4.42 (q, *J* = 7.1 Hz, 2H), 4.08 (t, *J* = 8.4 Hz, 2H), 3.22 (t, *J* = 8.3 Hz, 2H), 1.45 (t, *J* = 7.1 Hz, 3H).
^{13}C NMR (101 MHz, $CDCl_3$) δ 166.6, 147.8, 145.5, 132.0, 131.1, 127.2, 125.4, 121.5, 120.4, 115.4, 109.6, 60.6, 51.9, 28.1, 14.5.
HRMS (EI) (m/z): calculated for $C_{18}H_{21}NO_2^+$ [M$^+$] 267.1254; found 267.1247.

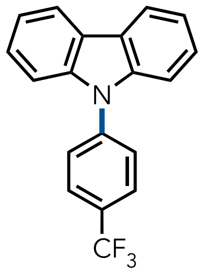

9-(4-(trifluoromethyl)phenyl)-9*H*-carbazole (20)

Compound **20** was prepared following **general procedure** using 1-bromo-4-(trifluoromethyl)benzene (45.0 mg, 0.2 mmol, 1.0 equiv) and 9*H*-carbazole (50.2 mg, 0.3 mmol, 1.5 equiv). The reaction mixture was photoirradiated under N_2 at 60 °C for 14 h. After completion, the reaction mixture was subjected to the **general work-up procedure** and purified using flash chromatography (gradient to 10% EtOAc in PE) on silica gel. The title compound was obtained in 65% yield (40.5 mg).

^{1}H NMR (400 MHz, $CDCl_3$) δ 8.20 – 8.13 (m, 2H), 7.93 – 7.85 (m, 2H), 7.78 – 7.69 (m, 2H), 7.50 – 7.40 (m, 4H), 7.38 – 7.30 (m, 2H).
^{13}C NMR (101 MHz, $CDCl_3$) δ 141.2, 140.4, 129.3 (q, *J* = 32.8 Hz), 127.2 (q, *J* = 3.77 Hz), 127.1, 126.3, 124.0 (q, J = 272.85 Hz), 123.9, 120.7, 120.6, 109.7.
^{19}F NMR (376 MHz, $CDCl_3$) δ -62.85.
The spectroscopic data are consistent with the literature.[21]

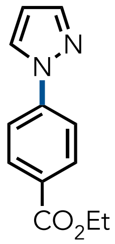

ethyl 4-(1*H*-pyrazol-1-yl)benzoate (21)

Compound **21** was prepared following **general procedure** using ethyl 4-bromobenzoate (45.8 mg, 0.2 mmol, 1.0 equiv) and 1*H*-pyrazole (20.4 mg, 0.3 mmol, 1.5 equiv). The reaction mixture was photoirradiated under N_2 at 60 °C for 14 h. After completion, the reaction mixture was subjected to the **general work-up procedure** and purified using flash chromatography (gradient to 12% EtOAc in PE) on silica gel. The title compound was obtained in 51% yield (22.1 mg).

^{1}H NMR (400 MHz, $CDCl_3$) δ 8.17 – 8.09 (m, 2H), 8.02 – 7.96 (m, 1H), 7.79 (m, 1H), 7.78 – 7.75 (m, 2H), 6.50 (m, 1H), 4.39 (q, *J* = 7.1 Hz, 2H), 1.41 (t, *J* = 7.1 Hz, 3H).
^{13}C NMR (101 MHz, $CDCl_3$) δ 166.0, 143.3, 142.0, 131.2, 128.3, 126.9, 118.4, 108.5, 61.2, 14.4.
The spectroscopic data are consistent with the literature.[22]

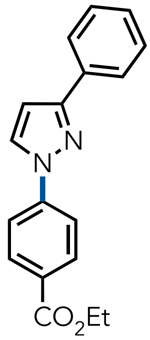

ethyl 4-(3-phenyl-1*H*-pyrazol-1-yl)benzoate (22)

Compound **22** was prepared following **general procedure** using ethyl 4-bromobenzoate (45.8 mg, 0.2 mmol, 1.0 equiv) and 3-phenyl-1*H*-pyrazole (43.3 mg, 0.3 mmol, 1.5 equiv). The reaction mixture was photoirradiated under N_2 at 60 °C for 16 h. After completion, the reaction mixture was subjected to the **general work-up procedure** and purified using flash chromatography (gradient to 10% EtOAc in PE) on silica gel. The title compound was obtained in 78% yield (45.6 mg).

^{1}H NMR (400 MHz, $CDCl_3$) δ 8.19 – 8.12 (m, 2H), 8.03 (d, *J* = 2.6 Hz, 1H), 7.96 – 7.90 (m, 2H), 7.88 – 7.83 (m, 2H), 7.48 – 7.42 (m, 2H), 7.39 – 7.34 (m, 1H), 6.82 (d, *J* = 2.6 Hz, 1H), 4.41 (q, *J* = 7.1 Hz, 2H), 1.42 (t, *J* = 7.1 Hz, 3H).
^{13}C NMR (101 MHz, $CDCl_3$) δ 166.0, 153.8, 143.3, 132.8, 131.2, 128.8, 128.5, 128.2, 128.0, 126.0, 118.1, 106.0, 61.2, 14.4.
The spectroscopic data are consistent with the literature.[6]

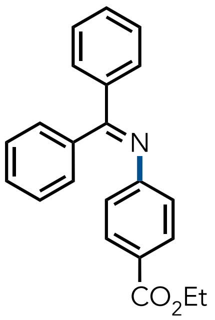

ethyl 4-((diphenylmethylene)amino)benzoate (23)

Compound **23** was prepared following **general procedure** using ethyl 4-bromobenzoate (45.8 mg, 0.2 mmol, 1.0 equiv) and diphenylmethanimine (54.4 mg, 0.3 mmol, 1.5 equiv). The reaction mixture was photoirradiated under N_2 at 60 °C for 3 h. After completion, the reaction mixture was subjected to the **general work-up procedure** and purified using flash chromatography (gradient to 70% EtOAc in PE) on silica gel. The title compound was obtained in 92% yield (60.6 mg).

^{1}H NMR (500 MHz, $CDCl_3$) δ 7.87 – 7.83 (m, 2H), 7.78 – 7.74 (m, 2H), 7.52 – 7.47 (m, 1H), 7.45 – 7.39 (m, 2H), 7.31 – 7.23 (m, 3H), 7.12 – 7.09 (m, 2H), 6.77 – 6.72 (m, 2H), 4.31 (q, *J* = 7.1 Hz, 2H), 1.35 (t, *J* = 7.1 Hz, 3H).
^{13}C NMR (126 MHz, $CDCl_3$) δ 168.9, 166.6, 155.8, 139.1, 135.8, 131.2, 130.4, 129.6, 129.4, 129.0, 128.4, 128.1, 125.1, 120.6, 60.8, 14.4.
The spectroscopic data are consistent with the literature.[6]

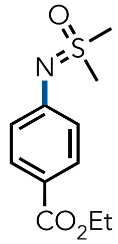

ethyl 4-((dimethyl(oxo)-λ^6-sulfaneylidene)amino)benzoate (24)

Compound **24** was prepared following **general procedure** using ethyl 4-bromobenzoate (45.8 mg, 0.2 mmol, 1.0 equiv) and iminodimethyl-λ^6-sulfanone (27.9 mg, 0.3 mmol, 1.5 equiv). The reaction mixture was photoirradiated under N_2 at 25 °C for 1 h. After completion, the reaction mixture was subjected to the **general work-up procedure** and purified using flash chromatography (gradient to 95% EtOAc in PE) on silica gel. The title compound was obtained in 99% yield (47.8 mg).
^{1}H NMR (400 MHz, $CDCl_3$) δ 7.93 – 7.85 (m, 2H), 7.06 (m, 2H), 4.31 (q, *J* = 7.1 Hz, 2H), 3.16 (s, 6H), 1.35 (t, *J* = 7.1 Hz, 3H).
^{13}C NMR (101 MHz, $CDCl_3$) δ 166.6, 150.3, 131.0, 123.7, 122.3, 60.6, 42.3, 14.4.
The spectroscopic data are consistent with the literature.[6]

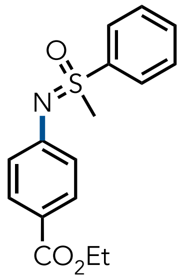

ethyl 4-((methyl(oxo)(phenyl)-λ^6-sulfaneylidene)amino)benzoate (25)

Compound **25** was prepared following **general procedure** using ethyl 4-bromobenzoate (45.8 mg, 0.2 mmol, 1.0 equiv) and imino(methyl)(phenyl)-λ^6-sulfanone (46. 6 mg, 0.3 mmol, 1.5 equiv). The reaction mixture was photoirradiated under N_2 at 25 °C for 3 h. After completion, the reaction mixture was subjected to the **general work-up procedure** and purified using flash chromatography (gradient to 20% EtOAc in PE) on silica gel. The title compound was obtained in 89% yield (54.0 mg).

^{1}H NMR (400 MHz, $CDCl_3$) δ 7.97 – 7.92 (m, 2H), 7.83 – 7.77 (m, 2H), 7.65 – 7.57 (m, 1H), 7.56 – 7.49 (m, 2H), 7.04 – 6.96 (m, 2H), 4.28 (q, *J* = 7.1 Hz, 2H), 3.28 (s, 3H), 1.32 (t, *J* = 7.1 Hz, 3H).
^{13}C NMR (101 MHz, $CDCl_3$) δ 166.7, 150.1, 138.8, 133.7, 130.9, 129.8, 128.6, 123.4, 122.5, 60.6, 46.4, 14.4.
The spectroscopic data are consistent with the literature.[6]

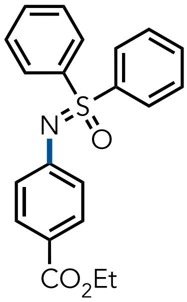

ethyl 4-((oxodiphenyl-λ^6-sulfaneylidene)amino)benzoate (26)

Compound **26** was prepared following **general procedure** using ethyl 4-bromobenzoate (45.8 mg, 0.2 mmol, 1.0 equiv) and iminodiphenyl-λ^6-sulfanone (65.2 mg, 0.3 mmol, 1.5 equiv). The reaction mixture was photoirradiated under N_2 at 25 °C for 3 h. After completion, the reaction mixture was subjected to the **general work-up procedure** and purified using flash chromatography (gradient to 25% EtOAc in PE) on silica gel. The title compound was obtained in 97% yield (70.9 mg).

^{1}H NMR (400 MHz, $CDCl_3$) δ 8.08 – 8.02 (m, 4H), 7.88 – 7.80 (m, 2H), 7.59 – 7.43 (m, 6H), 7.20 – 7.11 (m, 2H), 4.29 (q, *J* = 7.1 Hz, 2H), 1.33 (t, *J* = 7.1 Hz, 3H).
^{13}C NMR (101 MHz, $CDCl_3$) δ 166.8, 149.9, 140.5, 133.1, 130.9, 129.5, 128.5, 123.5, 123.1, 60.6, 14.5.
HRMS (ESI) (m/z): calculated for $C_{21}H_{20}NO_3S^+$ [(M+H)$^+$]: 366.1158; found 366.1165.

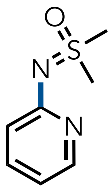

dimethyl(pyridin-2-ylimino)-λ^6-sulfanone (27)

Compound **27** was prepared following **general procedure** using 2-bromopyridine (31.6 mg, 0.2 mmol, 1.0 equiv) and iminodimethyl-λ^6-sulfanone (27.9 mg, 0.3 mmol, 1.5 equiv). The reaction mixture was photoirradiated under N_2 at 60 °C for 18 h. After completion, the reaction mixture was subjected to the **general work-up procedure** and purified using flash chromatography (gradient to 82% EtOAc in PE) on silica gel. The title compound was obtained in 87% yield (29.6 mg).
^{1}H NMR (400 MHz, $CDCl_3$) δ 8.22 – 8.16 (m, 1H), 7.55 – 7.47 (m, 1H), 6.82 – 6.74 (m, 2H), 3.37 (s, 6H).
^{13}C NMR (101 MHz, $CDCl_3$) δ 159.3, 147.4, 138.2, 116.8, 116.0, 42.9.
HRMS (ESI) (m/z): calculated for $C_7H_{11}N_2OS^+$ [(M+H)$^+$]: 171.0587; found 171.0587.

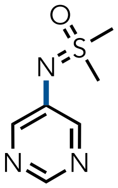

dimethyl(pyrimidin-5-ylimino)-λ^6-sulfanone (28)

Compound **28** was prepared following **general procedure** using 5-bromopyrimidine (31.8 mg, 0.2 mmol, 1.0 equiv) and iminodimethyl-λ^6-sulfanone (27.9 mg, 0.3 mmol, 1.5 equiv). The reaction mixture was photoirradiated under N_2 at 60 °C for 14 h. After completion, the reaction mixture was subjected to the **general work-up procedure** and purified using flash chromatography (gradient to 100% EtOAc in PE, then gradient to 15% MeOH in EtOAc) on silica gel. The title compound was obtained in 94% yield (32.2 mg).
^{1}H NMR (400 MHz, $CDCl_3$) δ 8.76 (s, 1H), 8.44 (s, 2H), 3.18 (s, 6H).
^{13}C NMR (101 MHz, $CDCl_3$) δ 152.0, 150.5, 140.7, 42.6.
The spectroscopic data are consistent with the literature.[6]

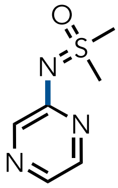

dimethyl(pyrazin-2-ylimino)-λ^6-sulfanone (29)

Compound **29** was prepared following **general procedure** using 2-bromopyrazine (31.8 mg, 0.2 mmol, 1.0 equiv) and iminodimethyl-λ^6-sulfanone (27.9 mg, 0.3 mmol, 1.5 equiv). The reaction mixture was photoirradiated under N_2 at 60 °C for 14 h. After completion, the reaction mixture was subjected to the **general work-up procedure** and purified using flash chromatography (gradient to 100% EtOAc in PE) on silica gel. The title compound was obtained in 82% yield (28.1 mg).
^{1}H NMR (400 MHz, $CDCl_3$) δ 8.13 (s, 1H), 8.06 (s, 1H), 8.00 (s, 1H), 3.36 (s, 6H).
^{13}C NMR (101 MHz, $CDCl_3$) δ 156.1, 141.3, 140.5, 135.8, 42.7.
HRMS (ESI) (m/z): calculated for $C_6H_{10}N_3OS^+$ [(M+H)$^+$]: 172.0539; found 172.0540.

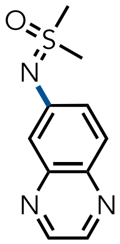

dimethyl(quinoxalin-6-ylimino)-λ^6-sulfanone (30)

Compound **30** was prepared following **general procedure** using 6-bromoquinoxaline (41.8 mg, 0.2 mmol, 1.0 equiv) and iminodimethyl-λ^6-sulfanone (27.9 mg, 0.3 mmol, 1.5 equiv). The reaction mixture was photoirradiated under N_2 at 60 °C for 14 h. After completion, the reaction mixture was subjected to the **general work-up procedure** and purified using flash chromatography (gradient to 80% EtOAc in PE) on silica gel. The title compound was obtained in 82% yield (36.3 mg).

^{1}H NMR (400 MHz, $CDCl_3$) δ 8.74 – 8.68 (m, 1H), 8.67 – 8.59 (m, 1H), 7.96 – 7.89 (m, 1H), 7.71 – 7.64 (m, 1H), 7.51 – 7.44 (m, 1H), 3.24 (s, 6H).

^{13}C NMR (101 MHz, $CDCl_3$) δ 147.7, 145.0, 144.3, 142.5, 139.7, 130.1, 129.3, 117.9, 42.3.

HRMS (ESI) (m/z): calculated for $C_{10}H_{12}N_3OS^+$ [(M+H)$^+$]: 222.0696; found 222.0698.

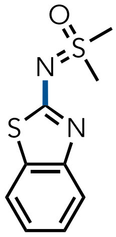

(benzo[*d*]thiazol-2-ylimino)dimethyl-λ^6-sulfanone (31)

Compound **31** was prepared following **general procedure** using 2-bromobenzo[*d*]thiazole (42.8 mg, 0.2 mmol, 1.0 equiv) and iminodimethyl-λ^6-sulfanone (27.9 mg, 0.3 mmol, 1.5 equiv). The reaction mixture was photoirradiated under N_2 at 60 °C for 14 h. After completion, the reaction mixture was subjected to the **general work-up procedure** and purified using flash chromatography (gradient to 60% EtOAc in PE) on silica gel. The title compound was obtained in 84% yield (38.0 mg).

^{1}H NMR (400 MHz, $CDCl_3$) δ 7.69 – 7.65 (m, 1H), 7.65 – 7.61 (m, 1H), 7.35 – 7.27 (m, 1H), 7.20 – 7.12 (m, 1H), 3.43 (s, 6H).

^{13}C NMR (101 MHz, $CDCl_3$) δ 167.8, 151.8, 133.1, 125.6, 122.8, 120.9, 120.4, 42.0.

HRMS (ESI) (m/z): calculated for $C_9H_{11}N_2OS_2^+$ [(M+H)$^+$]: 227.0307; found 227.0309.

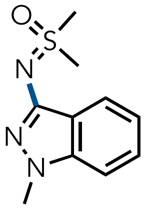

dimethyl((1-methyl-1*H*-indazol-3-yl)imino)-λ^6-sulfanone (32)

Compound **32** was prepared following **general procedure** using 3-bromo-1-methyl-1*H*-indazole (42.2 mg, 0.2 mmol, 1.0 equiv) and iminodimethyl-λ^6-sulfanone (27.9 mg, 0.3 mmol, 1.5 equiv). The reaction mixture was photoirradiated under N_2 at 60 °C for 18 h. After completion, the reaction mixture was subjected to **the general work-up procedure** and purified using flash chromatography (gradient to 90% EtOAc in PE) on silica gel. The title compound was obtained in 85% yield (38.0 mg).

^{1}H NMR (400 MHz, $CDCl_3$) δ 7.74 – 7.71 (m, 1H), 7.35 – 7.30 (m, 1H), 7.23 – 7.19 (m, 1H), 7.07 – 6.99 (m, 1H), 3.90 (s, 3H), 3.33 (s, 6H).

^{13}C NMR (101 MHz, $CDCl_3$) δ 145.9, 141.1, 126.7, 120.9, 119.0, 118.5, 108.3, 42.3, 35.1.

HRMS (ESI) (m/z): calculated for $C_{10}H_{14}N_3OS^+$ [(M+H)$^+$]: 224.0852; found 224.0855.

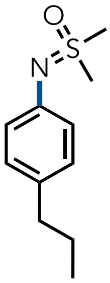

dimethyl((4-propylphenyl)imino)-λ^6-sulfanone (33)

Compound **33** was prepared following **general procedure** using 1-bromo-4-propylbenzene (39.8 mg, 0.2 mmol, 1.0 equiv) and iminodimethyl-λ^6-sulfanone (27.9 mg, 0.3 mmol, 1.5 equiv). The reaction mixture was photoirradiated under N_2 at 60 °C for 18 h. After completion, the reaction mixture was subjected to the **general work-up procedure** and purified using flash chromatography (gradient to 66% EtOAc in PE) on silica gel. The title compound was obtained in 92% yield (38.9 mg).

^{1}H NMR (400 MHz, $CDCl_3$) δ 7.06 – 7.01 (m, 2H), 7.00 – 6.95 (m, 2H), 3.12 (s, 6H), 2.55 – 2.47 (m, 2H), 1.60 (h, *J* = 7.4 Hz, 2H), 0.92 (t, *J* = 7.3 Hz, 3H).

^{13}C NMR (101 MHz, $CDCl_3$) δ 142.4, 136.7, 129.2, 123.3, 42.0, 37.4, 24.7, 13.9.

HRMS (ESI) (m/z): calculated for $C_{11}H_{18}NOS^+$ [(M+H)$^+$]: 212.1104; found 212.1105.

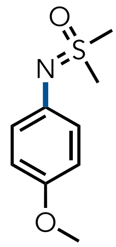

((4-methoxyphenyl)imino)dimethyl-λ^6-sulfanone (34)

Compound **34** was prepared following **general procedure** using 1-bromo-4-methoxybenzene (37.4 mg, 0.2 mmol, 1.0 equiv) and iminodimethyl-λ^6-sulfanone (27.9 mg, 0.3 mmol, 1.5 equiv). The reaction mixture was photoirradiated under N_2 at 60 °C for 18 h. After completion, the reaction mixture was subjected to the **general work-up procedure** and purified using flash chromatography (gradient to 96% EtOAc in PE) on silica gel. The title compound was obtained in 88% yield (35.1 mg).

^{1}H NMR (400 MHz, $CDCl_3$) δ 7.03 – 6.95 (m, 2H), 6.82 – 6.75 (m, 2H), 3.75 (s, 3H), 3.09 (s, 6H).

^{13}C NMR (101 MHz, $CDCl_3$) δ 155.4, 137.8, 124.8, 114.6, 55.5, 41.8.

The spectroscopic data are consistent with the literature.[23]

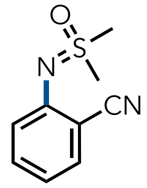

2-((dimethyl(oxo)-λ^6-sulfaneylidene)amino)benzonitrile (35)

Compound **35** was prepared following **general procedure** using 2-bromobenzonitrile (36.4 mg, 0.2 mmol, 1.0 equiv) and iminodimethyl-λ^6-sulfanone (27.9 mg, 0.3 mmol, 1.5 equiv). The reaction mixture was photoirradiated under N_2 at 25 °C for 3 h. After completion, the reaction mixture was subjected to the **general work-up procedure** and purified using flash chromatography (gradient to 60% EtOAc in PE) on silica gel. The title compound was obtained in 88% yield (34.2 mg).

^{1}H NMR (400 MHz, $CDCl_3$) δ 7.54 – 7.49 (m, 1H), 7.42 – 7.36 (m, 1H), 7.31 – 7.27 (m, 1H), 7.02 – 6.96 (m, 1H), 3.22 (s, 6H).

^{13}C NMR (101 MHz, $CDCl_3$) δ 148.9, 133.5, 133.5, 122.4, 121.9, 118.4, 108.0, 42.7.

HRMS (ESI) (m/z): calculated for $C_9H_{11}N_2OS^+$ [$(M+H)^+$]: 195.0587; found 195.0590.

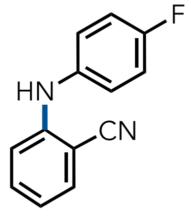

2-((4-fluorophenyl)amino)benzonitrile (36)

Compound **36** was prepared following **general procedure** using 2-bromobenzonitrile (36.4 mg, 0.2 mmol, 1.0 equiv) and 4-fluoroaniline (33.3 mg, 0.3 mmol, 1.5 equiv). The reaction mixture was photoirradiated under N_2 at 60 °C for 16 h. The title compound formed in 64% yield as determined with fluorobenzene as internal standard. Product formation was confirmed with GC-FID and GC-MS analysis.

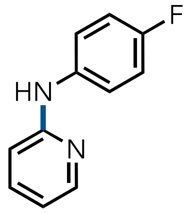

***N*-(4-fluorophenyl)pyridin-2-amine (37)**

Compound **37** was prepared following **general procedure** using 2-bromopyridine (31.6 mg, 0.2 mmol, 1.0 equiv) and 4-fluoroaniline (33.3 mg, 0.3 mmol, 1.5 equiv). The reaction mixture was photoirradiated under N_2 at 60 °C for 16 h. The title compound formed in 90% yield as determined with fluorobenzene as internal standard. Product formation was confirmed with GC-FID and GC-MS analysis.

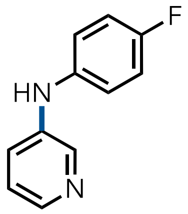

***N*-(4-fluorophenyl)pyridin-3-amine (38)**

Compound **38** was prepared following **general procedure** using 3-bromopyridine (31.6 mg, 0.2 mmol, 1.0 equiv) and 4-fluoroaniline (33.3 mg, 0.3 mmol, 1.5 equiv). The reaction mixture was photoirradiated under N_2 at 60 °C for 16 h. The title compound formed in 95% yield as determined with fluorobenzene as internal standard. Product formation was confirmed with GC-FID and GC-MS analysis.

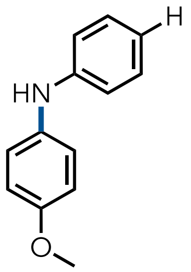

4-methoxy-*N*-phenylaniline (39)

Compound **39** was prepared following **general procedure** using 1-bromo-4-methoxybenzene (37.4 mg, 0.2 mmol, 1.0 equiv) and aniline (27.9 mg, 0.3 mmol, 1.5 equiv). The reaction mixture was photoirradiated under N_2 at 60 °C for 18 h. After completion, the reaction mixture was subjected to the **general work-up procedure** and purified using flash chromatography (gradient to 10% EtOAc in PE) on silica gel. The title compound was obtained in 22% yield (8.8 mg).

^{1}H NMR (400 MHz, $CDCl_3$) δ 7.26 – 7.17 (m, 2H), 7.08 (m, 2H), 6.94 – 6.79 (m, 5H), 5.50 (s, 1H), 3.81 (s, 3H).
The spectroscopic data are consistent with the literature.[24]

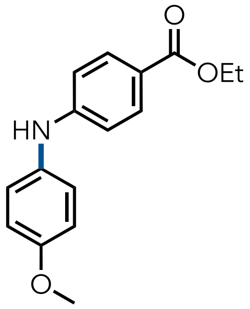

ethyl 4-((4-methoxyphenyl)amino)benzoate (40)

Compound **40** was prepared following **general procedure** using 1-bromo-4-methoxybenzene (37.4 mg, 0.2 mmol, 1.0 equiv) and ethyl 4-aminobenzoate (49.6 mg, 0.3 mmol, 1.5 equiv). The reaction mixture was photoirradiated under N_2 at 60 °C for 18 h. After completion, the reaction mixture was subjected to the **general work-up procedure** and purified using flash chromatography (gradient to 15% EtOAc in PE) on silica gel. The title compound was obtained in 35% yield (19.0 mg).

^{1}H NMR (400 MHz, $CDCl_3$) δ 7.92 – 7.84 (m, 2H), 7.18 – 7.09 (m, 2H), 6.94 – 6.86 (m, 2H), 6.85 – 6.77 (m, 2H), 5.86 (s, 1H), 4.32 (q, *J* = 7.1 Hz, 2H), 3.82 (s, 3H), 1.36 (t, *J* = 7.1 Hz, 3H).
^{13}C NMR (101 MHz, $CDCl_3$) δ 166.7, 156.6, 149.8, 133.6, 131.6, 124.4, 120.5, 114.8, 113.3, 60.4, 55.6, 14.5.
The spectroscopic data are consistent with the literature.[25]

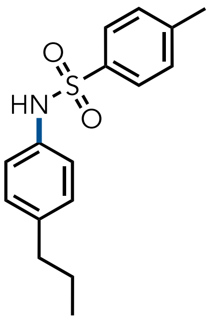

4-methyl-*N*-(4-propylphenyl)benzenesulfonamide (41)

Compound **41** was prepared following **general procedure** using 1-bromo-4-propylbenzene (39.8 mg, 0.2 mmol, 1.0 equiv) and 4-methylbenzenesulfonamide (51.4 mg, 0.3 mmol, 1.5 equiv). The reaction mixture was photoirradiated under N_2 at 60 °C for 14 h. After completion, the reaction mixture was subjected to the **general work-up procedure** and purified using flash chromatography (gradient to 25% EtOAc in PE) on silica gel. The title compound was obtained in yield: 52% yield (30.1 mg).

^{1}H NMR (400 MHz, $CDCl_3$) δ 7.70 – 7.63 (m, 2H), 7.24 – 7.18 (m, 2H), 7.15 (s, 1H), 7.05 – 6.95 (m, 4H), 2.53 – 2.45 (m, 2H), 2.36 (s, 3H), 1.56 (h, *J* = 7.4 Hz, 2H), 0.88 (t, *J* = 7.3 Hz, 3H).
^{13}C NMR (101 MHz, $CDCl_3$) δ 143.7, 140.0, 136.2, 134.1, 129.6, 129.3, 127.4, 122.1, 37.4, 24.4, 21.6, 13.7.
HRMS (EI) (m/z): calculated for $C_{16}H_{19}NO_2S^+$ [M^+] 289.1137; found 289.1133.

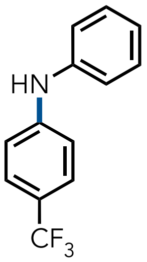

***N*-phenyl-4-(trifluoromethyl)aniline (42)**

Compound **42** was prepared following **general procedure** using 1-chloro-4-(trifluoromethyl)benzene (36.1 mg, 0.2 mmol, 1.0 equiv) and aniline (27.9 mg, 0.3 mmol, 1.5 equiv). The reaction mixture was photoirradiated under N_2 at 60 °C for 14 h. After completion, the reaction mixture was subjected to the **general work-up procedure** and purified using flash chromatography (gradient to 10% EtOAc in PE) on silica gel. The title compound was obtained in 23% yield (10.9 mg).

^1H NMR (400 MHz, $CDCl_3$) δ 7.51 – 7.43 (m, 2H), 7.38 – 7.29 (m, 2H), 7.20 – 7.11 (m, 2H), 7.10 – 7.00 (m, 3H), 5.91 (s, 1H).
^{13}C NMR (101 MHz, $CDCl_3$) δ 146.9, 141.2, 129.6, 126.8 (q, *J* = 3.8 Hz), 124.7 (q, *J* = 270.8 Hz), 123.0, 121.8 (q, *J* = 32.5 Hz), 120.1, 115.4.
^{19}F NMR (377 MHz, $CDCl_3$) δ -61.98.
The spectroscopic data are consistent with the literature.[12]

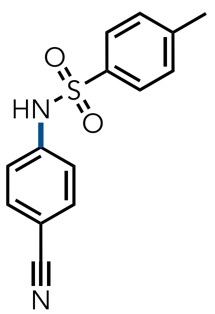

N-(4-cyanophenyl)-4-methylbenzenesulfonamide (43)

Compound **43** was prepared following **general procedure** using 4-chlorobenzonitrile (27.5 mg, 0.2 mmol, 1.0 equiv) and 4-methylbenzenesulfonamide (51.4 mg, 0.3 mmol, 1.5 equiv). The reaction mixture was photoirradiated under N_2 at 60 °C for 14 h. After completion, the reaction mixture was subjected to the **general work-up procedure** and purified using flash chromatography (gradient to 25% EtOAc in PE) on silica gel. The title compound was obtained in 69% yield (37.6 mg).

^1H NMR (400 MHz, $CDCl_3$) δ 7.78 – 7.70 (m, 2H), 7.56 – 7.48 (m, 2H), 7.46 (s, 1H), 7.30 – 7.26 (m, 2H), 7.21 – 7.13 (m, 2H), 2.40 (s, 3H).
^{13}C NMR (101 MHz, $CDCl_3$) δ 144.9, 141.1, 135.6, 133.7, 130.1, 127.3, 119.4, 118.5, 107.8, 21.7.
The spectroscopic data are consistent with the literature.[26]

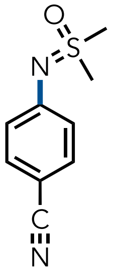

4-((dimethyl(oxo)-λ^6-sulfaneylidene)amino)benzonitrile (44)

Compound **44** was prepared following **general procedure** using 4-chlorobenzonitrile (27.5 mg, 0.2 mmol, 1.0 equiv) and iminodimethyl-λ^6-sulfanone (27.9 mg, 0.3 mmol, 1.5 equiv). The reaction mixture was photoirradiated under N_2 at 60 °C for 3 h. After completion, the reaction mixture was subjected to the **general work-up procedure** and purified using flash chromatography (gradient to 25% EtOAc in PE) on silica gel. The title compound was obtained in 98% yield (38.1 mg).

^1H NMR (400 MHz, $CDCl_3$) δ 7.52 – 7.43 (m, 2H), 7.13 – 7.04 (m, 2H), 3.19 (s, 6H).
^{13}C NMR (101 MHz, $CDCl_3$) δ 150.4, 133.4, 123.0, 119.7, 104.3, 42.6.
The spectroscopic data are consistent with the literature.[27]

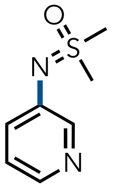

dimethyl(pyridin-3-ylimino)-λ^6-sulfanone (45)

Compound **45** was prepared following **general procedure** using 3-chloropyridine (22.7 mg, 0.2 mmol, 1.0 equiv) and iminodimethyl-λ^6-sulfanone (27.9 mg, 0.3 mmol, 1.5 equiv). The reaction mixture was photoirradiated under N_2 at 60 °C for 14 h. After completion, the reaction mixture was subjected to the **general work-up procedure** and purified using flash chromatography (gradient to 100% EtOAc in PE, then gradient to 15% MeOH in EtOAc) on silica gel. The title compound was obtained in 60% yield (20.4 mg).
^1H NMR (400 MHz, $CDCl_3$) δ 8.34 (m, 1H), 8.21 (m, 1H), 7.43 – 7.36 (m, 1H), 7.16 – 7.10 (m, 1H), 3.16 (s, 6H).
^{13}C NMR (101 MHz, $CDCl_3$) δ 145.2, 143.2, 141.9, 129.9, 123.8, 42.4.
The spectroscopic data are consistent with the literature.[6]

ethyl 4-(((3a*R*,5*R*,5a*S*,8a*S*,8b*R*)-2,2,7,7-tetramethyltetrahydro-5*H*-bis([1,3]dioxolo)[4,5-*b*:4',5'-*d*]pyran-5-yl)methoxy)benzoate (46)

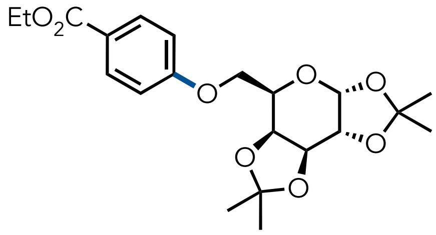

Compound **46** was prepared following **general procedure** using ethyl 4-bromobenzoate (45.8 mg, 0.2 mmol, 1.0 equiv) and ((3a*R*,5*R*,5a*S*,8a*S*,8b*R*)-2,2,7,7-tetramethyltetrahydro-5*H*-bis([1,3]dioxolo)[4,5-*b*:4',5'-*d*]pyran-5-yl)methanol (78.0 mg, 0.3 mmol, 1.5 equiv). The reaction mixture was photoirradiated under N_2 at 60 °C for 14 h. After completion, the reaction mixture was subjected to the **general work-up procedure** and purified using flash chromatography (gradient to 15% EtOAc in PE) on silica gel. The title compound was obtained in 78% yield (63.7 mg).

^{1}H NMR (400 MHz, $CDCl_3$) δ 8.02 – 7.92 (m, 2H), 6.99 – 6.91 (m, 2H), 5.57 (d, *J* = 5.0 Hz, 1H), 4.66 (dd, *J* = 7.9, 2.5 Hz, 1H), 4.39 – 4.30 (m, 4H), 4.25 – 4.14 (m, 3H), 1.52 (s, 3H), 1.47 (s, 3H), 1.38 (t, *J* = 7.1 Hz, 3H), 1.35 (s, 3H), 1.34 (s, 3H).

^{13}C NMR (101 MHz, $CDCl_3$) δ 166.5, 162.4, 131.6, 123.2, 114.4, 109.6, 108.9, 96.4, 71.0, 70.7, 70.7, 66.9, 66.3, 60.7, 26.1, 26.1, 25.0, 24.5, 14.5.

The spectroscopic data are consistent with the literature.[6]

ethyl (*S*)-4-(4-(2-((*tert*-butoxycarbonyl)amino)-3-methoxy-3-oxopropyl)phenoxy)benzoate (47)

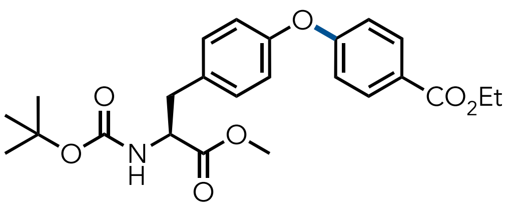

Compound **47** was prepared following **general procedure** using ethyl 4-bromobenzoate (45.8 mg, 0.2 mmol, 1.0 equiv) and methyl (*tert*-butoxycarbonyl)-*L*-tyrosinate (88.6 mg, 0.3 mmol, 1.5 equiv). The reaction mixture was photoirradiated under N_2 at 60 °C for 14 h. After completion, the reaction mixture was subjected to the **general work-up procedure** and purified using flash chromatography (gradient to 20% EtOAc in PE) on silica gel. The title compound was obtained in 82% yield (72.7 mg).

^{1}H NMR (400 MHz, $CDCl_3$) δ 8.03 – 7.96 (m, 2H), 7.18 – 7.10 (m, 2H), 7.02 – 6.92 (m, 4H), 5.09 – 5.00 (m, 1H), 4.64 – 4.54 (m, 1H), 4.35 (q, *J* = 7.1 Hz, 2H), 3.72 (s, 3H), 3.18 – 2.97 (m, 2H), 1.42 (s, 9H), 1.37 (t, *J* = 7.1 Hz, 3H).

^{13}C NMR (101 MHz, $CDCl_3$) δ 172.3, 166.2, 161.7, 155.1, 154.8, 132.3, 131.7, 130.9, 125.0, 120.1, 117.4, 80.1, 60.9, 54.5, 52.4, 37.9, 28.4, 14.4.

The spectroscopic data are consistent with the literature.[6]

ethyl 4-((4-(*N*-((2*R*,3*S*)-3-(((((3*R*,3a*S*,6a*R*)-hexahydrofuro[2,3-*b*]furan-3-yl)oxy)carbonyl)amino)-2-hydroxy-4-phenylbutyl)-*N*-isobutylsulfamoyl)phenyl)amino)benzoate (48)

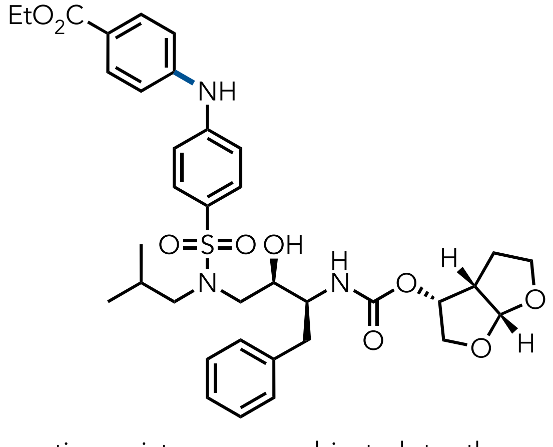

Compound **48** was prepared following **general procedure** using ethyl 4-bromobenzoate (45.8 mg, 0.2 mmol, 1.0 equiv) and (3*R*,3a*S*,6a*R*)-hexahydrofuro[2,3-*b*]furan-3-yl((2*S*,3*R*)-4-((4-amino-*N*-isobutylphenyl)sulfonamido)-3-hydroxy-1-phenylbutan-2-yl)carbamate (164.3 mg, 0.3 mmol, 1.5 equiv). The reaction mixture was photoirradiated under N_2 at 60 °C for 14 h. After completion, the reaction mixture was subjected to the **general work-up procedure** and purified using flash chromatography (gradient to 50%–70% EtOAc in PE) on silica gel. The title compound was obtained in 61% yield (84.9 mg).

^{1}H NMR (400 MHz, Acetone) δ 8.49 (br s, 1H), 7.97 – 7.92 (m, 2H), 7.77 – 7.72 (m, 2H), 7.37 – 7.32 (m, 2H), 7.31 – 7.25 (m, 4H), 7.23 (m, 2H), 7.18 – 7.13 (m, 1H), 6.42 (d, *J* = 9.1 Hz, 1H), 5.51 (d, *J* = 5.2 Hz,

1H), 4.91 (dt, J = 8.2, 5.9 Hz, 1H), 4.30 (q, J = 7.1 Hz, 2H), 4.26 (br s, 1H), 3.93 – 3.80 (m, 3H), 3.75 – 3.68 (m, 1H), 3.64 – 3.54 (m, 2H), 3.45 (dd, J = 15.0, 3.0 Hz, 1H), 3.21 (dd, J = 13.9, 3.4 Hz, 1H), 3.11 – 2.98 (m, 2H), 2.96 – 2.90 (m, 1H), 2.89 (m, 2 H), 2.70 (dd, J = 13.9, 10.6 Hz, 1H), 1.55 – 1.39 (m, 2H), 1.33 (t, J = 7.1 Hz, 3H), 0.90 (m, 6H).
^{13}C NMR (101 MHz, Acetone) δ 166.3, 156.3, 147.2, 147.1, 140.1, 131.9, 131.2, 130.3, 130.1, 128.9, 126.8, 123.9, 117.7, 117.5, 110.1, 74.1, 73.8, 71.4, 69.8, 61.0, 58.7, 56.9, 54.0, 46.2, 36.4, 27.7, 26.6, 20.5, 20.4, 14.6.
HRMS (ESI) (m/z): calculated for $C_{36}H_{46}N_3O_9S^+$ [(M+H)$^+$]: 696.2949; found 696.2957.

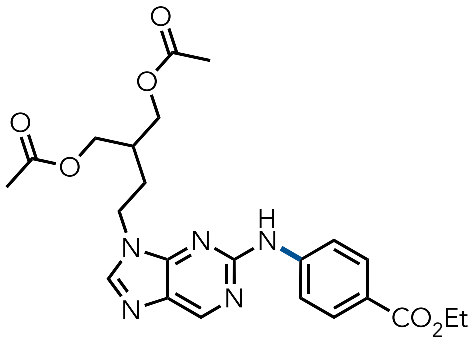

2-(2-(2-((4-(ethoxycarbonyl)phenyl)amino)-9*H*-purin-9-yl)ethyl)propane-1,3-diyl diacetate (49)

Compound **49** was prepared following **general procedure** using ethyl 4-bromobenzoate (45.8 mg, 0.2 mmol, 1.0 equiv) and 2-(2-(2-amino-9*H*-purin-9-yl)ethyl)propane-1,3-diyl diacetate (164.3 mg, 0.3 mmol, 1.5 equiv). The reaction mixture was photoirradiated under N_2 at 60 °C for 14 h. After completion, the reaction mixture was subjected to the **general work-up procedure** and purified using flash chromatography (gradient to 100% EtOAc in PE) on silica gel. The title compound was obtained in 42% yield (39.4 mg).
^{1}H NMR (400 MHz, $CDCl_3$) δ 8.81 (s, 1H), 8.24 (s, 1H), 8.07 – 7.98 (m, 2H), 7.91 (s, 1H), 7.84 – 7.76 (m, 2H), 4.36 (q, J = 7.1 Hz, 2H), 4.33 (t, J = 6.9 Hz, 2H), 4.16 – 4.12 (m, 4H), 2.02 (m, 9H), 1.38 (t, J = 7.1 Hz, 3H).
^{13}C NMR (101 MHz, $CDCl_3$) δ 170.9, 166.5, 155.8, 152.6, 149.5, 144.1, 143.3, 130.9, 128.9, 123.7, 117.4, 63.7, 60.7, 41.4, 35.2, 28.8, 20.9, 14.5.
HRMS (ESI) (m/z): calculated for $C_{23}H_{28}N_5O_6^+$ [(M+H)$^+$]: 470.2034; found 470.2042.

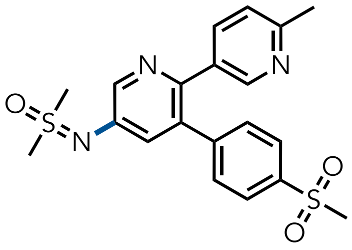

dimethyl((6'-methyl-3-(4-(methylsulfonyl)phenyl)-[2,3'-bipyridin]-5-yl)imino)-λ^6-sulfanone (50)

Compound **50** was prepared following **general procedure** using 5-chloro-6'-methyl-3-(4-(methylsulfonyl)phenyl)-2,3'-bipyridine (71.8 mg, 0.2 mmol, 1.0 equiv) and iminodimethyl-λ^6-sulfanone (27.9 mg, 0.3 mmol, 1.5 equiv). The reaction mixture was photoirradiated under N_2 at 60 °C for 14 h. After completion, the reaction mixture was subjected to the **general work-up procedure** and purified using flash chromatography (gradient to 15%–20% MeOH in EtOAc) on silica gel. The title compound was obtained in 82% yield (68.1 mg).
^{1}H NMR (600 MHz, $CDCl_3$) δ 8.51 – 8.48 (m, 1H), 8.40 – 8.36 (m, 1H), 7.90 – 7.86 (m, 2H), 7.70 – 7.66 (m, 1H), 7.45 – 7.42 (m, 1H), 7.41 – 7.37 (m, 2H), 7.16 – 7.11 (m, 1H), 3.25 (s, 6H), 3.09 (s, 3H), 2.58 (s, 3H).
^{13}C NMR (151 MHz, $CDCl_3$) δ 156.5, 148.4, 146.2, 145.1, 144.9, 141.8, 139.9, 138.8, 134.8, 133.1, 131.4, 130.5, 127.9, 123.5, 44.7, 42.8, 23.4.
HRMS (ESI) (m/z): calculated for $C_{20}H_{22}N_3O_3S_2^+$ [(M+H)$^+$]: 416.1097; found 416.1099.

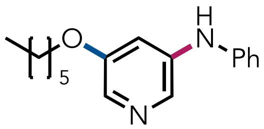

5-(hexyloxy)-*N*-phenylpyridin-3-amine (51)

Compound **51** was prepared following **general difunctionalization procedure** using 3,5-dibromopyridine (47.4 mg, 0.2 mmol, 1.0 equiv) and hexan-1-ol (27.1 uL, 0.22 mmol, 1.08 equiv) in the first step. The reaction mixture was photoirradiated under N_2 at 60 °C for 3 h. In the second step, aniline (27.9 mg, 0.3 mmol, 1.5 equiv) and *tert*-butylamine (1.3 equiv.) were added before irradiation under N_2 at 60 °C for 18 h.

After completion, the reaction mixture was subjected to the **general work-up procedure** and purified using flash chromatography (gradient to 40% EtOAc in PE) on silica gel. The title compound was obtained in 63% yield (34.0 mg).

^{1}H NMR (400 MHz, $CDCl_3$) δ 8.03 – 7.97 (m, 1H), 7.88 – 7.81 (m, 1H), 7.34 – 7.27 (m, 2H), 7.14 – 7.08 (m, 2H), 7.04 – 6.94 (m, 2H), 6.02 (br s, 1H), 3.95 (t, J = 6.5 Hz, 2H), 1.83 – 1.71 (m, 2H), 1.49 – 1.39 (m, 2H), 1.37 – 1.28 (m, 4H), 0.93 – 0.87 (m, 3H).

^{13}C NMR (101 MHz, $CDCl_3$) δ 156.1, 141.7, 141.1, 131.9, 129.6, 128.8, 122.4, 119.0, 109.3, 68.6, 31.6, 29.2, 25.7, 22.7, 14.1.

HRMS (EI) (m/z): calculated for $C_{17}H_{22}N_2O^+$ [M^+] 270.1727; found 270.1720.

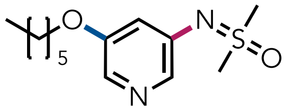

((5-(hexyloxy)pyridin-3-yl)imino)dimethyl-λ^6-sulfanone (52)

Compound **52** was prepared following **general difunctionalization procedure** using 3,5-dibromopyridine (47.4 mg, 0.2 mmol, 1.0 equiv) and hexan-1-ol (27.1 uL, 0.22 mmol, 1.08 equiv) in the first step. The reaction mixture was photoirradiated under N_2 at 60 °C for 3 h. In the second step, iminodimethyl-λ^6-sulfanone (27.9 mg, 0.3 mmol, 1.5 equiv) and *tert*-butylamine (1.3 equiv.) were added before irradiation under N_2 at 60 °C for 18 h.

After completion, the reaction mixture was subjected to the **general work-up procedure** and purified using flash chromatography (gradient to 100% EtOAc in PE) on silica gel. The title compound was obtained in 57% yield (30.8 mg).

^{1}H NMR (400 MHz, $CDCl_3$) δ 8.01 – 7.90 (m, 2H), 6.96 – 6.89 (m, 1H), 3.94 (t, J = 6.5 Hz, 2H), 3.15 (s, 6H), 1.80 – 1.69 (m, 2H), 1.48 – 1.38 (m, 2H), 1.34 – 1.28 (m, 4H), 0.93 – 0.83 (m, 3H).

^{13}C NMR (101 MHz, $CDCl_3$) δ 155.8, 142.51, 137.1, 131.6, 115.6, 68.4, 42.3, 31.6, 29.2, 25.7, 22.6, 14.1.

HRMS (EI) (m/z): calculated for $C_{13}H_{22}N_2O_2S^+$ [M^+] 270.1397; found 270.1393.

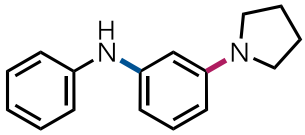

***N*-phenyl-3-(pyrrolidin-1-yl)aniline (53)**

Compound **53** was prepared following **general difunctionalization procedure** using 1,3-dibromobenzene (47.2 mg, 0.2 mmol, 1.0 equiv) and aniline (19.1 uL, 0.21 mmol, 1.05 equiv) in the first step. The reaction mixture was photoirradiated under N_2 at 25 °C for 2 h. In the second step, pyrrolidine (42.7 mg, 0.6 mmol, 3.0 equiv) (**note:** no *tert*-butylamine required in second step for pyrrolidine) was added before irradiation under N_2 at 25 °C for 14 h.

After completion, the reaction mixture was subjected to the **general work-up procedure** and purified using flash chromatography (gradient to 10% EtOAc in PE) on silica gel. The title compound was obtained in 54% yield (25.7 mg).

^{1}H NMR (400 MHz, $CDCl_3$) δ 7.31 – 7.20 (m, 2H), 7.17 – 7.05 (m, 3H), 6.94 – 6.85 (m, 1H), 6.46 – 6.39 (m, 1H), 6.32 (s, 1H), 6.25 – 6.18 (m, 1H), 5.67 (s, 1H), 3.34 – 3.21 (m, 4H), 2.06 – 1.93 (m, 4H).

^{13}C NMR (101 MHz, $CDCl_3$) δ 149.0, 144.0, 143.8, 130.0, 129.3, 120.5, 117.7, 106.0, 105.5, 101.6, 47.8, 25.5.

HRMS (EI) (m/z): calculated for $C_{16}H_{18}N_2^+$ [M^+] 238.1465; found 238.1463.

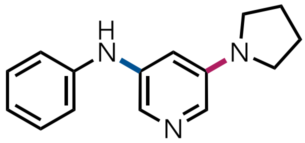

***N*-phenyl-5-(pyrrolidin-1-yl)pyridin-3-amine (54)**

Compound **54** was prepared following **general difunctionalization procedure** using 3,5-dibromopyridine (47.4 mg, 0.2 mmol, 1.0 equiv) and aniline (19.1 uL, 0.21 mmol, 1.05 equiv) in the first step. The reaction mixture was photoirradiated under N_2 at 25 °C for 3 h. In the second step, pyrrolidine (42.7 mg, 0.6 mmol, 3.0 equiv) (**note:** no *tert*-butylamine required in second step for pyrrolidine) was added before irradiation under N_2 at 60 °C for 16 h. After completion, the reaction mixture was subjected to the **general work-up procedure** and purified using flash chromatography

(gradient to 70%–80% EtOAc in PE) on silica gel. The title compound was obtained in 60% yield (28.7 mg).
^{1}H NMR (400 MHz, $CDCl_3$) δ 7.74 (d, *J* = 2.3 Hz, 1H), 7.60 (d, *J* = 2.5 Hz, 1H), 7.31 – 7.23 (m, 2H), 7.15 – 7.06 (m, 2H), 6.94 (t, *J* = 7.4 Hz, 1H), 6.58 (s, 1H), 5.81 (s, 1H), 3.33 – 3.20 (m, 4H), 2.07 – 1.95 (m, 4H).
^{13}C NMR (101 MHz, $CDCl_3$) δ 144.4, 142.8, 139.9, 129.5, 128.1, 127.5, 121.4, 118.0, 106.4, 47.5, 25.4.
HRMS (EI) (m/z): calculated for $C_{15}H_{17}N_3^+$ [M$^+$] 239.1417; found 239.1411.

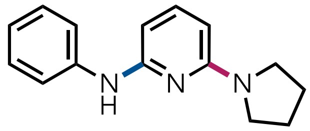

_N_-phenyl-6-(pyrrolidin-1-yl)pyridin-2-amine (55)
Compound **55** was prepared following **general difunctionalization procedure** using 2,6-dibromopyridine (47.4 mg, 0.2 mmol, 1.0 equiv) and aniline (19.1 uL, 0.21 mmol, 1.05 equiv) in the first step. The reaction mixture was photoirradiated under N_2 at 25 °C for 3 h. In the second step, pyrrolidine (42.7 mg, 0.6 mmol, 3.0 equiv) (**note:** no *tert*-butylamine required in second step for pyrrolidine) was added before irradiation under N_2 at 60 °C for 16 h. After completion, the reaction mixture was subjected to the **general work-up procedure** and purified using flash chromatography (gradient to 20% EtOAc in PE) on silica gel. The title compound was obtained in 33% yield (15.8 mg).
^{1}H NMR (400 MHz, $CDCl_3$) δ 7.43 – 7.35 (m, 2H), 7.31 – 7.21 (m, 3H), 6.99 – 6.89 (m, 1H), 6.27 (s, 1H), 6.06 (d, *J* = 7.8 Hz, 1H), 5.81 (d, *J* = 8.1 Hz, 1H), 3.49 – 3.39 (m, 4H), 2.03 – 1.90 (m, 4H).
^{13}C NMR (101 MHz, $CDCl_3$) δ 156.9, 154.7, 141.5, 138.6, 129.0, 121.5, 119.5, 97.2, 95.2, 46.7, 25.6.
HRMS (EI) (m/z): calculated for $C_{15}H_{17}N_3^+$ [M$^+$] 239.1417; found 239.1415.

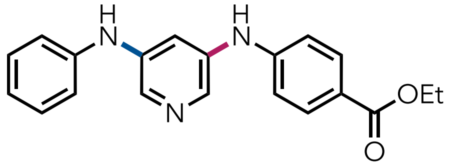

ethyl 4-((5-(phenylamino)pyridin-3-yl)amino)benzoate (56)
Compound **56** was prepared following **general difunctionalization procedure** using 3,5-dibromopyridine (47.4 mg, 0.2 mmol, 1.0 equiv) and aniline (19.1 uL, 0.21 mmol, 1.05 equiv) in the first step. The reaction mixture was photoirradiated under N_2 at 25 °C for 3 h. In the second step, ethyl 4-aminobenzoate (49.6 mg, 0.3 mmol, 1.5 equiv) and *tert*-butylamine (1.3 equiv.) were added before irradiation under N_2 at 60 °C for 16 h. After completion, the reaction mixture was subjected to the **general work-up procedure** and purified using flash chromatography (gradient to 50% EtOAc in PE) on silica gel. The title compound was obtained in 57% yield (38.0 mg).
^{1}H NMR (400 MHz, $CDCl_3$) δ 7.98 (d, *J* = 11.6 Hz, 2H), 7.90 (d, *J* = 7.3 Hz, 2H), 7.31 – 7.21 (m, 3H), 7.11 – 7.03 (m, 2H), 7.02 – 6.95 (m, 3H), 6.80 (s, 1H), 6.32 (s, 1H), 4.32 (q, *J* = 7.1 Hz, 2H), 1.35 (t, *J* = 7.1 Hz, 3H).
^{13}C NMR (101 MHz, $CDCl_3$) δ 166.5, 146.9, 141.4, 141.1, 138.8, 132.5, 131.5, 129.6, 129.6, 122.5, 119.1, 118.9, 115.5, 112.7, 60.7, 14.4.
HRMS (ESI) (m/z): calculated for $C_{20}H_{20}N_3O_2^+$ [(M+H)$^+$]: 334.1551; found 334.1553

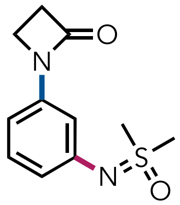

1-(3-((dimethyl(oxo)-λ^6-sulfaneylidene)amino)phenyl)azetidin-2-one (57)
Compound **57** was prepared following **general difunctionalization procedure** using 1,3-dibromobenzene (47.2 mg, 0.2 mmol, 1.0 equiv) and azetidin-2-one (15.6 mg, 0.22 mmol, 1.1 equiv) in the first step. The reaction mixture was photoirradiated under N_2 at 60 °C for 16 h. In the second step, iminodimethyl-λ^6-sulfanone (27.9 mg, 0.3 mmol, 1.5 equiv) and *tert*-butylamine (1.3 equiv.) were added before irradiation under N_2 at 60 °C for 16 h. After completion, the reaction mixture was subjected to the **general work-up procedure** and purified using flash chromatography (gradient to 100% EtOAc in PE) on silica gel. The title compound was obtained in 60% yield (28.6 mg).
^{1}H NMR (400 MHz, $CDCl_3$) δ 7.20 – 7.14 (m, 1H), 7.11 – 7.07 (m, 1H), 6.96 – 6.92 (m, 1H), 6.85 – 6.80 (m, 1H), 3.58 (t, *J* = 4.5 Hz, 2H), 3.14 (s, 6H), 3.06 (t, *J* = 4.5 Hz, 2H).

^{13}C NMR (101 MHz, $CDCl_3$) δ 164.5, 146.0, 139.4, 129.9, 118.7, 111.1, 110.7, 42.2, 38.1, 36.0.
HRMS (ESI) (m/z): calculated for $C_{11}H_{15}N_2O_2S^+$ [$(M+H)^+$]: 239.0849; found 239.0851.

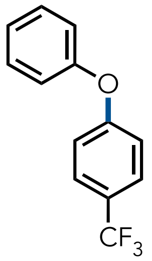

1-phenoxy-4-(trifluoromethyl)benzene (2)
A 20 mL crimp top vial was charged with a magnetic stirring bar, 1-bromo-4-(trifluoromethyl)benzene (1.8 g, 8.0 mmol, 1.0 equiv), phenol (1.13 g, 12.0 mmol, 1.5 equiv) and 16.0 mL of a catalyst stock solution containing 4CzIPN (32.0 mg, 0.04 mmol, 0.005 equiv) and $NiBr_2$•glyme (128.0 mg, 0.4 mmol, 0.05 equiv) dissolved in DMA. The reaction mixture was then degassed and refilled with nitrogen two times via a syringe needle before *tert*-butylamine (1.09 mL, 10.4 mmol, 1.3 equiv) was added via syringe. After degassing one more time and refilling with nitrogen, the reaction mixture was photoirradiated for 18 h at 60 °C through the plane bottom side of the snap vial using a single higher power (see above for specifications) blue LED (455 (± 15) nm). After completion, the reaction mixture was subjected to the **general work-up procedure** and purified using flash chromatography (100% petroleum ether) on silica gel. The title compound was obtained in 79% yield (1.5 g).
Note: *Tert*-butylamine is volatile and therefore, it is recommended to not evacuate the reaction mixture to high vacuum for an extended time period.
^1H NMR (300 MHz, $CDCl_3$) δ 7.62 – 7.54 (m, 2H), 7.45 – 7.36 (m, 2H), 7.24 – 7.17 (m, 1H), 7.10 – 7.02 (m, 4H).
The spectroscopic data are consistent with the literature and small-scale reaction of compound **2**.[7]

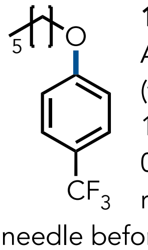

1-(hexan-2-yloxy)-4-(trifluoromethyl)benzene (5)
A 20 mL crimp top vial was charged with a magnetic stirring bar, 1-bromo-4-(trifluoromethyl)benzene (1.35 g, 6.0 mmol, 1.0 equiv), n-hexanol (918 mg, 9.0 mmol, 1.5 equiv) and 12.0 mL of a catalyst stock solution containing 4CzIPN (24.0 mg, 0.03 mmol, 0.005 equiv) and $NiBr_2$•glyme (96.0 mg, 0.3 mmol, 0.05 equiv) dissolved in DMA. The reaction mixture was then degassed and refilled with nitrogen two times via a syringe needle before *tert*-butylamine (815.0 uL, 7.8 mmol, 1.3 equiv) was added via syringe. After degassing one more time and refilling with nitrogen, the reaction mixture was photoirradiated for 18 h at 60 °C through the plane bottom side of the snap vial using a single higher power (see above for specifications) blue LED (455 (± 15) nm). After completion, the reaction mixture was subjected to the **general work-up procedure** and purified using flash chromatography (100% petroleum ether) on silica gel. The title compound was obtained in 78% yield (1.15 g).
Note: *Tert*-butylamine is volatile and therefore, it is recommended to not evacuate the reaction mixture to high vacuum for an extended time period.
^1H NMR (400 MHz, $CDCl_3$) δ 7.57 – 7.50 (m, 2H), 6.99 – 6.92 (m, 2H), 3.99 (t, *J* = 6.5 Hz, 2H), 1.87 – 1.76 (m, 2H), 1.55 – 1.43 (m, 2H), 1.40 – 1.31 (m, 4H), 0.96 – 0.86 (m, 3H).
^{19}F NMR (377 MHz, $CDCl_3$) δ -61.97.
The spectroscopic data are consistent with the literature and small-scale reaction of compound **5**.[10]

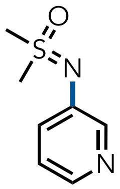

dimethyl(pyridin-3-ylimino)-λ^6-sulfanone (45)
A 20 mL crimp top vial was charged with a magnetic stirring bar, 3-bromopyridine (1.26 g, 8.0 mmol, 1.0 equiv), dimethylsulfoximine (1.12 g, 12.0 mmol, 1.5 equiv) and 16.0 mL of a catalyst stock solution containing 4CzIPN (32.0 mg, 0.04 mmol, 0.005 equiv) and $NiBr_2$•glyme (128.0 mg, 0.4 mmol, 0.05 equiv) dissolved in DMA. The reaction mixture was then degassed and refilled with nitrogen two times via a syringe needle before *tert*-butylamine (1.09 mL, 10.4 mmol, 1.3 equiv) was added via syringe. After degassing one more time and refilling with nitrogen, the reaction mixture was photoirradiated for 5 h at 35 °C through the plane bottom side of the snap vial using a single higher power (see above for specifications) blue LED (455

(± 15) nm). After completion, the reaction mixture was subjected to the **general work-up procedure** and purified using flash chromatography (gradient to 10%–15% MeOH in EtOAc) on silica gel. The title compound was obtained in 91% yield (1.24 g).

Note: *Tert*-butylamine is volatile and therefore, it is recommended to not evacuate the reaction mixture to high vacuum for an extended time period.

^{1}H NMR (400 MHz, $CDCl_3$) δ 8.37 – 8.32 (m, 1H), 8.25 – 8.18 (m, 1H), 7.42 – 7.35 (m, 1H), 7.17 – 7.10 (m, 1H), 3.16 (s, 6H).

The spectroscopic data are consistent with the literature and small-scale reaction of compound **45**.[6]

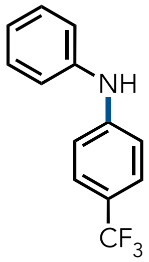

N-phenyl-4-(trifluoromethyl)aniline (58)

A 20 mL crimp top vial was charged with a magnetic stirring bar, 1-bromo-4-(trifluoromethyl)benzene (1.35 g, 6.0 mmol, 1.0 equiv), aniline (838 mg, 9.0 mmol, 1.5 equiv) and 12.0 mL of a catalyst stock solution containing 4CzIPN (24.0 mg, 0.03 mmol, 0.005 equiv) and $NiBr_2$•glyme (96.0 mg, 0.3 mmol, 0.05 equiv) dissolved in DMA. The reaction mixture was then degassed and refilled with nitrogen two times via a syringe needle before *tert*-butylamine (815.0 uL, 7.8 mmol, 1.3 equiv) was added via syringe. After degassing one more time and refilling with nitrogen, the reaction mixture was photoirradiated for 18 h at 60 °C through the plane bottom side of the snap vial using a single higher power (see above for specifications) blue LED (455 (± 15) nm). After completion, the NMR yield (100%) was determined by ^{19}F-NMR using fluorobenzene (3 mmol) as internal standard.

Note: *Tert*-butylamine is volatile and therefore, it is recommended to not evacuate the reaction mixture to high vacuum for an extended time period.

10. NMR Spectra

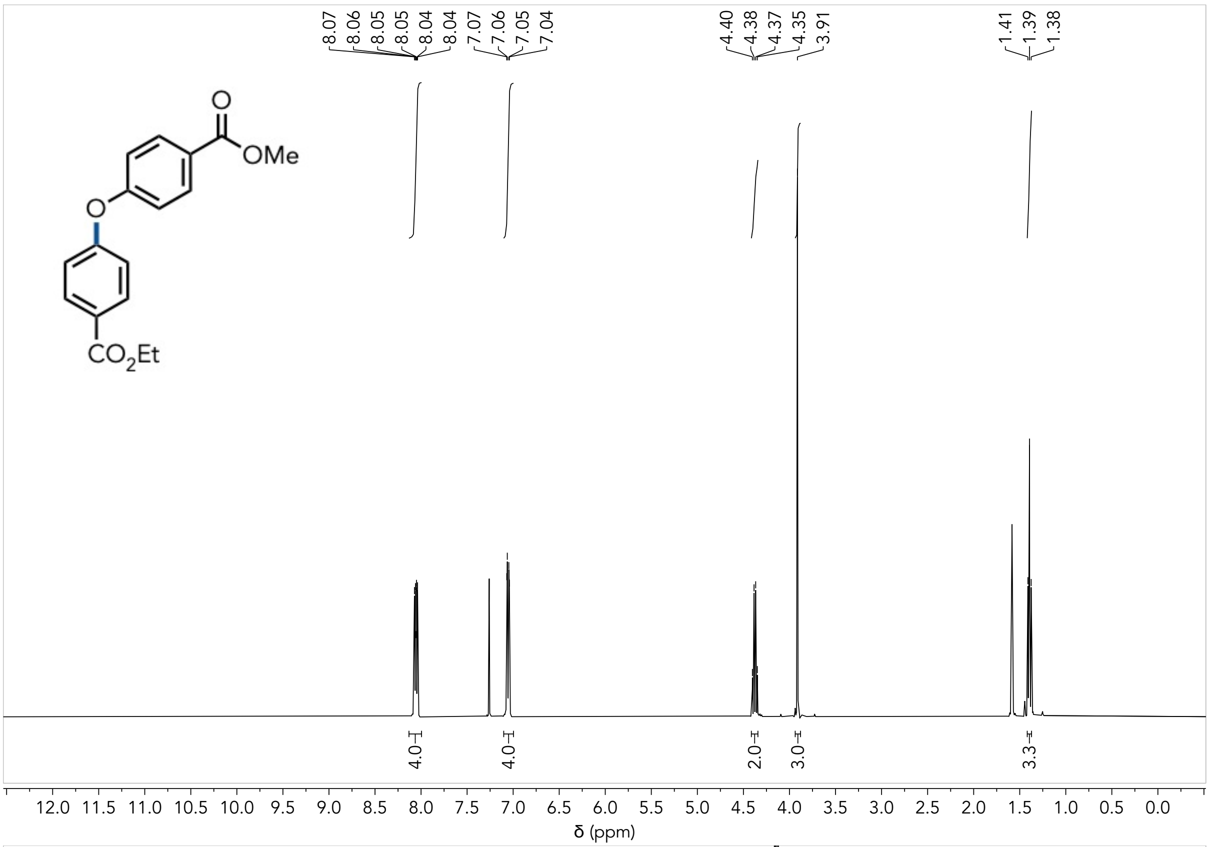

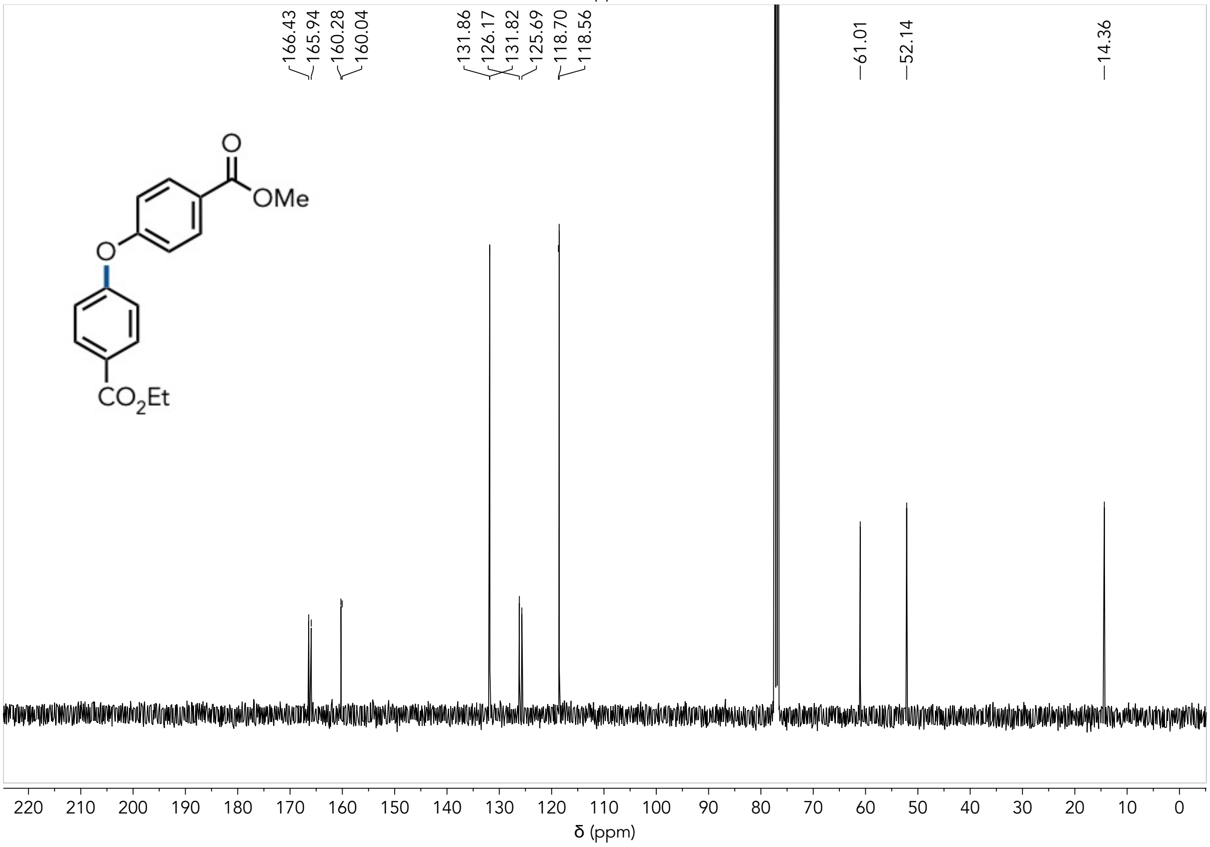

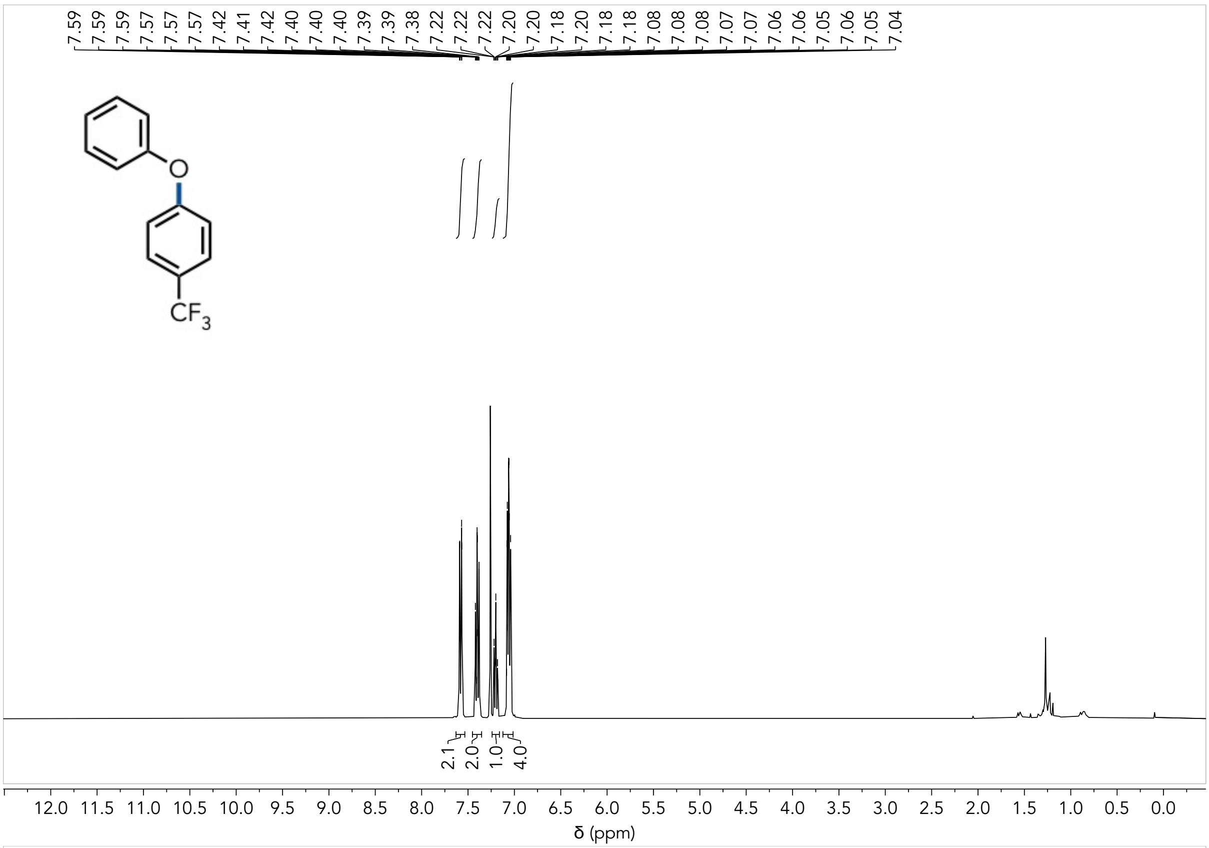
7.59 7.59 7.59 7.57 7.57 7.57 7.42 7.41 7.42 7.40 7.40 7.40 7.39 7.39 7.38 7.22 7.22 7.22 7.20 7.20 7.18 7.20 7.18 7.18 7.08 7.08 7.07 7.07 7.06 7.06 7.05 7.06 7.05 7.04
O
CF3
2.1 2.0 1.0 4.0
12.0 11.5 11.0 10.5 10.0 9.5 9.0 8.5 8.0 7.5 7.0 6.5 6.0 5.5 5.0 4.5 4.0 3.5 3.0 2.5 2.0 1.5 1.0 0.5 0.0
δ (ppm)

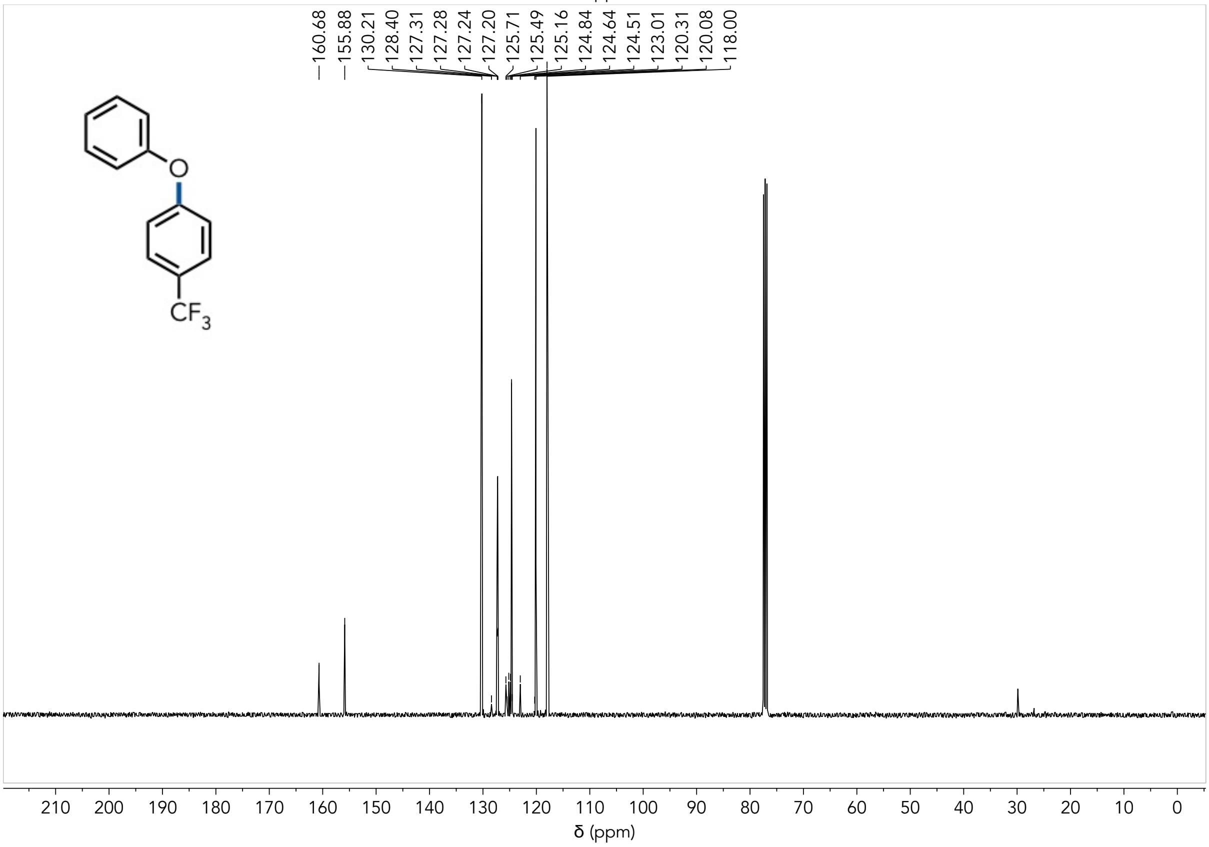
160.68 155.88 130.21 128.40 127.31 127.28 127.24 127.20 125.71 125.49 125.16 124.84 124.64 124.51 123.01 120.31 120.08 118.00
O
CF3
210 200 190 180 170 160 150 140 130 120 110 100 90 80 70 60 50 40 30 20 10 0
δ (ppm)

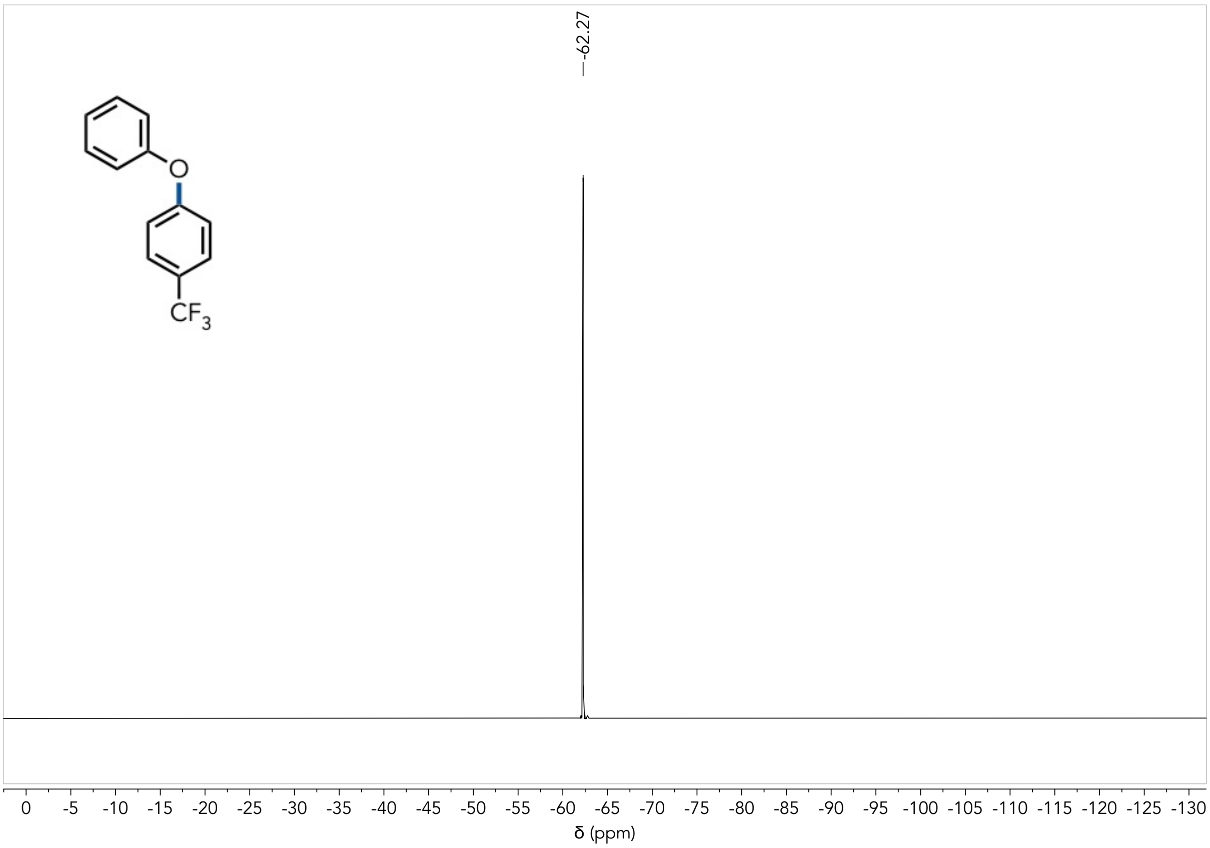
CF3
O
—62.27
0 -5 -10 -15 -20 -25 -30 -35 -40 -45 -50 -55 -60 -65 -70 -75 -80 -85 -90 -95 -100 -105 -110 -115 -120 -125 -130
δ (ppm)

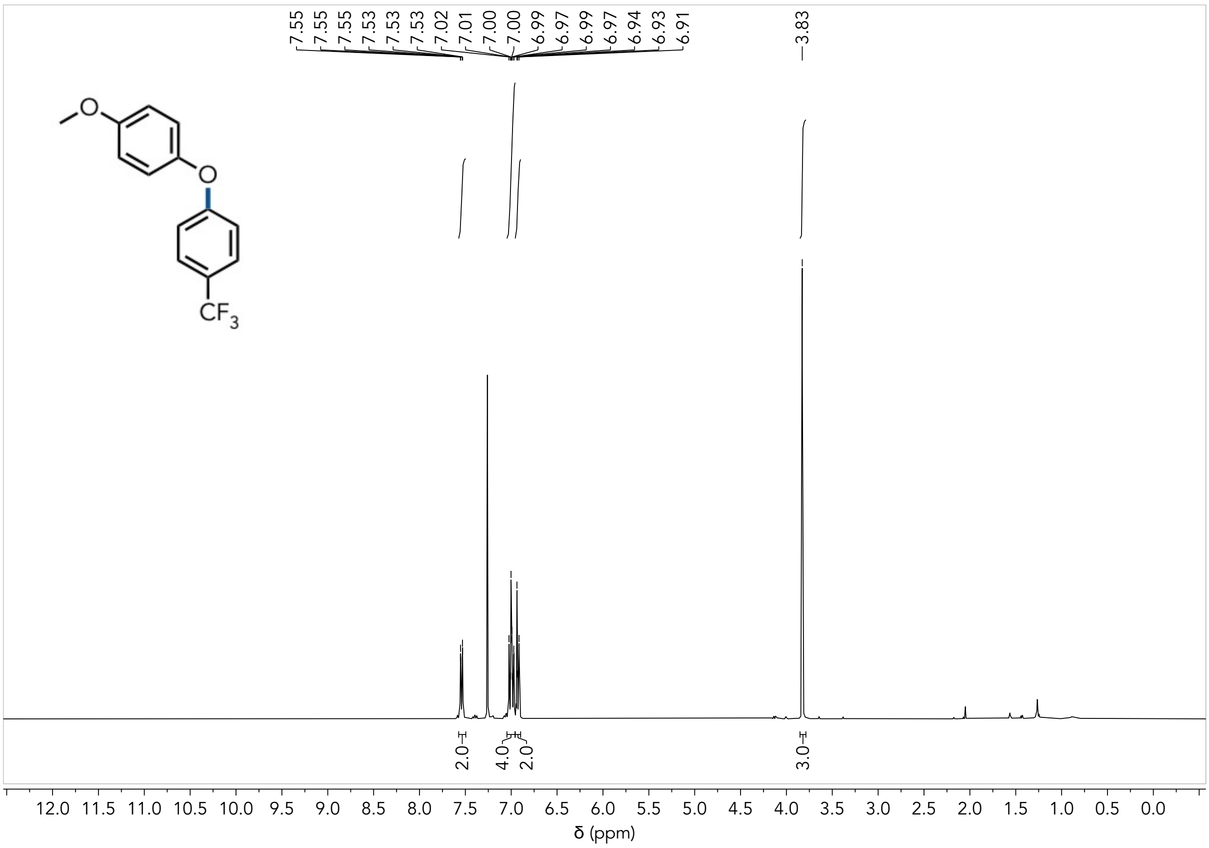
O
O
CF3
7.55 7.55 7.55 7.53 7.53 7.53 7.02 7.01 7.00 7.00 6.99 6.97 6.99 6.97 6.94 6.93 6.91
—3.83
2.0
4.0
2.0
3.0
12.0 11.5 11.0 10.5 10.0 9.5 9.0 8.5 8.0 7.5 7.0 6.5 6.0 5.5 5.0 4.5 4.0 3.5 3.0 2.5 2.0 1.5 1.0 0.5 0.0
δ (ppm)

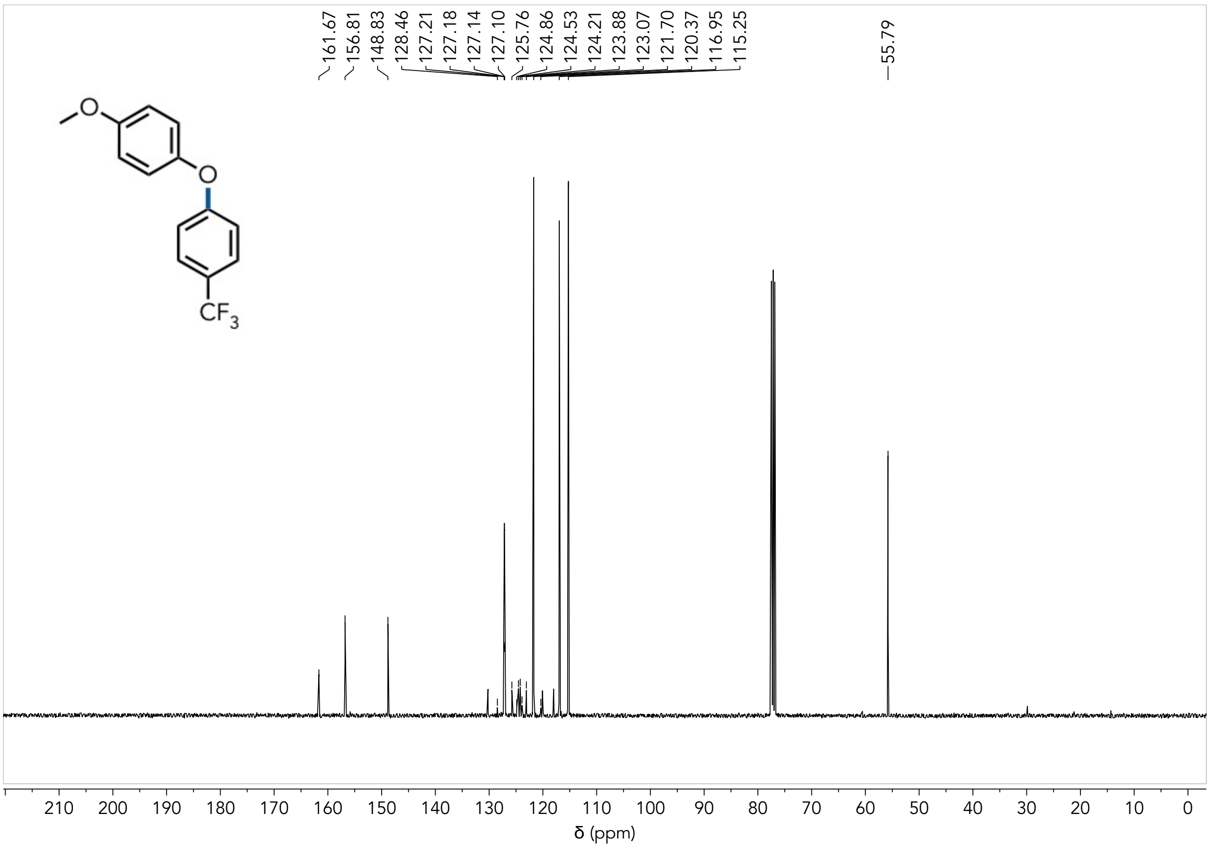
161.67
156.81
148.83
128.46
127.21
127.18
127.14
127.10
125.76
124.86
124.53
124.21
123.88
123.07
121.70
120.37
116.95
115.25
55.79
O
O
CF3
210 200 190 180 170 160 150 140 130 120 110 100 90 80 70 60 50 40 30 20 10 0
δ (ppm)

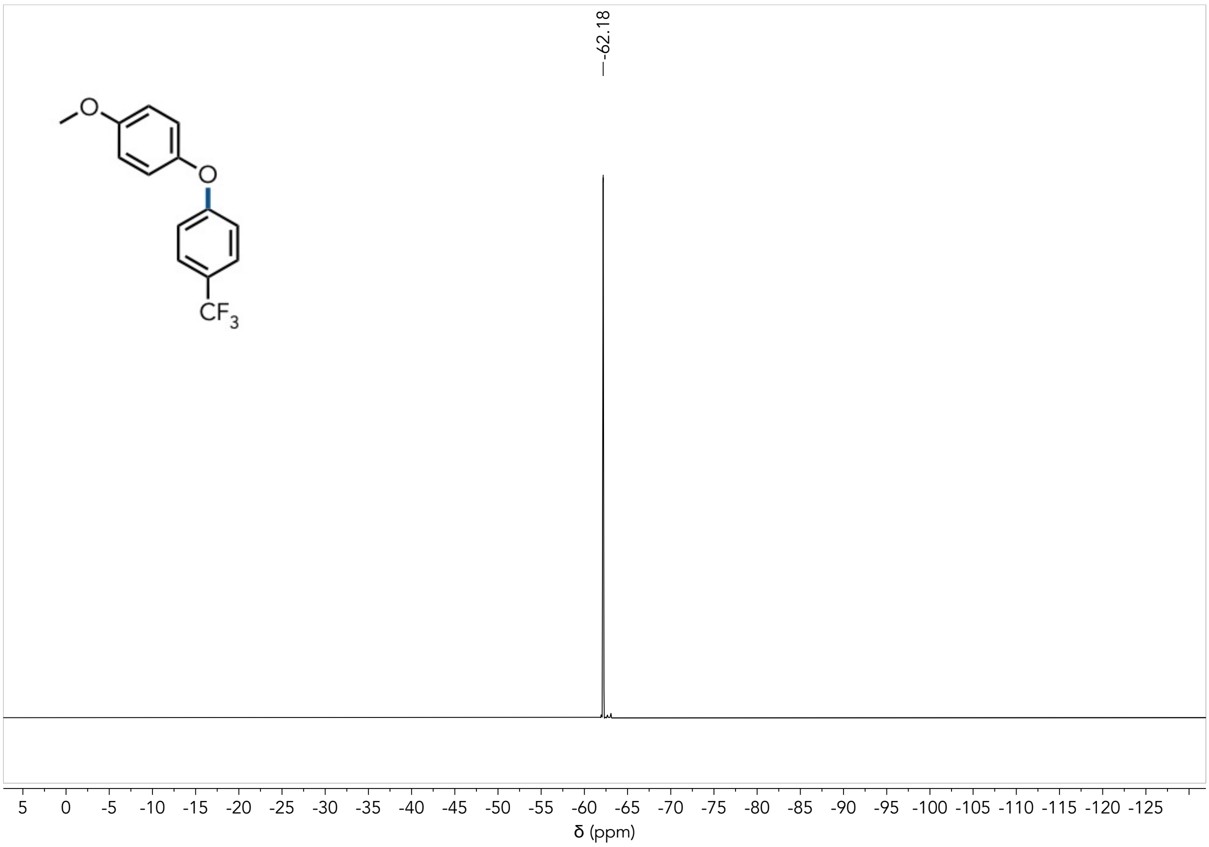
-62.18
O
O
CF3
5 0 -5 -10 -15 -20 -25 -30 -35 -40 -45 -50 -55 -60 -65 -70 -75 -80 -85 -90 -95 -100 -105 -110 -115 -120 -125
δ (ppm)

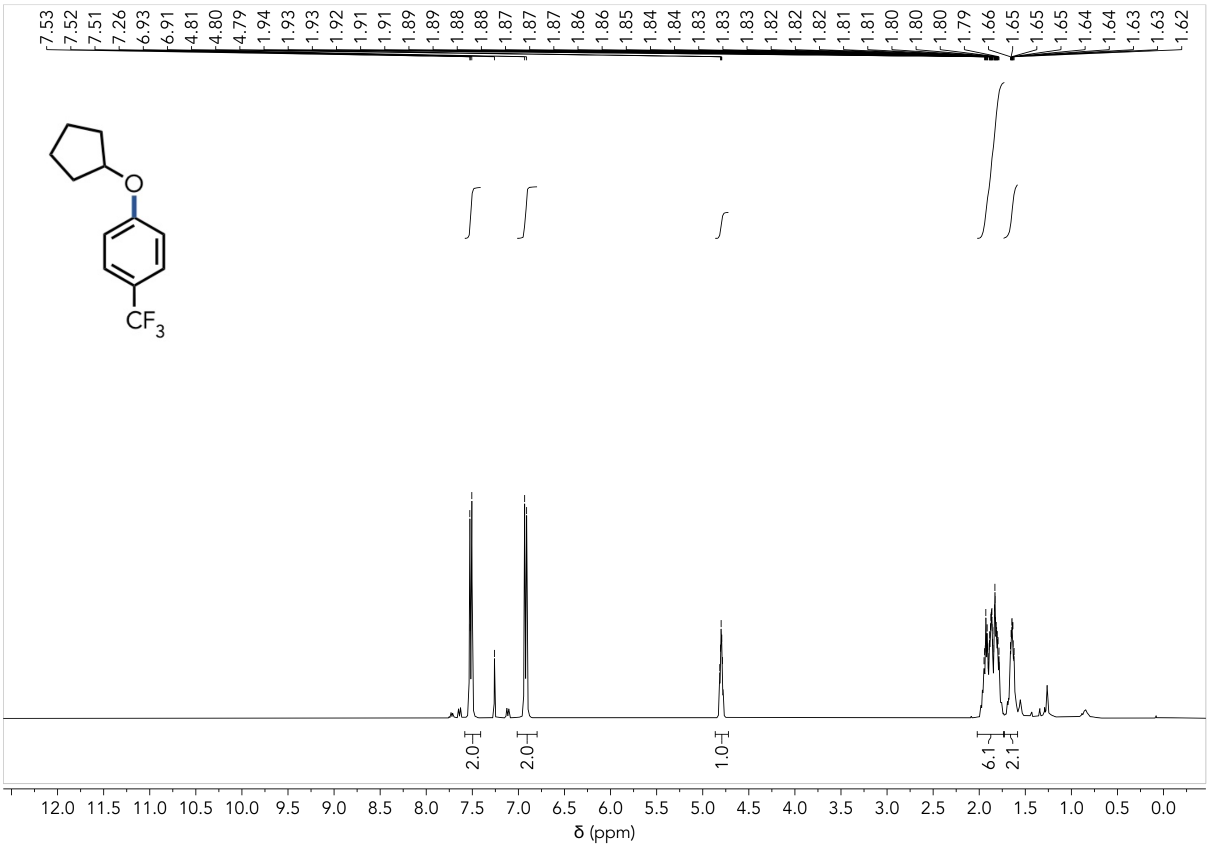

7.53
7.52
7.51
7.26
6.93
6.91
4.81
4.80
4.79
1.94
1.93
1.93
1.92
1.91
1.91
1.89
1.89
1.88
1.88
1.87
1.87
1.87
1.86
1.86
1.85
1.84
1.84
1.83
1.83
1.83
1.82
1.82
1.82
1.81
1.81
1.80
1.80
1.80
1.79
1.66
1.65
1.65
1.65
1.64
1.64
1.63
1.63
1.62
O
CF3
2.0
2.0
1.0
6.1
2.1
12.0 11.5 11.0 10.5 10.0 9.5 9.0 8.5 8.0 7.5 7.0 6.5 6.0 5.5 5.0 4.5 4.0 3.5 3.0 2.5 2.0 1.5 1.0 0.5 0.0
δ (ppm)

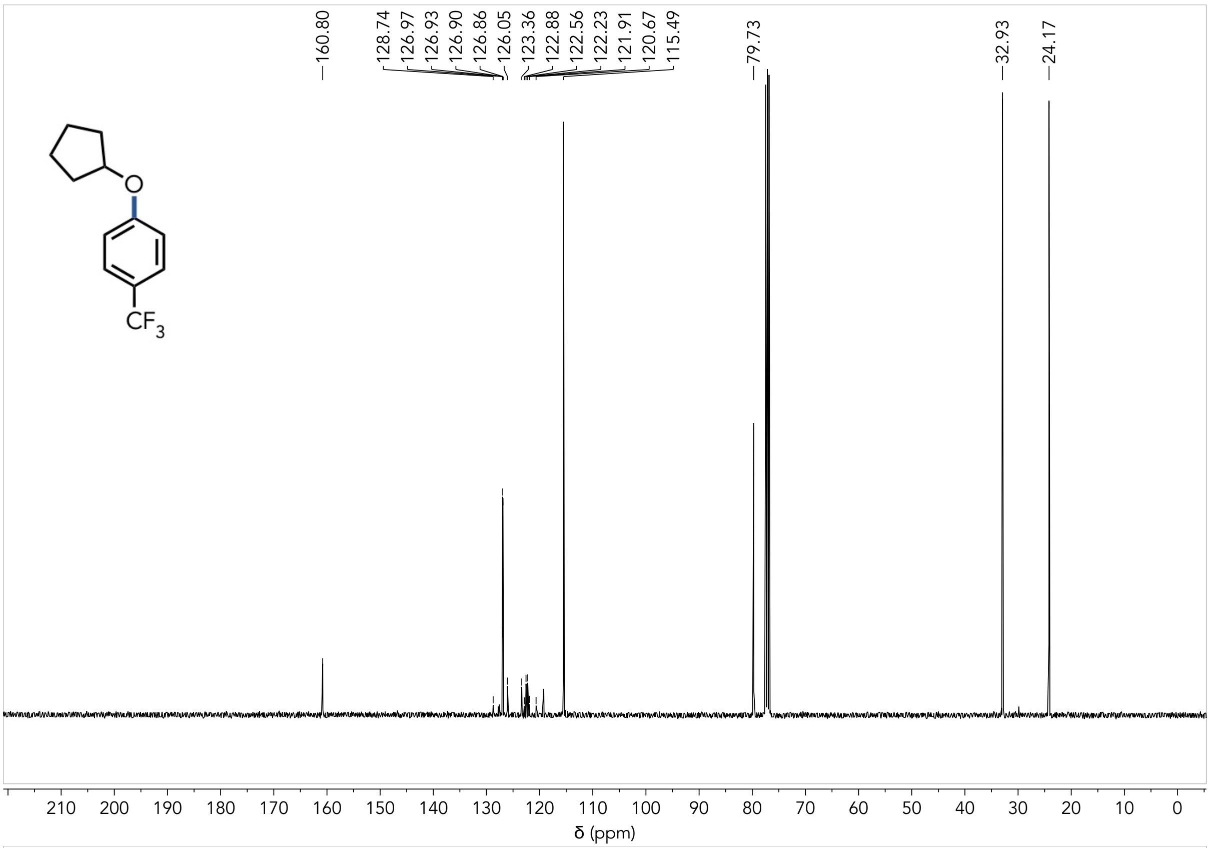
160.80
128.74
126.97
126.93
126.90
126.86
126.05
123.36
122.88
122.56
122.23
121.91
120.67
115.49
79.73
32.93
24.17
O
CF3
210 200 190 180 170 160 150 140 130 120 110 100 90 80 70 60 50 40 30 20 10 0
δ (ppm)

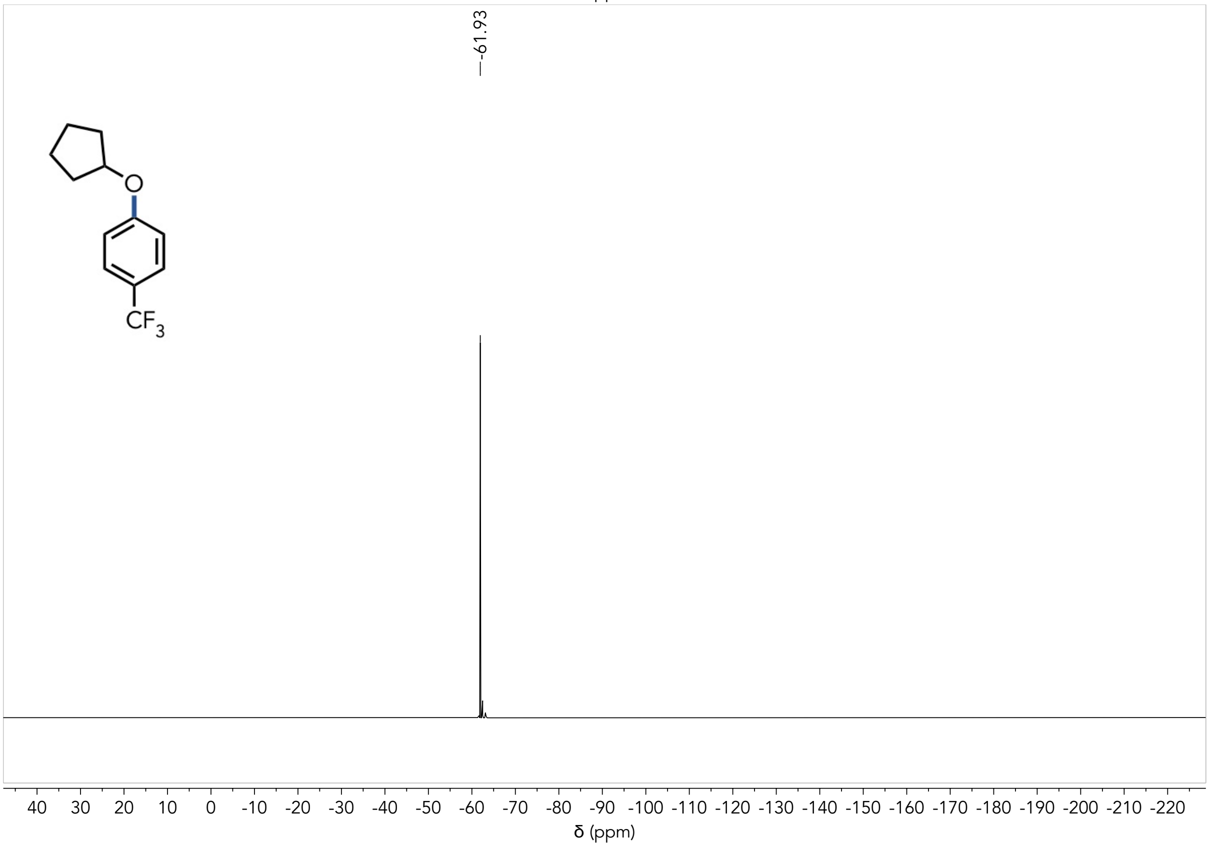
-61.93
O
CF3
40 30 20 10 0 -10 -20 -30 -40 -50 -60 -70 -80 -90 -100 -110 -120 -130 -140 -150 -160 -170 -180 -190 -200 -210 -220
δ (ppm)

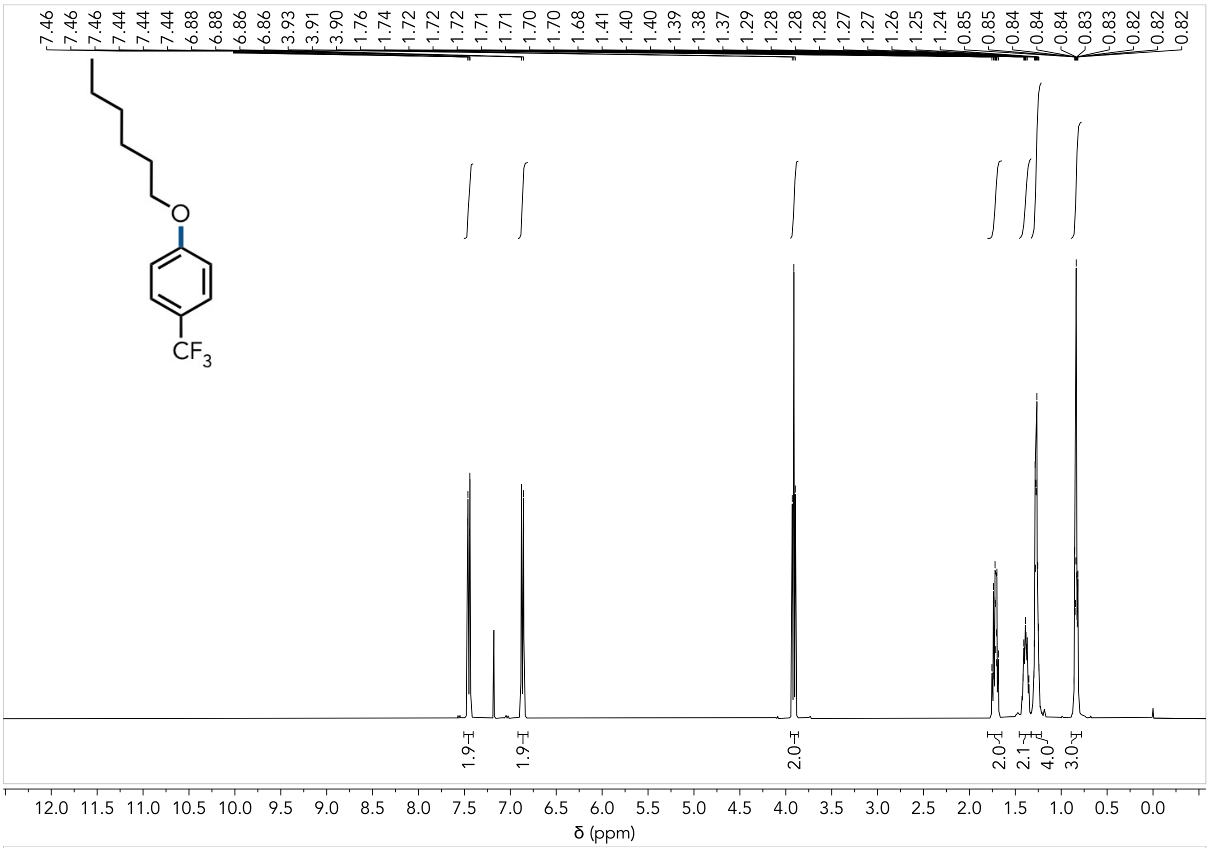
7.46
7.46
7.46
7.44
7.44
7.44
6.88
6.88
6.86
6.86
3.93
3.91
3.90
1.76
1.74
1.72
1.72
1.72
1.71
1.71
1.70
1.70
1.68
1.41
1.40
1.40
1.39
1.38
1.37
1.29
1.28
1.28
1.28
1.27
1.27
1.26
1.25
1.24
0.85
0.85
0.84
0.84
0.84
0.83
0.83
0.82
0.82
0.82
O
CF_3
1.9
1.9
2.0
2.0
2.1
4.0
3.0
12.0 11.5 11.0 10.5 10.0 9.5 9.0 8.5 8.0 7.5 7.0 6.5 6.0 5.5 5.0 4.5 4.0 3.5 3.0 2.5 2.0 1.5 1.0 0.5 0.0
δ (ppm)

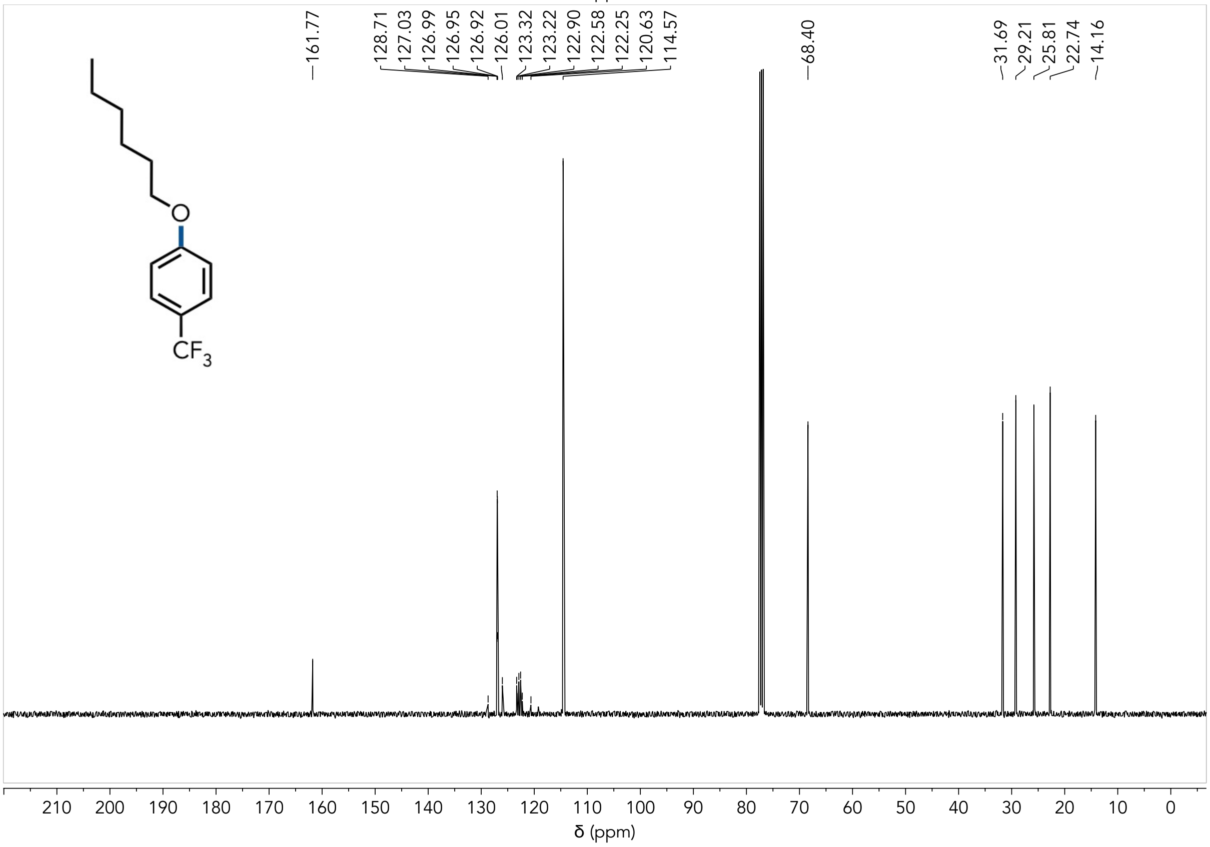
161.77
128.71
127.03
126.99
126.95
126.92
126.01
123.32
123.22
122.90
122.58
122.25
120.63
114.57
68.40
31.69
29.21
25.81
22.74
14.16
O
CF_3
210 200 190 180 170 160 150 140 130 120 110 100 90 80 70 60 50 40 30 20 10 0
δ (ppm)

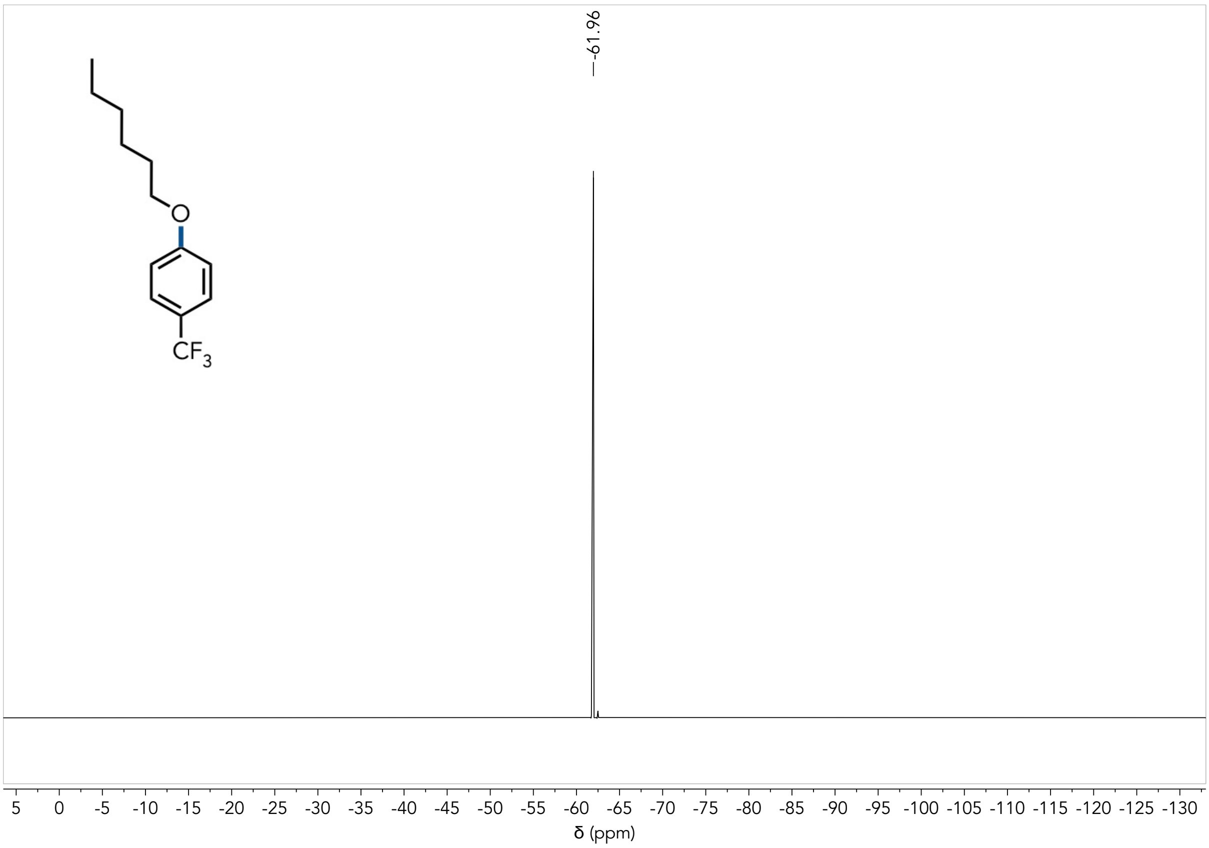

-61.96
CF3
5 0 -5 -10 -15 -20 -25 -30 -35 -40 -45 -50 -55 -60 -65 -70 -75 -80 -85 -90 -95 -100 -105 -110 -115 -120 -125 -130
δ (ppm)

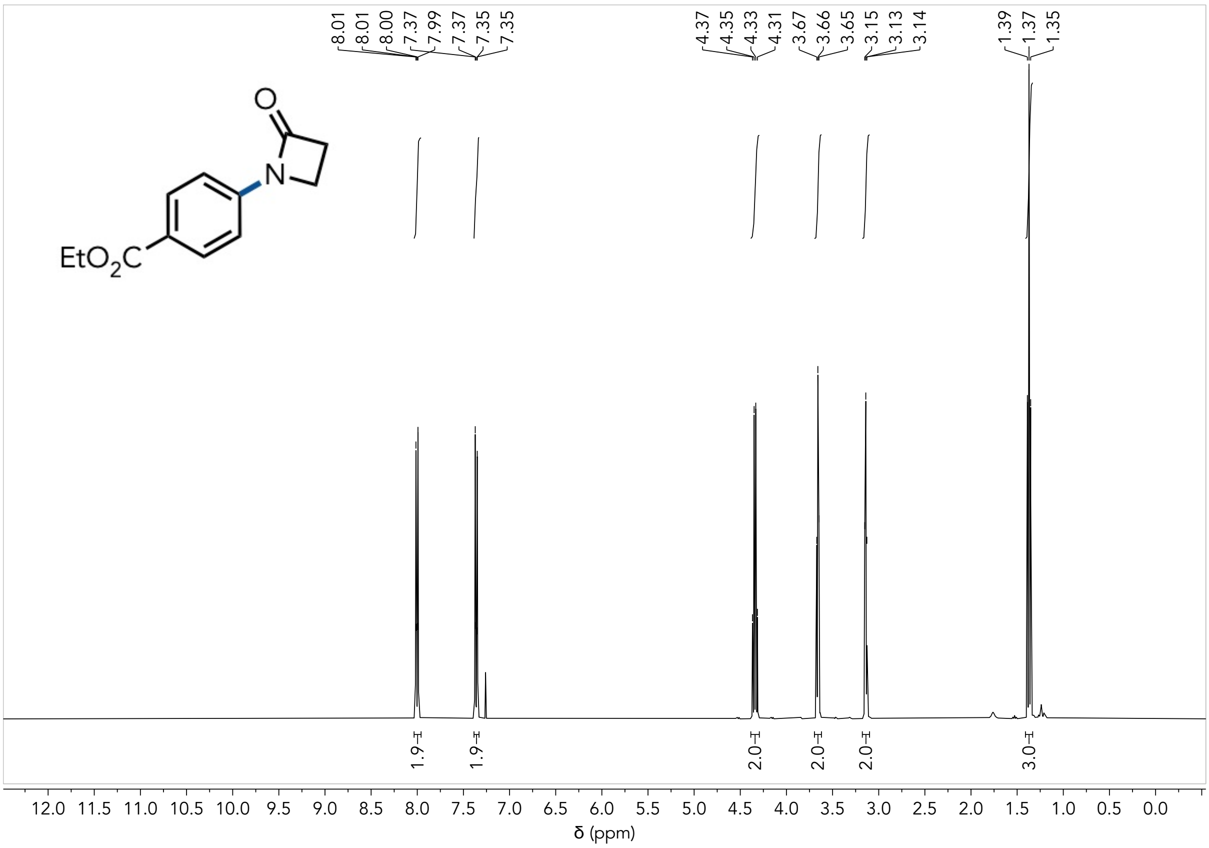

8.01 8.01 8.00 7.37 7.99 7.37 7.35 7.35
4.37 4.35 4.33 4.31 3.67 3.66 3.65 3.15 3.13 3.14
1.39 1.37 1.35
EtO2C
N
O
1.9 1.9 2.0 2.0 2.0 3.0
12.0 11.5 11.0 10.5 10.0 9.5 9.0 8.5 8.0 7.5 7.0 6.5 6.0 5.5 5.0 4.5 4.0 3.5 3.0 2.5 2.0 1.5 1.0 0.5 0.0
δ (ppm)

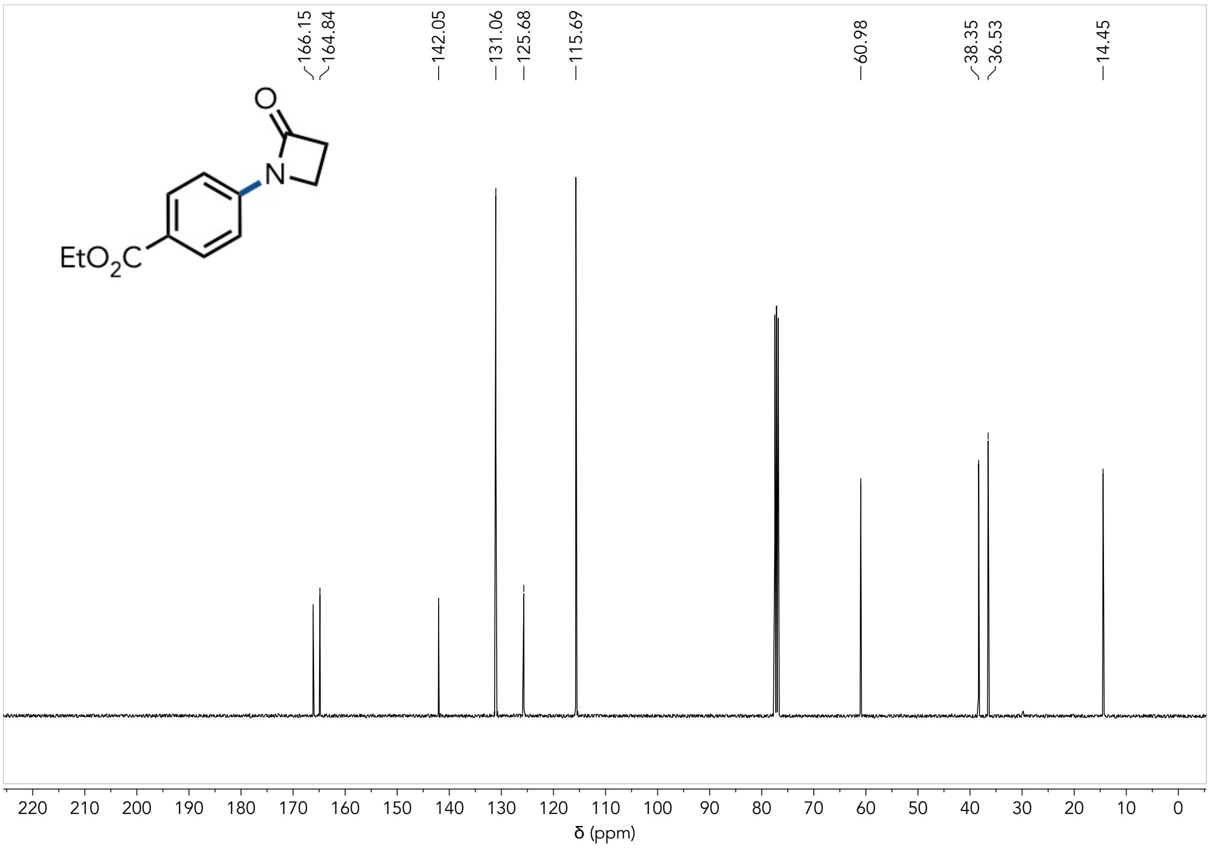
166.15
164.84
142.05
131.06
125.68
115.69
60.98
38.35
36.53
14.45
EtO2C
N
O
220 210 200 190 180 170 160 150 140 130 120 110 100 90 80 70 60 50 40 30 20 10 0
δ (ppm)

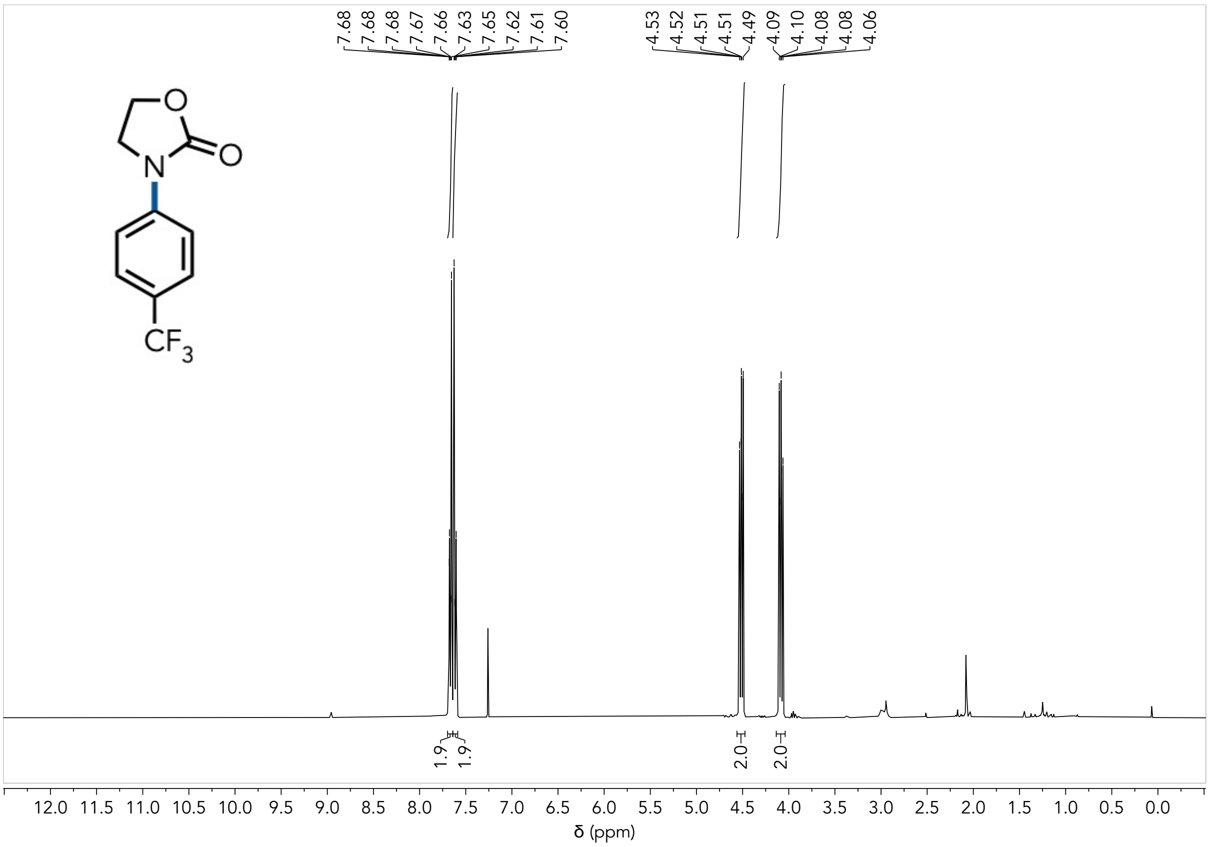
7.68
7.68
7.68
7.67
7.66
7.63
7.65
7.62
7.61
7.60
4.53
4.52
4.51
4.51
4.49
4.09
4.10
4.08
4.08
4.06
O
N
O
CF3
1.9
1.9
2.0
2.0
12.0 11.5 11.0 10.5 10.0 9.5 9.0 8.5 8.0 7.5 7.0 6.5 6.0 5.5 5.0 4.5 4.0 3.5 3.0 2.5 2.0 1.5 1.0 0.5 0.0
δ (ppm)

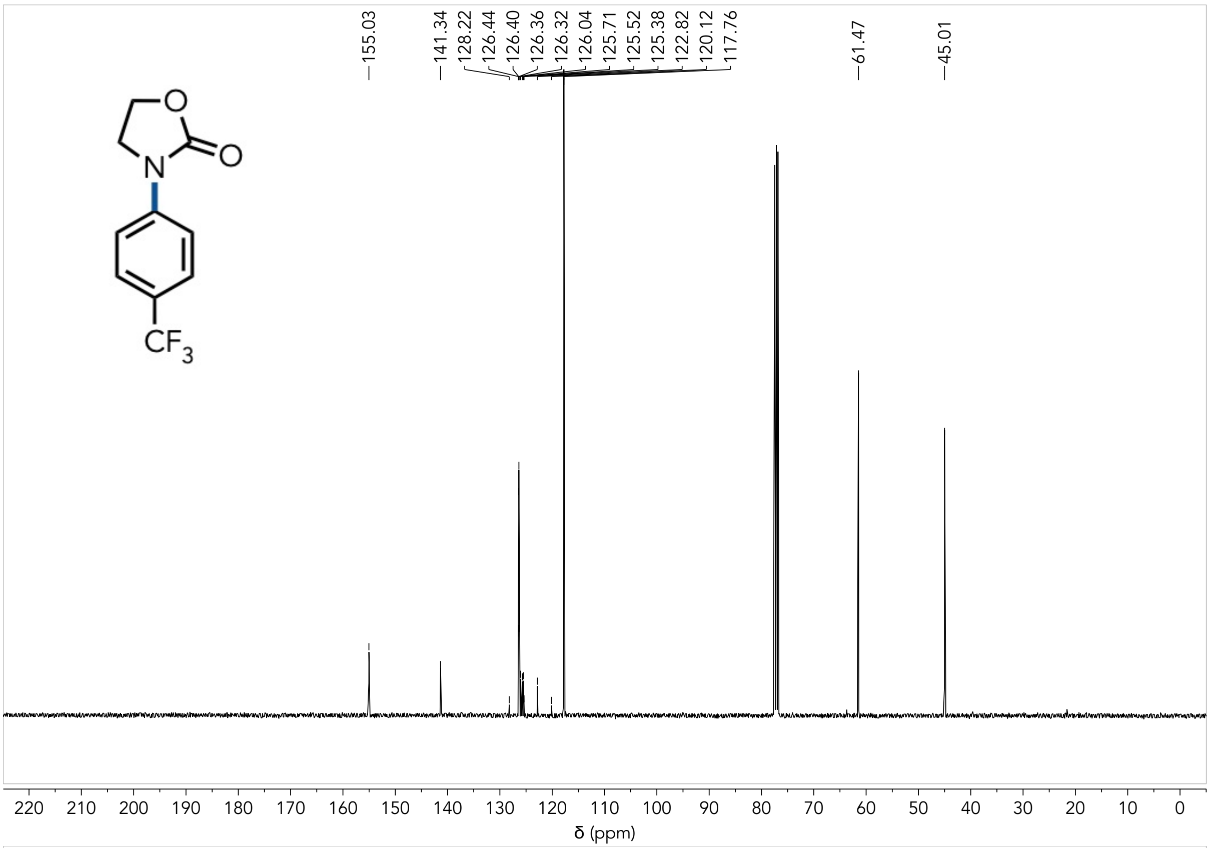
155.03
141.34
128.22
126.44
126.40
126.36
126.32
126.04
125.71
125.52
125.38
122.82
120.12
117.76
61.47
45.01
O
N
O
CF_3
220 210 200 190 180 170 160 150 140 130 120 110 100 90 80 70 60 50 40 30 20 10 0
δ (ppm)

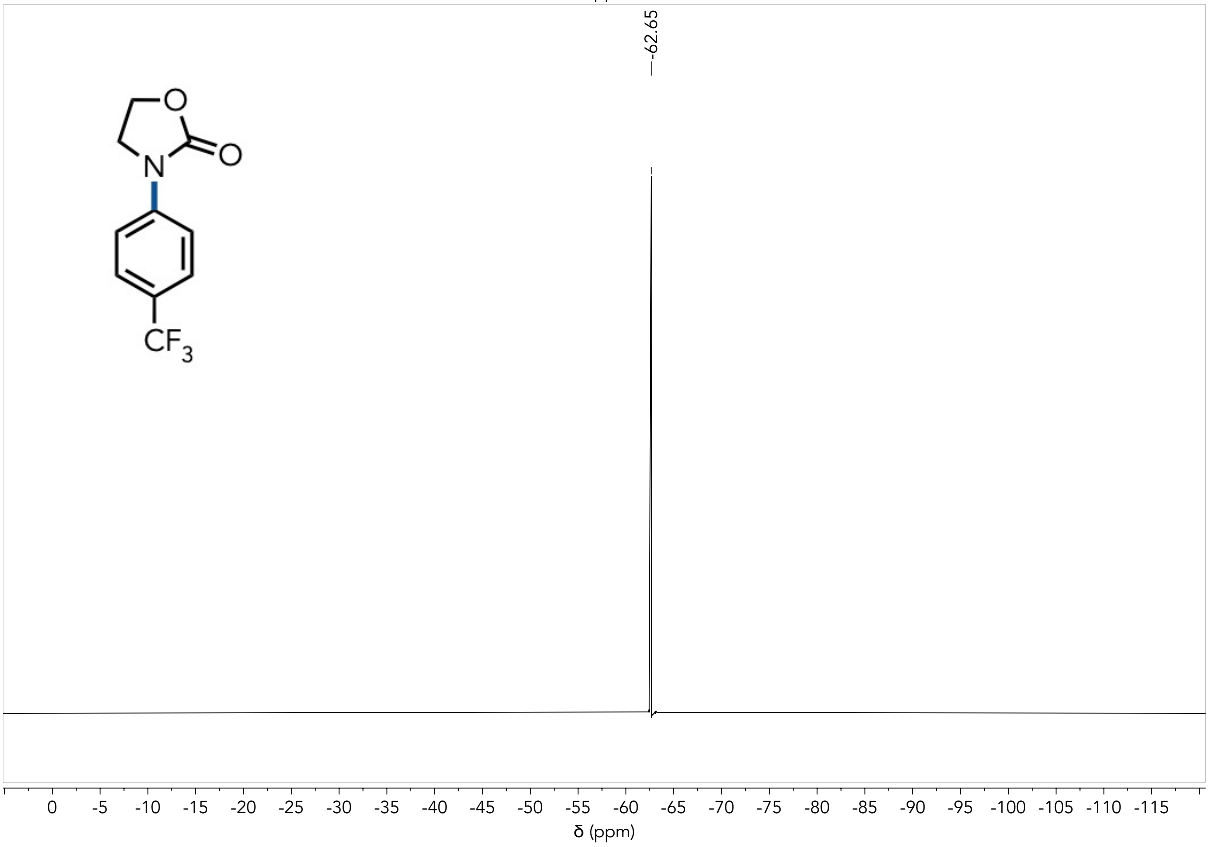
-62.65
O
N
O
CF_3
0 -5 -10 -15 -20 -25 -30 -35 -40 -45 -50 -55 -60 -65 -70 -75 -80 -85 -90 -95 -100 -105 -110 -115
δ (ppm)

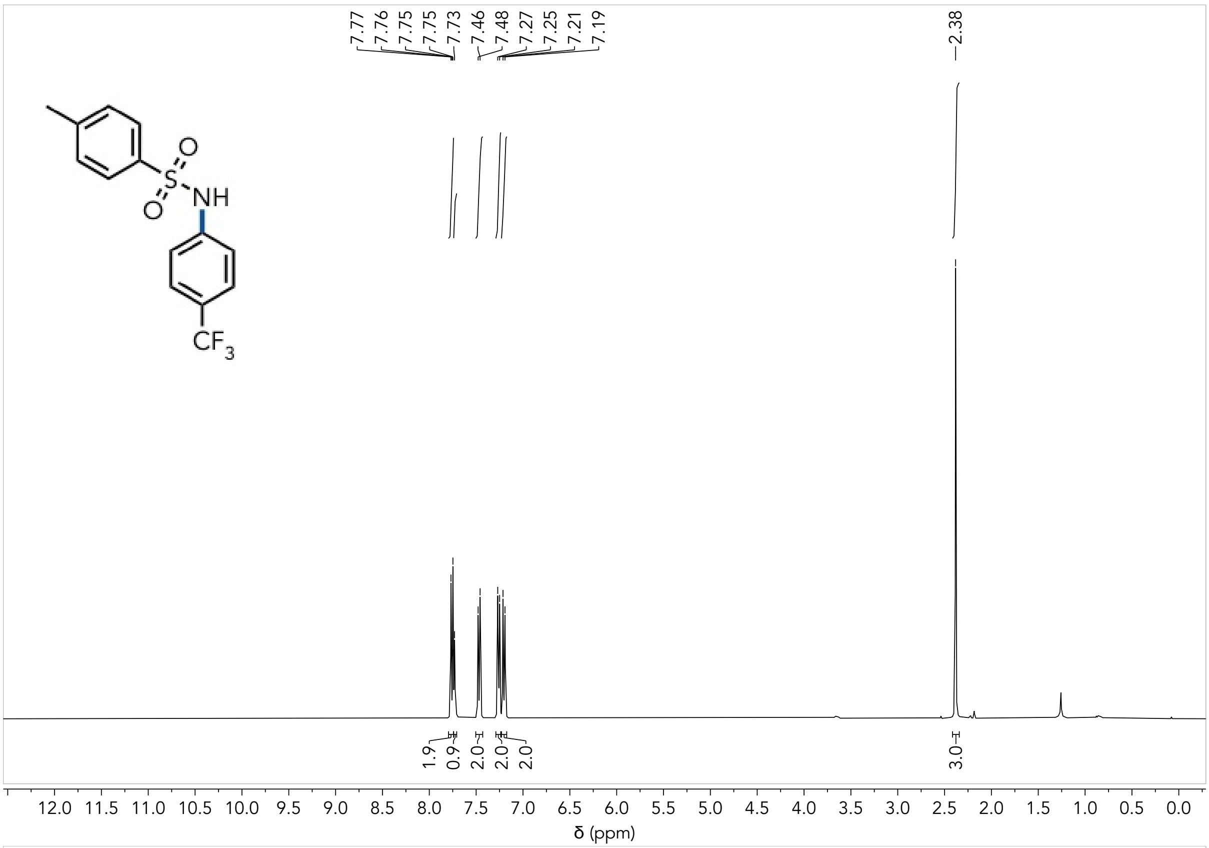
7.77
7.76
7.75
7.75
7.73
7.46
7.48
7.27
7.25
7.21
7.19
2.38
S
O
O
NH
CF3
1.9
0.9
2.0
2.0
2.0
3.0
12.0 11.5 11.0 10.5 10.0 9.5 9.0 8.5 8.0 7.5 7.0 6.5 6.0 5.5 5.0 4.5 4.0 3.5 3.0 2.5 2.0 1.5 1.0 0.5 0.0
δ (ppm)

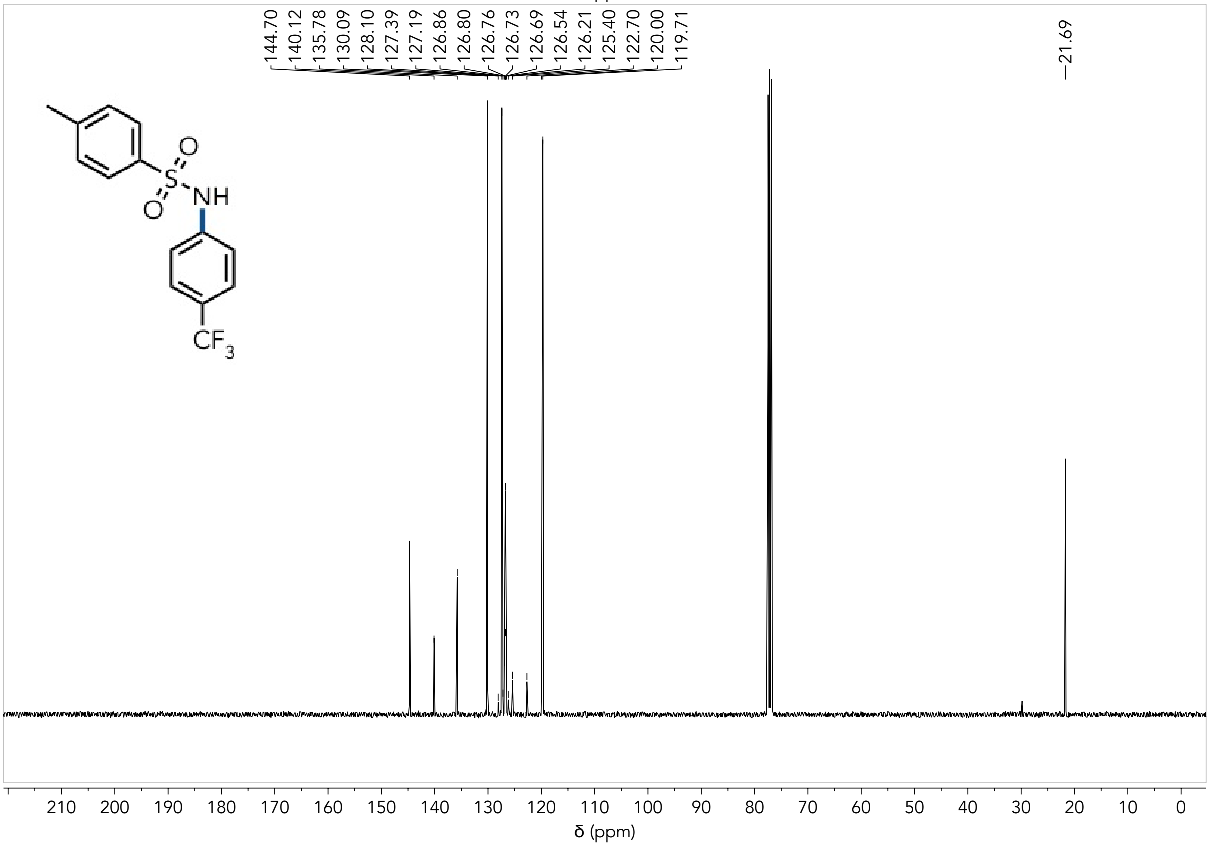
144.70
140.12
135.78
130.09
128.10
127.39
127.19
126.86
126.80
126.76
126.73
126.69
126.54
126.21
125.40
122.70
120.00
119.71
21.69
S
O
O
NH
CF3
210 200 190 180 170 160 150 140 130 120 110 100 90 80 70 60 50 40 30 20 10 0
δ (ppm)

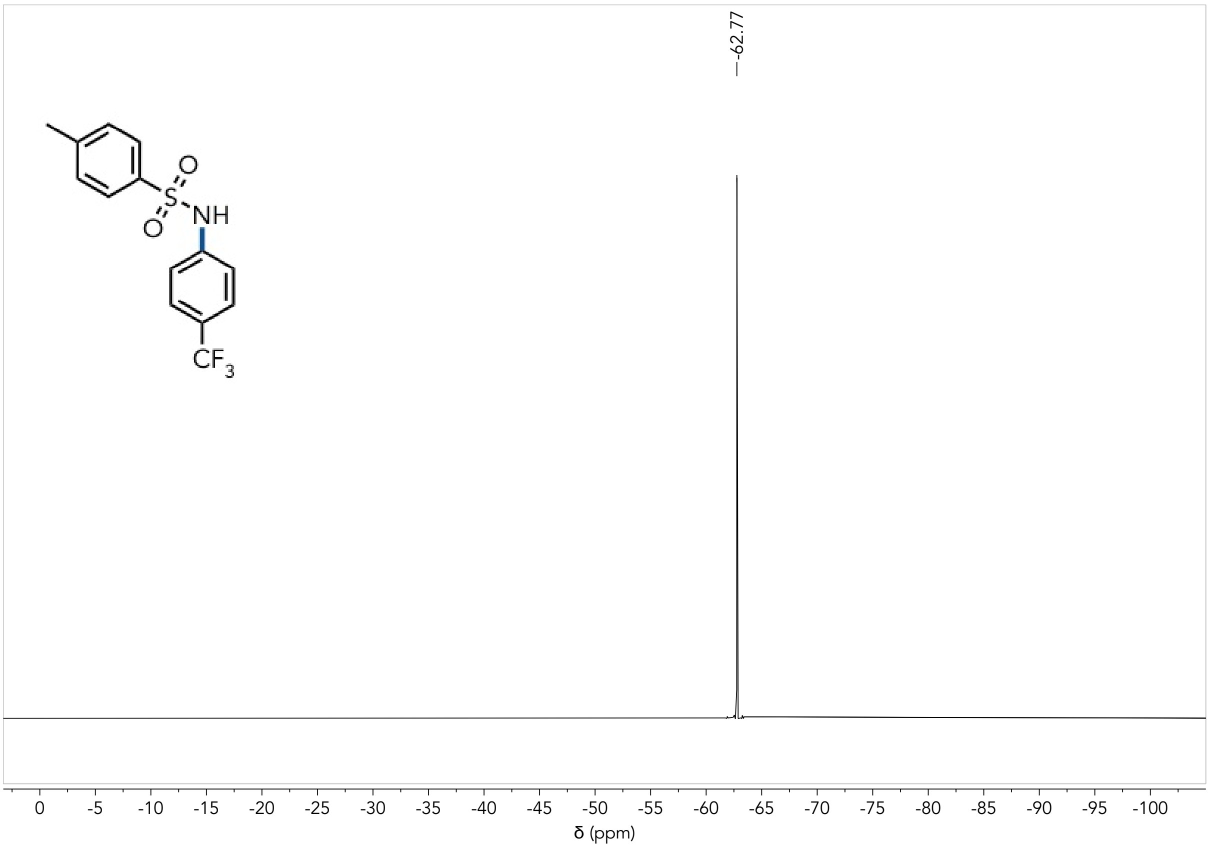

-62.77
O
S
NH
O
CF3
0 -5 -10 -15 -20 -25 -30 -35 -40 -45 -50 -55 -60 -65 -70 -75 -80 -85 -90 -95 -100
δ (ppm)

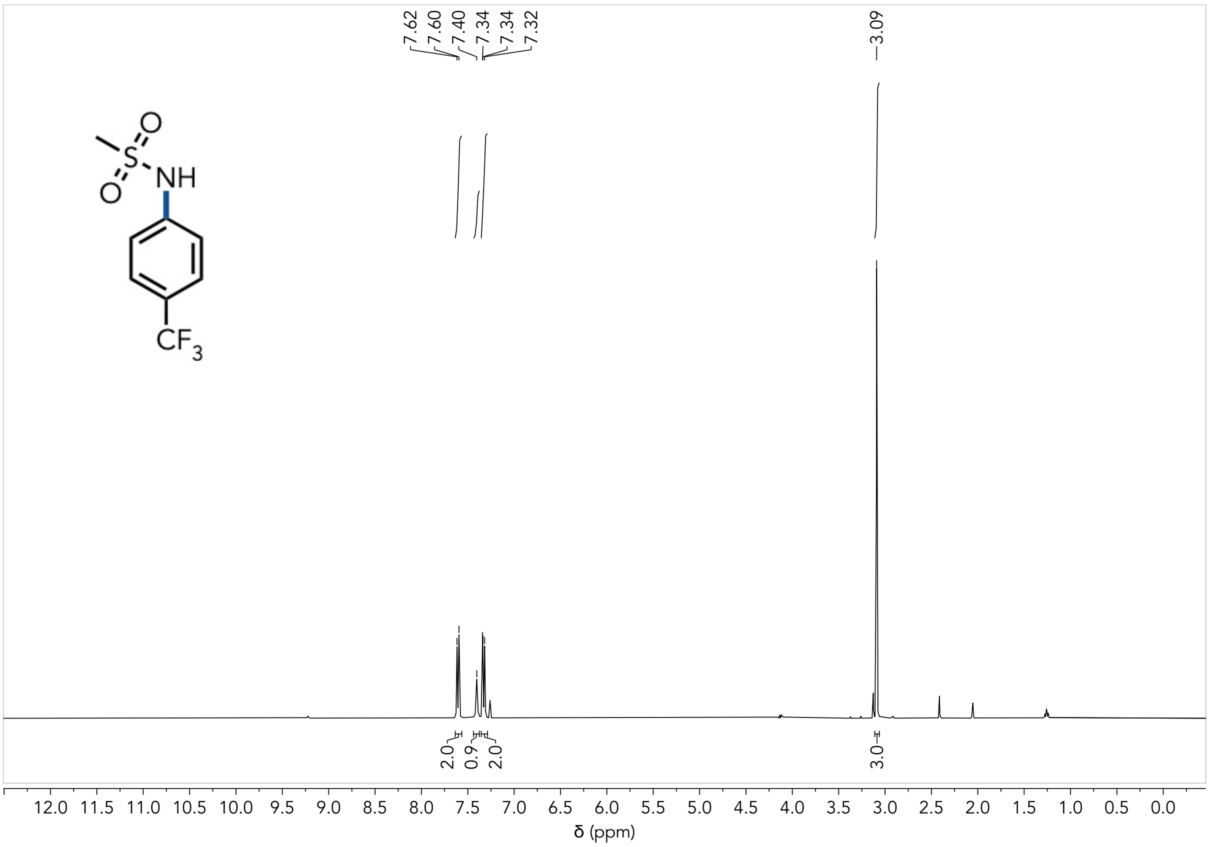

7.62
7.60
7.40
7.34
7.34
7.32
3.09
O
S
NH
O
CF3
2.0
0.9
2.0
3.0
12.0 11.5 11.0 10.5 10.0 9.5 9.0 8.5 8.0 7.5 7.0 6.5 6.0 5.5 5.0 4.5 4.0 3.5 3.0 2.5 2.0 1.5 1.0 0.5 0.0
δ (ppm)

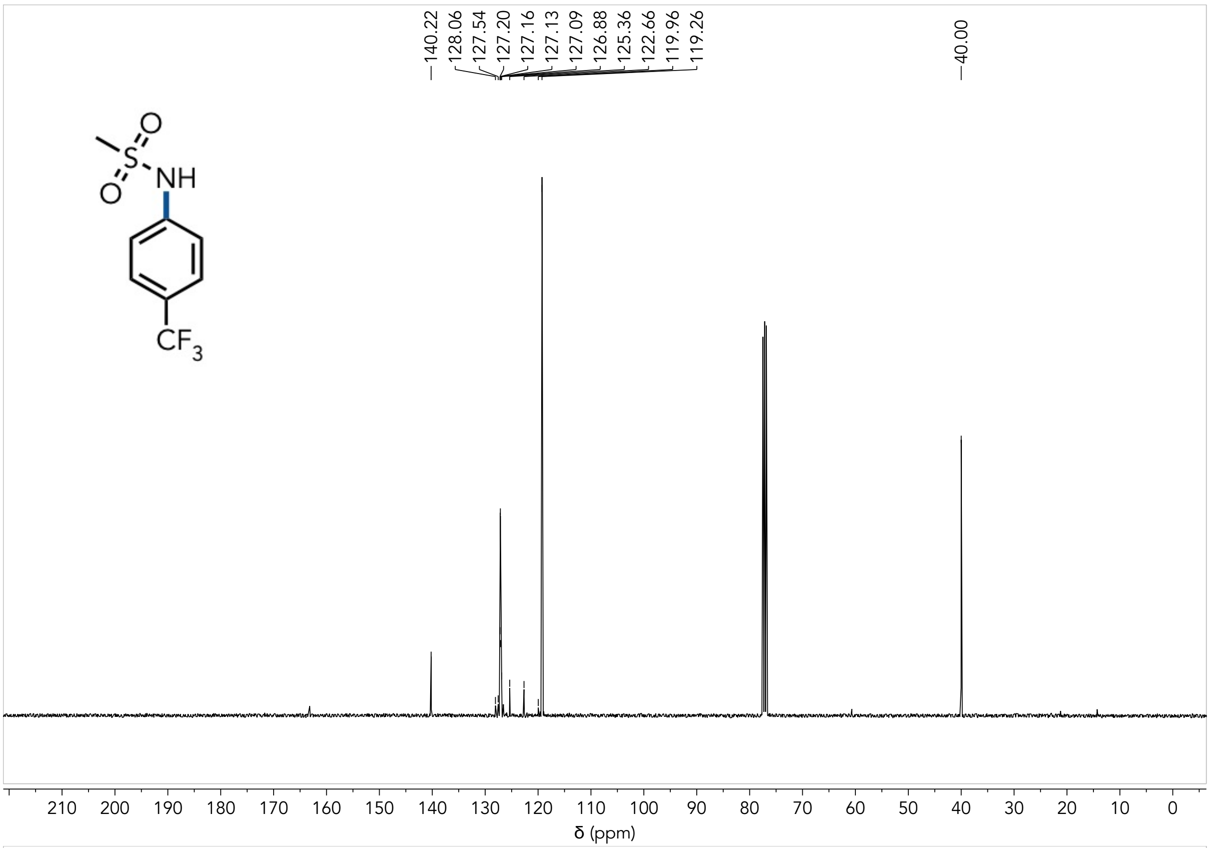
140.22
128.06
127.54
127.20
127.16
127.13
127.09
126.88
125.36
122.66
119.96
119.26
40.00
O
S
NH
O
CF3
δ (ppm)

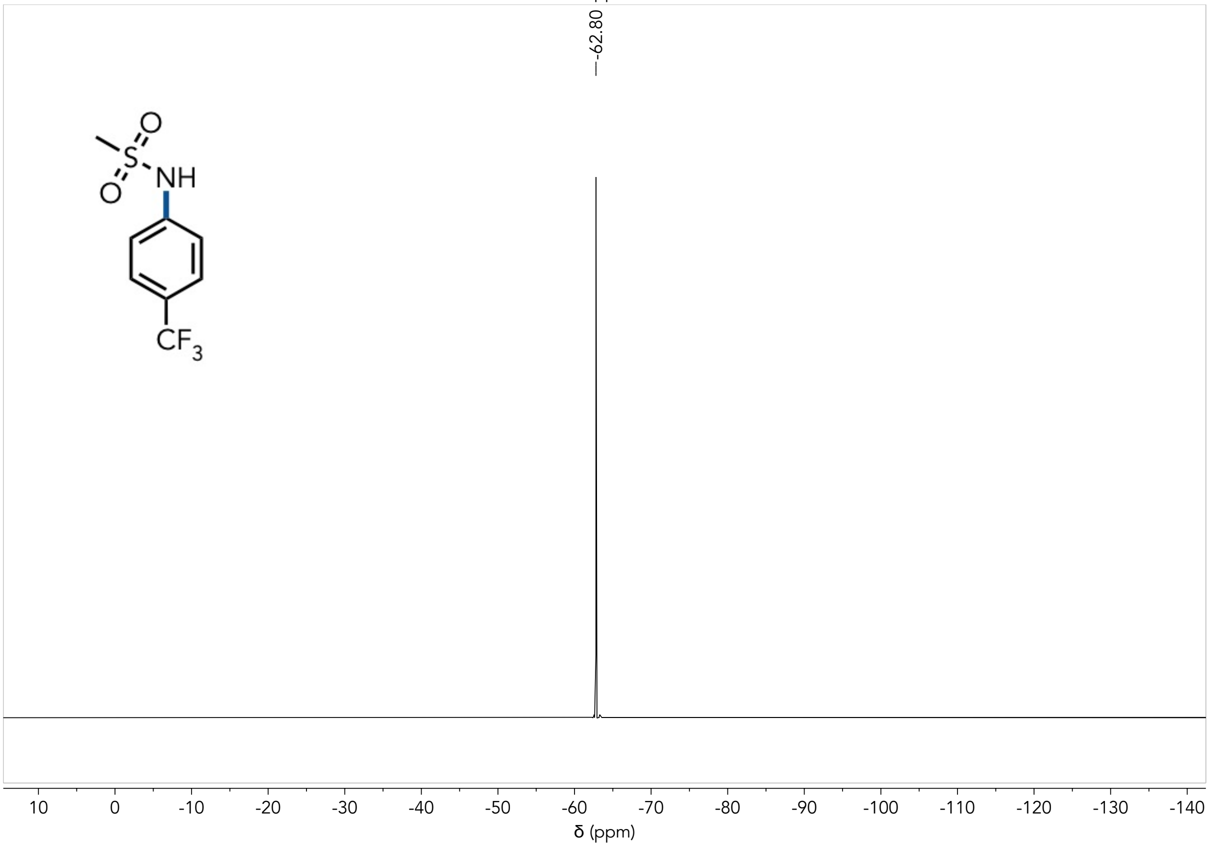
-62.80
O
S
NH
O
CF3
δ (ppm)

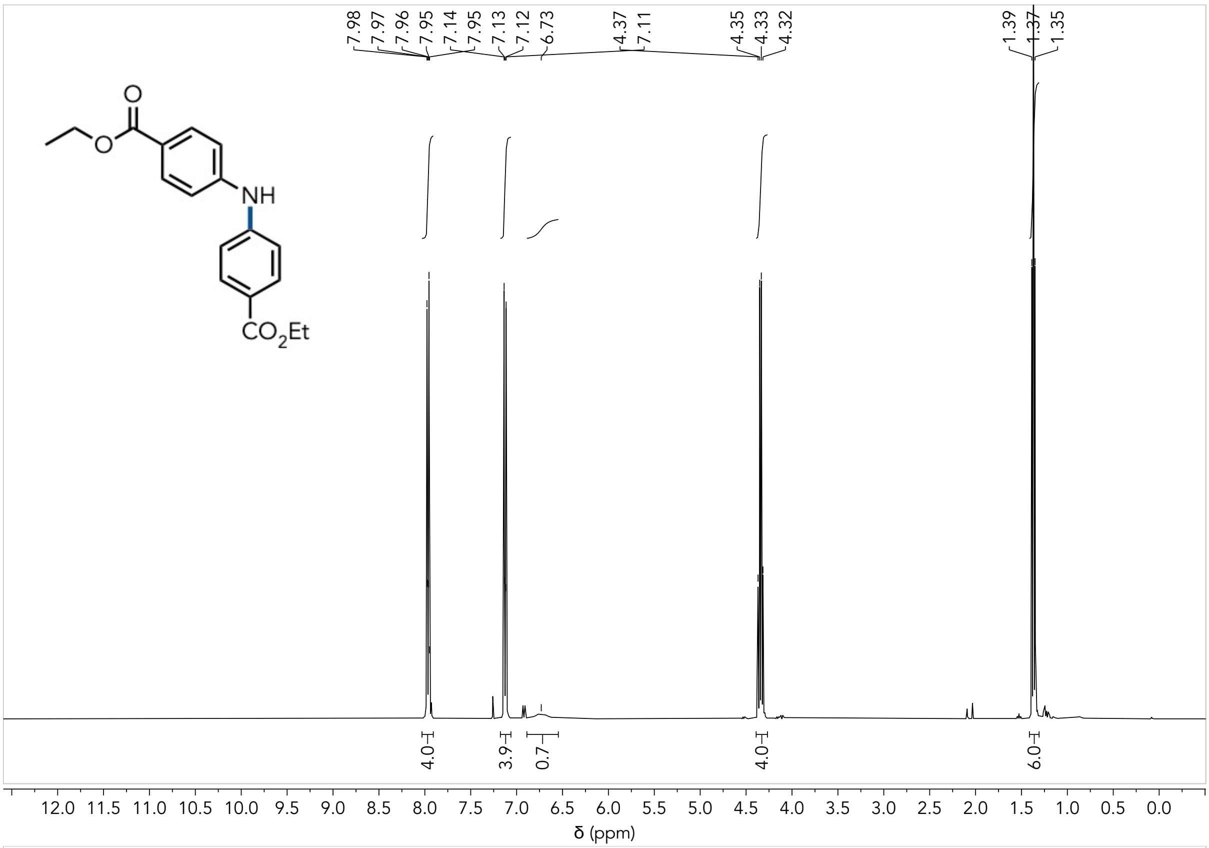

7.98
7.97
7.96
7.95
7.14
7.95
7.13
7.12
6.73
4.37
7.11
4.35
4.33
4.32
1.39
1.37
1.35
NH
CO2Et
4.0
3.9
0.7
4.0
6.0
12.0 11.5 11.0 10.5 10.0 9.5 9.0 8.5 8.0 7.5 7.0 6.5 6.0 5.5 5.0 4.5 4.0 3.5 3.0 2.5 2.0 1.5 1.0 0.5 0.0
δ (ppm)

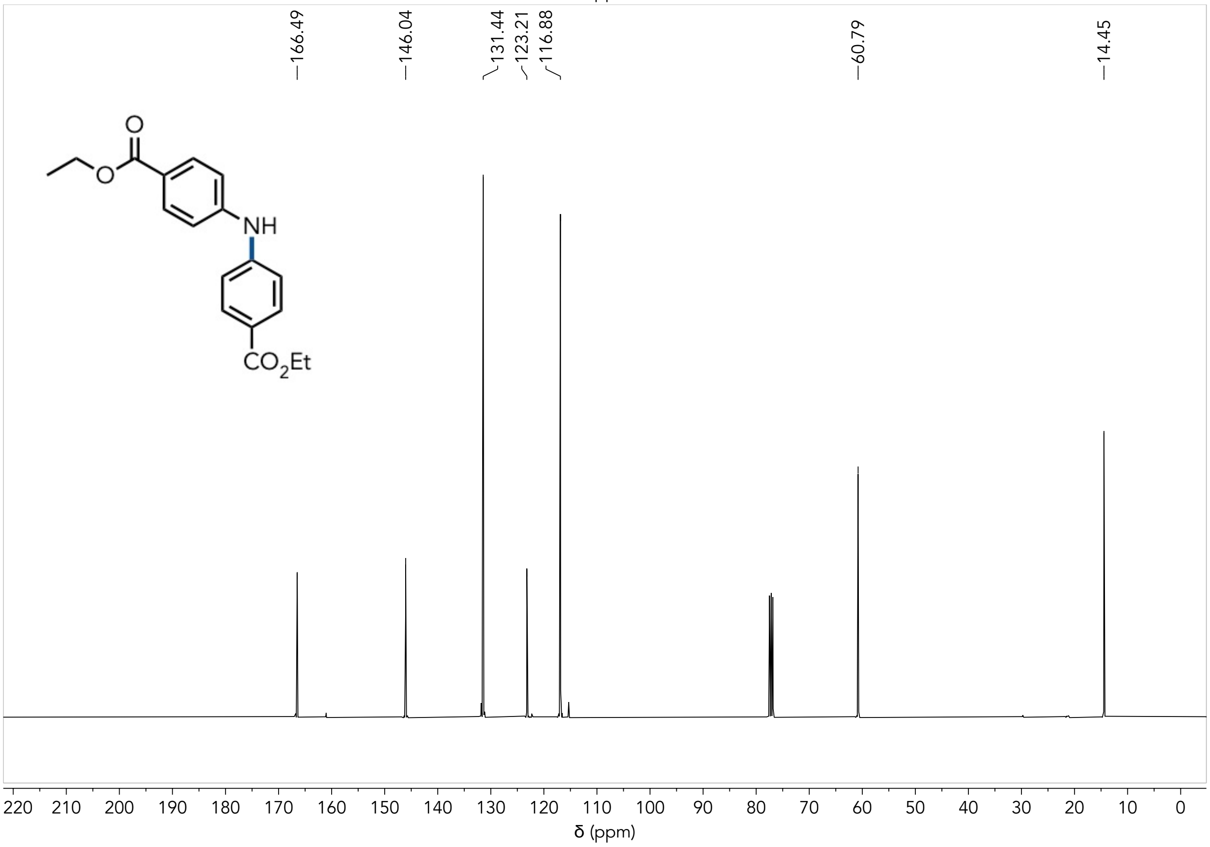

166.49
146.04
131.44
123.21
116.88
60.79
14.45
NH
CO2Et
220 210 200 190 180 170 160 150 140 130 120 110 100 90 80 70 60 50 40 30 20 10 0
δ (ppm)

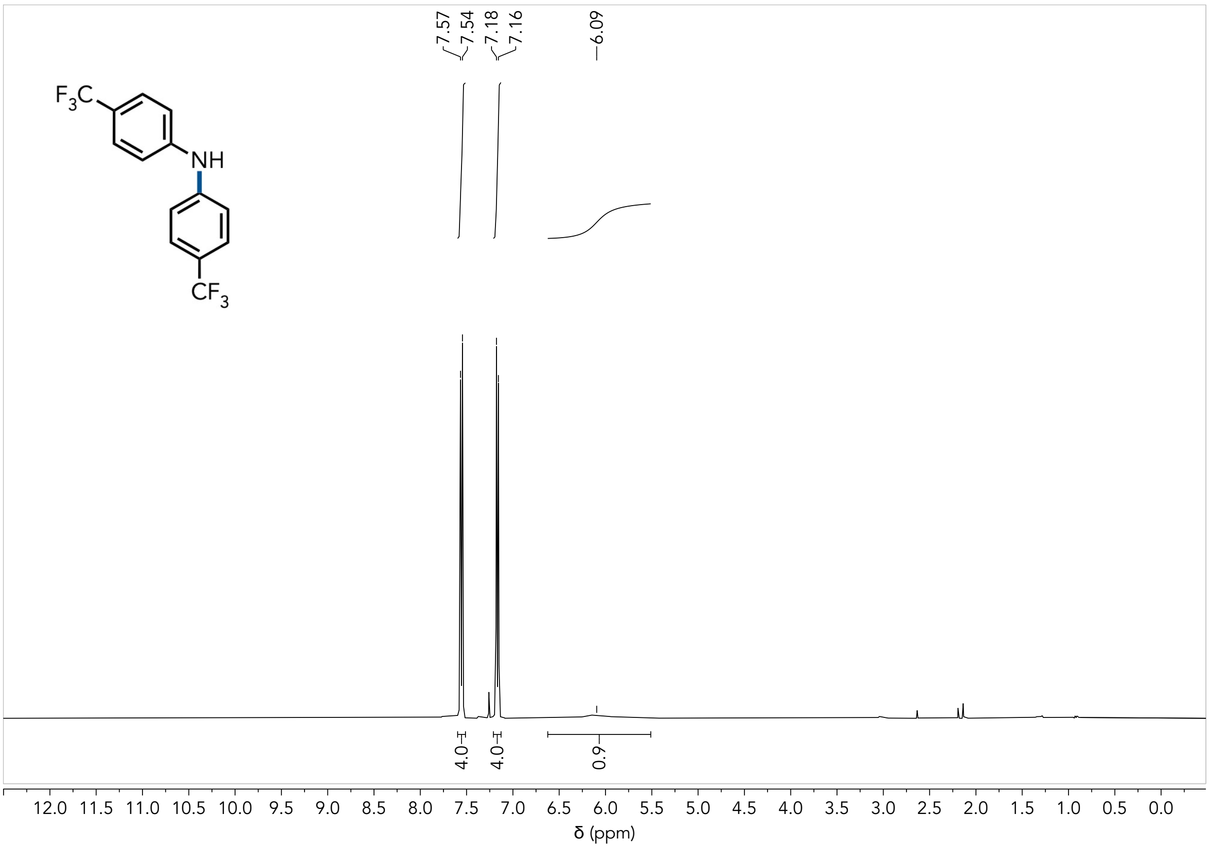

7.57
7.54
7.18
7.16
6.09
F_3C
NH
CF_3
4.0
4.0
0.9
12.0 11.5 11.0 10.5 10.0 9.5 9.0 8.5 8.0 7.5 7.0 6.5 6.0 5.5 5.0 4.5 4.0 3.5 3.0 2.5 2.0 1.5 1.0 0.5 0.0
δ (ppm)

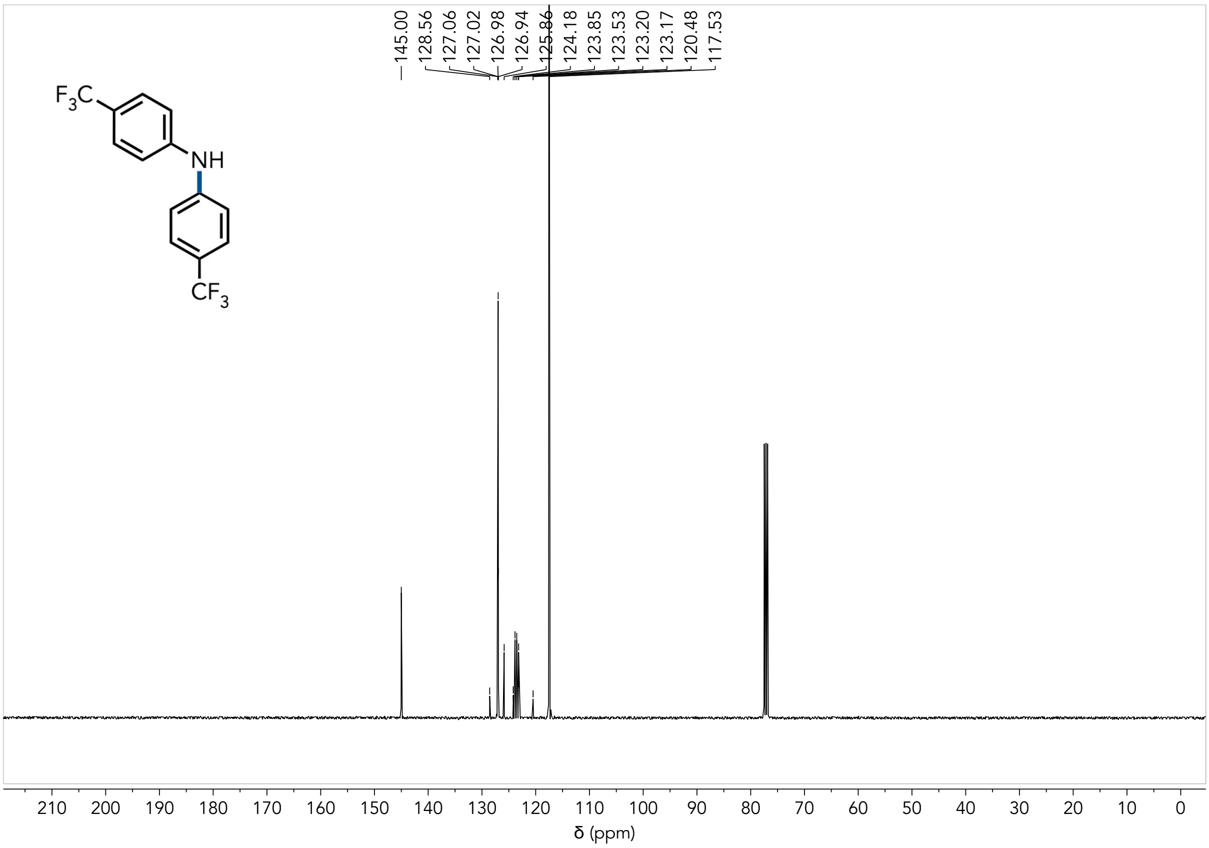

145.00
128.56
127.06
127.02
126.98
126.94
125.86
124.18
123.85
123.53
123.20
123.17
120.48
117.53
F_3C
NH
CF_3
210 200 190 180 170 160 150 140 130 120 110 100 90 80 70 60 50 40 30 20 10 0
δ (ppm)

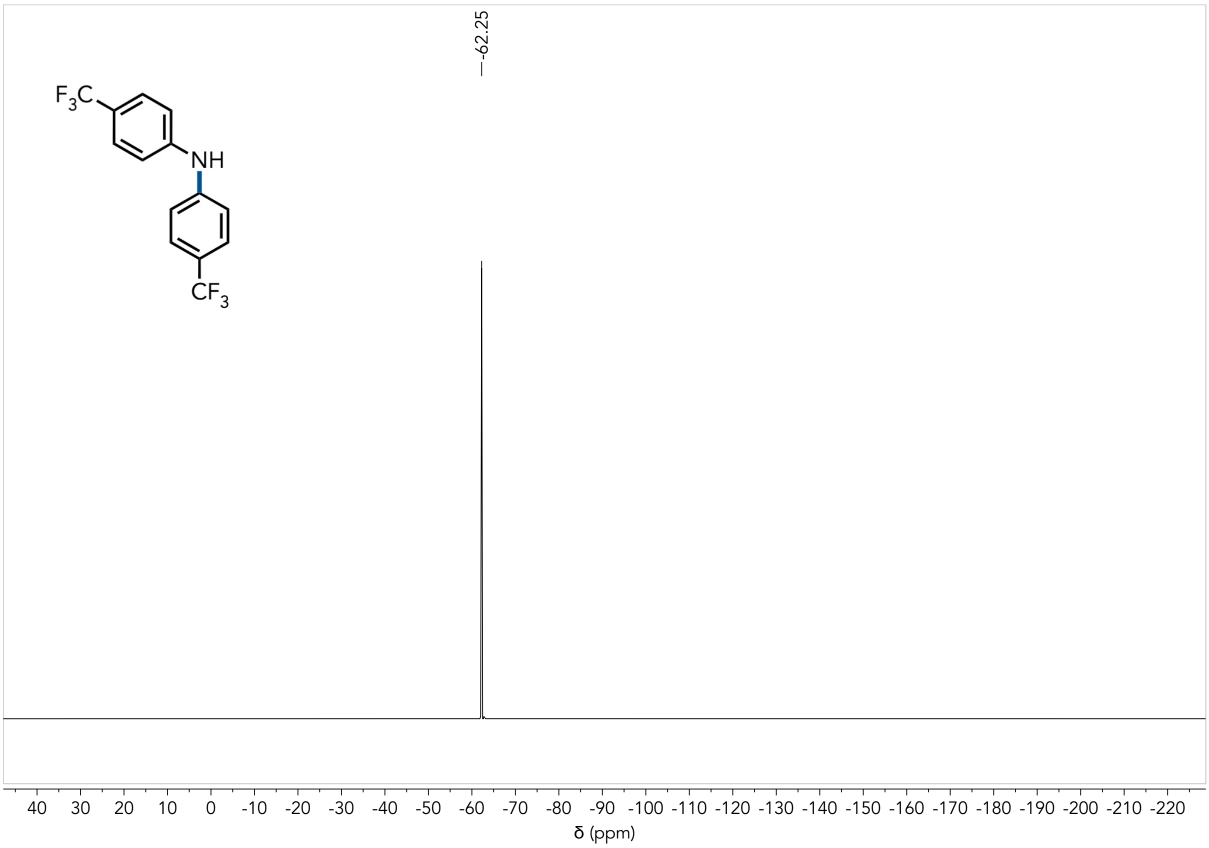

-62.25
F3C
NH
CF3
40 30 20 10 0 -10 -20 -30 -40 -50 -60 -70 -80 -90 -100 -110 -120 -130 -140 -150 -160 -170 -180 -190 -200 -210 -220
δ (ppm)

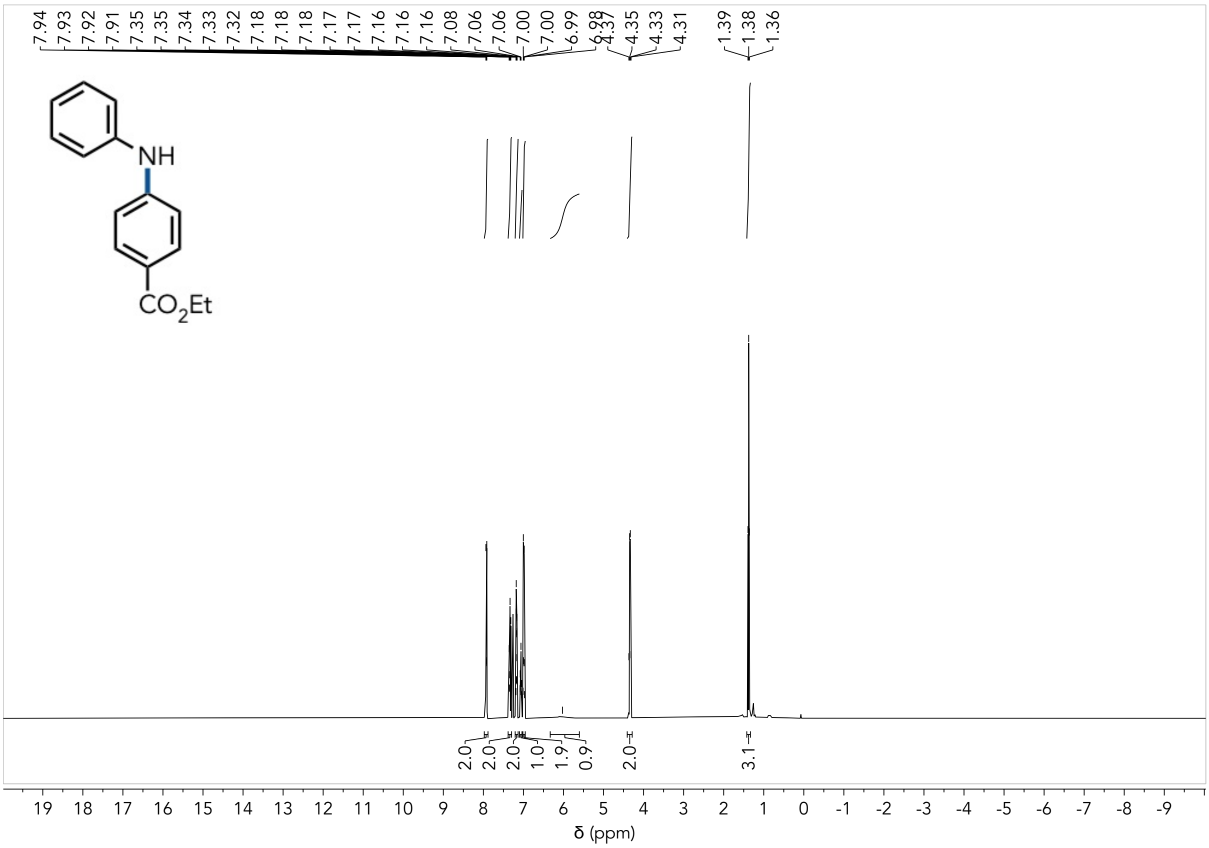

7.94 7.93 7.92 7.91 7.35 7.35 7.34 7.33 7.32 7.18 7.18 7.18 7.17 7.17 7.16 7.16 7.16 7.08 7.06 7.06 7.00 7.00 6.99 6.98 4.37 4.35 4.33 4.31 1.39 1.38 1.36
NH
CO2Et
2.0 2.0 2.0 1.0 1.9 0.9 2.0 3.1
19 18 17 16 15 14 13 12 11 10 9 8 7 6 5 4 3 2 1 0 -1 -2 -3 -4 -5 -6 -7 -8 -9
δ (ppm)

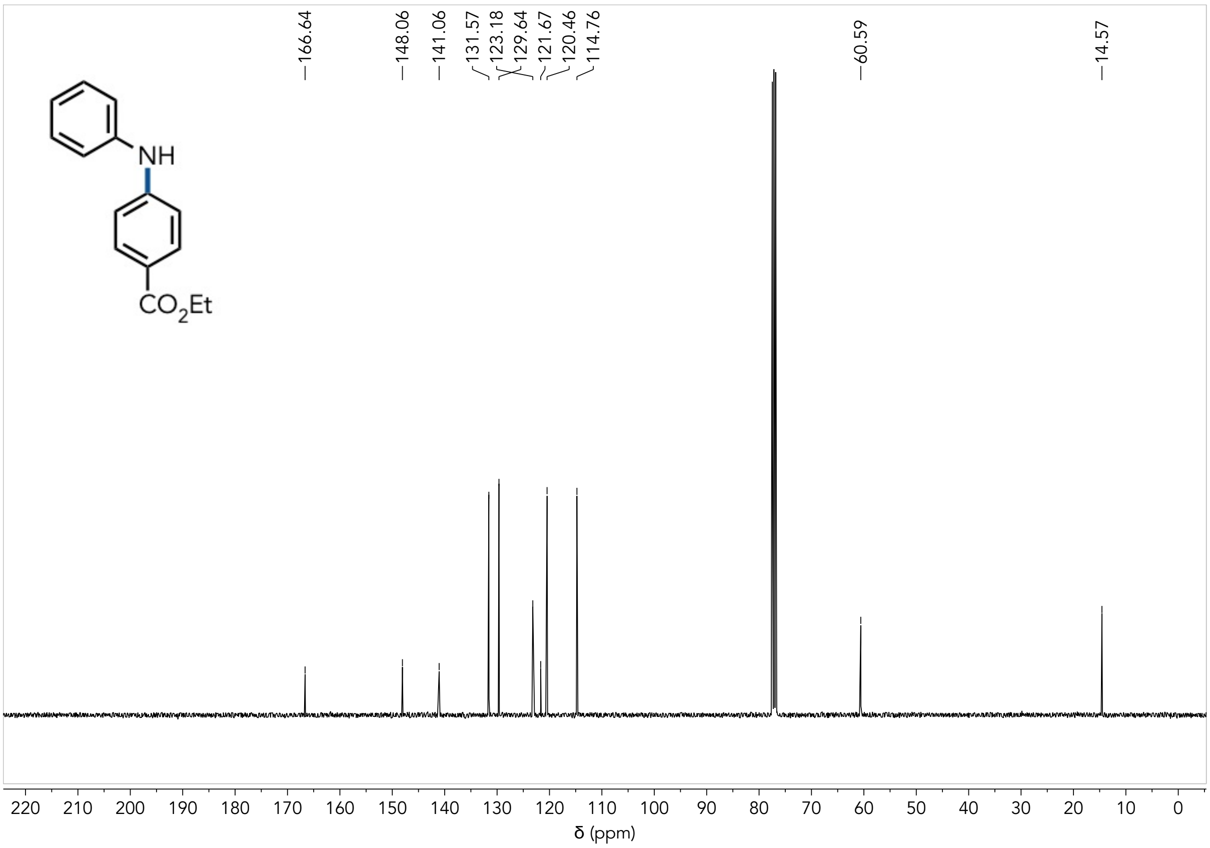
NH
CO2Et
166.64
148.06
141.06
131.57
123.18
129.64
121.67
120.46
114.76
60.59
14.57
220 210 200 190 180 170 160 150 140 130 120 110 100 90 80 70 60 50 40 30 20 10 0
δ (ppm)

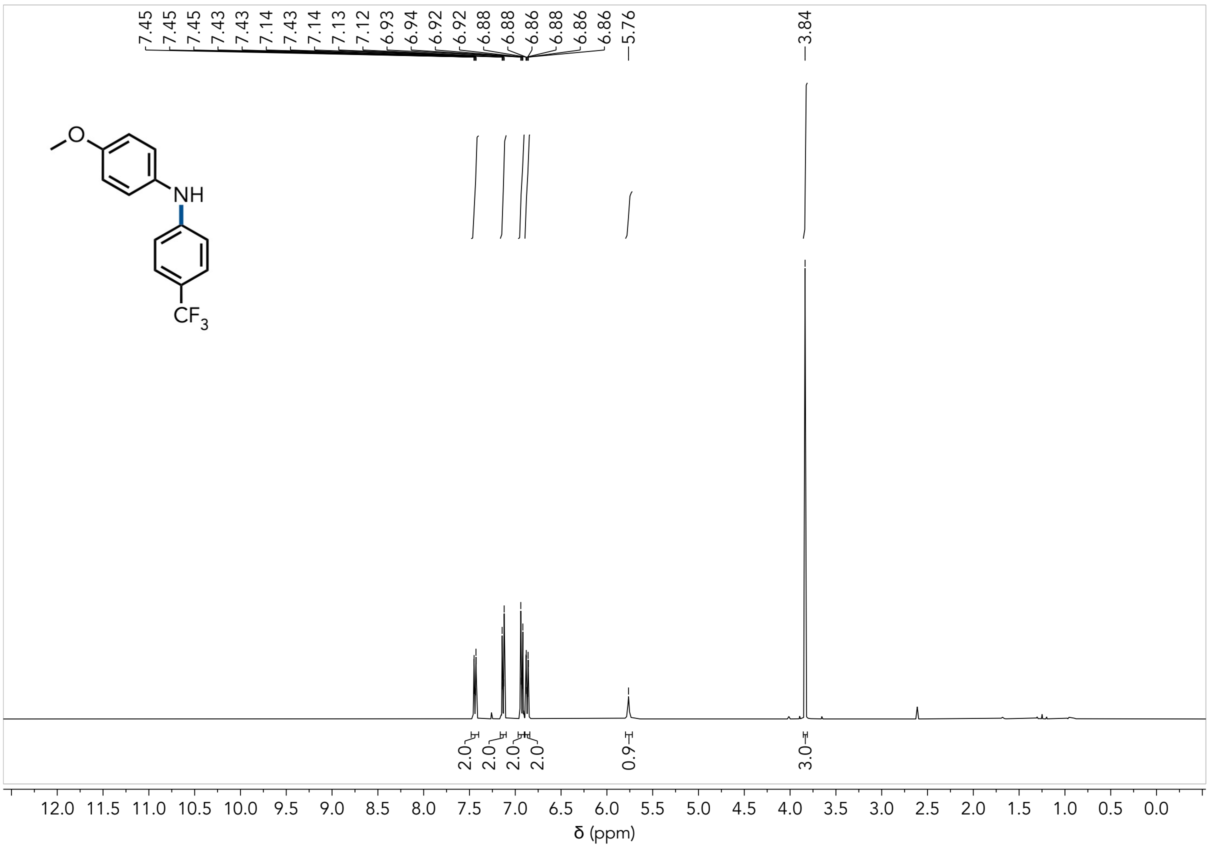
O
NH
CF3
7.45 7.45 7.45 7.43 7.43 7.14 7.43 7.14 7.13 7.12 6.93 6.94 6.92 6.88 6.88 6.86 6.88 6.86 6.86
5.76
3.84
2.0 2.0 2.0 2.0
0.9
3.0
12.0 11.5 11.0 10.5 10.0 9.5 9.0 8.5 8.0 7.5 7.0 6.5 6.0 5.5 5.0 4.5 4.0 3.5 3.0 2.5 2.0 1.5 1.0 0.5 0.0
δ (ppm)

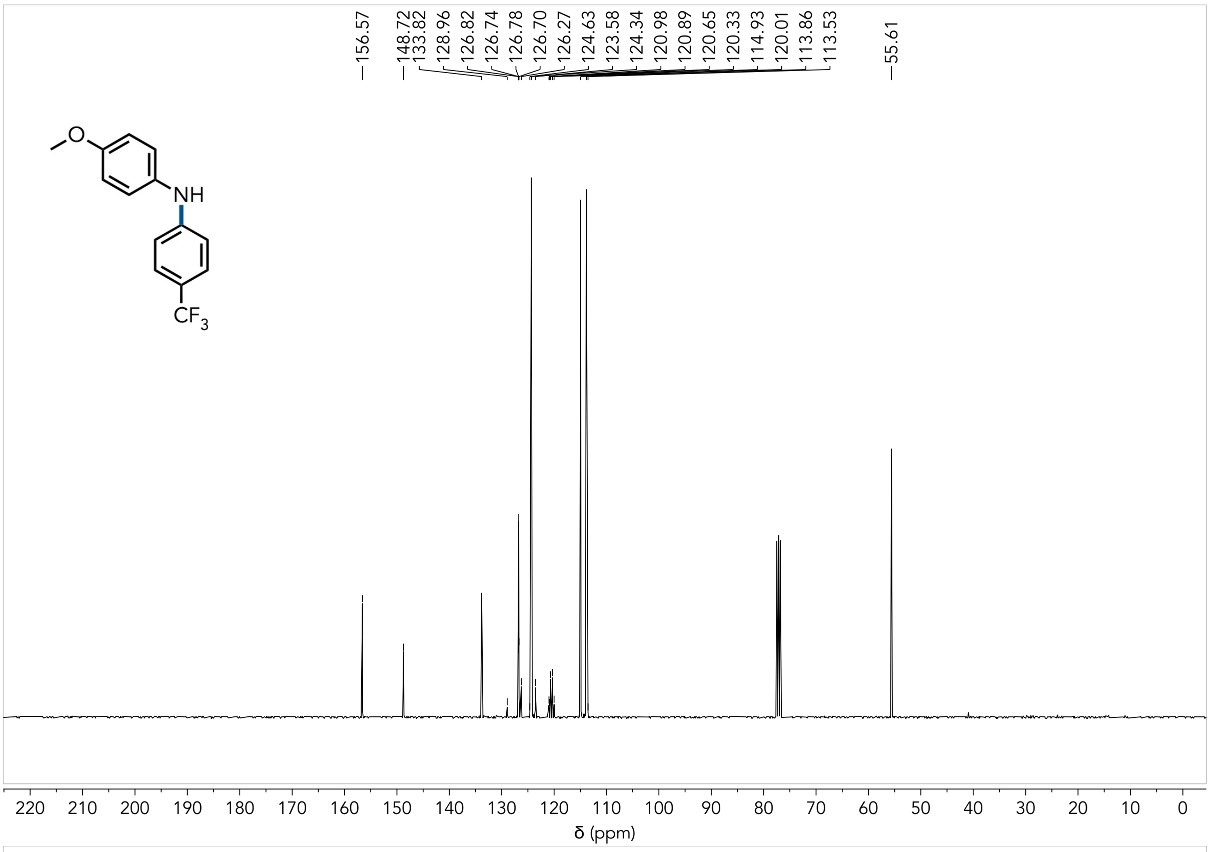
156.57
148.72
133.82
128.96
126.82
126.74
126.78
126.70
126.27
124.63
123.58
124.34
120.98
120.89
120.65
120.33
114.93
120.01
113.86
113.53
55.61
O
NH
CF3
220 210 200 190 180 170 160 150 140 130 120 110 100 90 80 70 60 50 40 30 20 10 0
δ (ppm)

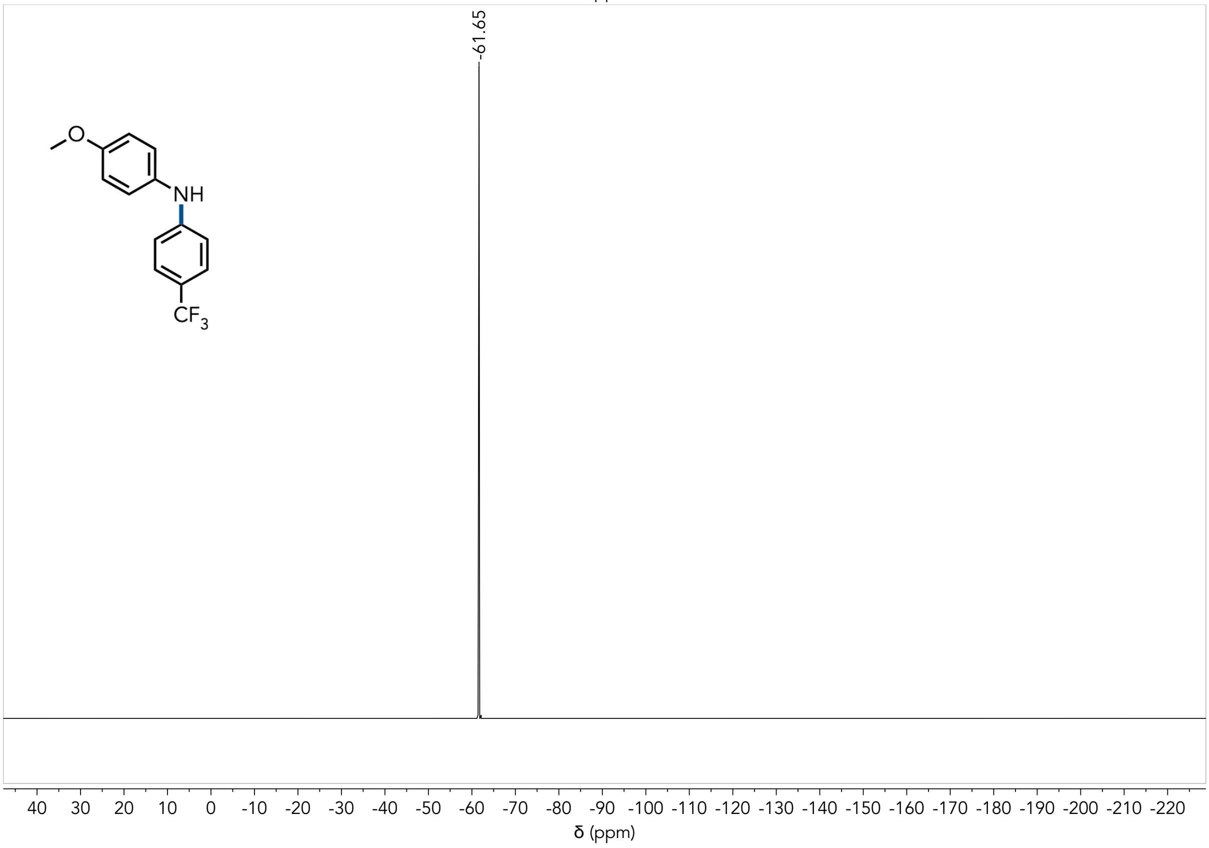
-61.65
O
NH
CF3
40 30 20 10 0 -10 -20 -30 -40 -50 -60 -70 -80 -90 -100 -110 -120 -130 -140 -150 -160 -170 -180 -190 -200 -210 -220
δ (ppm)

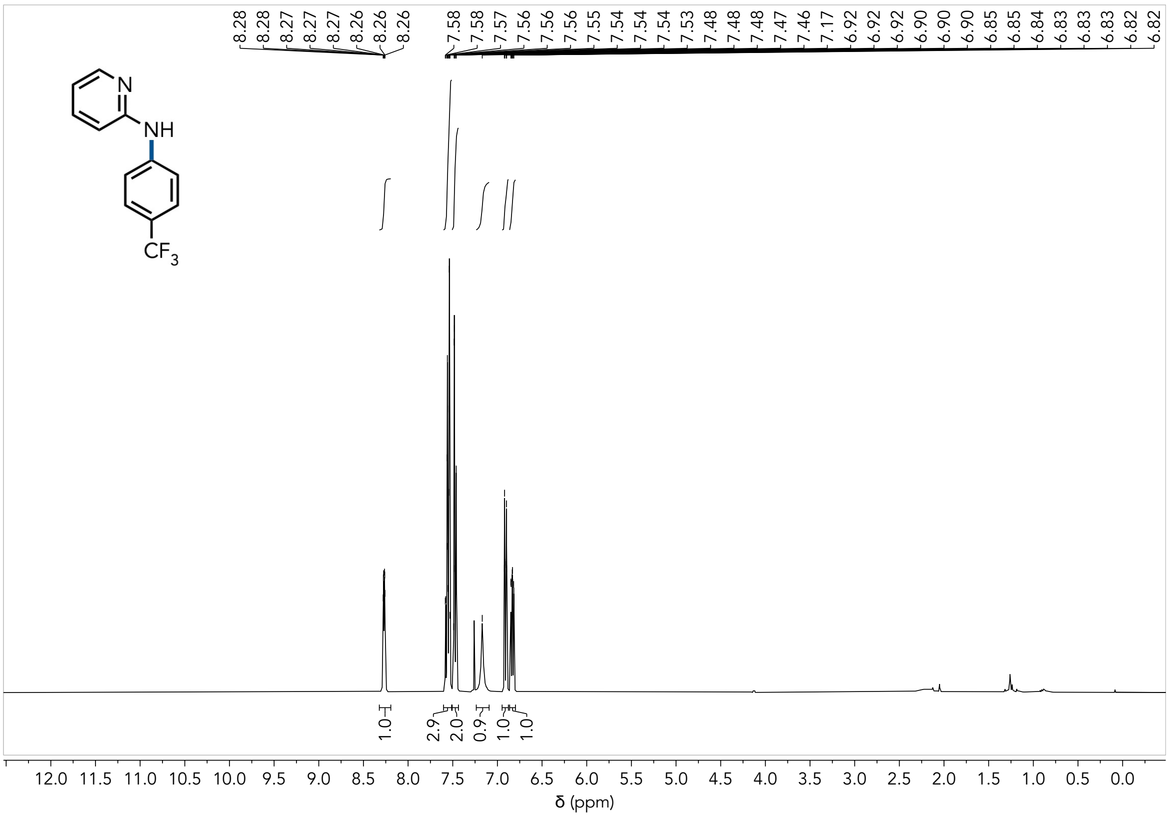
8.28
8.28
8.27
8.27
8.26
8.26
8.26
7.58
7.58
7.57
7.56
7.56
7.55
7.54
7.54
7.54
7.53
7.48
7.48
7.48
7.47
7.46
7.17
6.92
6.92
6.92
6.90
6.90
6.90
6.85
6.85
6.84
6.83
6.83
6.83
6.82
6.82
N
NH
CF3
1.0
2.9
2.0
0.9
1.0
1.0
12.0 11.5 11.0 10.5 10.0 9.5 9.0 8.5 8.0 7.5 7.0 6.5 6.0 5.5 5.0 4.5 4.0 3.5 3.0 2.5 2.0 1.5 1.0 0.5 0.0
δ (ppm)

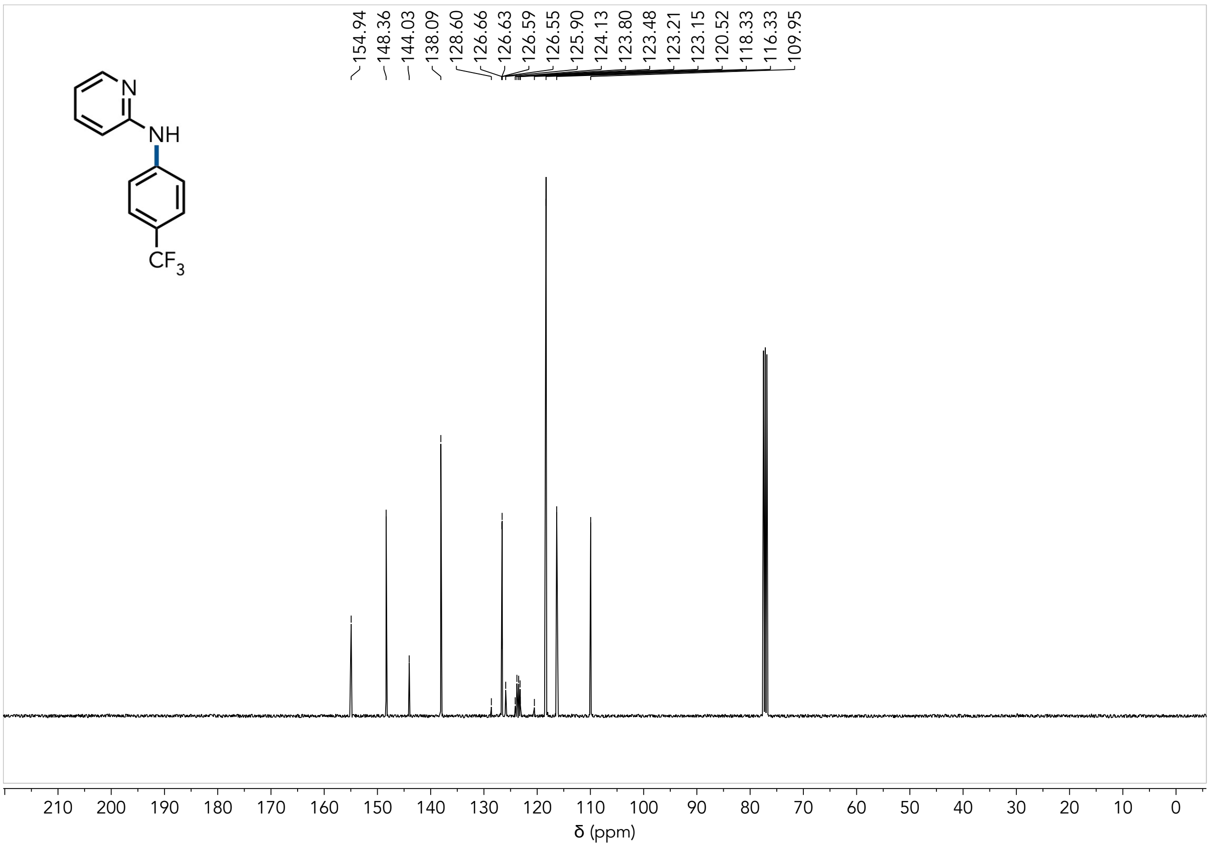
154.94
148.36
144.03
138.09
128.60
126.66
126.63
126.59
126.55
125.90
124.13
123.80
123.48
123.21
123.15
120.52
118.33
116.33
109.95
N
NH
CF3
210 200 190 180 170 160 150 140 130 120 110 100 90 80 70 60 50 40 30 20 10 0
δ (ppm)

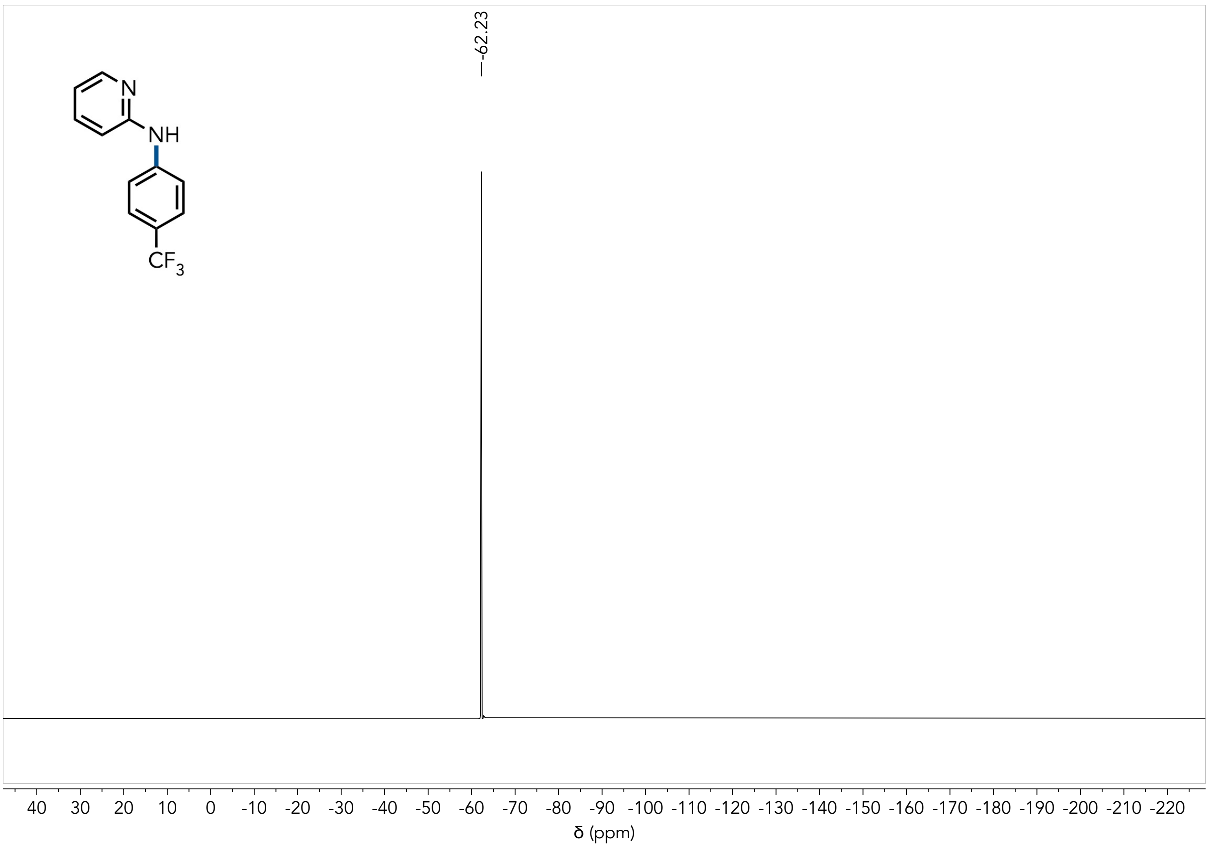
-62.23
N
NH
CF_3
40 30 20 10 0 -10 -20 -30 -40 -50 -60 -70 -80 -90 -100 -110 -120 -130 -140 -150 -160 -170 -180 -190 -200 -210 -220
δ (ppm)

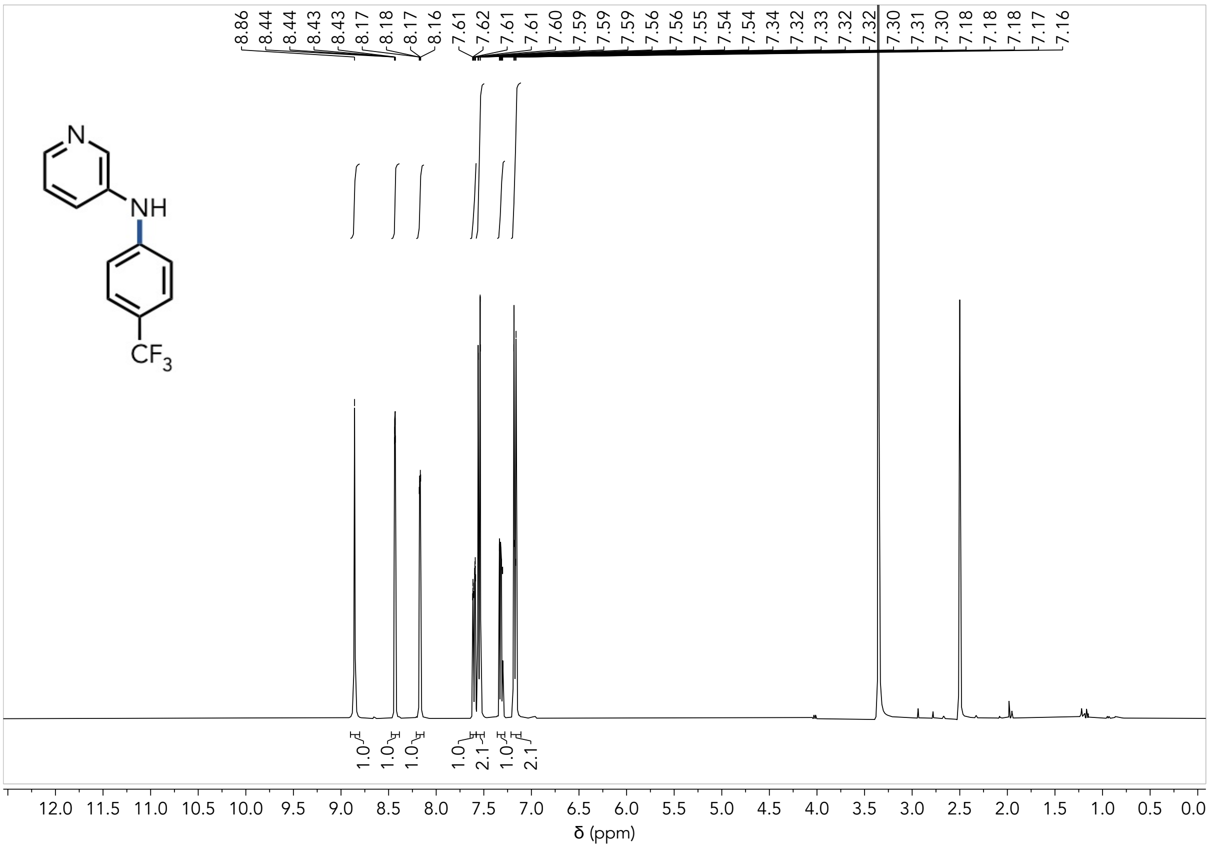
8.86 8.44 8.44 8.43 8.43 8.17 8.18 8.17 8.16 7.61 7.62 7.61 7.61 7.60 7.59 7.59 7.59 7.56 7.56 7.55 7.54 7.54 7.34 7.32 7.33 7.32 7.32 7.30 7.31 7.30 7.18 7.18 7.18 7.17 7.16
N
NH
CF_3
1.0 1.0 1.0 1.0 2.1 1.0 2.1
12.0 11.5 11.0 10.5 10.0 9.5 9.0 8.5 8.0 7.5 7.0 6.5 6.0 5.5 5.0 4.5 4.0 3.5 3.0 2.5 2.0 1.5 1.0 0.5 0.0
δ (ppm)

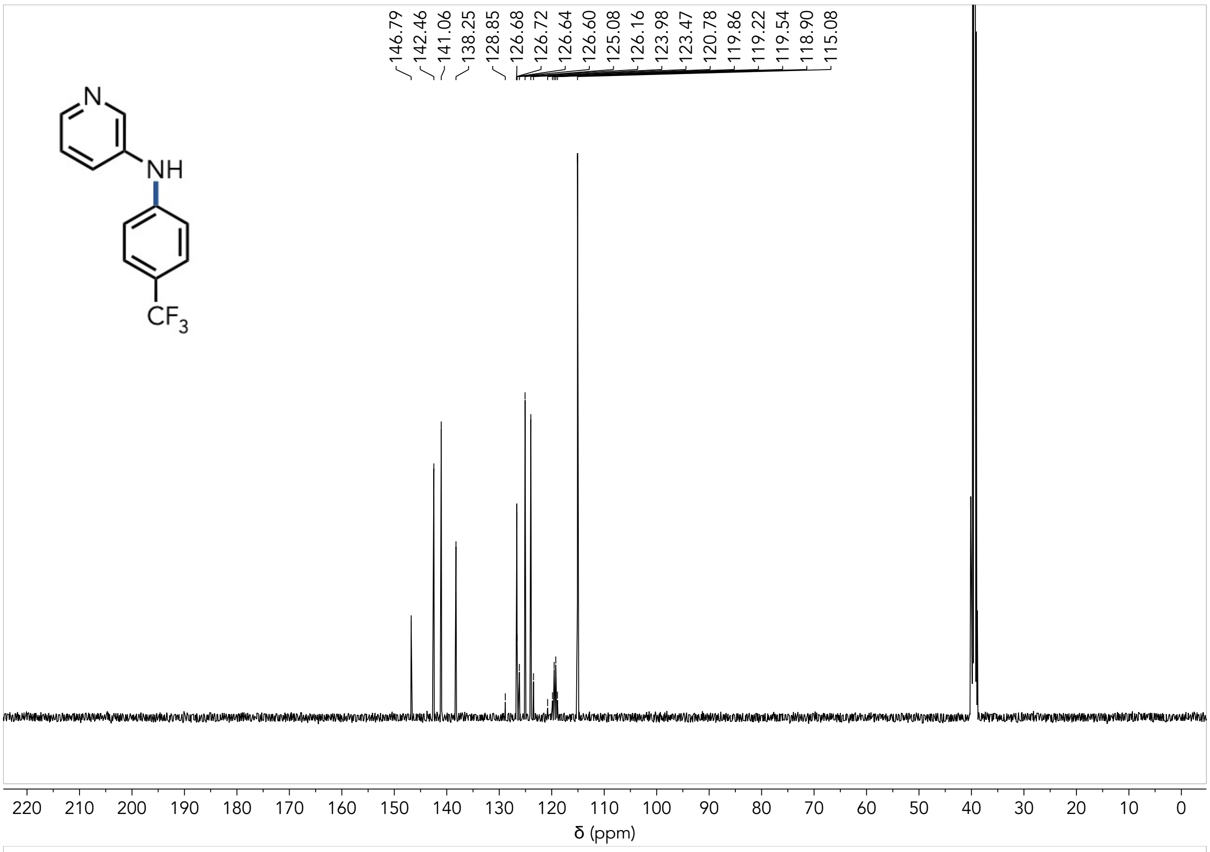
146.79
142.46
141.06
138.25
128.85
126.68
126.72
126.64
126.60
125.08
126.16
123.98
123.47
120.78
119.86
119.22
119.54
118.90
115.08
N
NH
CF3
220 210 200 190 180 170 160 150 140 130 120 110 100 90 80 70 60 50 40 30 20 10 0
δ (ppm)

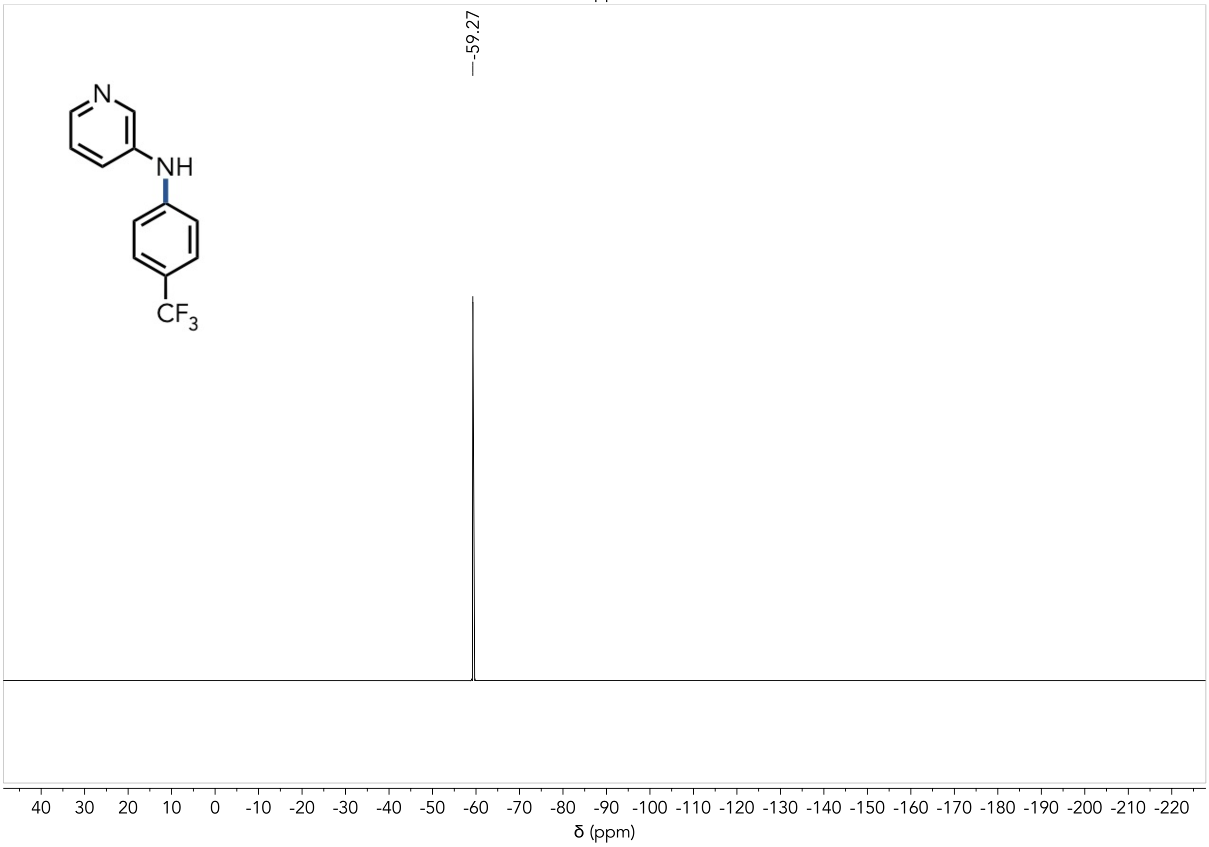
-59.27
N
NH
CF3
40 30 20 10 0 -10 -20 -30 -40 -50 -60 -70 -80 -90 -100 -110 -120 -130 -140 -150 -160 -170 -180 -190 -200 -210 -220
δ (ppm)

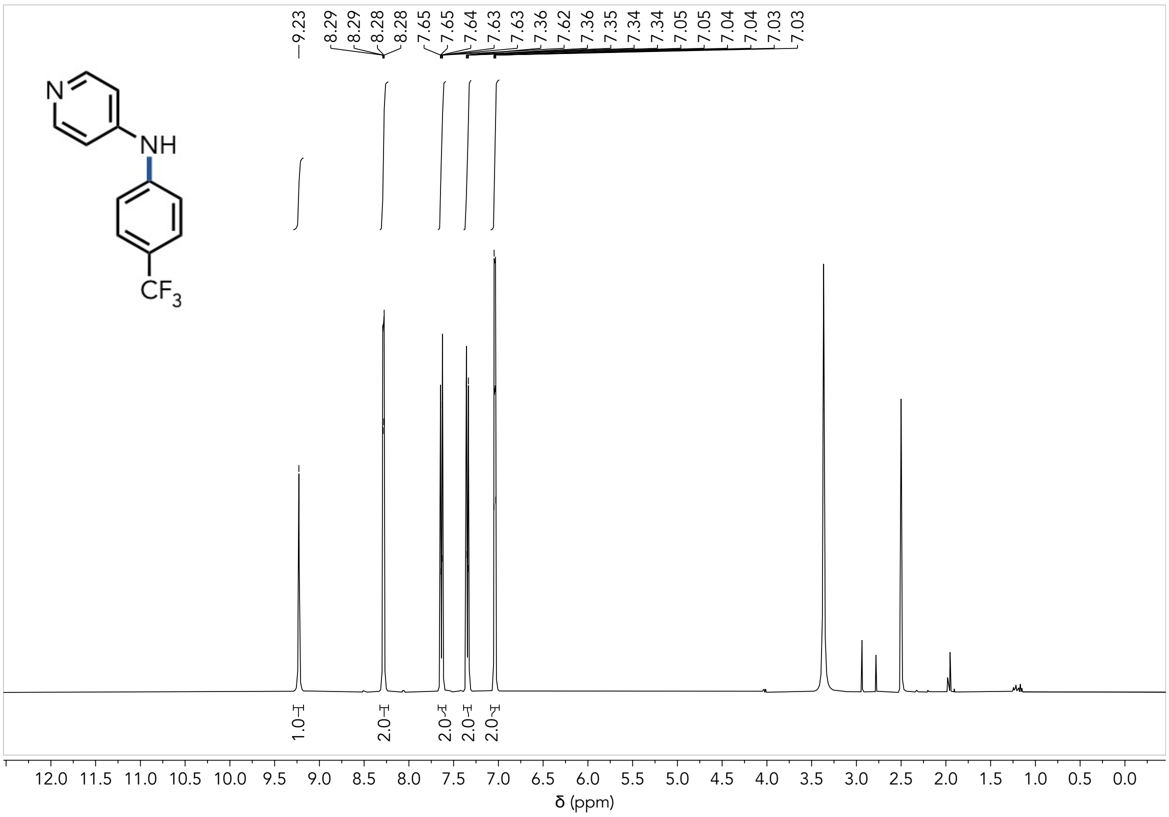
9.23
8.29
8.29
8.28
8.28
7.65
7.65
7.64
7.63
7.63
7.36
7.62
7.36
7.35
7.34
7.34
7.05
7.05
7.04
7.04
7.03
7.03
N
NH
CF_3
1.0
2.0
2.0
2.0
2.0
12.0 11.5 11.0 10.5 10.0 9.5 9.0 8.5 8.0 7.5 7.0 6.5 6.0 5.5 5.0 4.5 4.0 3.5 3.0 2.5 2.0 1.5 1.0 0.5 0.0
δ (ppm)

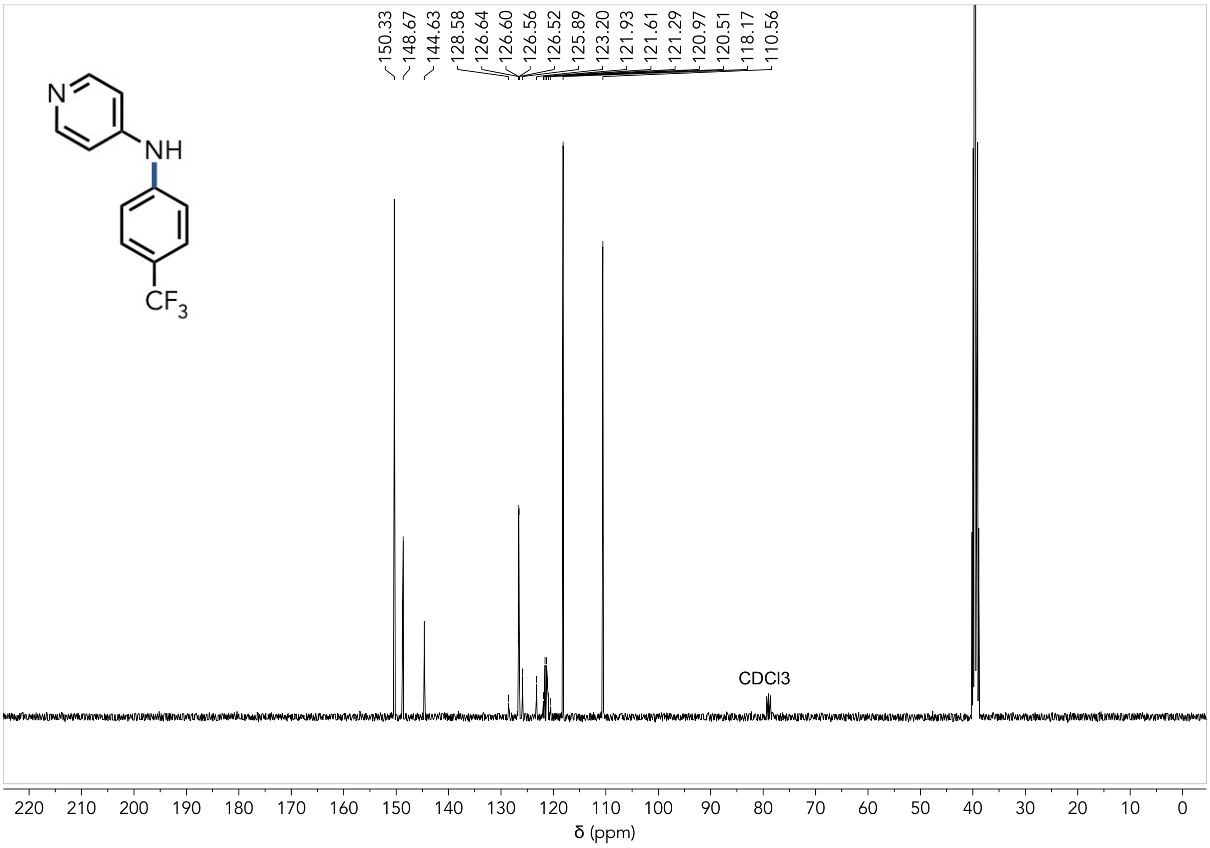
150.33
148.67
144.63
128.58
126.64
126.60
126.56
126.52
125.89
123.20
121.93
121.61
121.29
120.97
120.51
118.17
110.56
N
NH
CF_3
CDCl3
220 210 200 190 180 170 160 150 140 130 120 110 100 90 80 70 60 50 40 30 20 10 0
δ (ppm)

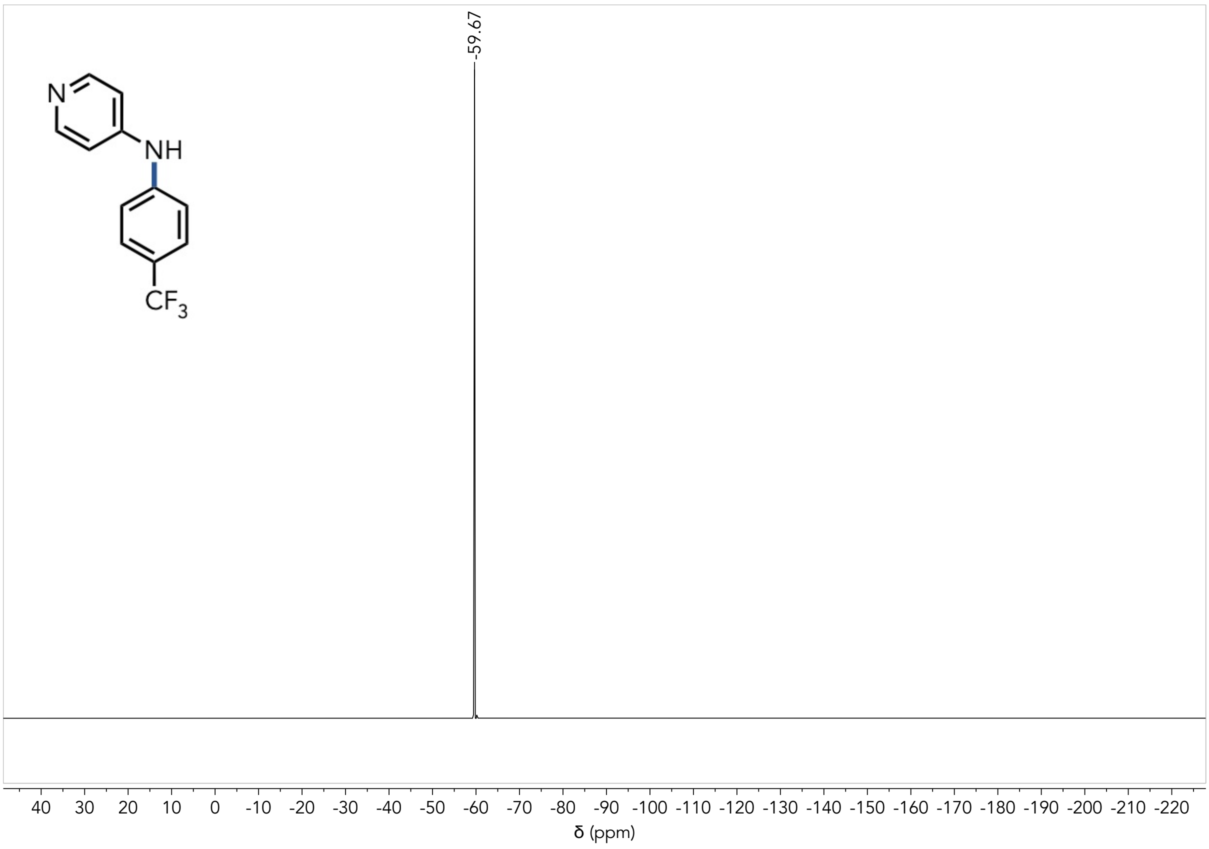

-59.67
N
NH
CF3
40 30 20 10 0 -10 -20 -30 -40 -50 -60 -70 -80 -90 -100 -110 -120 -130 -140 -150 -160 -170 -180 -190 -200 -210 -220
δ (ppm)

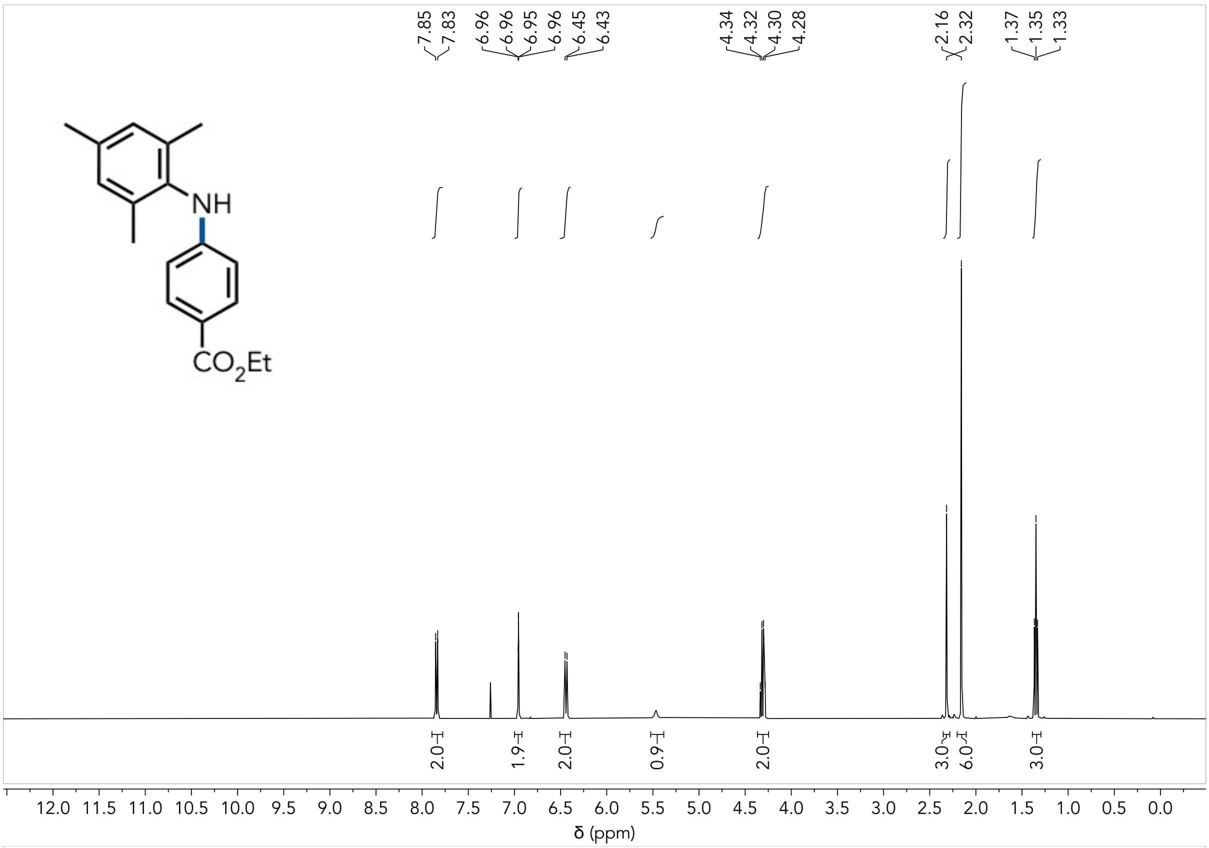
7.85
7.83
6.96
6.96
6.95
6.96
6.45
6.43
4.34
4.32
4.30
4.28
2.16
2.32
1.37
1.35
1.33
NH
CO2Et
2.0
1.9
2.0
0.9
2.0
3.0
6.0
3.0
12.0 11.5 11.0 10.5 10.0 9.5 9.0 8.5 8.0 7.5 7.0 6.5 6.0 5.5 5.0 4.5 4.0 3.5 3.0 2.5 2.0 1.5 1.0 0.5 0.0
δ (ppm)

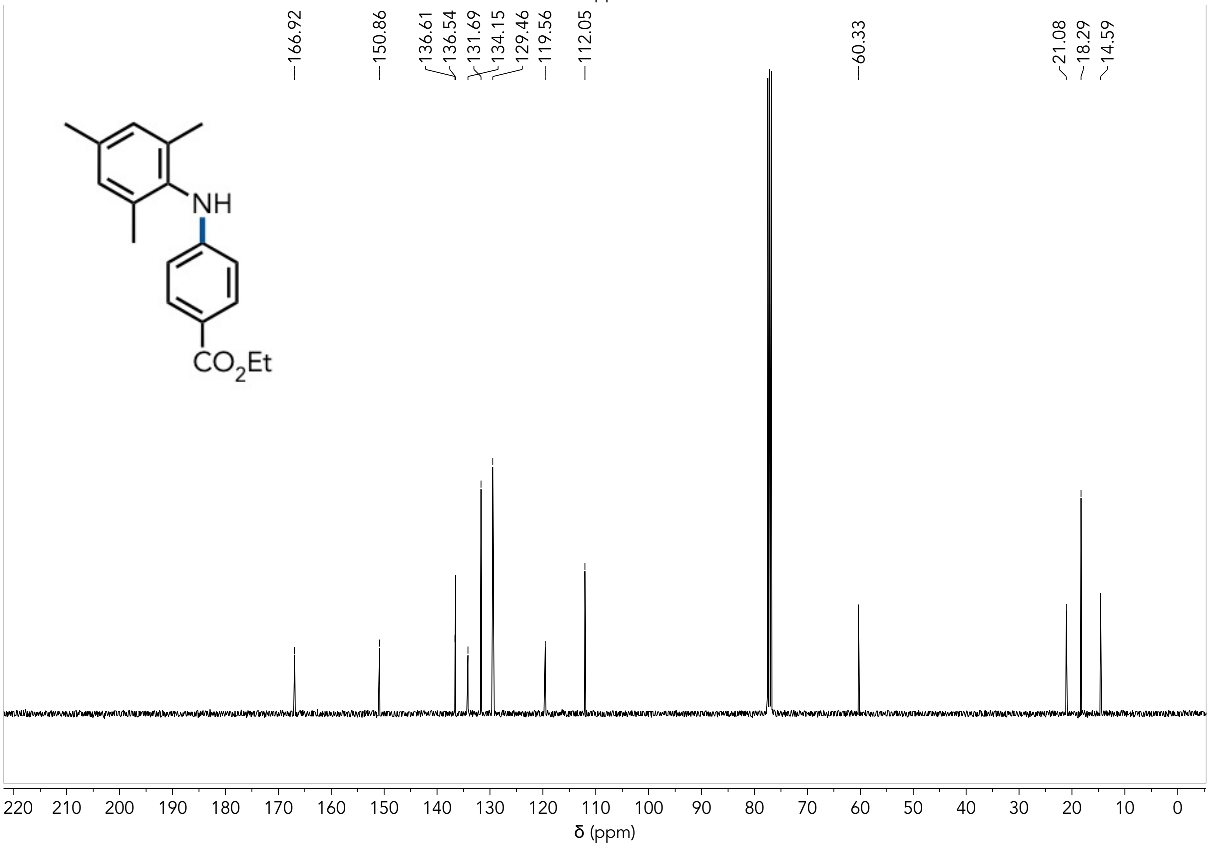
166.92
150.86
136.61
136.54
131.69
134.15
129.46
119.56
112.05
60.33
21.08
18.29
14.59
NH
CO2Et
220 210 200 190 180 170 160 150 140 130 120 110 100 90 80 70 60 50 40 30 20 10 0
δ (ppm)

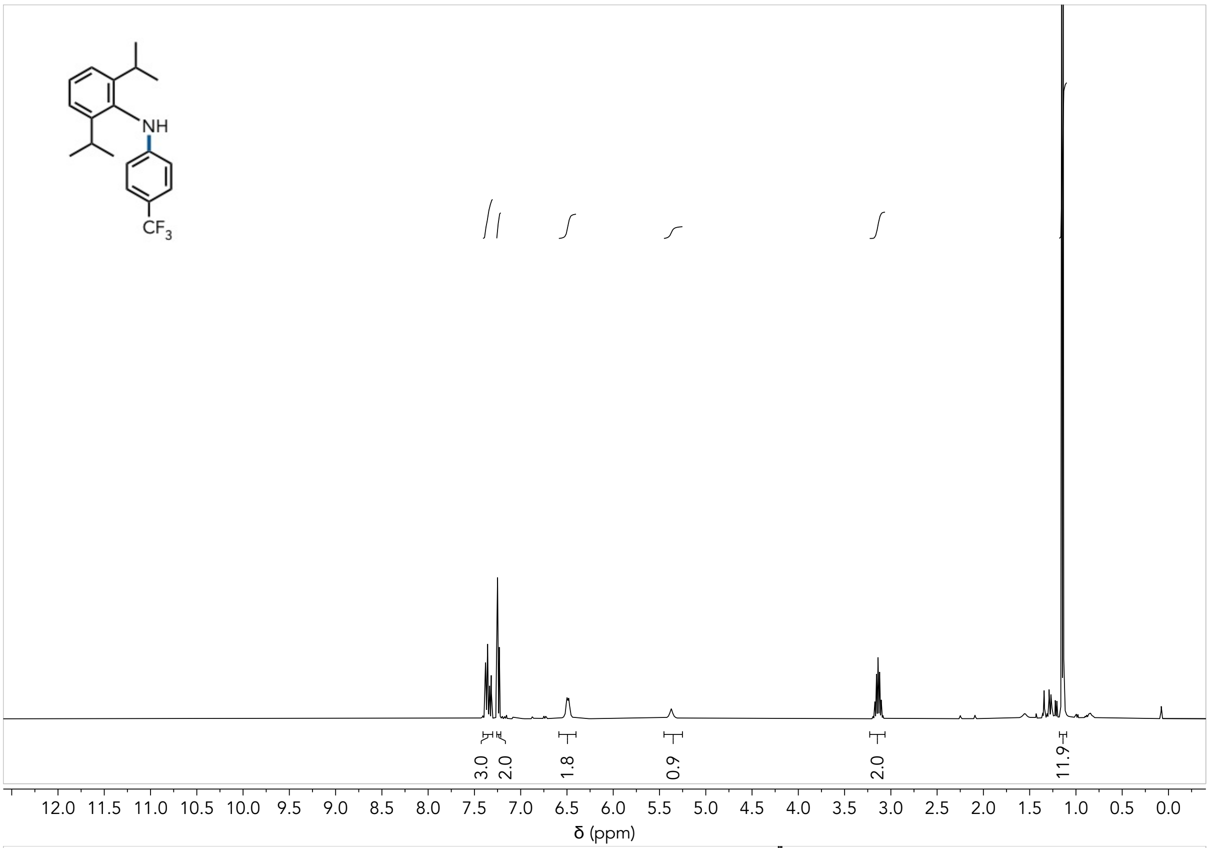
NH
CF3
3.0
2.0
1.8
0.9
2.0
11.9
12.0 11.5 11.0 10.5 10.0 9.5 9.0 8.5 8.0 7.5 7.0 6.5 6.0 5.5 5.0 4.5 4.0 3.5 3.0 2.5 2.0 1.5 1.0 0.5 0.0
δ (ppm)

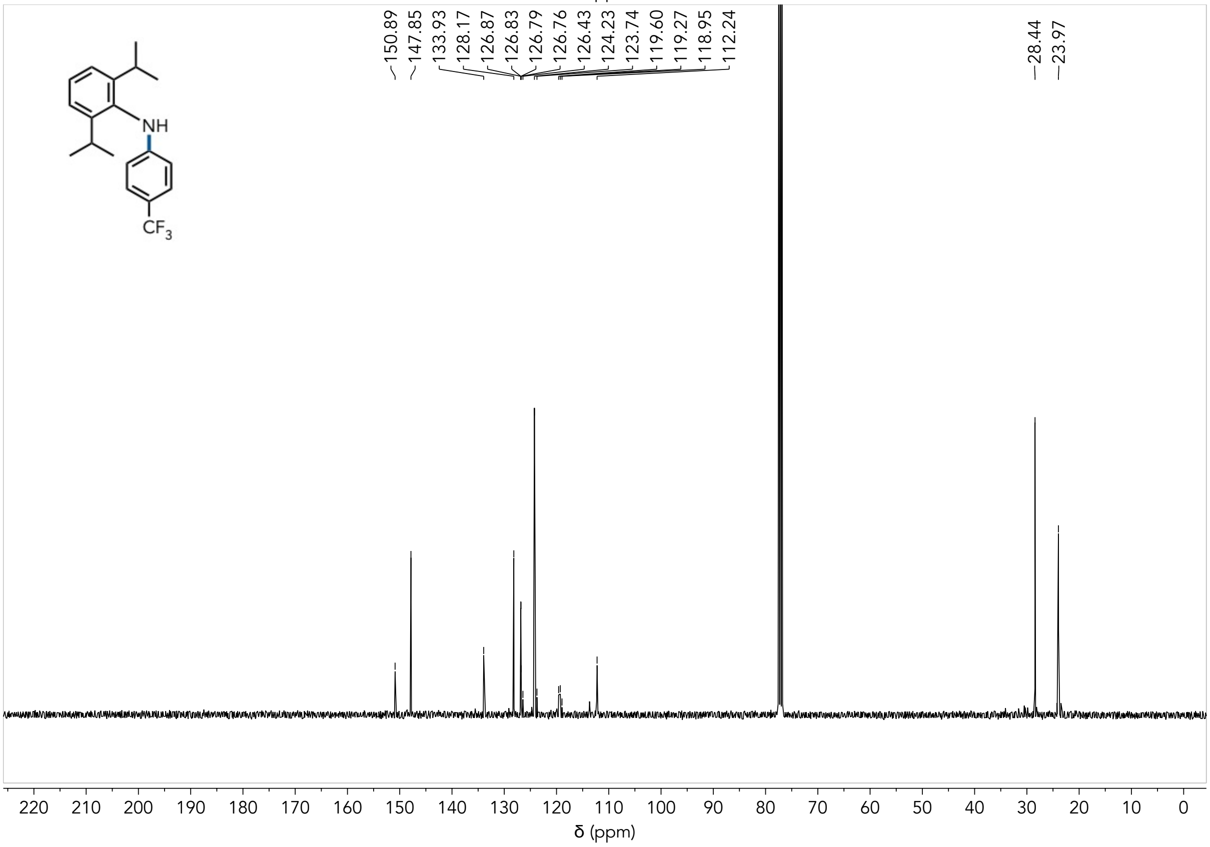
150.89
147.85
133.93
128.17
126.87
126.83
126.79
126.76
126.43
124.23
123.74
119.60
119.27
118.95
112.24
28.44
23.97
NH
CF3
220 210 200 190 180 170 160 150 140 130 120 110 100 90 80 70 60 50 40 30 20 10 0
δ (ppm)

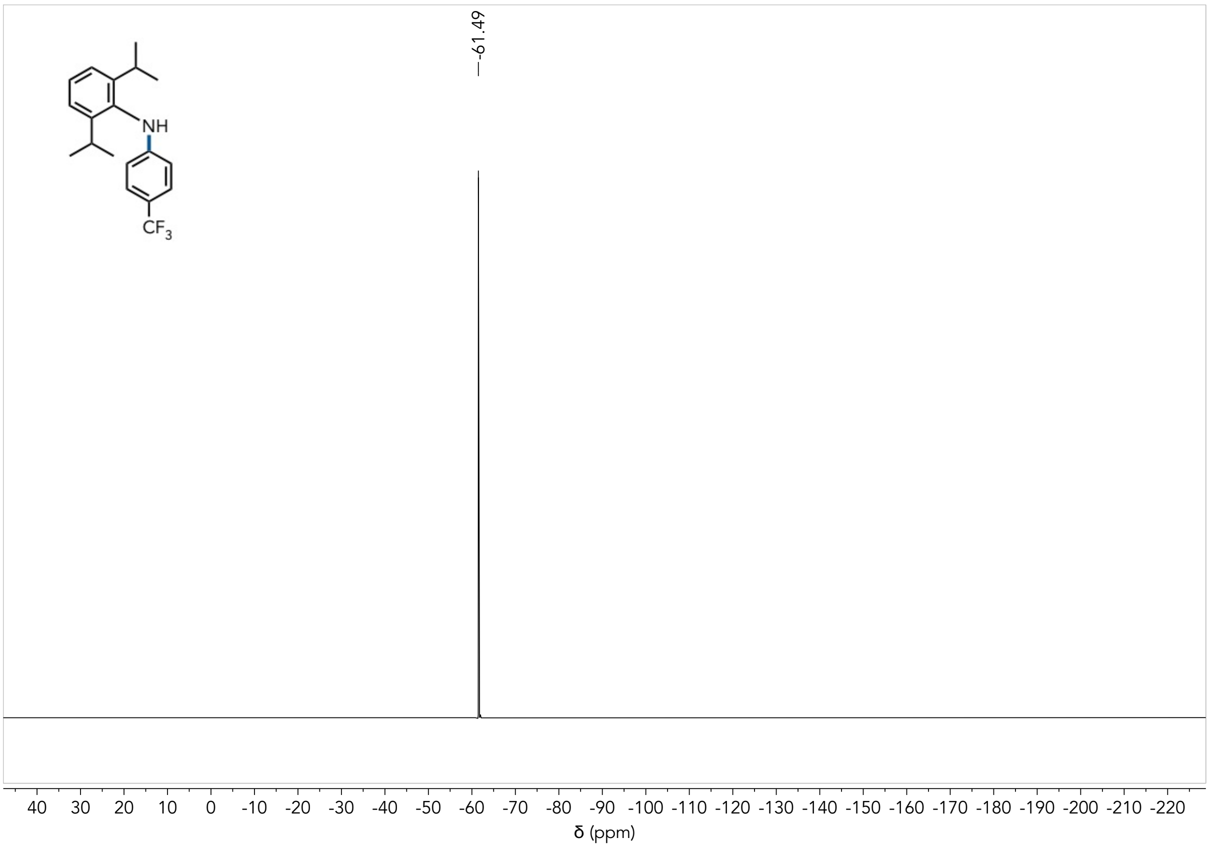

—61.49
NH
CF3
40 30 20 10 0 -10 -20 -30 -40 -50 -60 -70 -80 -90 -100 -110 -120 -130 -140 -150 -160 -170 -180 -190 -200 -210 -220
δ (ppm)

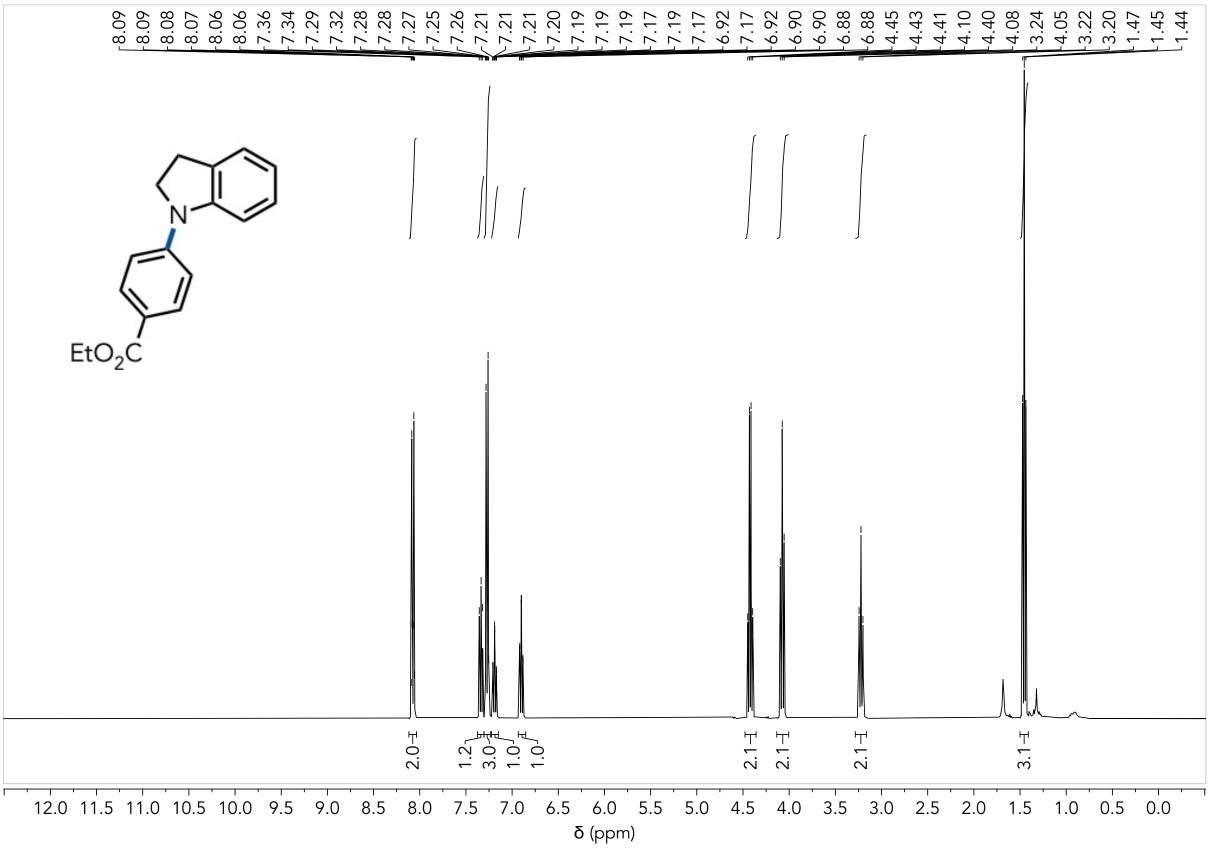
EtO2C
2.0
1.2
3.0
1.0
1.0
2.1
2.1
2.1
3.1
δ (ppm)

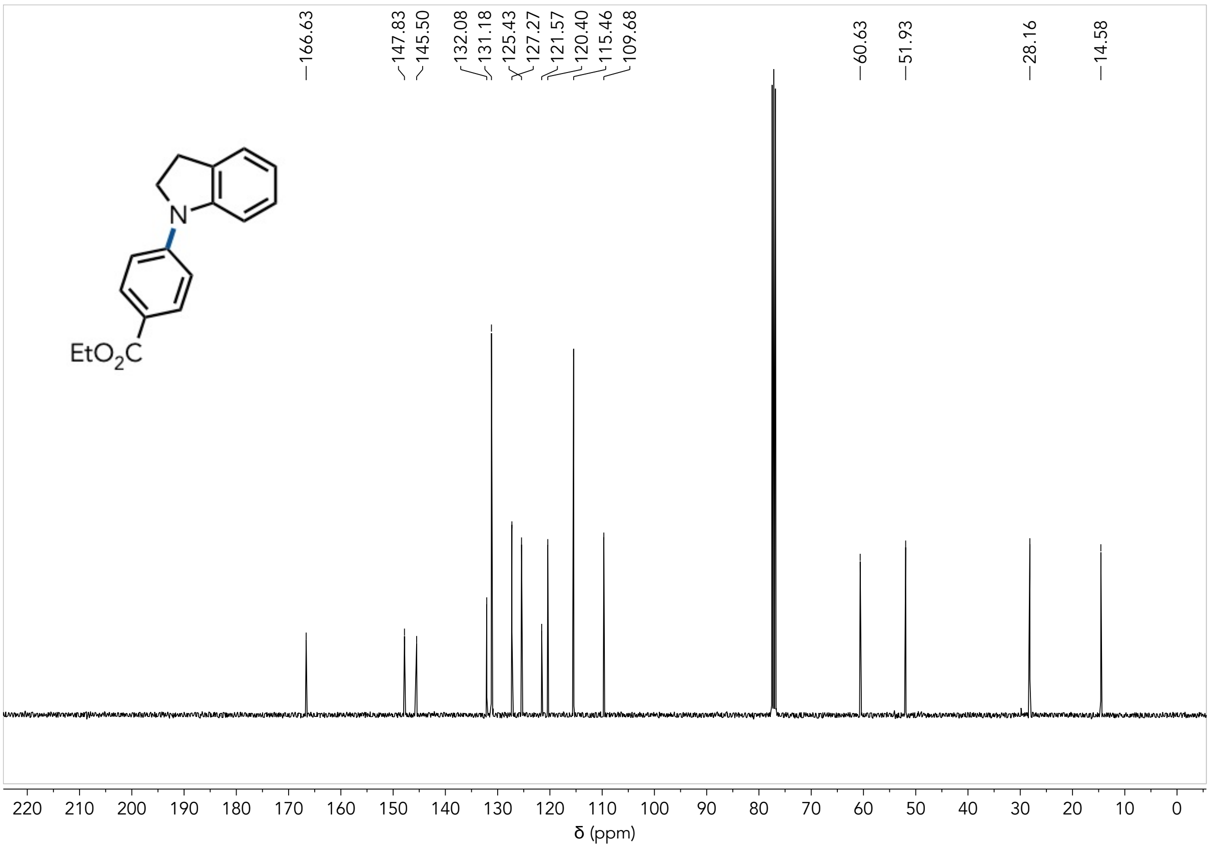
166.63
147.83
145.50
132.08
131.18
125.43
127.27
121.57
120.40
115.46
109.68
60.63
51.93
28.16
14.58
EtO2C
δ (ppm)

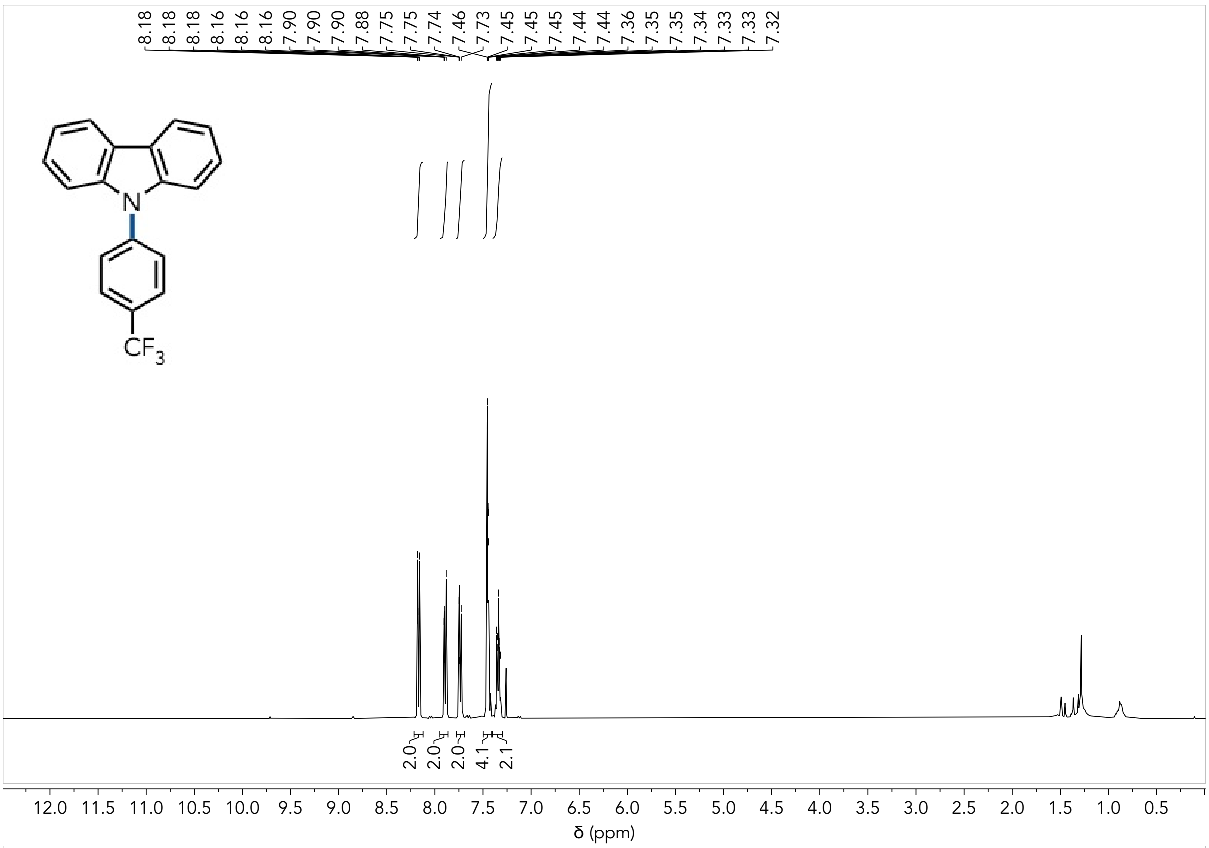
8.18
8.18
8.18
8.16
8.16
8.16
7.90
7.90
7.90
7.88
7.75
7.75
7.74
7.46
7.73
7.45
7.45
7.45
7.44
7.44
7.36
7.35
7.35
7.34
7.33
7.33
7.32
N
CF3
2.0
2.0
2.0
4.1
2.1
12.0 11.5 11.0 10.5 10.0 9.5 9.0 8.5 8.0 7.5 7.0 6.5 6.0 5.5 5.0 4.5 4.0 3.5 3.0 2.5 2.0 1.5 1.0 0.5
δ (ppm)

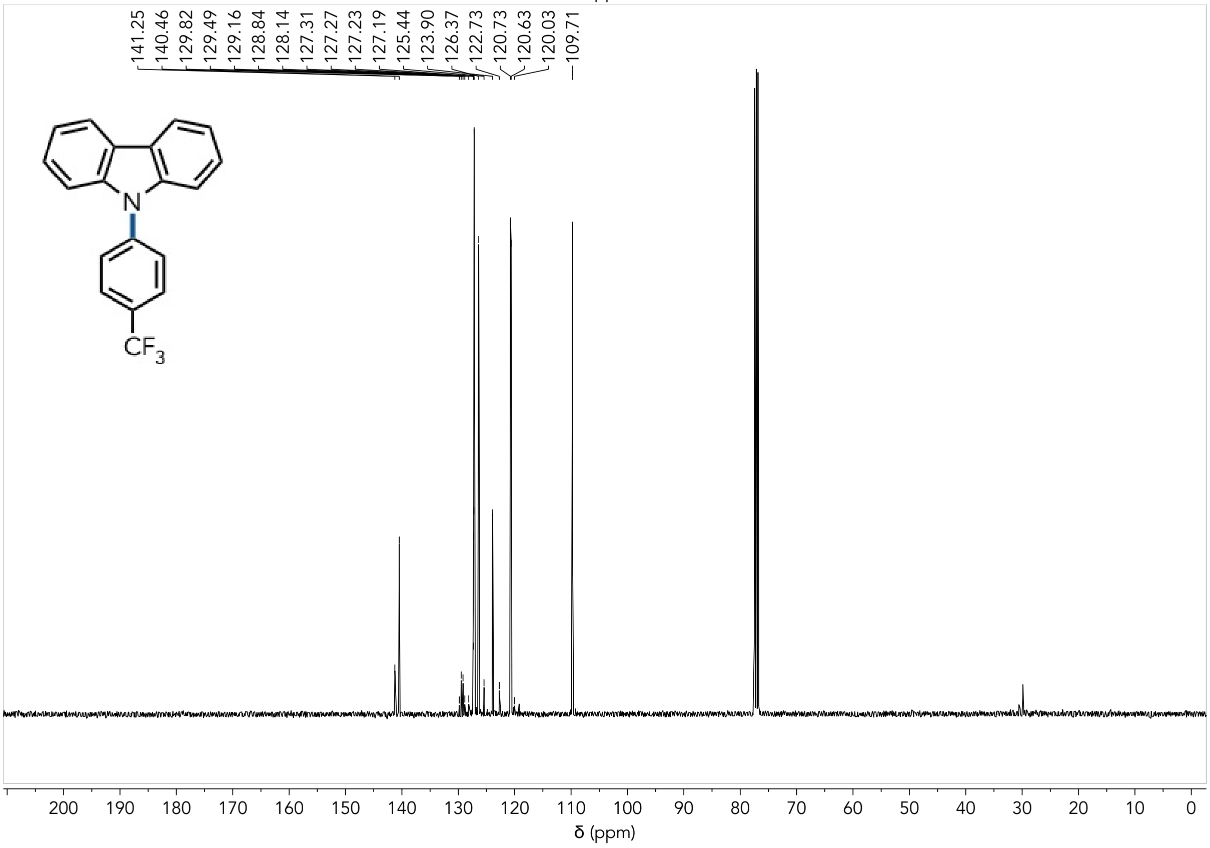
141.25
140.46
129.82
129.49
129.16
128.84
128.14
127.31
127.27
127.23
127.19
125.44
123.90
126.37
122.73
120.73
120.63
120.03
109.71
N
CF3
200 190 180 170 160 150 140 130 120 110 100 90 80 70 60 50 40 30 20 10 0
δ (ppm)

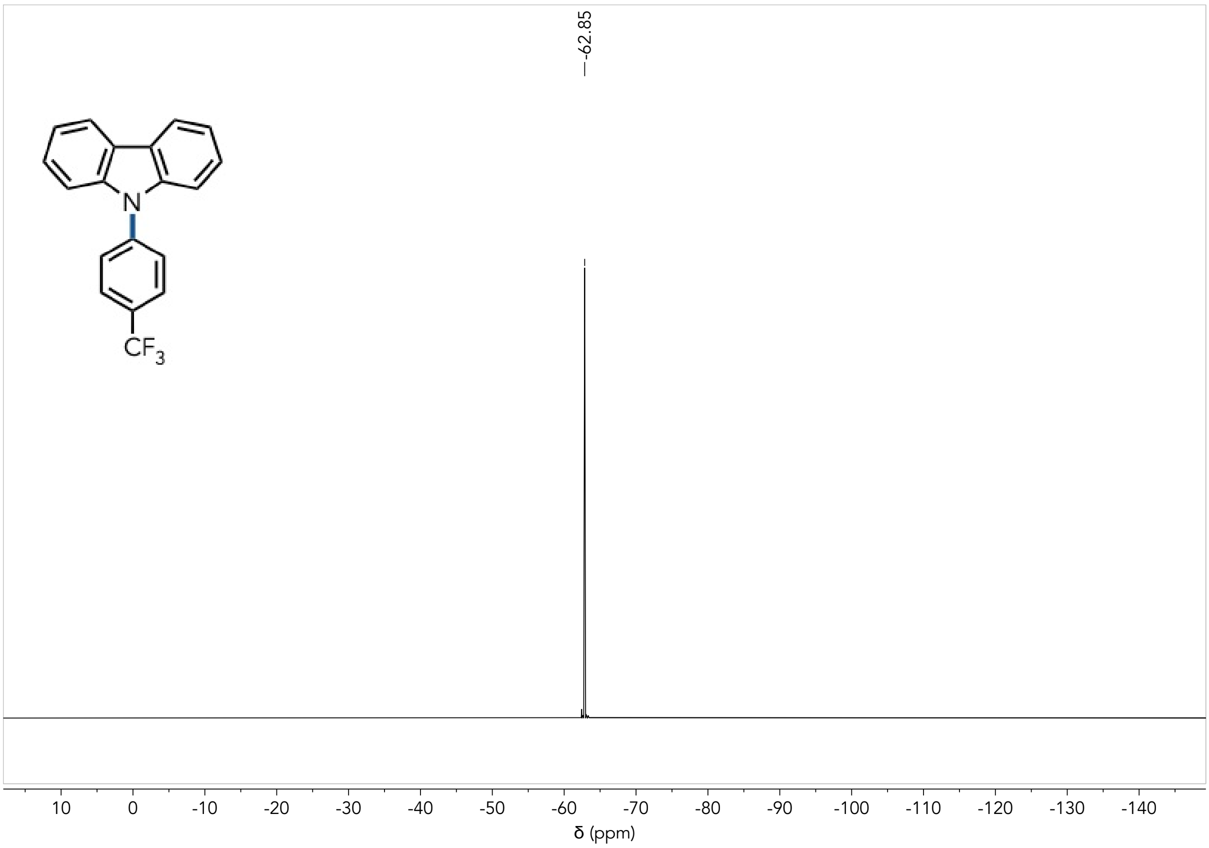

CF3
-62.85
δ (ppm)

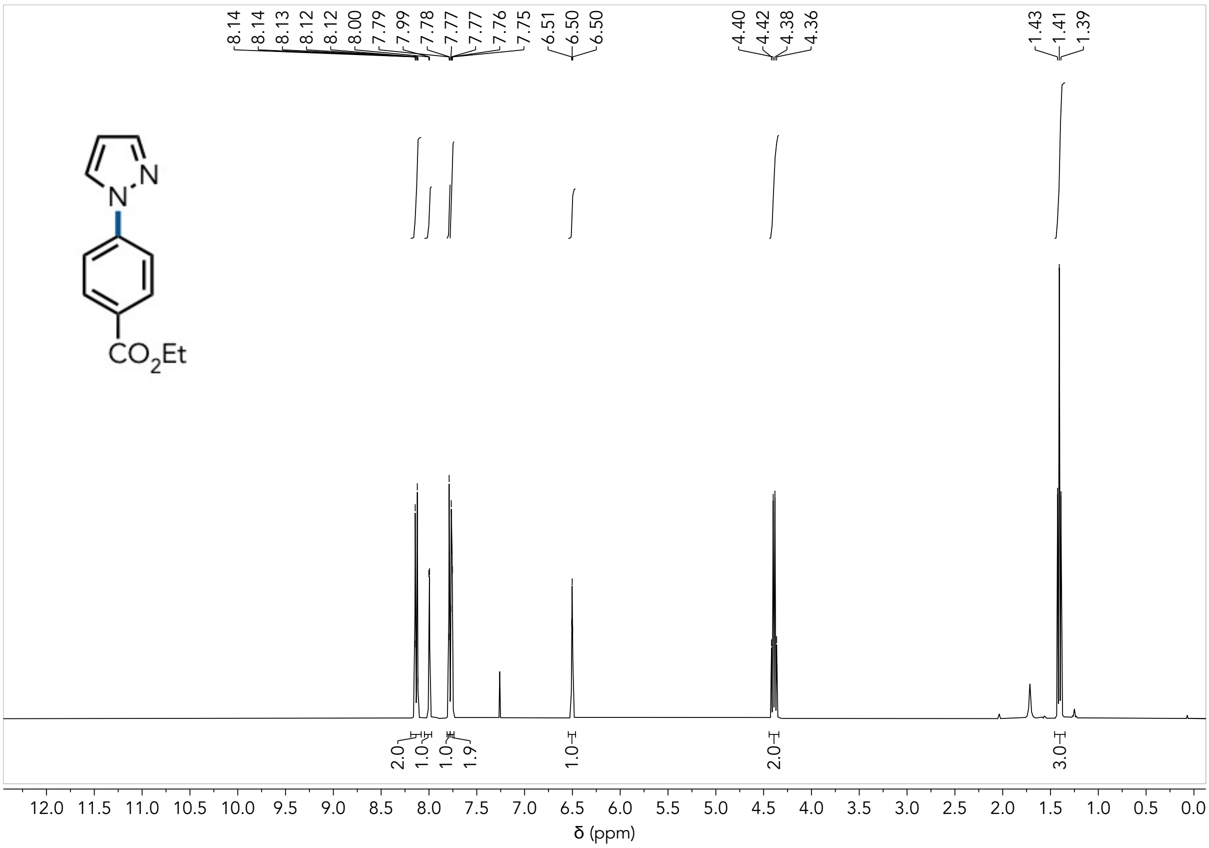

CO2Et
8.14 8.14 8.13 8.12 8.12 8.00 7.99 7.79 7.78 7.77 7.77 7.76 7.75 6.51 6.50 6.50
4.40 4.42 4.38 4.36
1.43 1.41 1.39
2.0 1.0 1.0 1.9 1.0 2.0 3.0
δ (ppm)

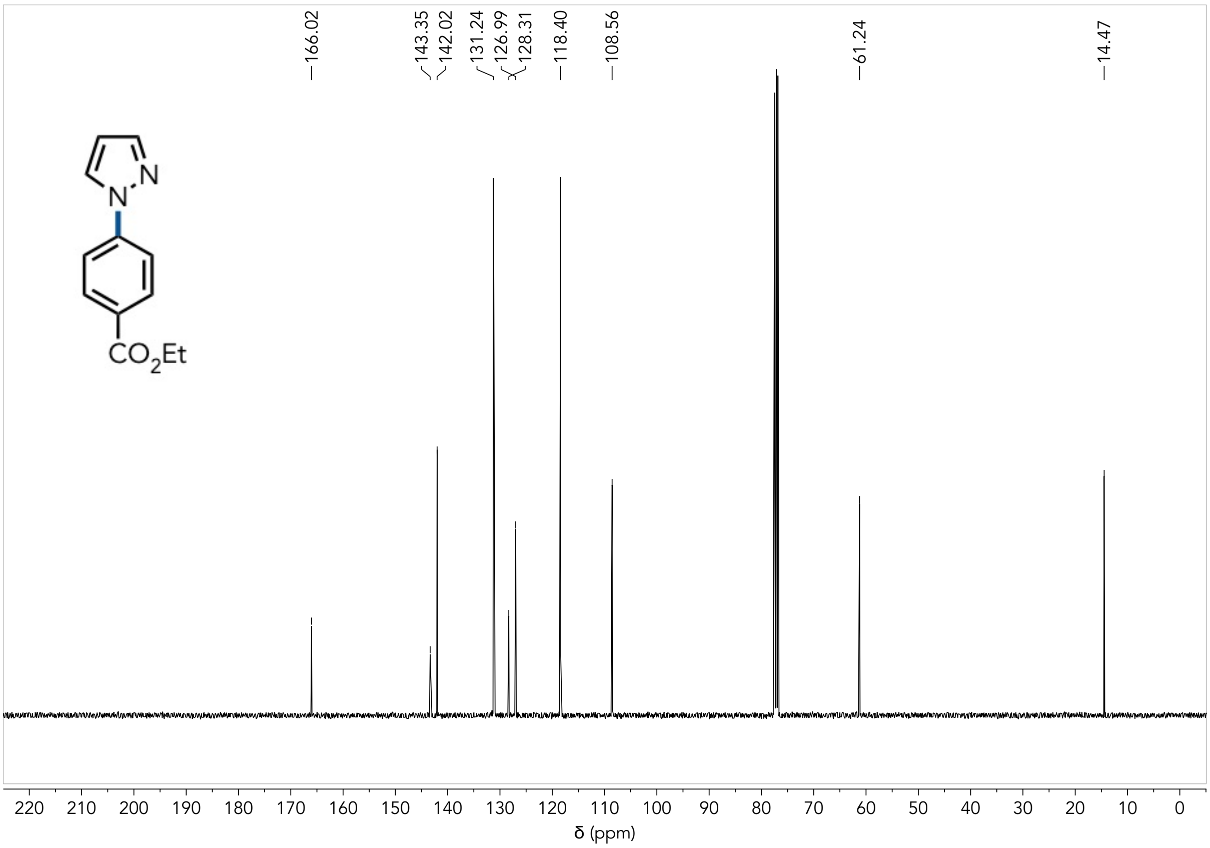
166.02
143.35
142.02
131.24
126.99
128.31
118.40
108.56
61.24
14.47
CO2Et
220 210 200 190 180 170 160 150 140 130 120 110 100 90 80 70 60 50 40 30 20 10 0
δ (ppm)

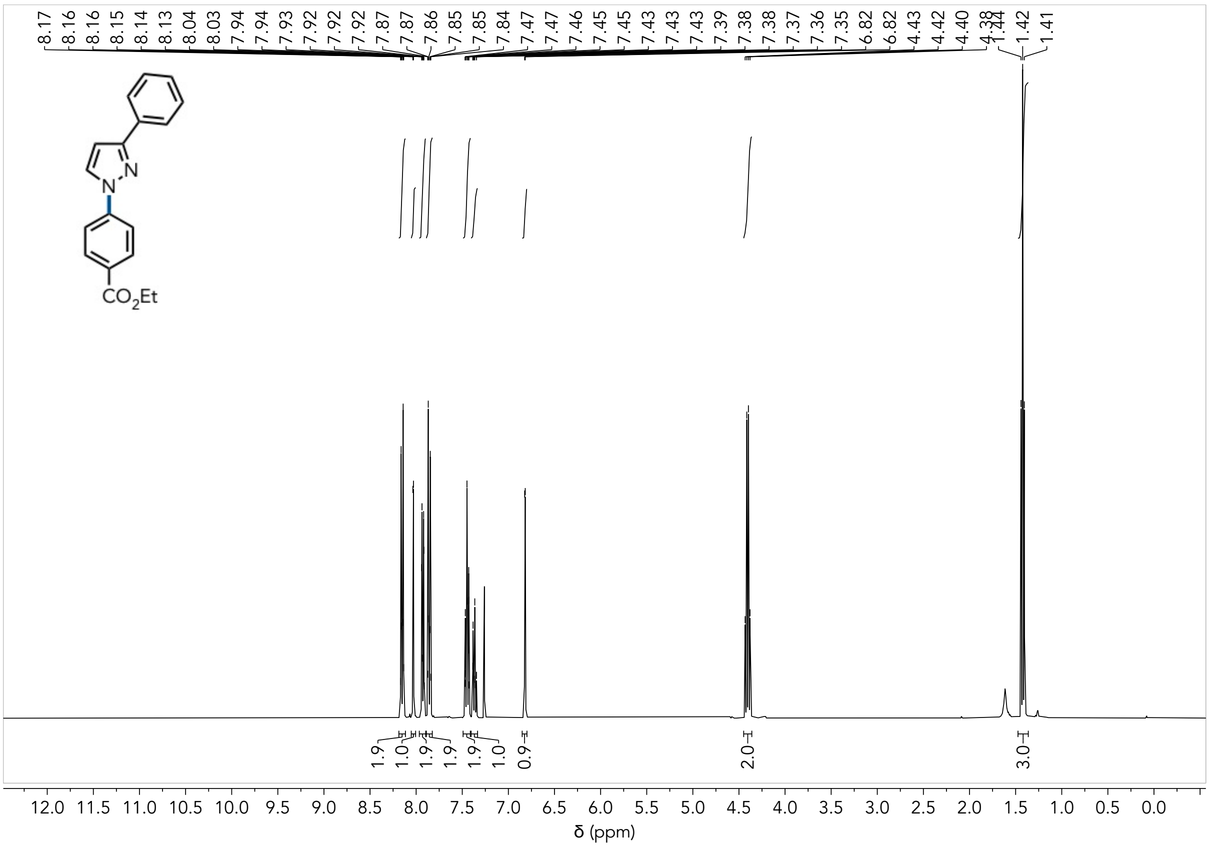
8.17 8.16 8.16 8.15 8.14 8.13 8.04 8.03 7.94 7.94 7.93 7.92 7.92 7.92 7.87 7.87 7.86 7.85 7.85 7.84 7.47 7.47 7.46 7.45 7.45 7.43 7.43 7.43 7.39 7.38 7.38 7.37 7.36 7.35 6.82 6.82 4.43 4.42 4.40 4.38 1.44 1.42 1.41
CO2Et
1.9 1.0 1.9 1.9 1.9 1.0 0.9 2.0 3.0
12.0 11.5 11.0 10.5 10.0 9.5 9.0 8.5 8.0 7.5 7.0 6.5 6.0 5.5 5.0 4.5 4.0 3.5 3.0 2.5 2.0 1.5 1.0 0.5 0.0
δ (ppm)

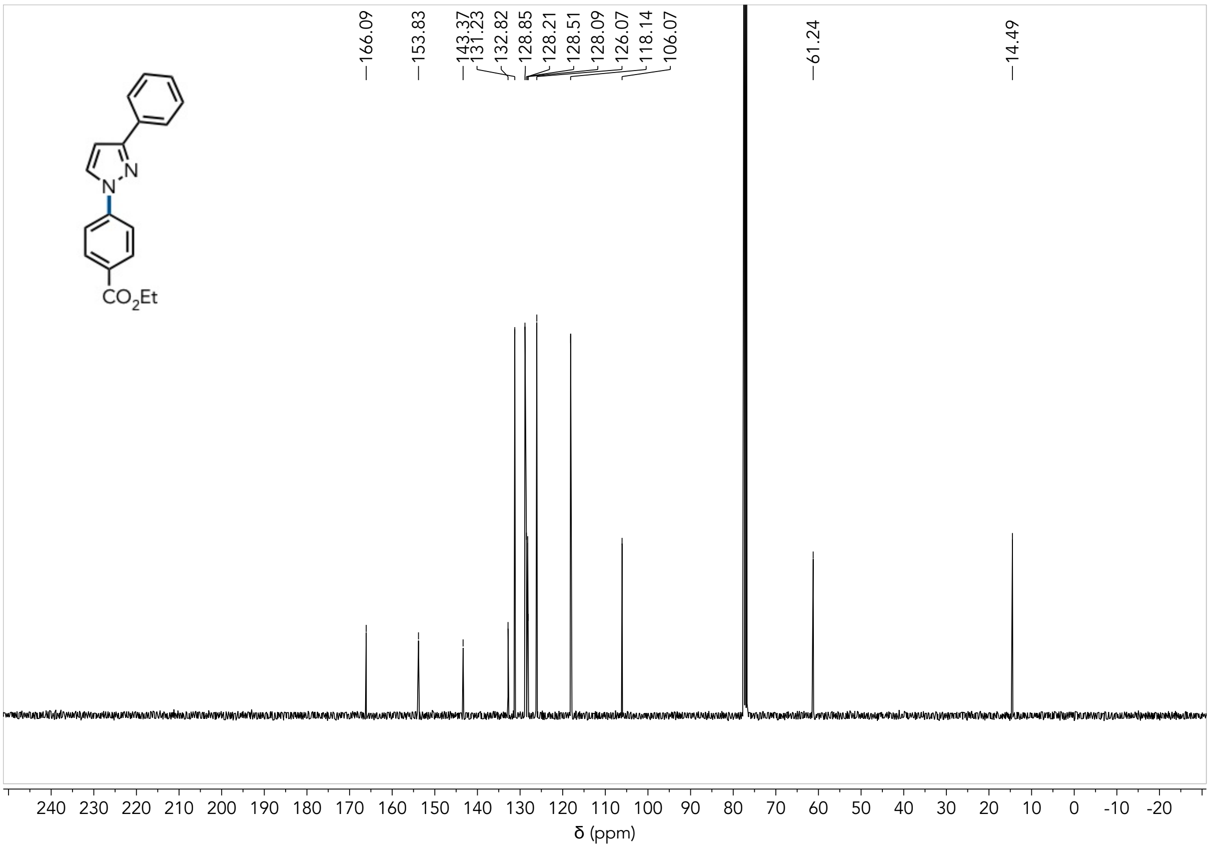
166.09
153.83
143.37
131.23
132.82
128.85
128.21
128.51
128.09
126.07
118.14
106.07
61.24
14.49
CO2Et
N
N
δ (ppm)

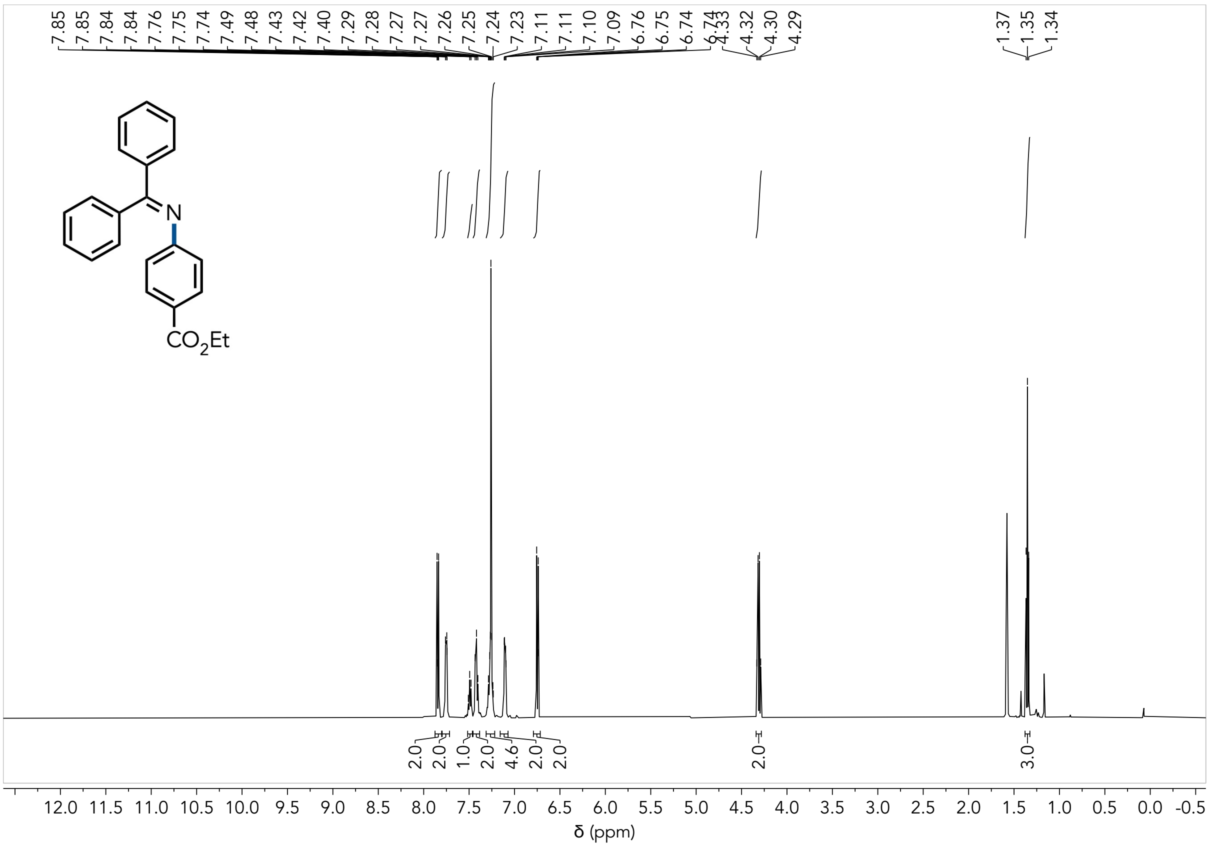
7.85
7.85
7.84
7.84
7.76
7.75
7.74
7.49
7.48
7.43
7.42
7.40
7.29
7.28
7.27
7.27
7.26
7.25
7.24
7.23
7.11
7.11
7.10
7.09
6.76
6.75
6.74
6.74
4.33
4.32
4.30
4.29
1.37
1.35
1.34
N
CO2Et
δ (ppm)

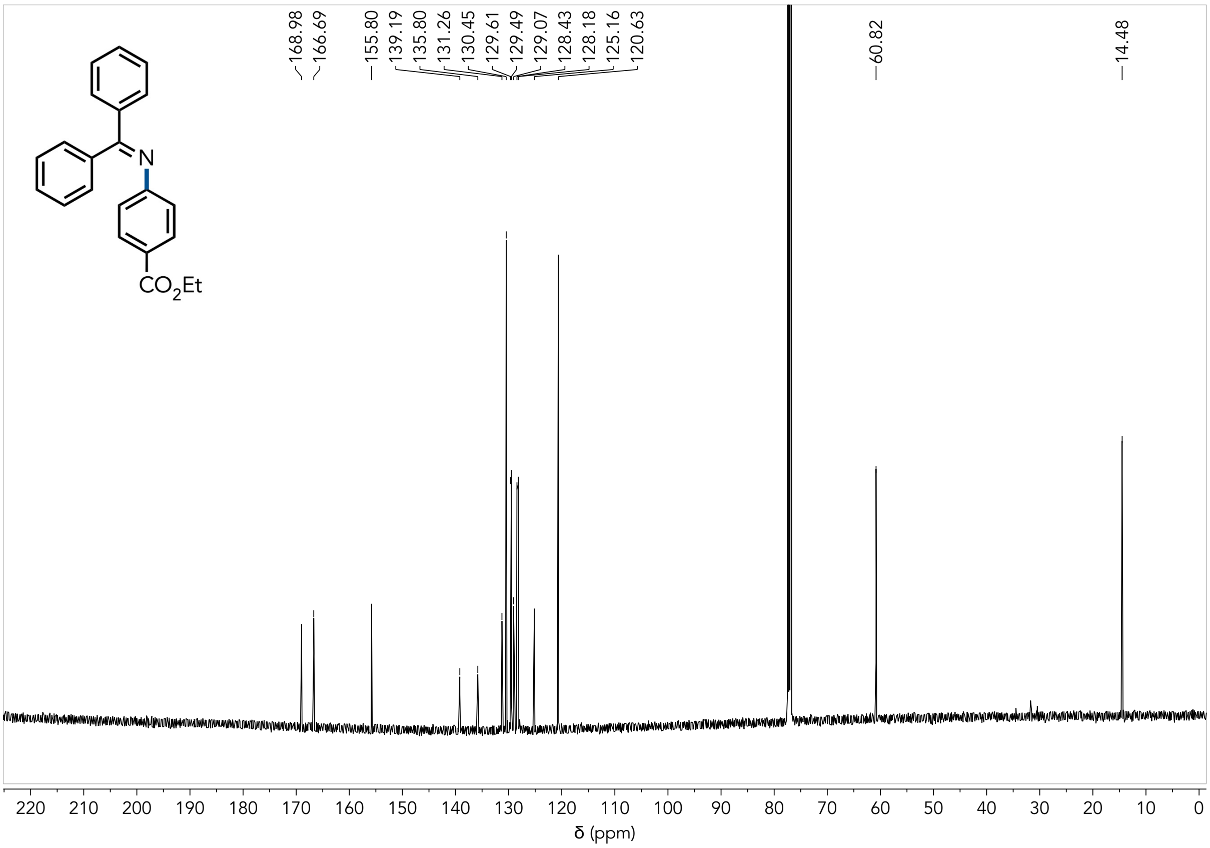
168.98
166.69
155.80
139.19
135.80
131.26
130.45
129.61
129.49
129.07
128.43
128.18
125.16
120.63
60.82
14.48
N
CO2Et
220 210 200 190 180 170 160 150 140 130 120 110 100 90 80 70 60 50 40 30 20 10 0
δ (ppm)

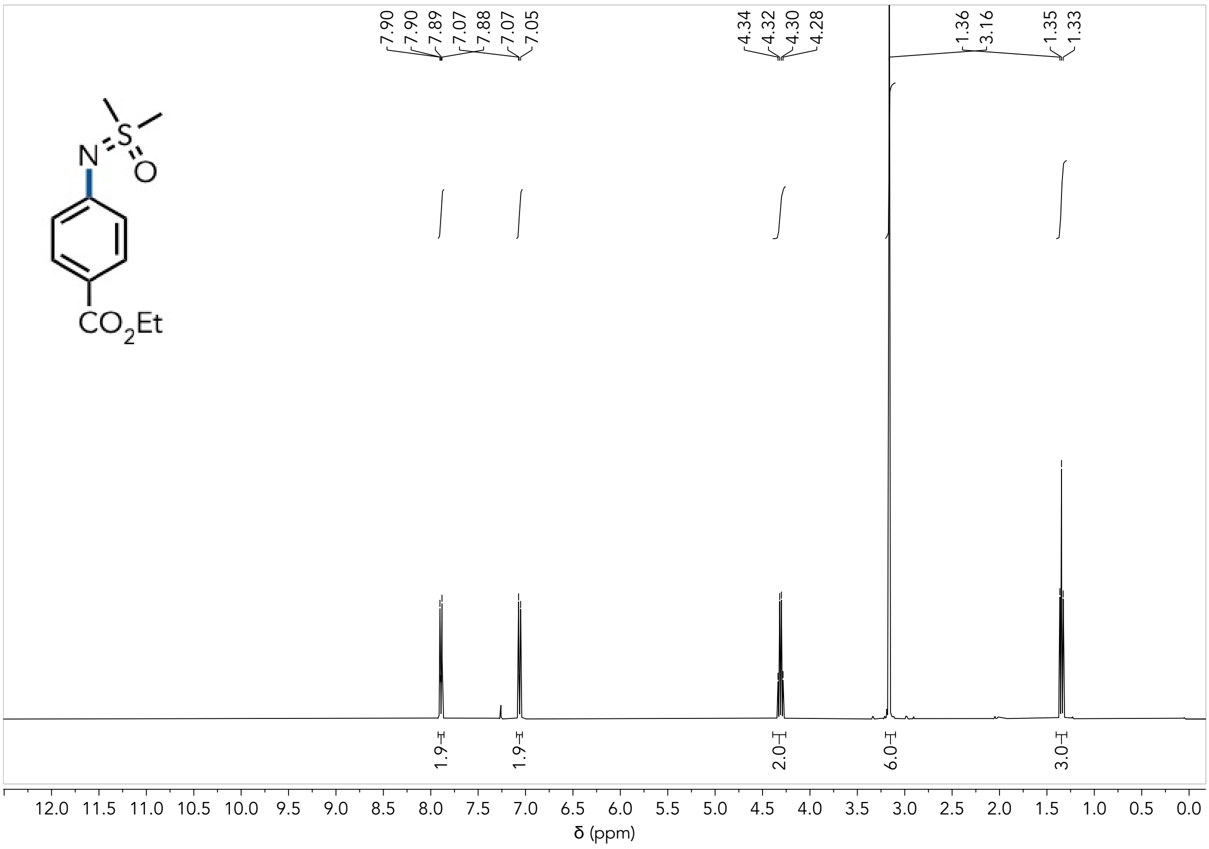
7.90
7.90
7.89
7.07
7.88
7.07
7.05
4.34
4.32
4.30
4.28
1.36
3.16
1.35
1.33
N
S
O
CO2Et
1.9
1.9
2.0
6.0
3.0
12.0 11.5 11.0 10.5 10.0 9.5 9.0 8.5 8.0 7.5 7.0 6.5 6.0 5.5 5.0 4.5 4.0 3.5 3.0 2.5 2.0 1.5 1.0 0.5 0.0
δ (ppm)

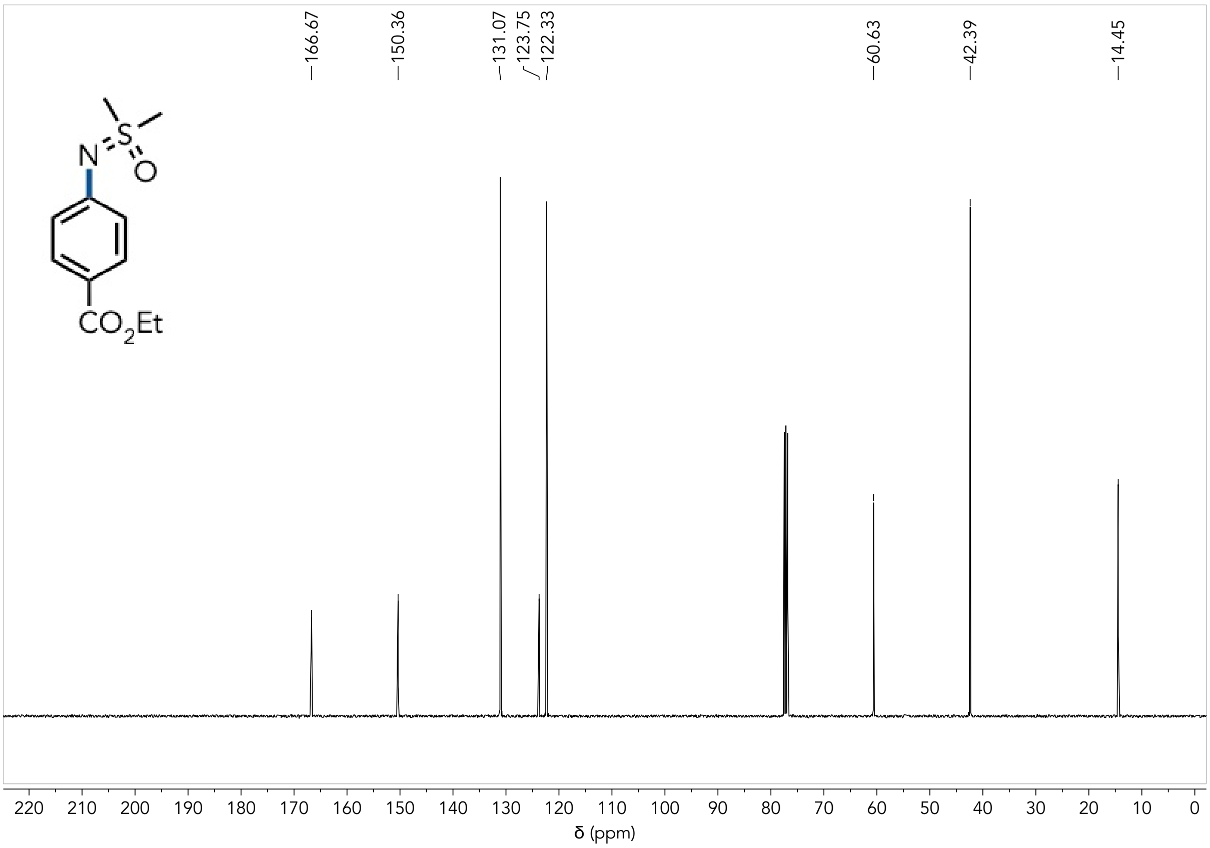

166.67
150.36
131.07
123.75
122.33
60.63
42.39
14.45
N
S
O
CO2Et
220 210 200 190 180 170 160 150 140 130 120 110 100 90 80 70 60 50 40 30 20 10 0
δ (ppm)

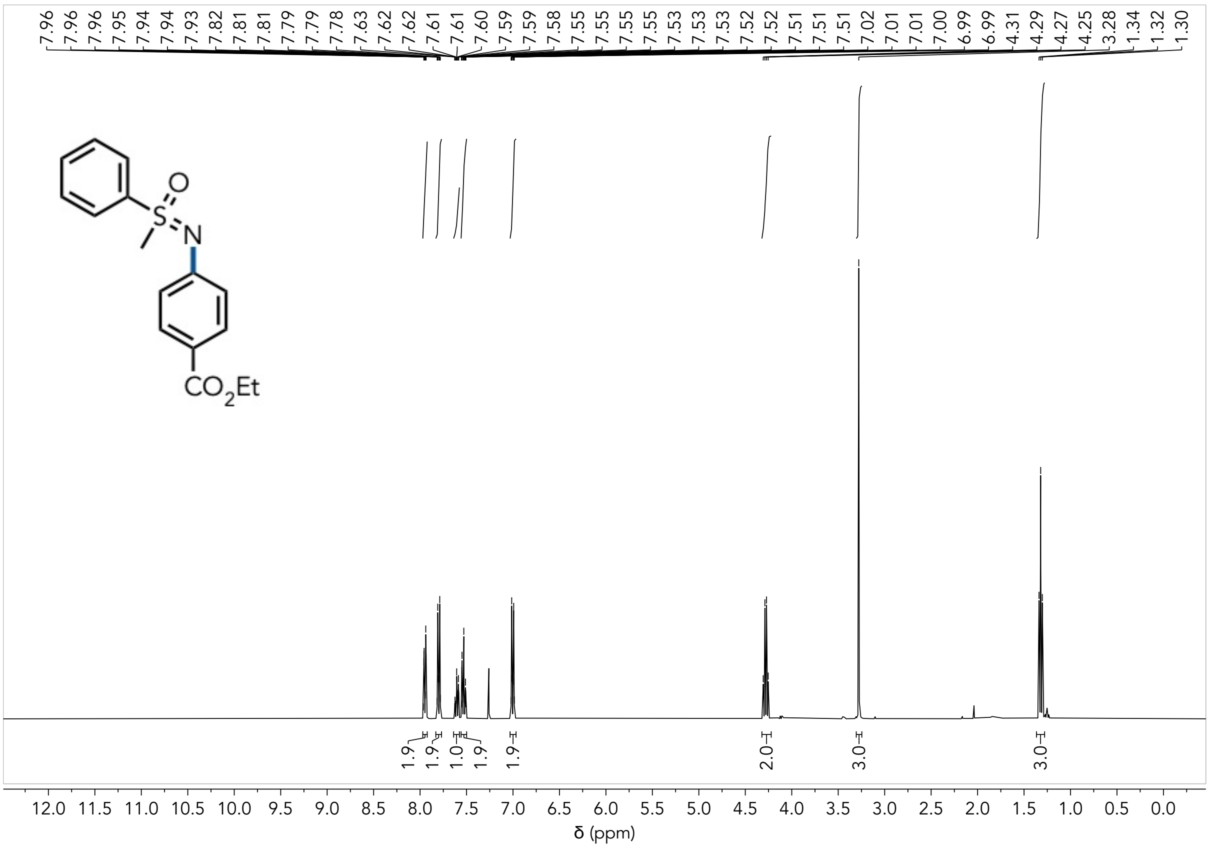

7.96 7.96 7.96 7.95 7.94 7.94 7.93 7.82 7.81 7.79 7.79 7.78 7.63 7.62 7.62 7.61 7.61 7.61 7.60 7.59 7.59 7.58 7.55 7.55 7.55 7.55 7.53 7.53 7.53 7.52 7.52 7.51 7.51 7.51 7.02 7.01 7.01 7.00 6.99 6.99 4.31 4.29 4.27 4.25 3.28 1.34 1.32 1.30
O
S
N
CO2Et
1.9 1.9 1.0 1.9 1.9 2.0 3.0 3.0
12.0 11.5 11.0 10.5 10.0 9.5 9.0 8.5 8.0 7.5 7.0 6.5 6.0 5.5 5.0 4.5 4.0 3.5 3.0 2.5 2.0 1.5 1.0 0.5 0.0
δ (ppm)

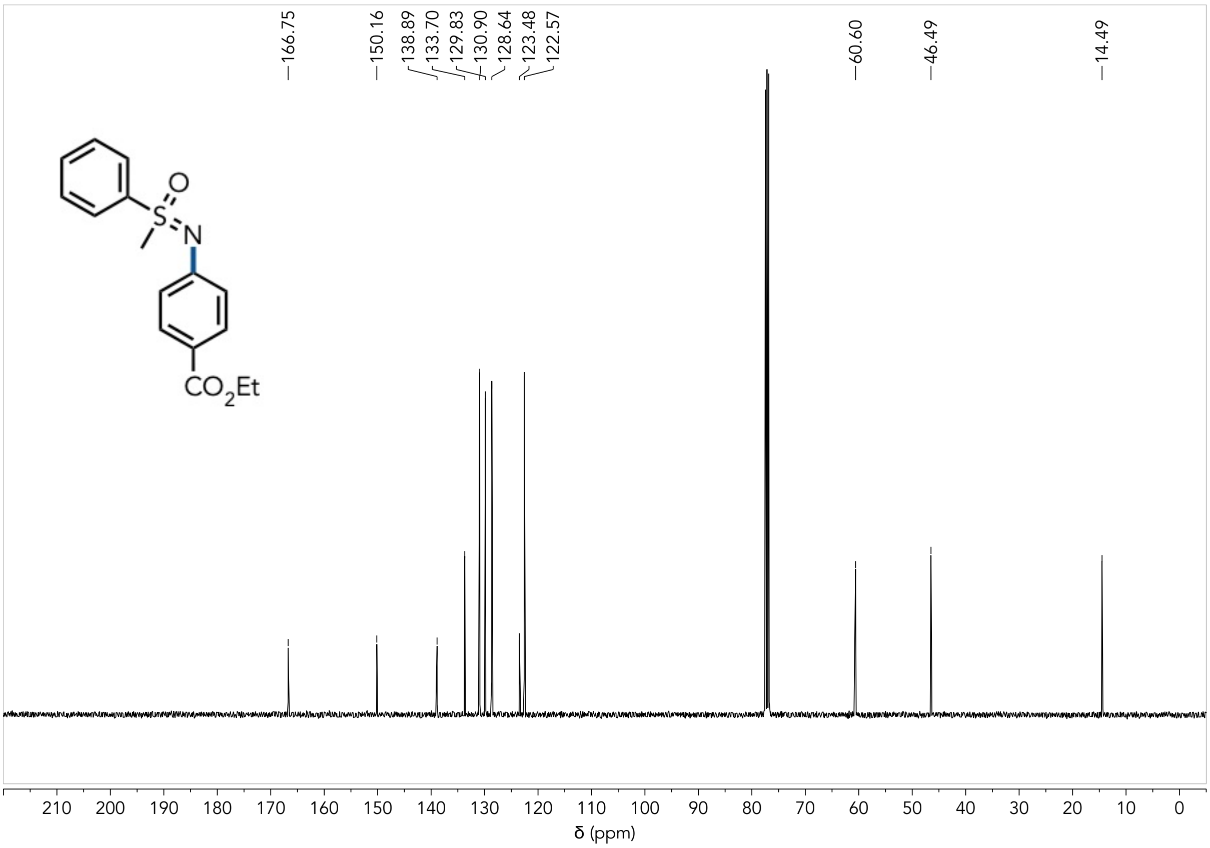
166.75
150.16
138.89
133.70
129.83
130.90
128.64
123.48
122.57
60.60
46.49
14.49
O
S
N
CO2Et
210 200 190 180 170 160 150 140 130 120 110 100 90 80 70 60 50 40 30 20 10 0
δ (ppm)

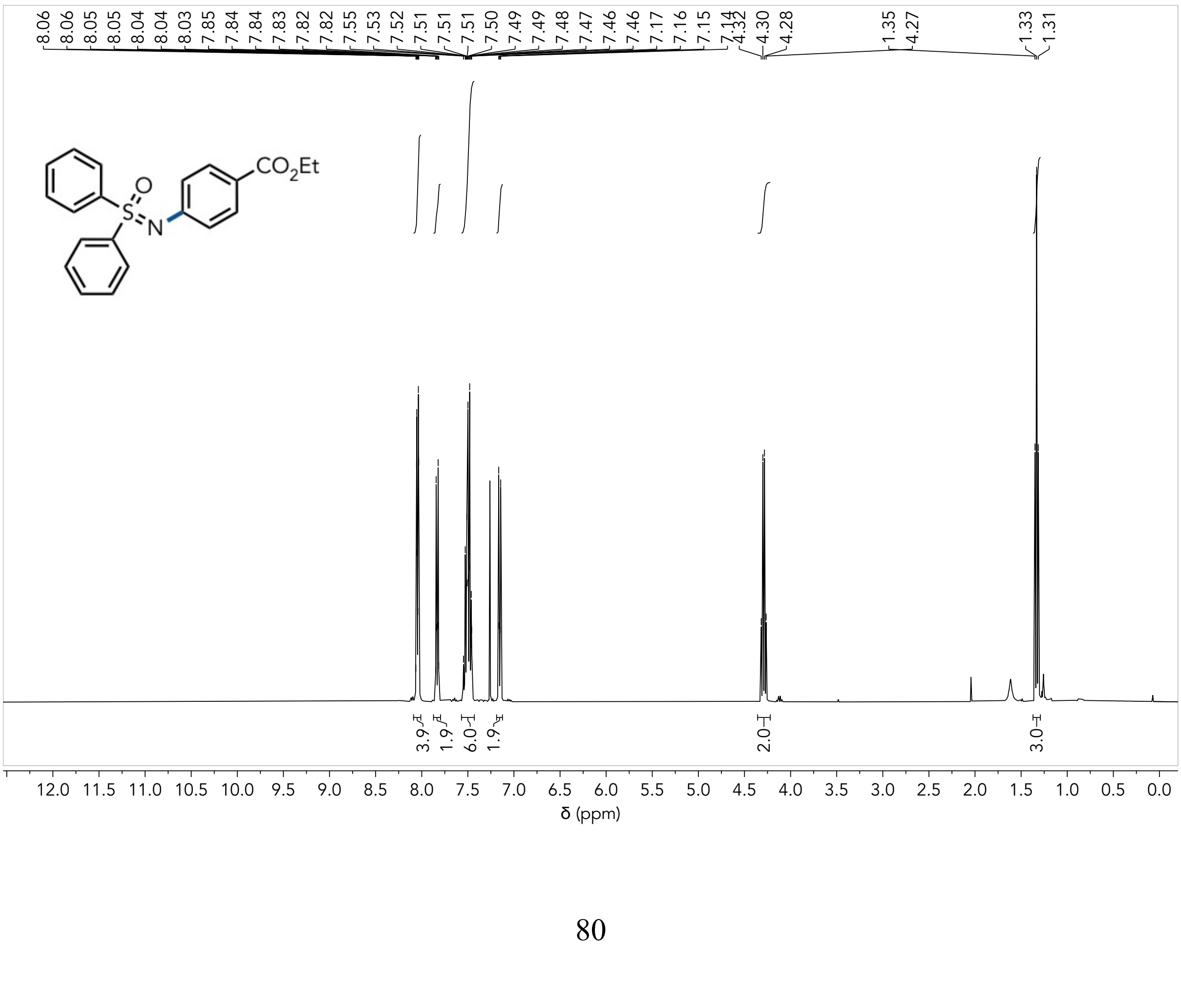
8.06 8.06 8.05 8.05 8.04 8.04 8.03 7.85 7.84 7.84 7.83 7.82 7.82 7.55 7.53 7.52 7.51 7.51 7.51 7.50 7.49 7.49 7.48 7.47 7.46 7.46 7.17 7.16 7.15 7.14 4.32 4.30 4.28 1.35 4.27 1.33 1.31
CO2Et
O
S
N
3.9
1.9
6.0
1.9
2.0
3.0
12.0 11.5 11.0 10.5 10.0 9.5 9.0 8.5 8.0 7.5 7.0 6.5 6.0 5.5 5.0 4.5 4.0 3.5 3.0 2.5 2.0 1.5 1.0 0.5 0.0
δ (ppm)

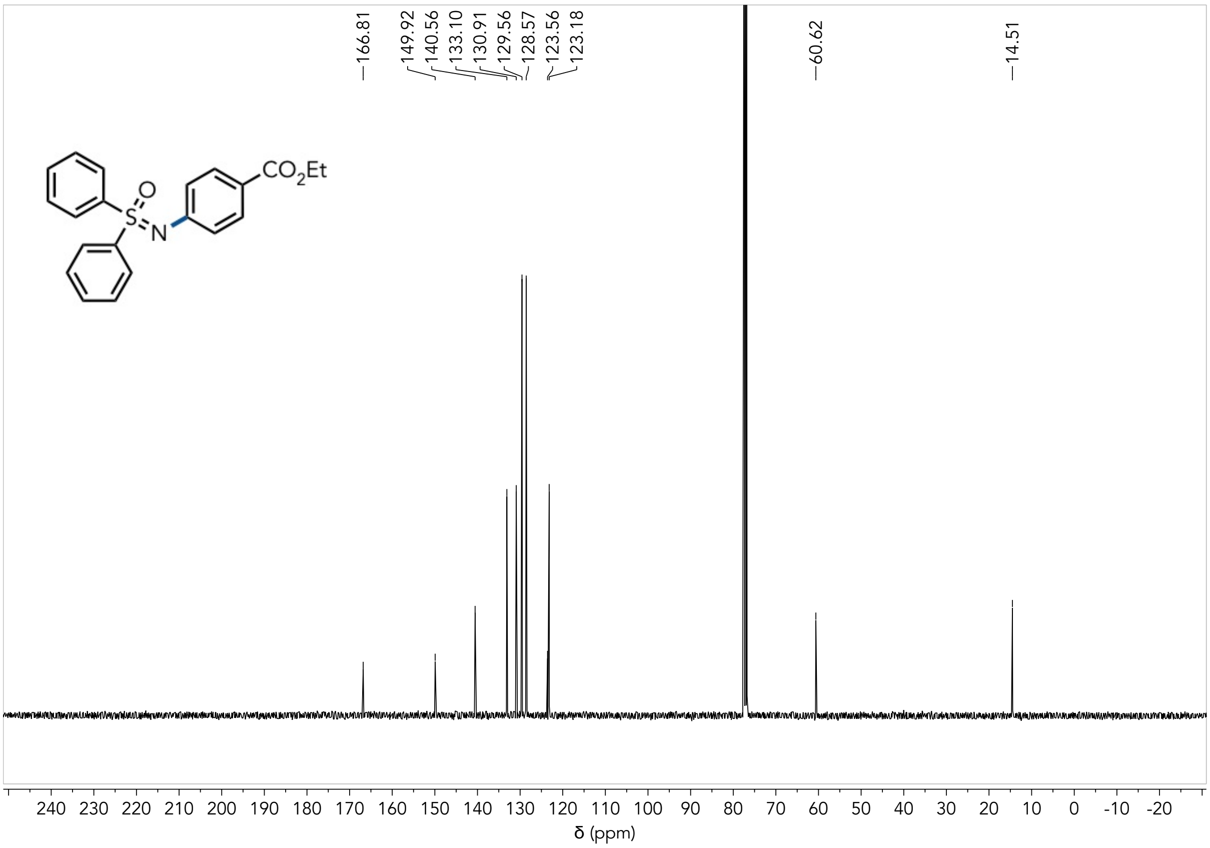
166.81
149.92
140.56
133.10
130.91
129.56
128.57
123.56
123.18
60.62
14.51
CO2Et
O
S
N
240 230 220 210 200 190 180 170 160 150 140 130 120 110 100 90 80 70 60 50 40 30 20 10 0 -10 -20
δ (ppm)

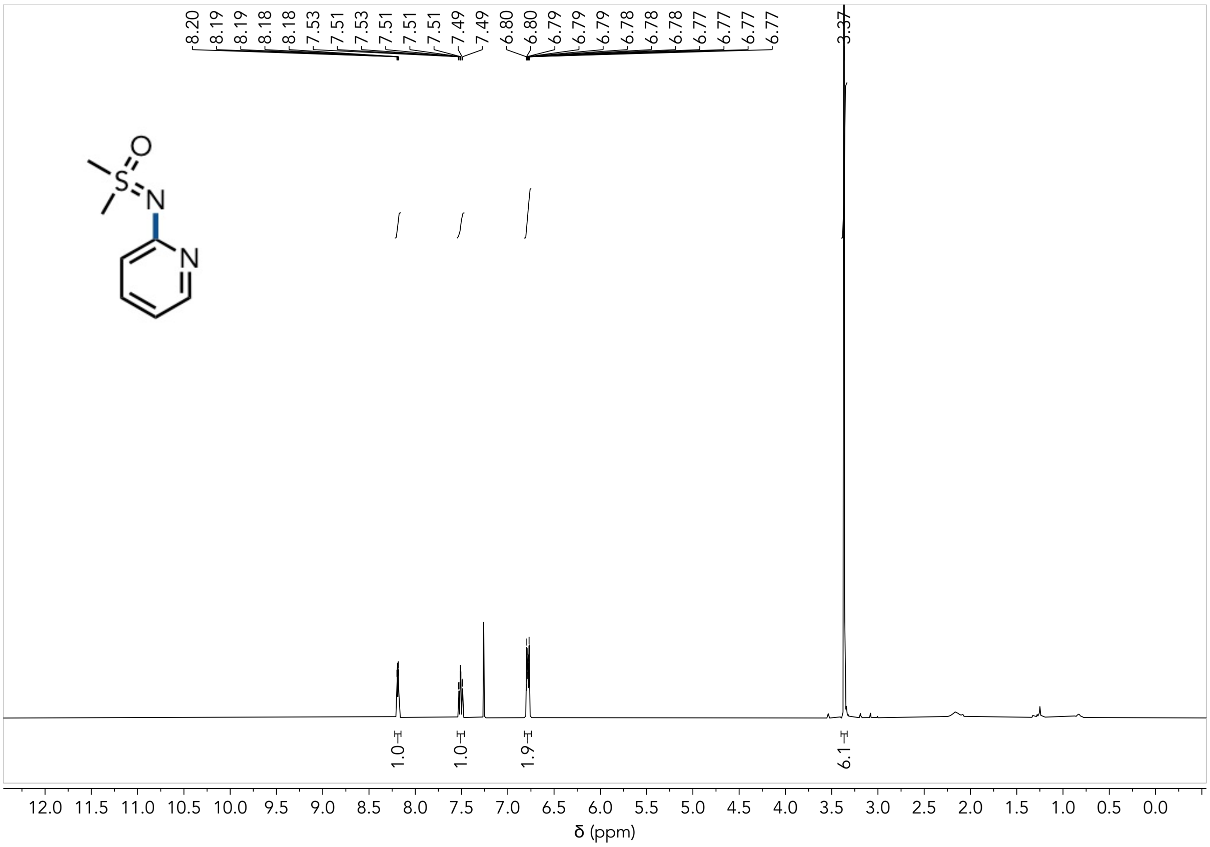
8.20
8.19
8.19
8.18
8.18
7.53
7.51
7.53
7.51
7.51
7.51
7.49
6.80
6.80
6.79
6.79
6.79
6.78
6.78
6.78
6.77
6.77
6.77
6.77
3.37
O
S
N
N
1.0
1.0
1.9
6.1
12.0 11.5 11.0 10.5 10.0 9.5 9.0 8.5 8.0 7.5 7.0 6.5 6.0 5.5 5.0 4.5 4.0 3.5 3.0 2.5 2.0 1.5 1.0 0.5 0.0
δ (ppm)

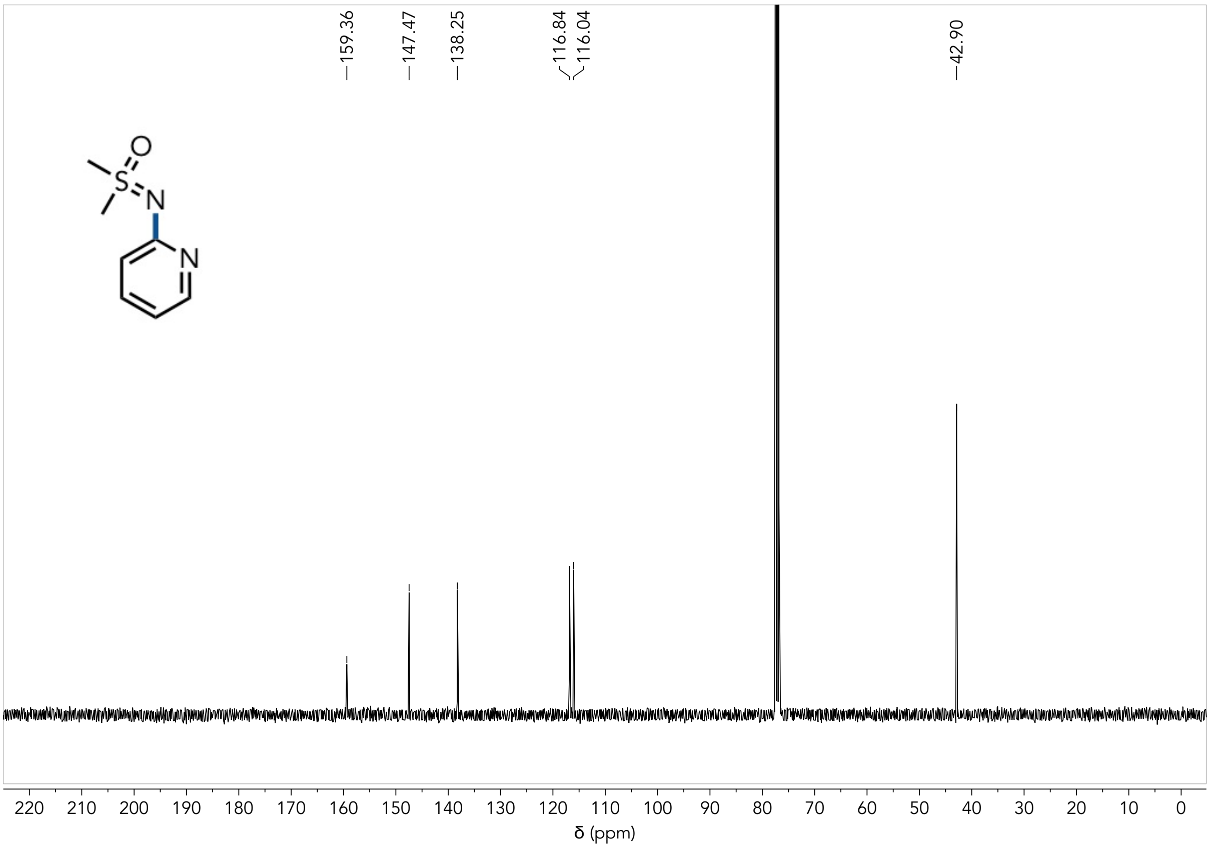
159.36
147.47
138.25
116.84
116.04
42.90
O
S
N
N
220 210 200 190 180 170 160 150 140 130 120 110 100 90 80 70 60 50 40 30 20 10 0
δ (ppm)

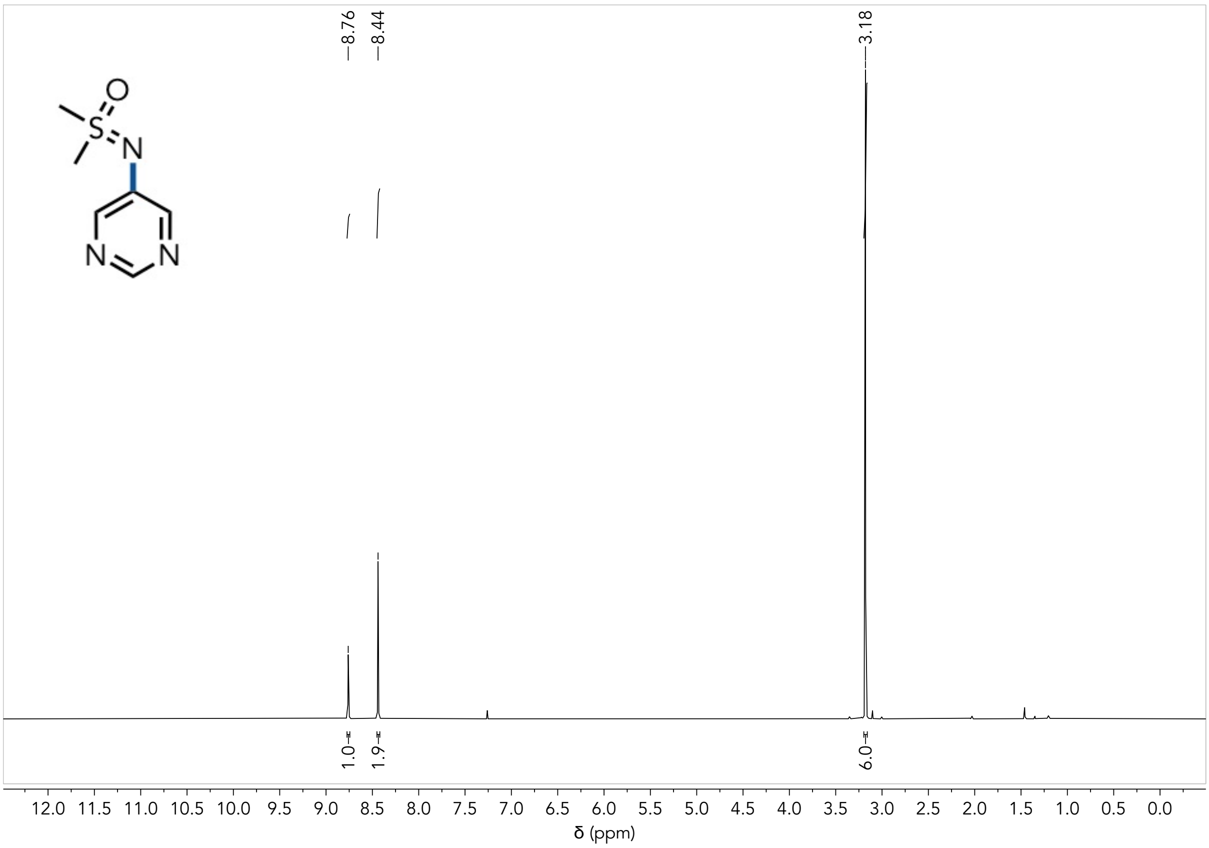
8.76
8.44
3.18
O
S
N
N
N
1.0
1.9
6.0
12.0 11.5 11.0 10.5 10.0 9.5 9.0 8.5 8.0 7.5 7.0 6.5 6.0 5.5 5.0 4.5 4.0 3.5 3.0 2.5 2.0 1.5 1.0 0.5 0.0
δ (ppm)

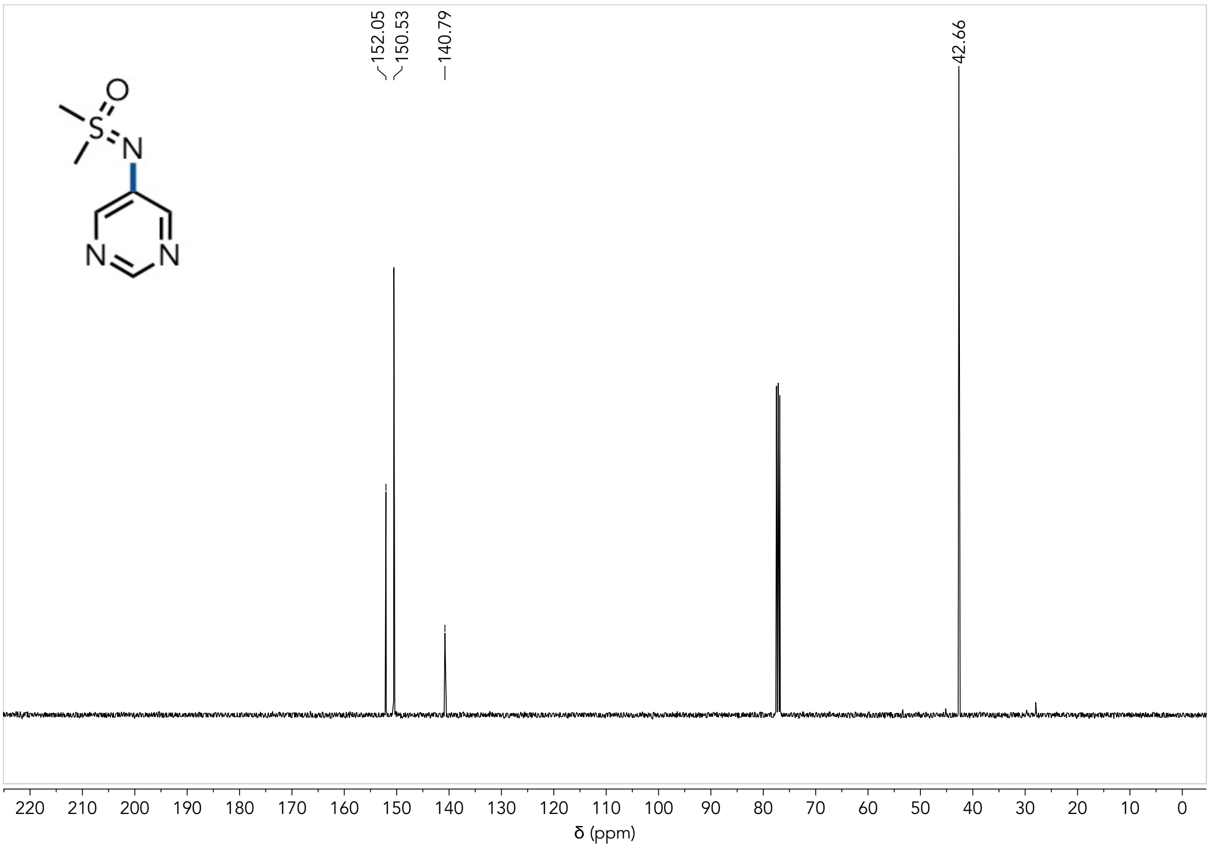

152.05
150.53
140.79
42.66
O
S
N
N
N
220 210 200 190 180 170 160 150 140 130 120 110 100 90 80 70 60 50 40 30 20 10 0
δ (ppm)

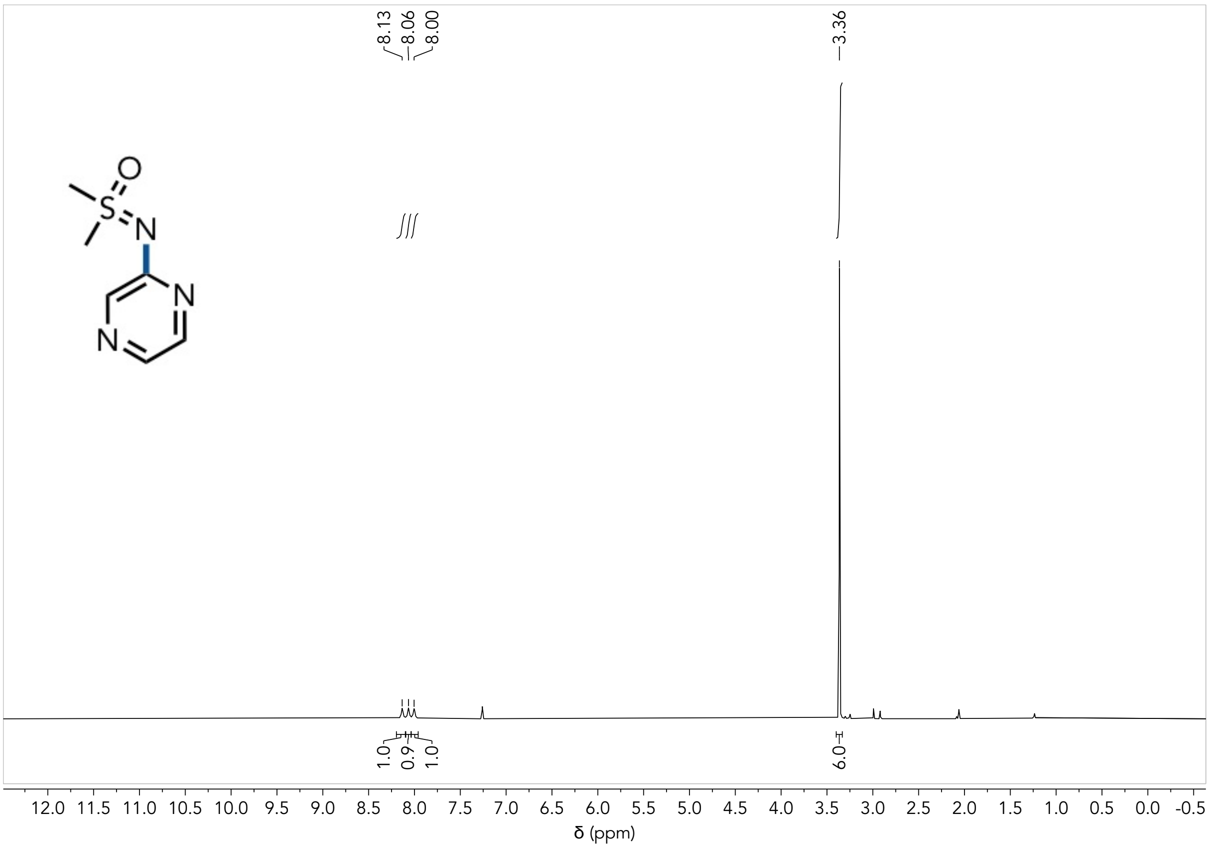

8.13
8.06
8.00
3.36
O
S
N
N
N
1.0
0.9
1.0
6.0
12.0 11.5 11.0 10.5 10.0 9.5 9.0 8.5 8.0 7.5 7.0 6.5 6.0 5.5 5.0 4.5 4.0 3.5 3.0 2.5 2.0 1.5 1.0 0.5 0.0 -0.5
δ (ppm)

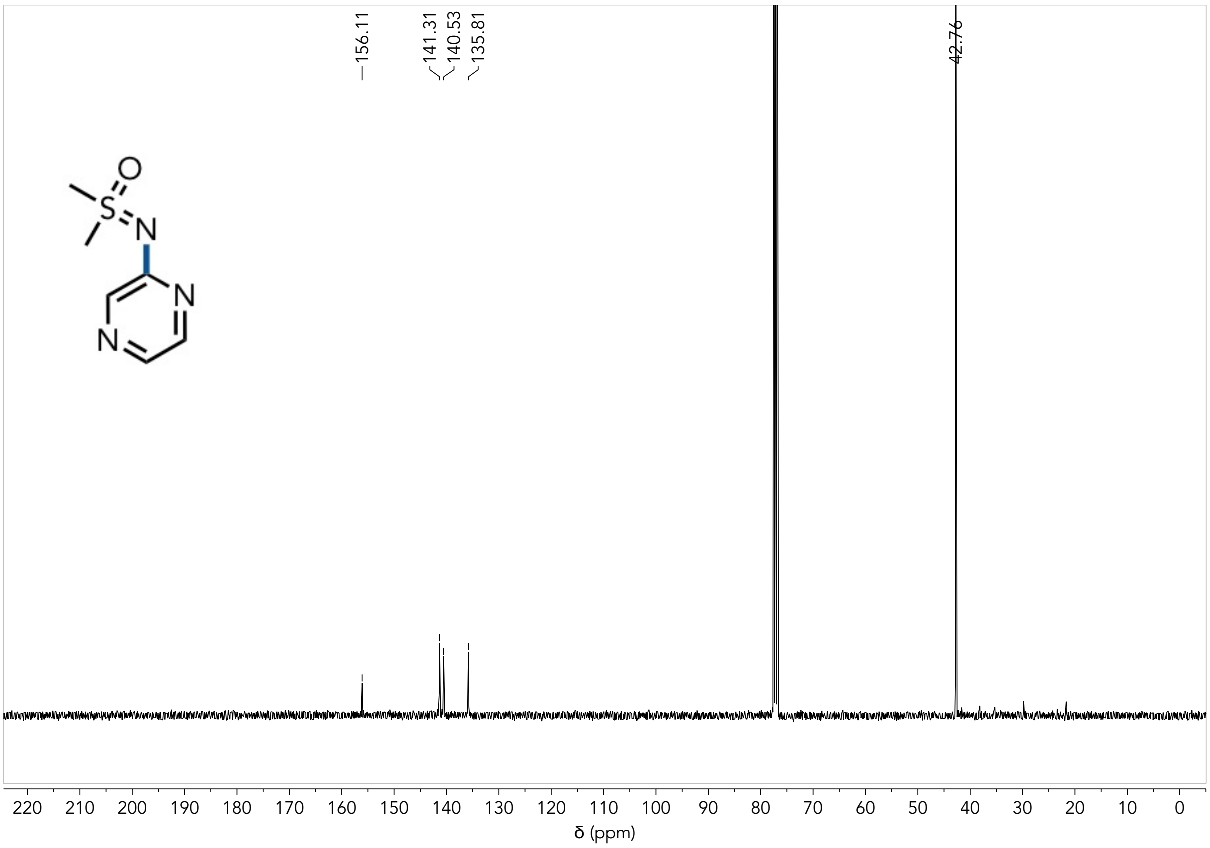
156.11
141.31
140.53
135.81
42.76
220 210 200 190 180 170 160 150 140 130 120 110 100 90 80 70 60 50 40 30 20 10 0
δ (ppm)

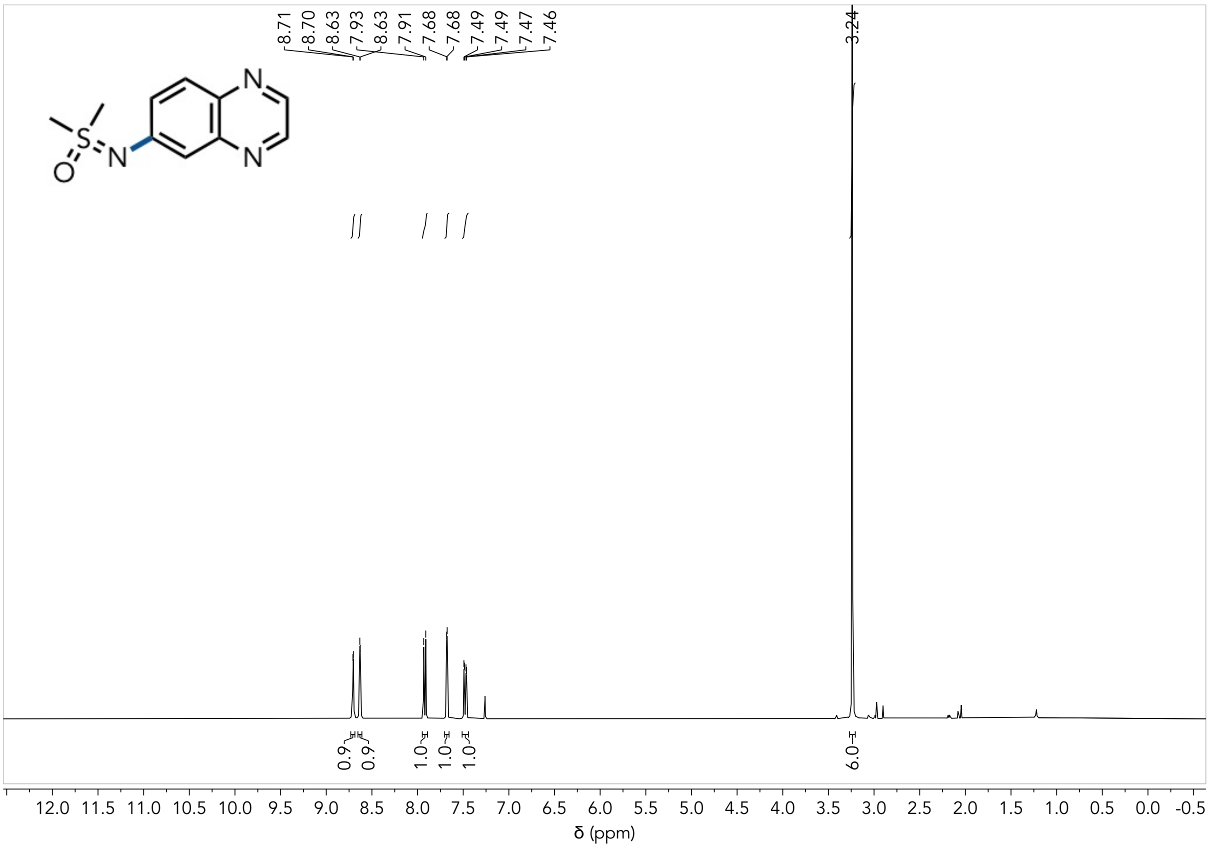
8.71
8.70
8.63
7.93
8.63
7.91
7.68
7.68
7.49
7.49
7.47
7.46
3.24
0.9
0.9
1.0
1.0
1.0
6.0
12.0 11.5 11.0 10.5 10.0 9.5 9.0 8.5 8.0 7.5 7.0 6.5 6.0 5.5 5.0 4.5 4.0 3.5 3.0 2.5 2.0 1.5 1.0 0.5 0.0 -0.5
δ (ppm)

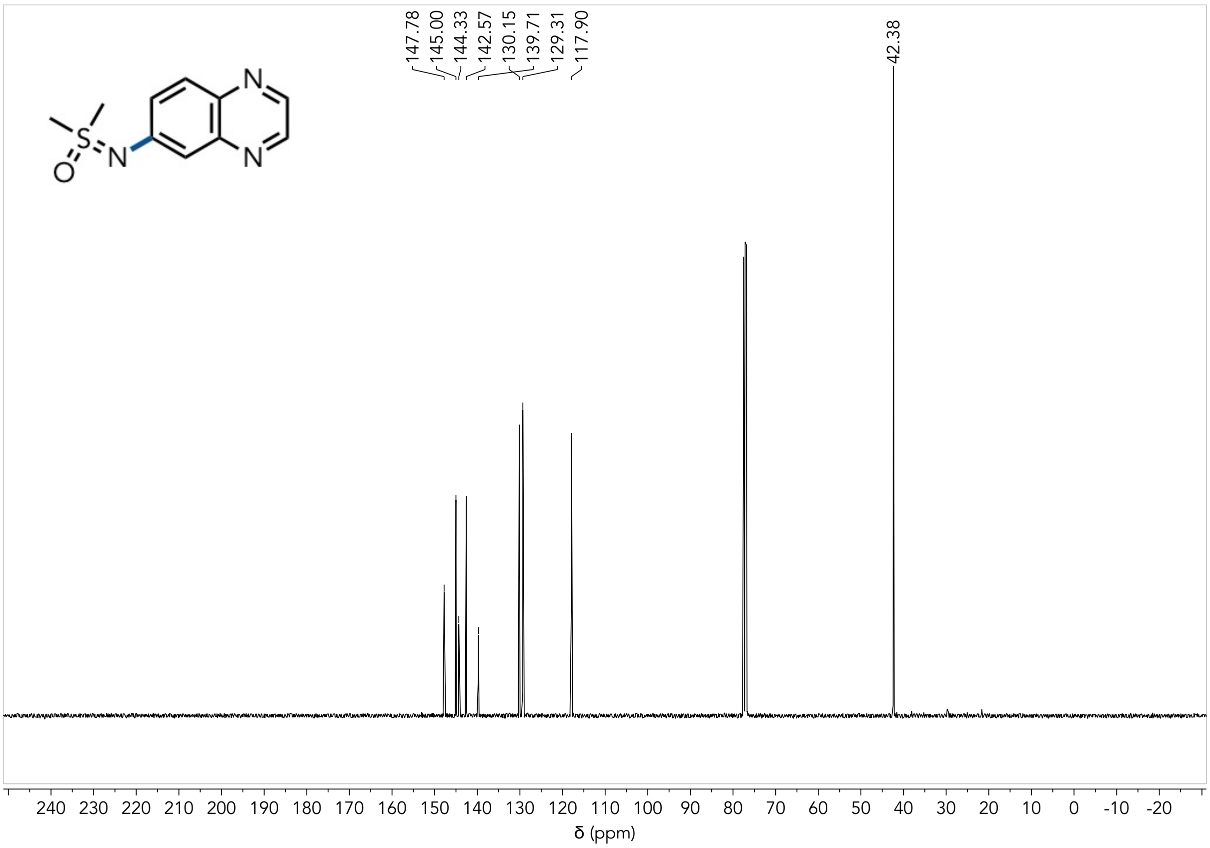
147.78
145.00
144.33
142.57
130.15
139.71
129.31
117.90
42.38
240 230 220 210 200 190 180 170 160 150 140 130 120 110 100 90 80 70 60 50 40 30 20 10 0 -10 -20
δ (ppm)

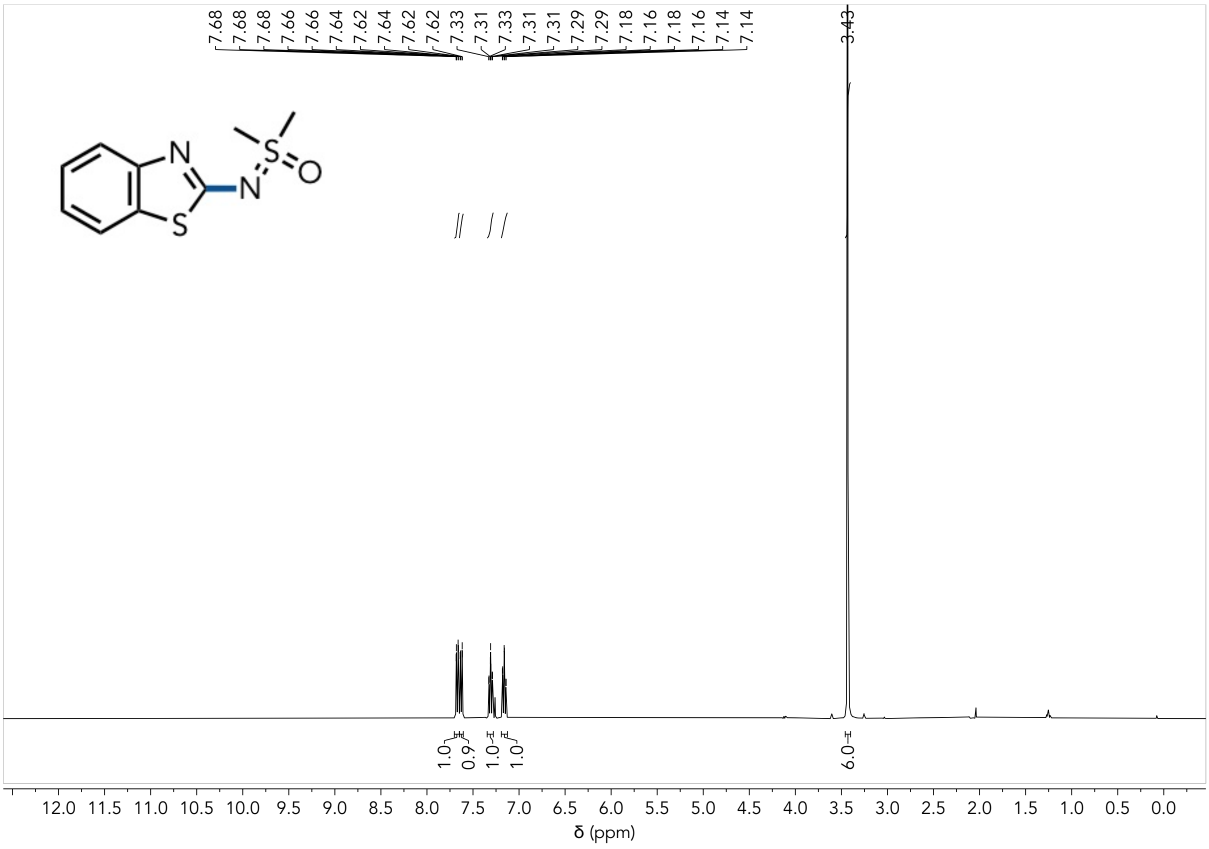
7.68
7.68
7.68
7.66
7.66
7.64
7.62
7.64
7.62
7.62
7.33
7.31
7.33
7.31
7.31
7.29
7.29
7.18
7.16
7.18
7.16
7.14
7.14
3.43
1.0
0.9
1.0
1.0
6.0
12.0 11.5 11.0 10.5 10.0 9.5 9.0 8.5 8.0 7.5 7.0 6.5 6.0 5.5 5.0 4.5 4.0 3.5 3.0 2.5 2.0 1.5 1.0 0.5 0.0
δ (ppm)

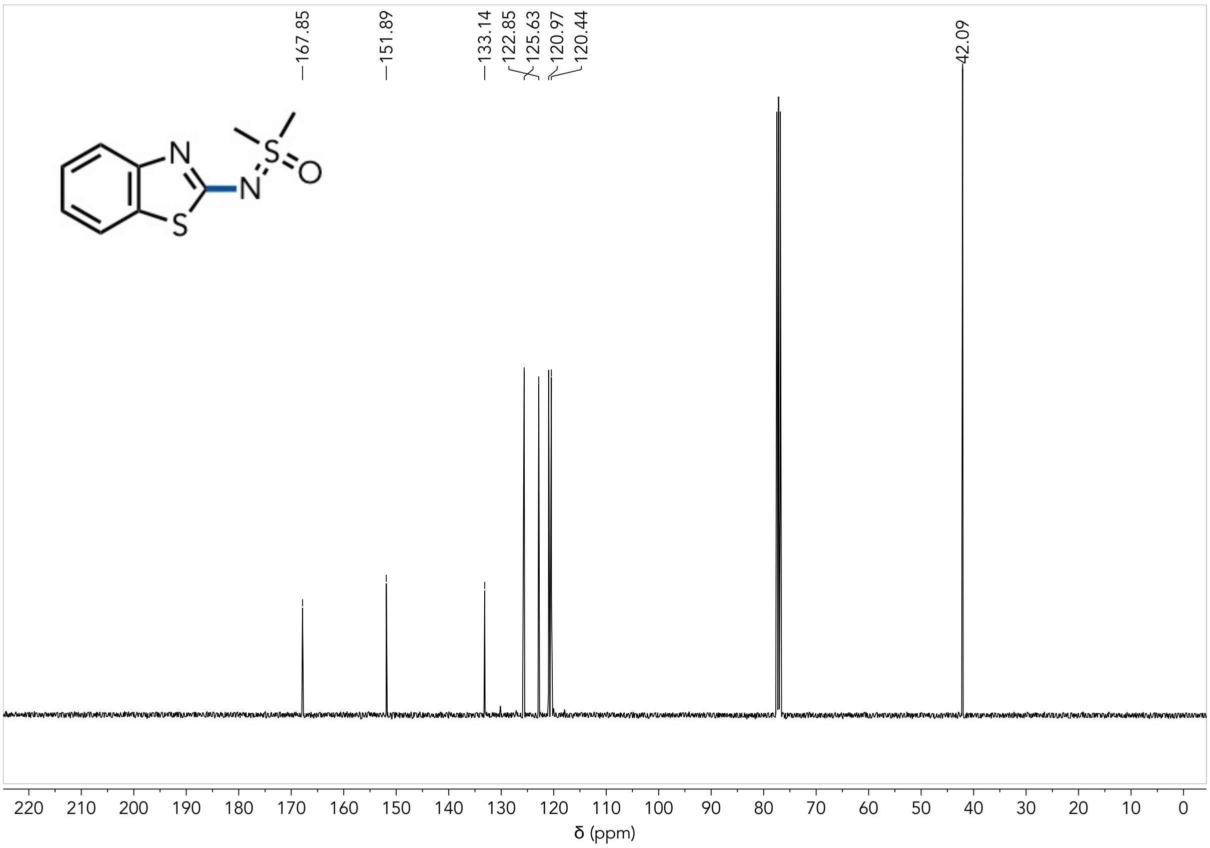

167.85
151.89
133.14
122.85
125.63
120.97
120.44
42.09
N
S
N
S
O
220 210 200 190 180 170 160 150 140 130 120 110 100 90 80 70 60 50 40 30 20 10 0
δ (ppm)

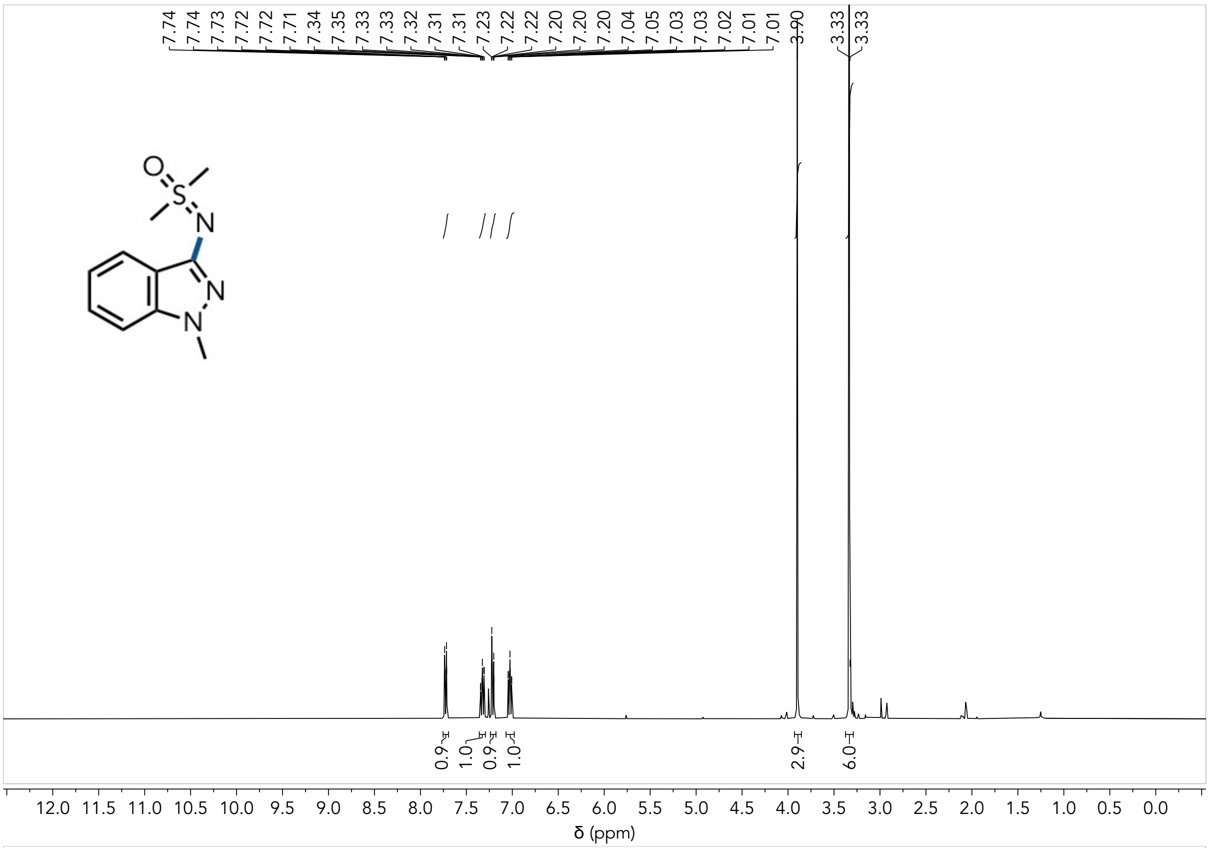
7.74
7.74
7.73
7.72
7.72
7.71
7.34
7.35
7.33
7.33
7.32
7.31
7.23
7.22
7.22
7.20
7.20
7.20
7.04
7.05
7.03
7.03
7.02
7.01
7.01
3.90
3.33
3.33
0.9
1.0
0.9
1.0
2.9
6.0
12.0 11.5 11.0 10.5 10.0 9.5 9.0 8.5 8.0 7.5 7.0 6.5 6.0 5.5 5.0 4.5 4.0 3.5 3.0 2.5 2.0 1.5 1.0 0.5 0.0
δ (ppm)

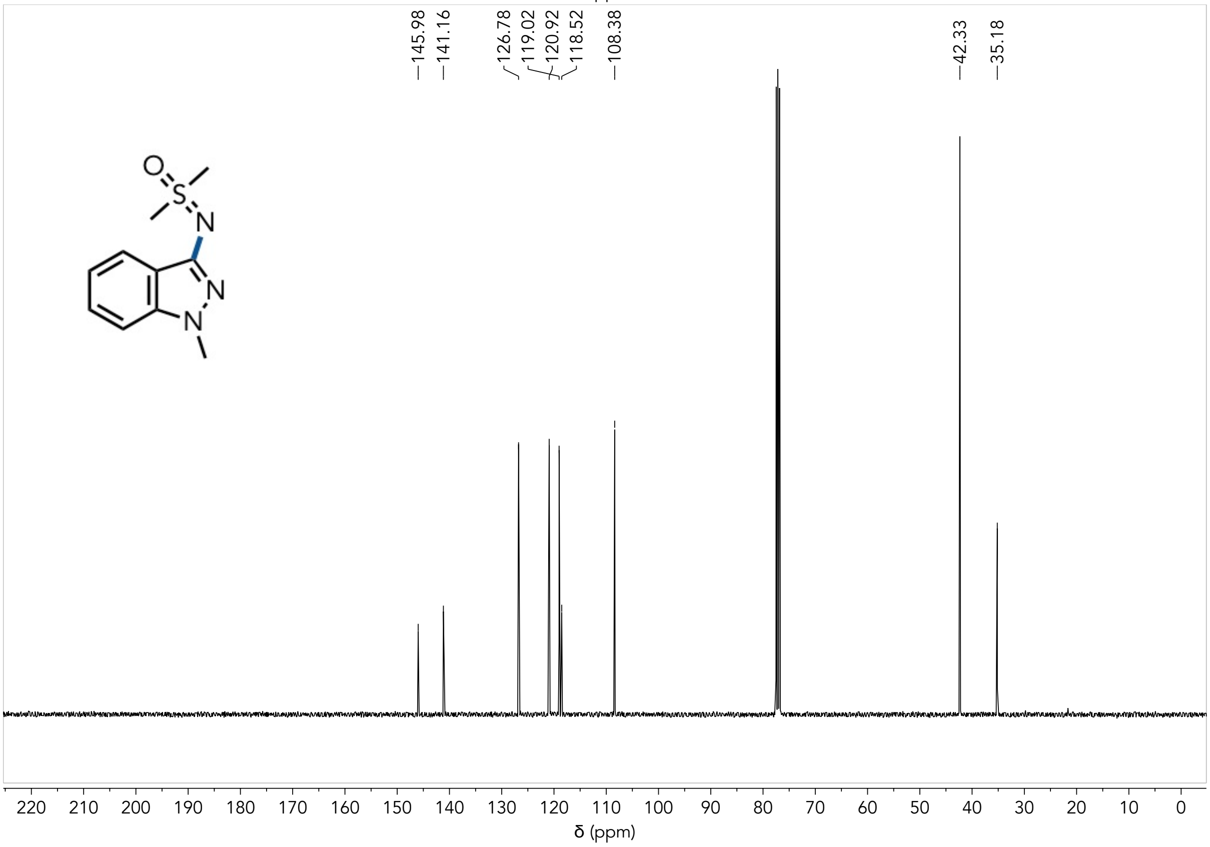
145.98
141.16
126.78
119.02
120.92
118.52
108.38
42.33
35.18
220 210 200 190 180 170 160 150 140 130 120 110 100 90 80 70 60 50 40 30 20 10 0
δ (ppm)

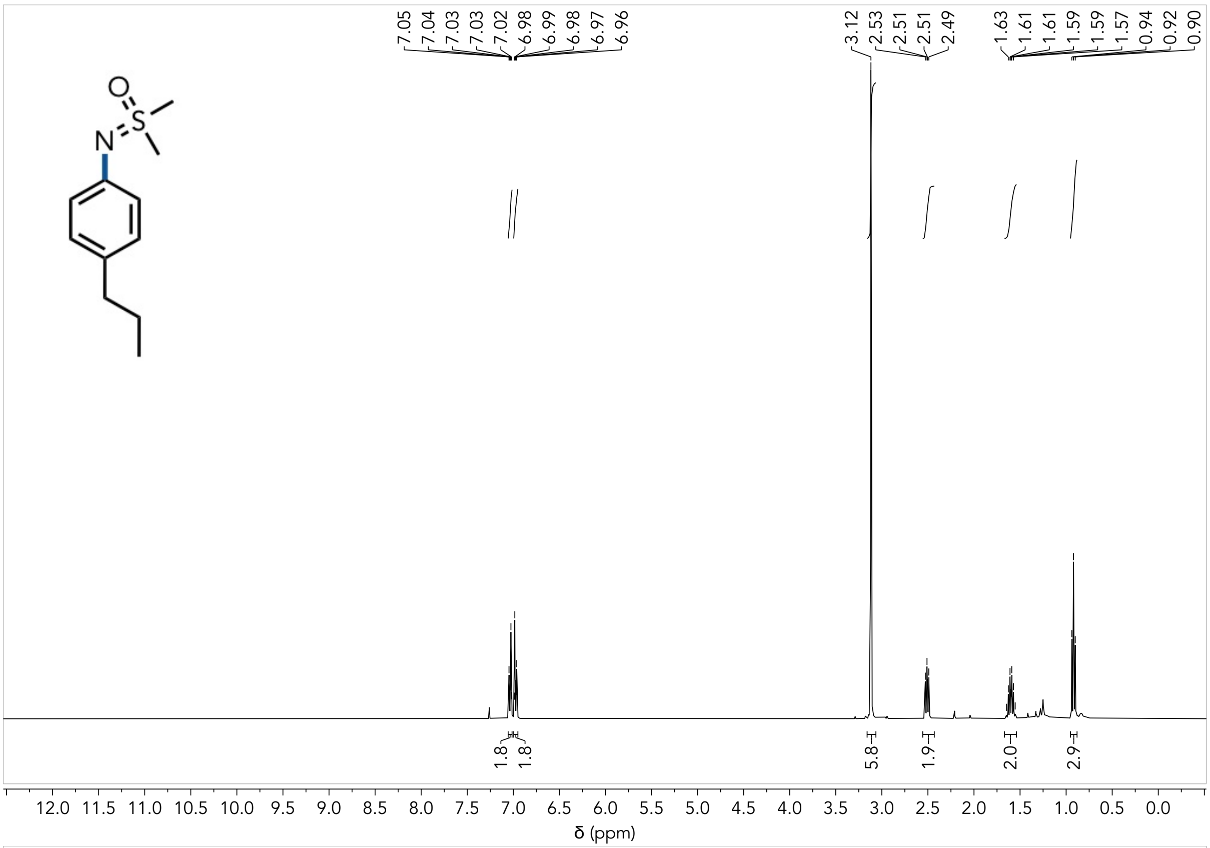
7.05
7.04
7.03
7.03
7.02
6.98
6.99
6.98
6.97
6.96
3.12
2.53
2.51
2.51
2.49
1.63
1.61
1.61
1.59
1.59
1.57
0.94
0.92
0.90
N
S
O
1.8
1.8
5.8
1.9
2.0
2.9
12.0 11.5 11.0 10.5 10.0 9.5 9.0 8.5 8.0 7.5 7.0 6.5 6.0 5.5 5.0 4.5 4.0 3.5 3.0 2.5 2.0 1.5 1.0 0.5 0.0
δ (ppm)

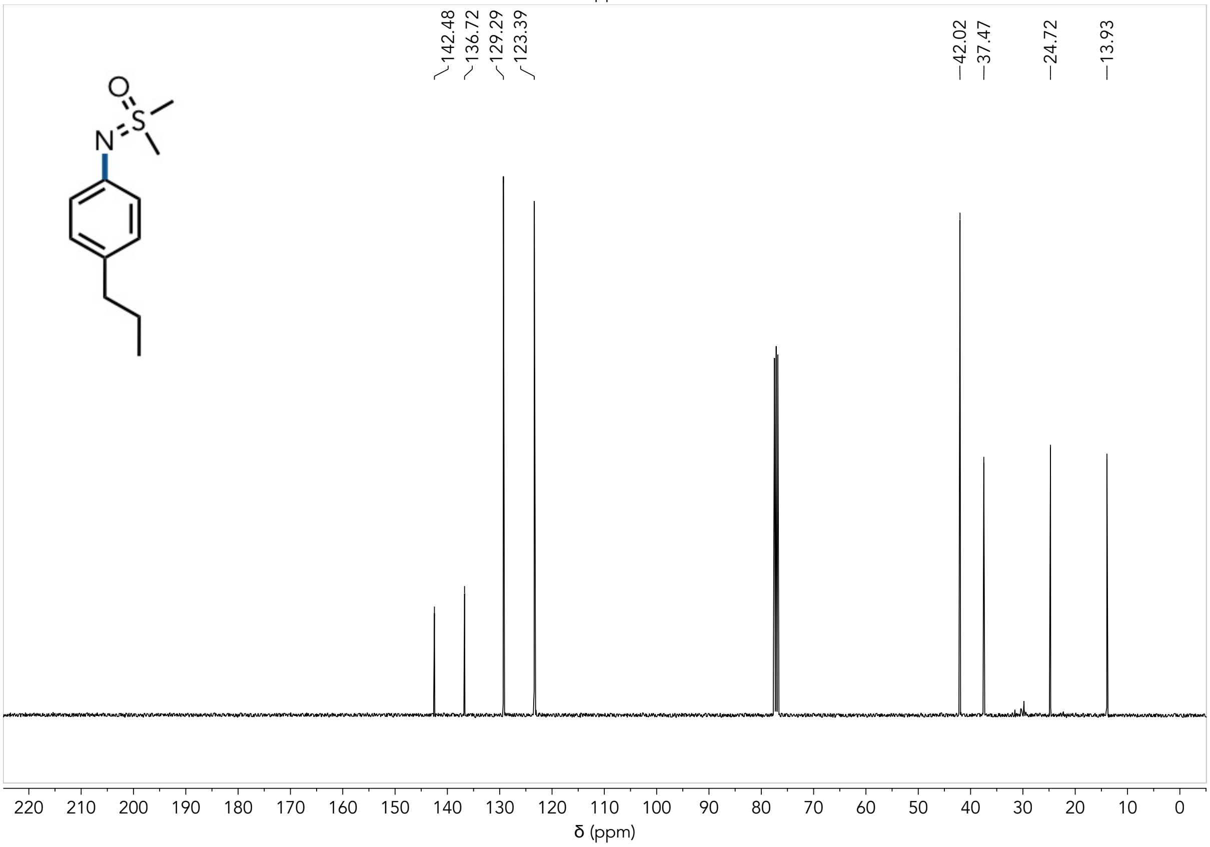
142.48
136.72
129.29
123.39
42.02
37.47
24.72
13.93
N
S
O
220 210 200 190 180 170 160 150 140 130 120 110 100 90 80 70 60 50 40 30 20 10 0
δ (ppm)

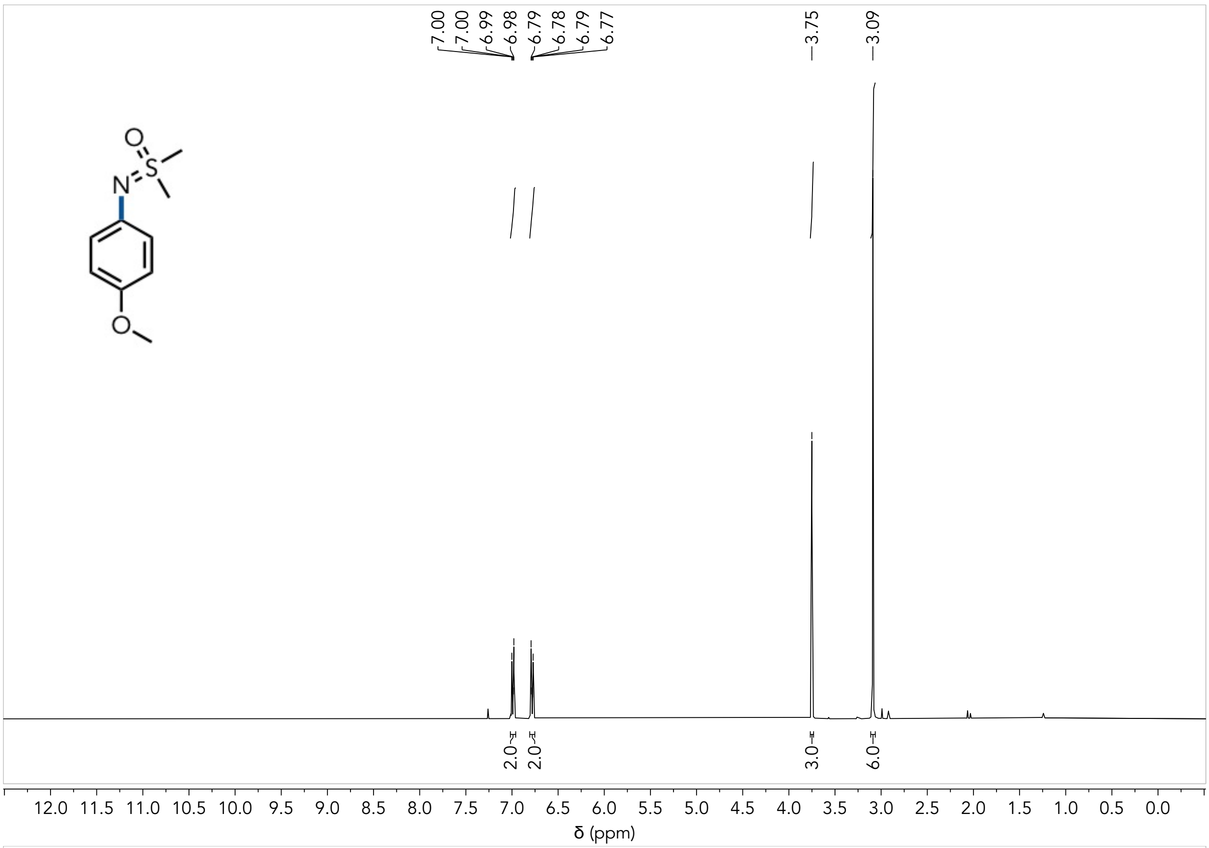
7.00
7.00
6.99
6.98
6.79
6.78
6.79
6.77
3.75
3.09
O
N
S
O
2.0
2.0
3.0
6.0
12.0 11.5 11.0 10.5 10.0 9.5 9.0 8.5 8.0 7.5 7.0 6.5 6.0 5.5 5.0 4.5 4.0 3.5 3.0 2.5 2.0 1.5 1.0 0.5 0.0
δ (ppm)
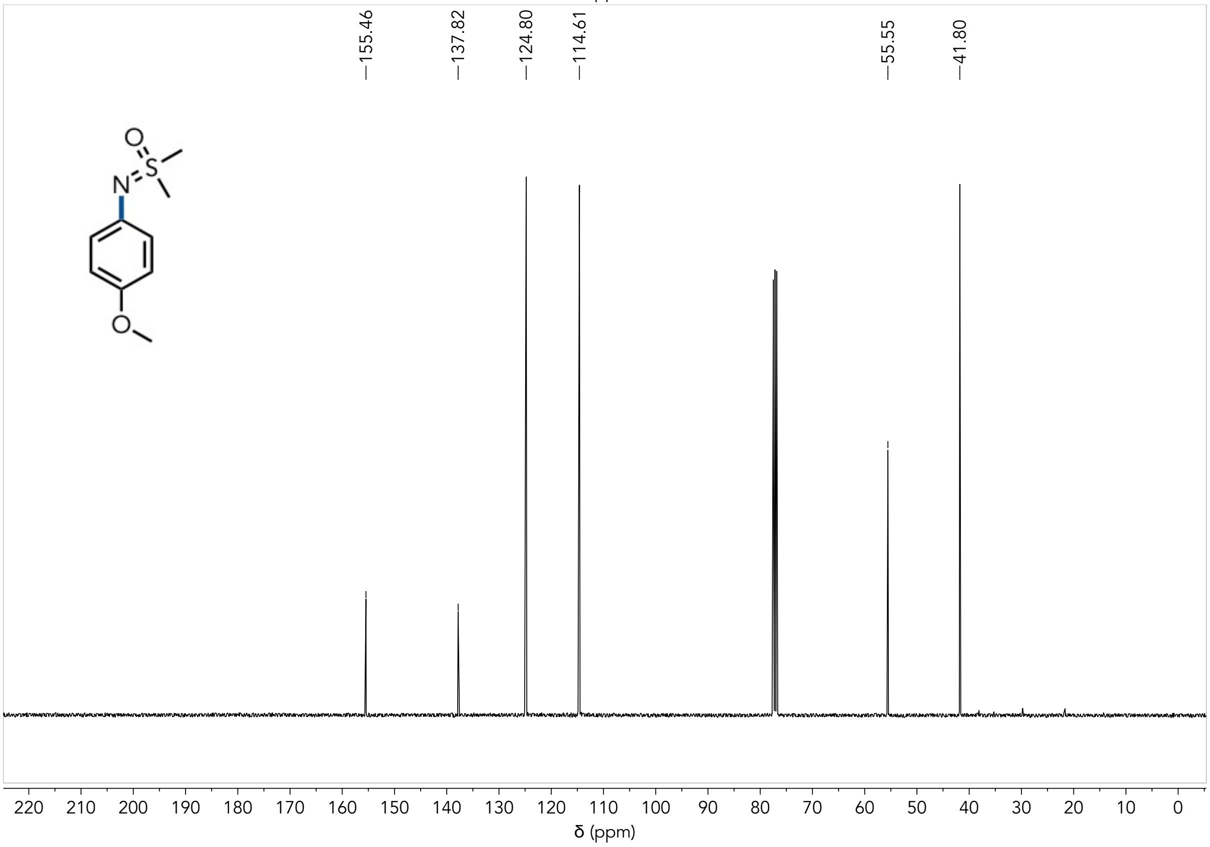
155.46
137.82
124.80
114.61
55.55
41.80
O
N
S
O
220 210 200 190 180 170 160 150 140 130 120 110 100 90 80 70 60 50 40 30 20 10 0
δ (ppm)

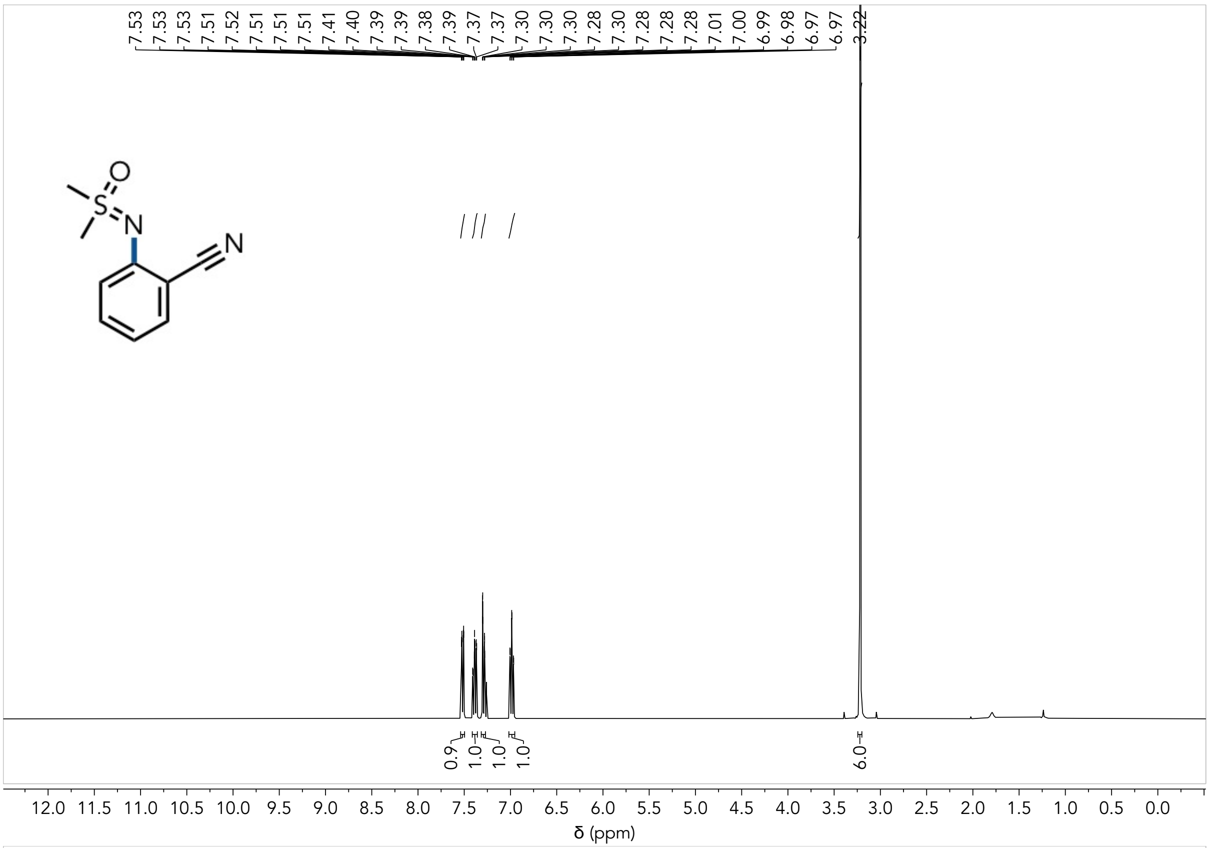

7.53
7.53
7.53
7.51
7.52
7.51
7.51
7.51
7.41
7.40
7.39
7.39
7.38
7.39
7.37
7.37
7.30
7.30
7.30
7.28
7.30
7.28
7.28
7.28
7.01
7.00
6.99
6.98
6.97
6.97
3.22
O
S
N
N
0.9
1.0
1.0
1.0
6.0
12.0 11.5 11.0 10.5 10.0 9.5 9.0 8.5 8.0 7.5 7.0 6.5 6.0 5.5 5.0 4.5 4.0 3.5 3.0 2.5 2.0 1.5 1.0 0.5 0.0
δ (ppm)

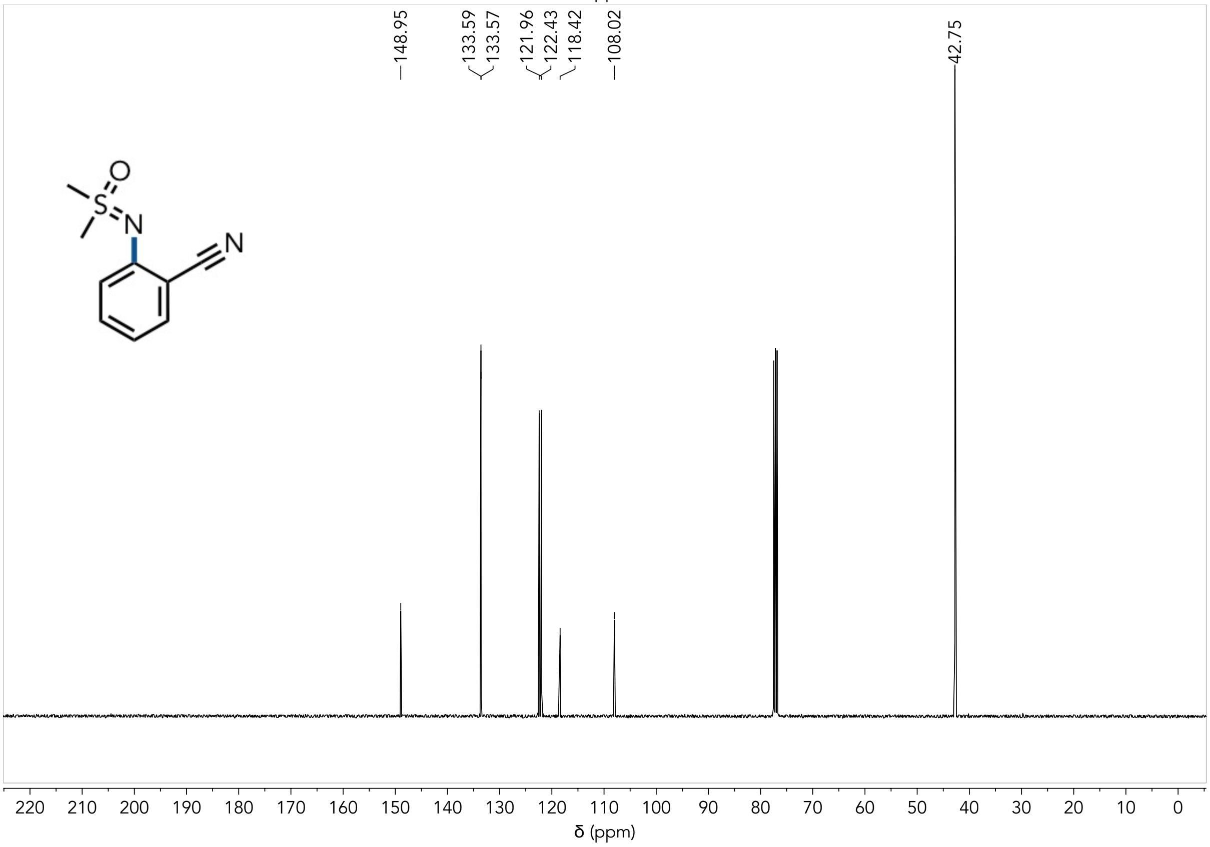

148.95
133.59
133.57
121.96
122.43
118.42
108.02
42.75
O
S
N
N
220 210 200 190 180 170 160 150 140 130 120 110 100 90 80 70 60 50 40 30 20 10 0
δ (ppm)

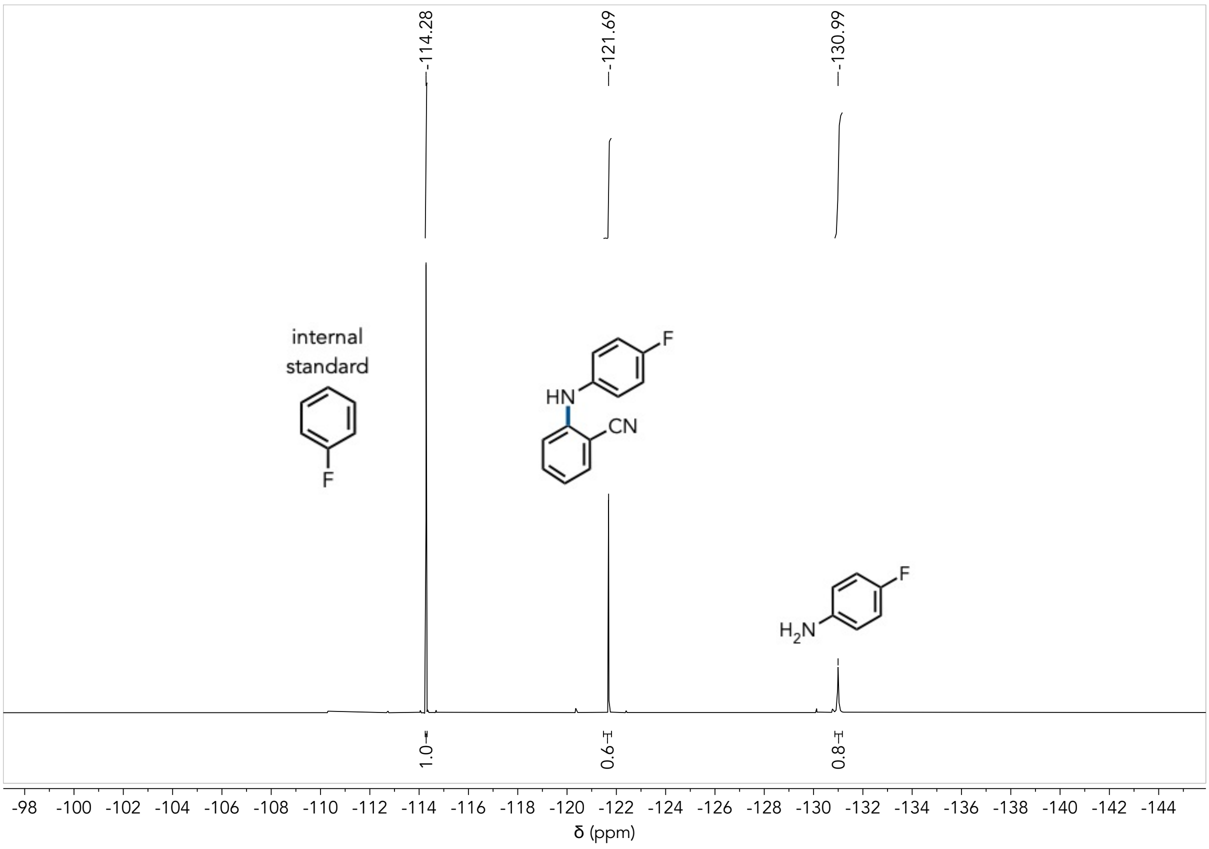
-114.28
-121.69
-130.99
internal
standard
F
HN
F
CN
F
H2N
1.0
0.6
0.8
-98 -100 -102 -104 -106 -108 -110 -112 -114 -116 -118 -120 -122 -124 -126 -128 -130 -132 -134 -136 -138 -140 -142 -144
δ (ppm)

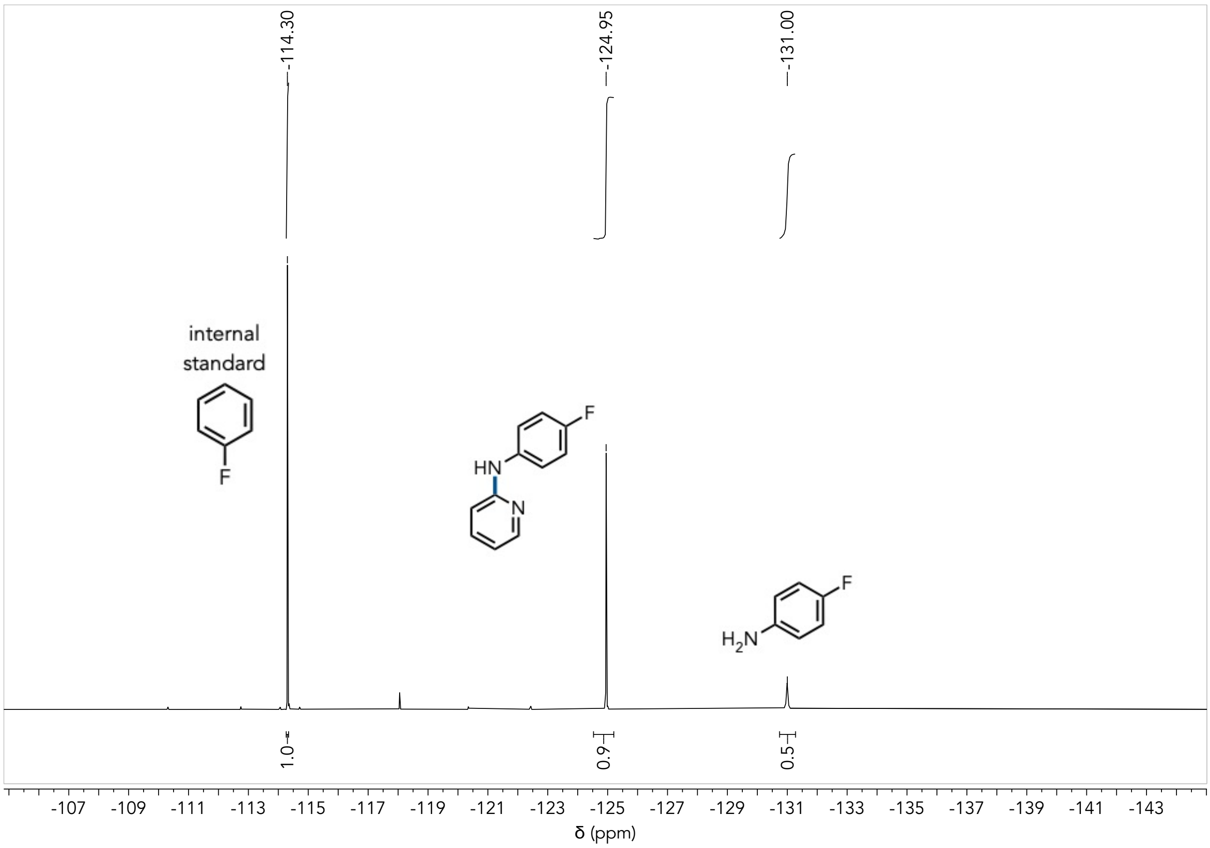
-114.30
-124.95
-131.00
internal
standard
F
HN
N
F
H2N
F
1.0
0.9
0.5
-107 -109 -111 -113 -115 -117 -119 -121 -123 -125 -127 -129 -131 -133 -135 -137 -139 -141 -143
δ (ppm)

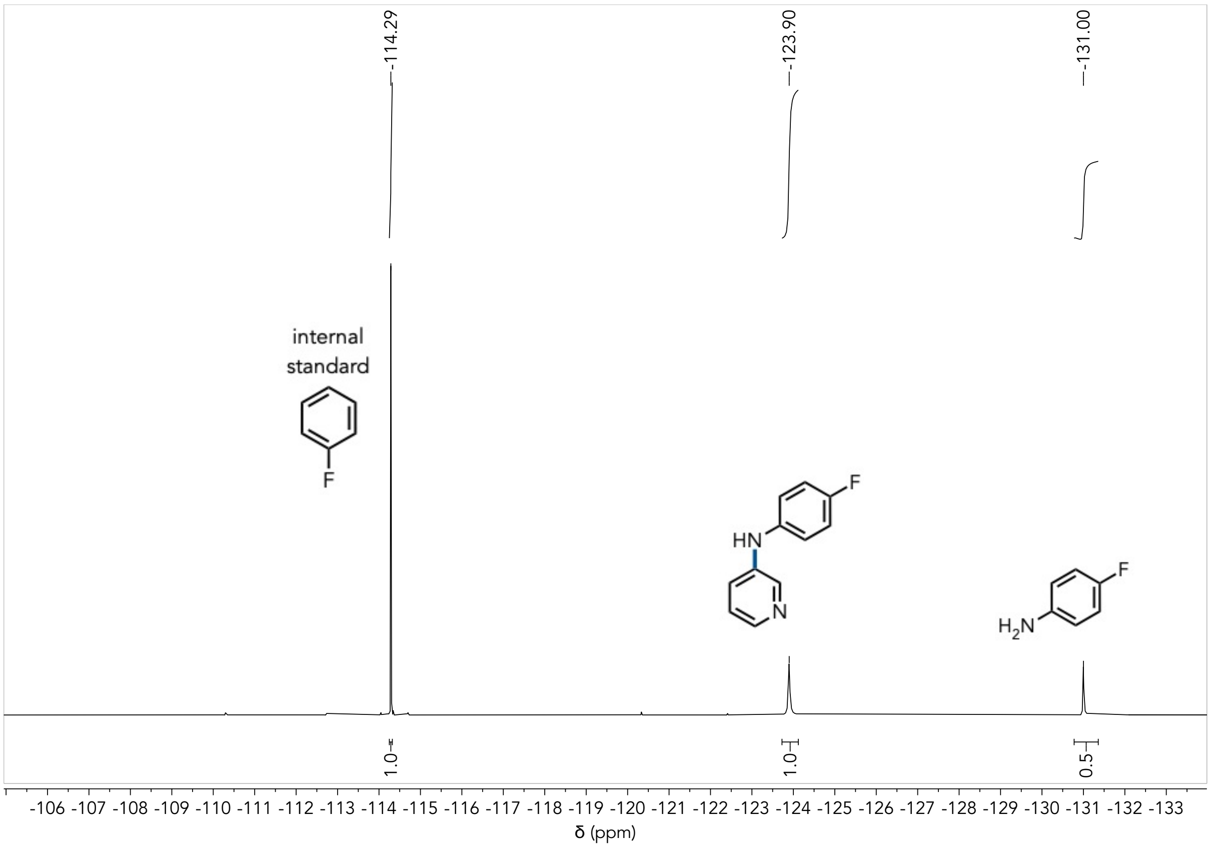
-114.29
-123.90
-131.00
internal
standard
F
HN
N
F
H2N
F
1.0
1.0
0.5
-106 -107 -108 -109 -110 -111 -112 -113 -114 -115 -116 -117 -118 -119 -120 -121 -122 -123 -124 -125 -126 -127 -128 -129 -130 -131 -132 -133
δ (ppm)

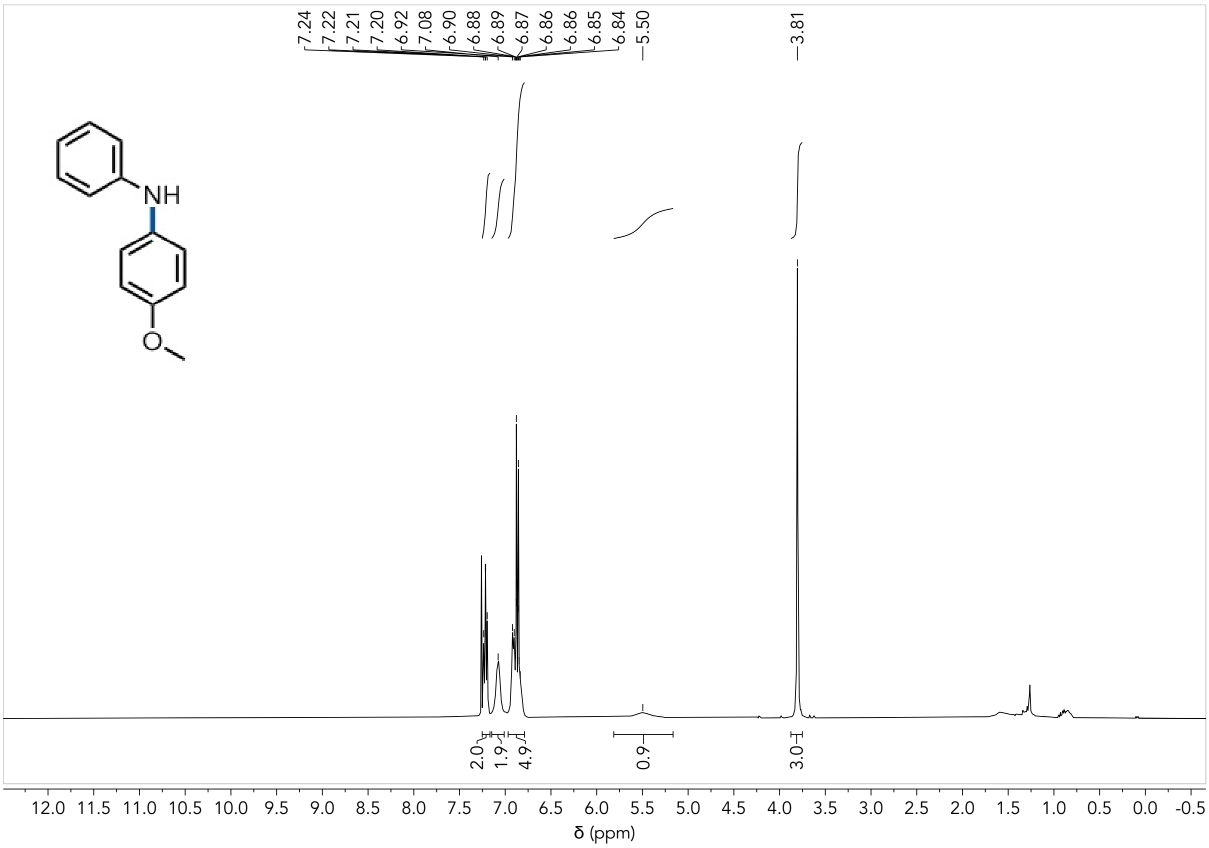
7.24
7.22
7.21
7.20
6.92
7.08
6.90
6.88
6.89
6.87
6.86
6.86
6.85
6.84
5.50
3.81
NH
2.0
1.9
4.9
0.9
3.0
12.0 11.5 11.0 10.5 10.0 9.5 9.0 8.5 8.0 7.5 7.0 6.5 6.0 5.5 5.0 4.5 4.0 3.5 3.0 2.5 2.0 1.5 1.0 0.5 0.0 -0.5
δ (ppm)

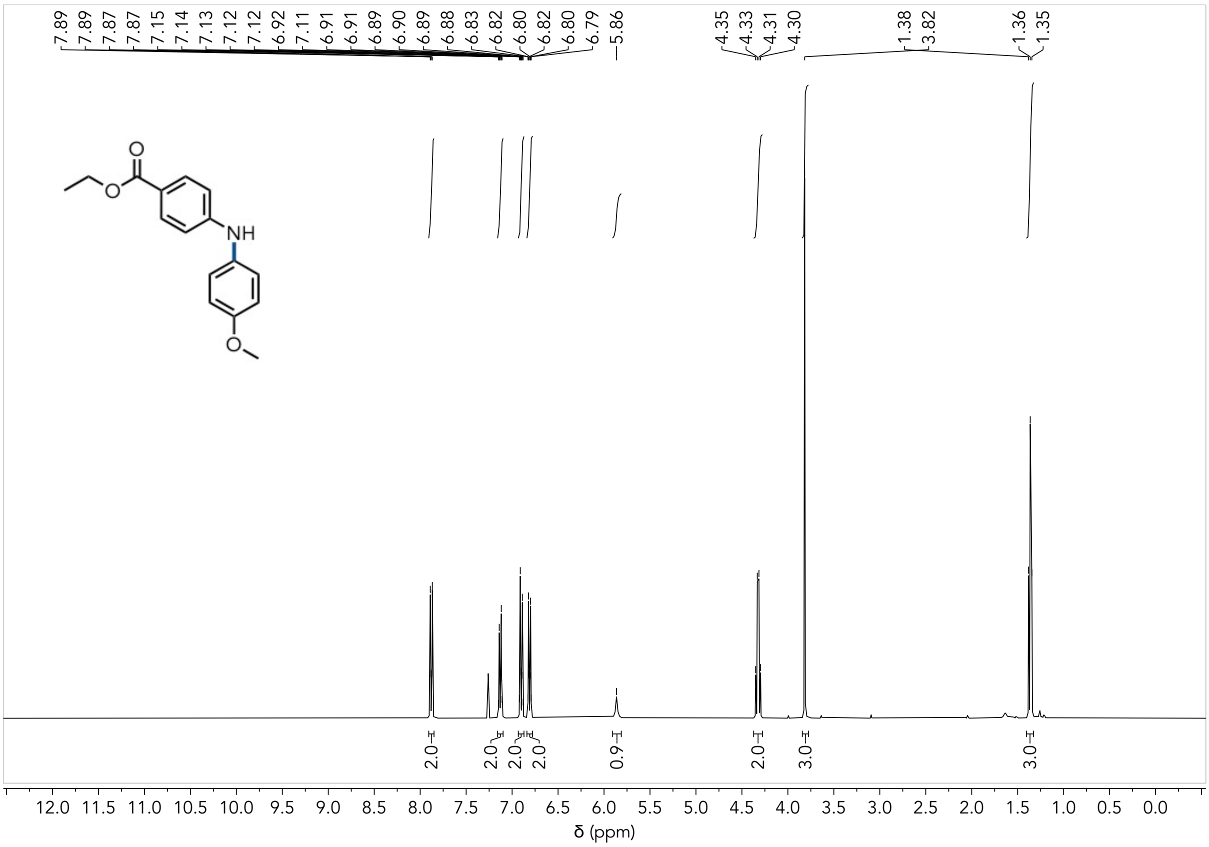
7.89
7.89
7.87
7.87
7.15
7.14
7.13
7.12
7.12
6.92
7.11
6.91
6.91
6.89
6.90
6.89
6.88
6.83
6.82
6.80
6.82
6.80
6.79
5.86
4.35
4.33
4.31
4.30
1.38
3.82
1.36
1.35
NH
2.0
2.0
2.0
2.0
0.9
2.0
3.0
3.0
12.0 11.5 11.0 10.5 10.0 9.5 9.0 8.5 8.0 7.5 7.0 6.5 6.0 5.5 5.0 4.5 4.0 3.5 3.0 2.5 2.0 1.5 1.0 0.5 0.0
δ (ppm)

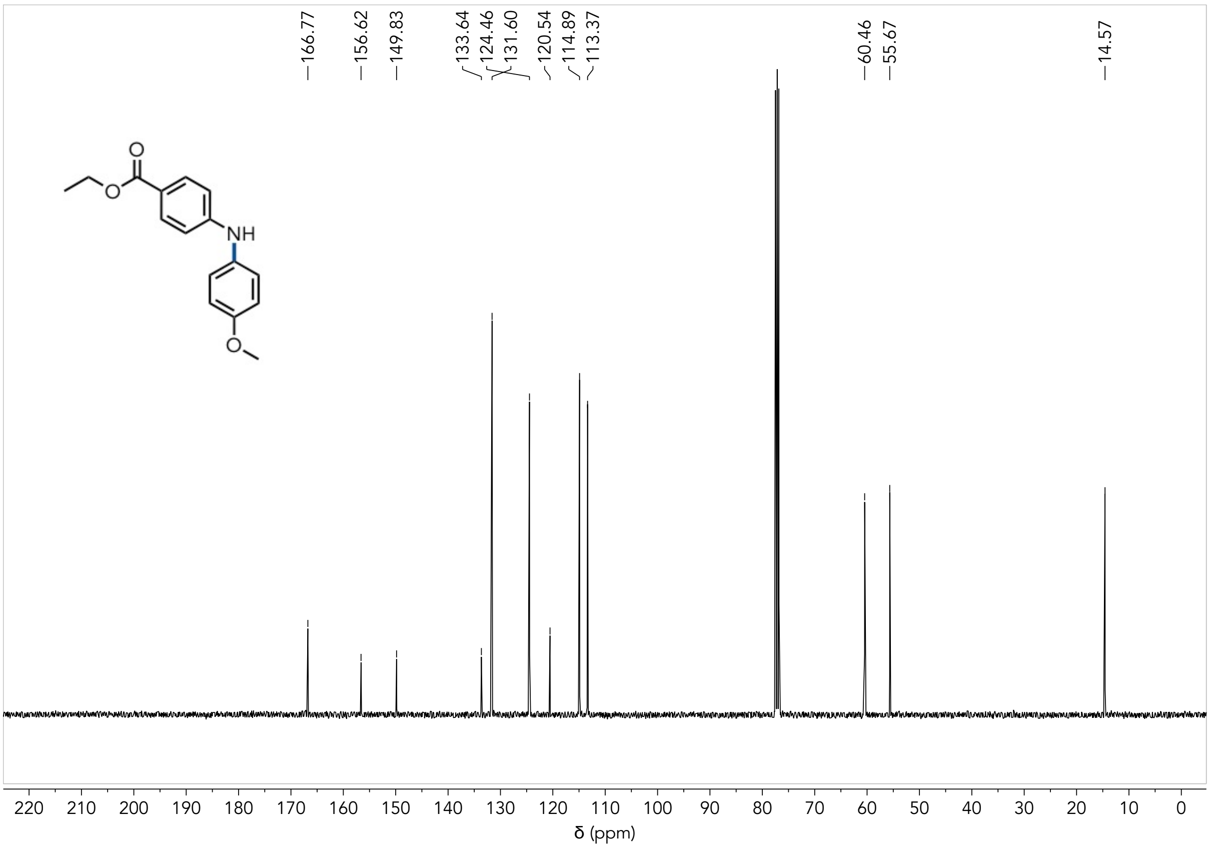
166.77
156.62
149.83
133.64
124.46
131.60
120.54
114.89
113.37
60.46
55.67
14.57
NH
O
220 210 200 190 180 170 160 150 140 130 120 110 100 90 80 70 60 50 40 30 20 10 0
δ (ppm)

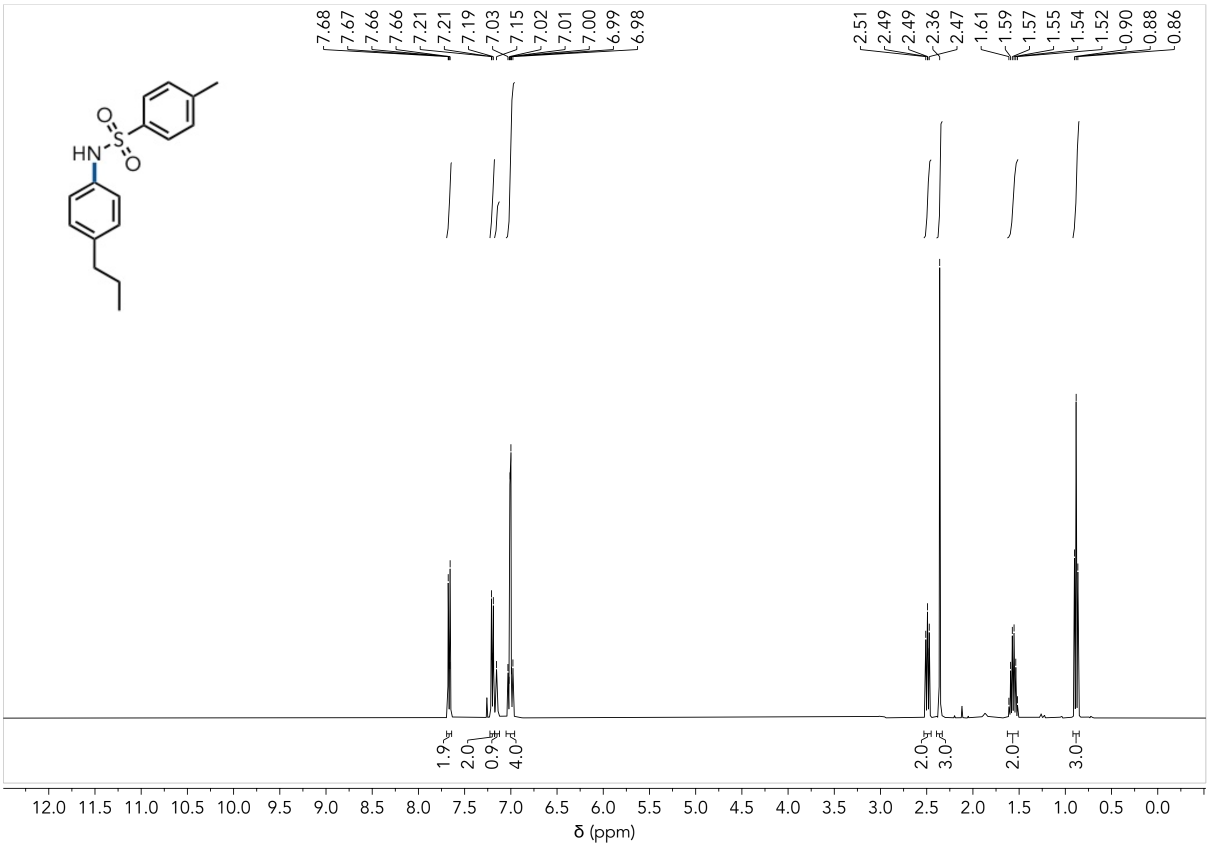
7.68
7.67
7.66
7.66
7.21
7.21
7.19
7.03
7.15
7.02
7.01
7.00
6.99
6.98
2.51
2.49
2.49
2.36
2.47
1.61
1.59
1.57
1.55
1.54
1.52
0.90
0.88
0.86
HN
S
O
1.9
2.0
0.9
4.0
2.0
3.0
2.0
3.0
12.0 11.5 11.0 10.5 10.0 9.5 9.0 8.5 8.0 7.5 7.0 6.5 6.0 5.5 5.0 4.5 4.0 3.5 3.0 2.5 2.0 1.5 1.0 0.5 0.0
δ (ppm)

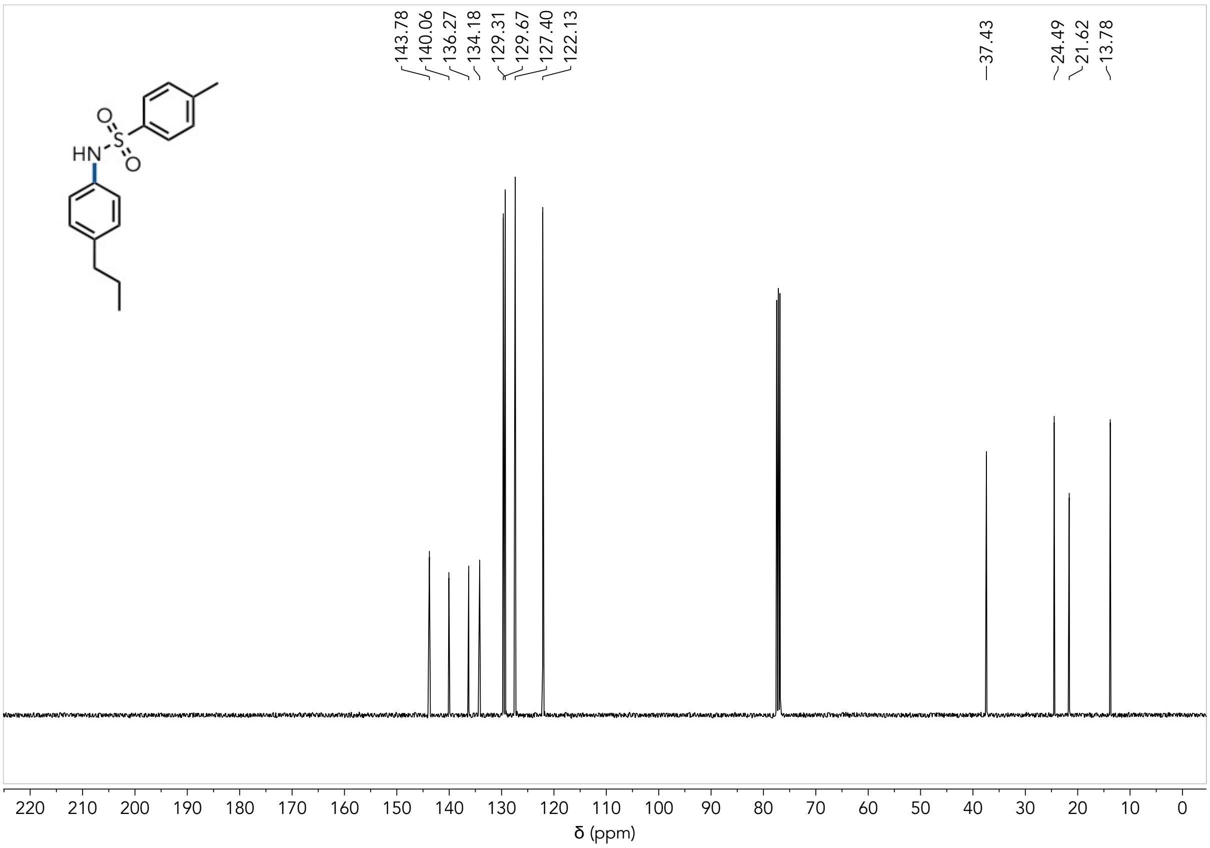
143.78
140.06
136.27
134.18
129.31
129.67
127.40
122.13
37.43
24.49
21.62
13.78
HN
S
O
O
220 210 200 190 180 170 160 150 140 130 120 110 100 90 80 70 60 50 40 30 20 10 0
δ (ppm)

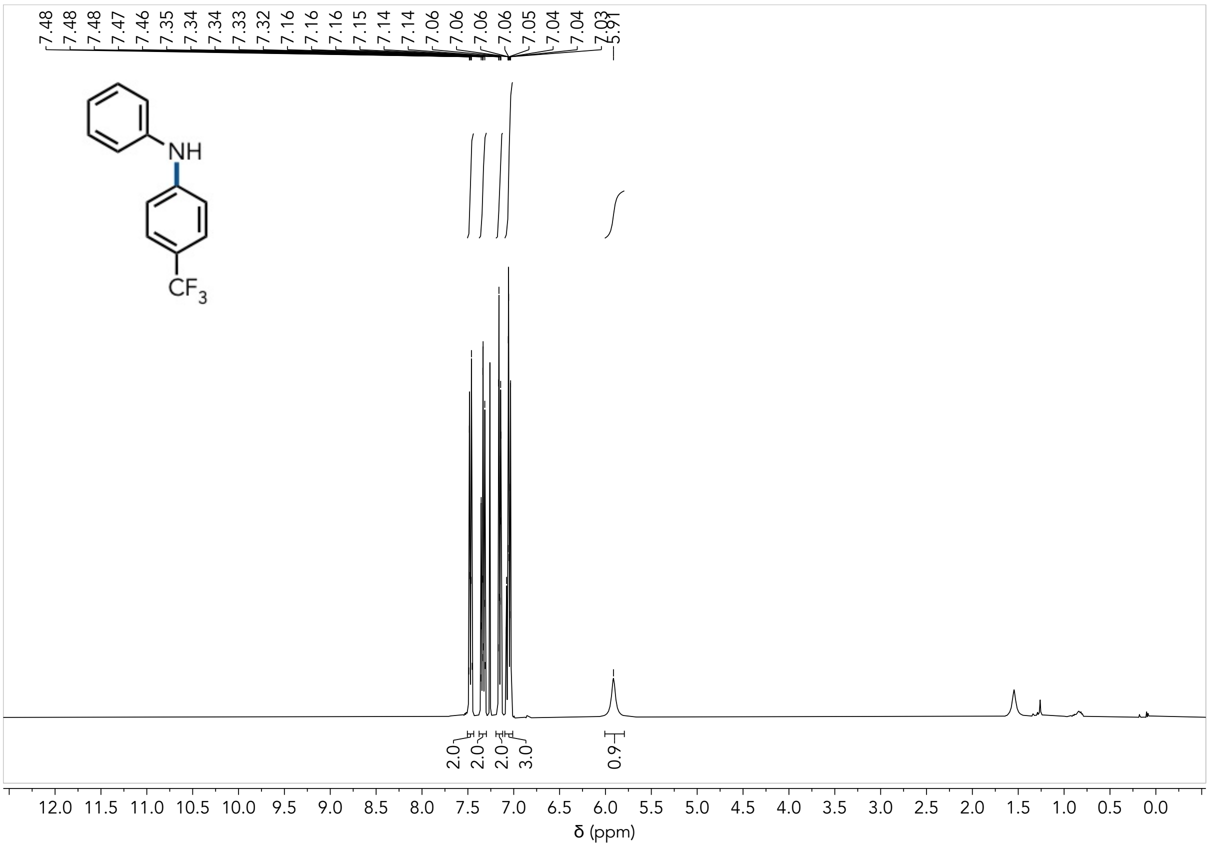
7.48
7.48
7.48
7.47
7.46
7.35
7.34
7.34
7.33
7.32
7.16
7.16
7.16
7.15
7.14
7.06
7.06
7.06
7.06
7.05
7.04
7.04
7.03
5.91
NH
CF3
2.0
2.0
2.0
3.0
0.9
12.0 11.5 11.0 10.5 10.0 9.5 9.0 8.5 8.0 7.5 7.0 6.5 6.0 5.5 5.0 4.5 4.0 3.5 3.0 2.5 2.0 1.5 1.0 0.5 0.0
δ (ppm)

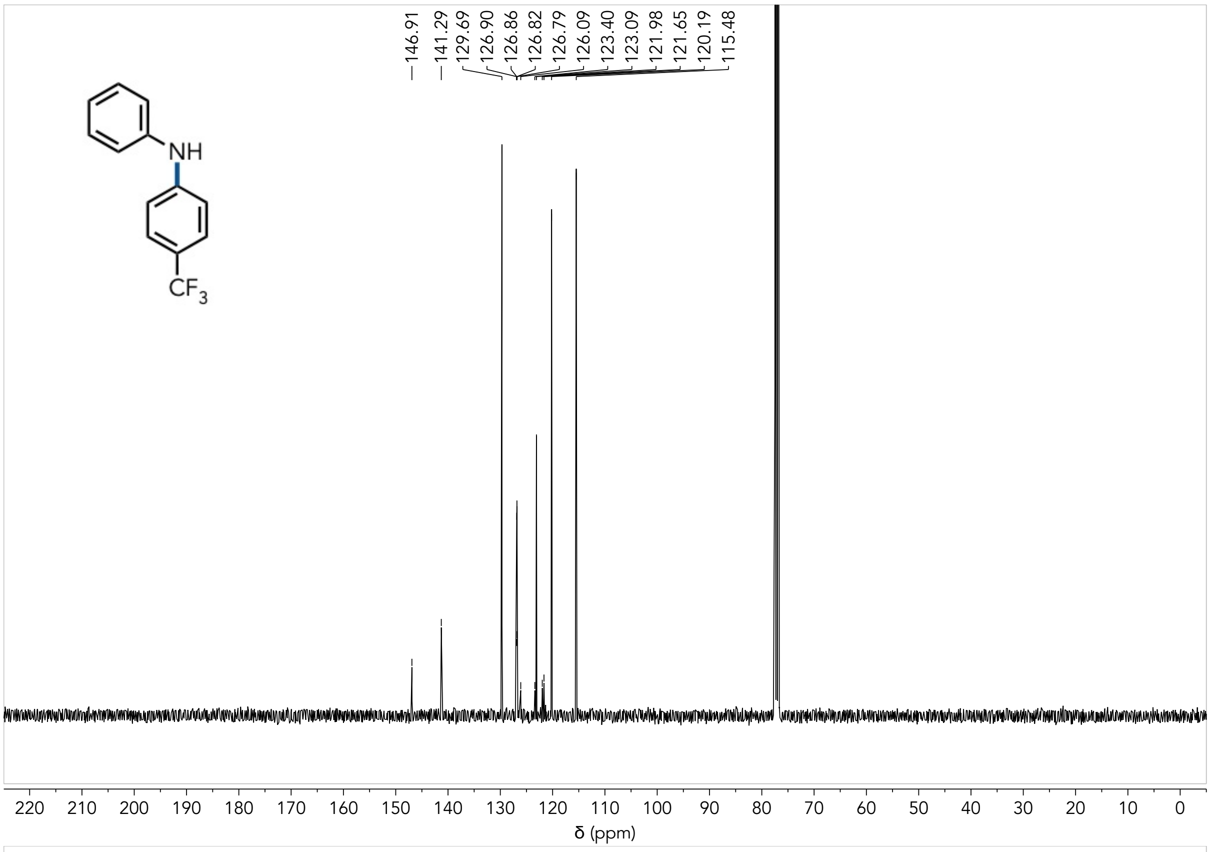
146.91
141.29
129.69
126.90
126.86
126.82
126.79
126.09
123.40
123.09
121.98
121.65
120.19
115.48
NH
CF3
220 210 200 190 180 170 160 150 140 130 120 110 100 90 80 70 60 50 40 30 20 10 0
δ (ppm)

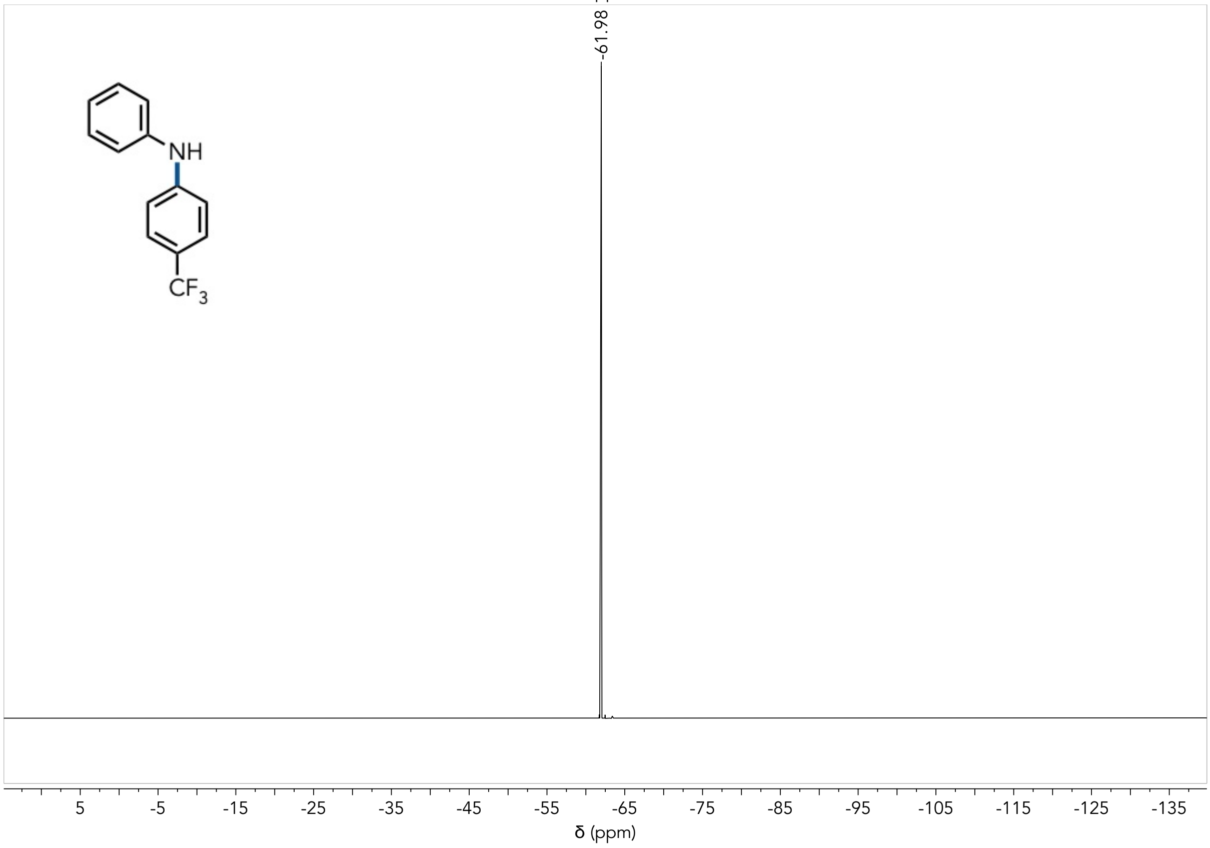
-61.98
NH
CF3
5 -5 -15 -25 -35 -45 -55 -65 -75 -85 -95 -105 -115 -125 -135
δ (ppm)

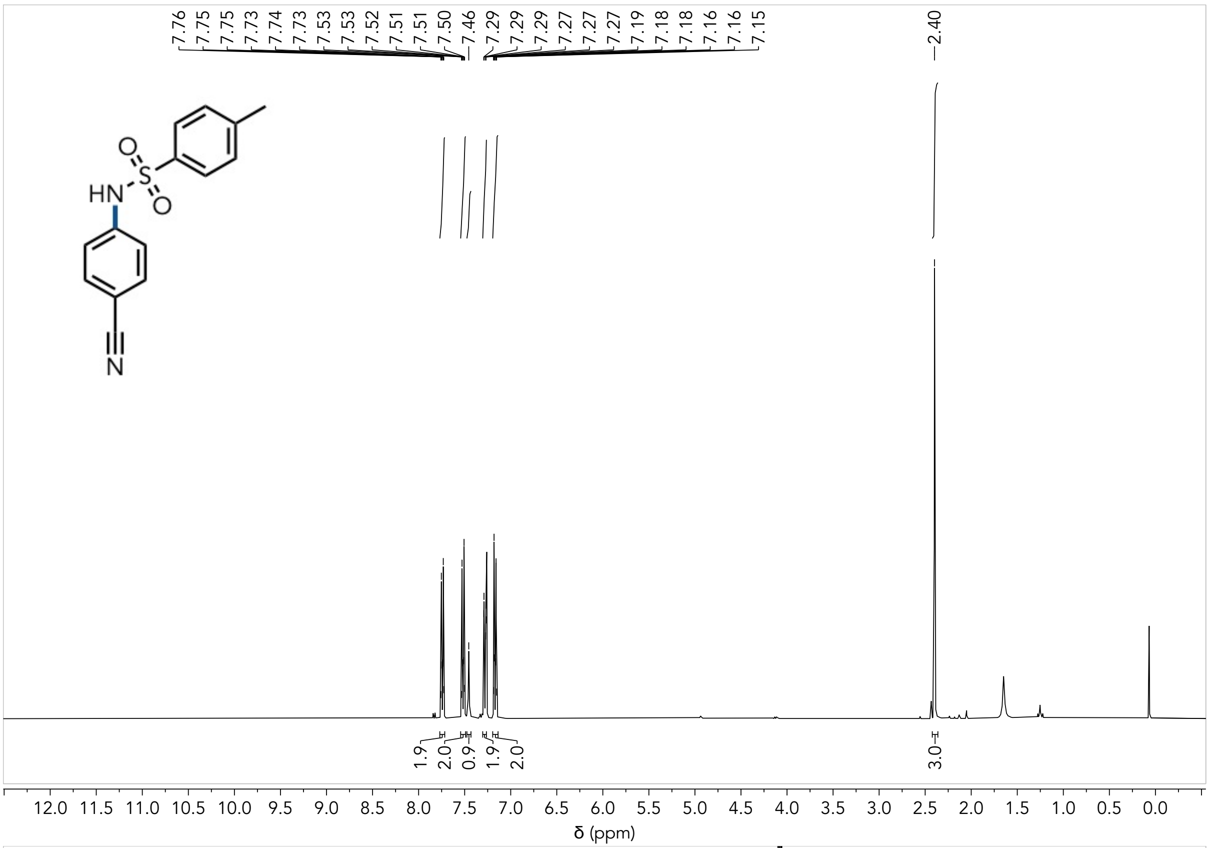
7.76
7.75
7.75
7.73
7.74
7.73
7.73
7.53
7.53
7.52
7.51
7.51
7.50
7.46
7.29
7.29
7.29
7.27
7.27
7.27
7.19
7.18
7.18
7.16
7.16
7.15
2.40
HN
S
O
O
N
1.9
2.0
0.9
1.9
2.0
3.0
12.0 11.5 11.0 10.5 10.0 9.5 9.0 8.5 8.0 7.5 7.0 6.5 6.0 5.5 5.0 4.5 4.0 3.5 3.0 2.5 2.0 1.5 1.0 0.5 0.0
δ (ppm)

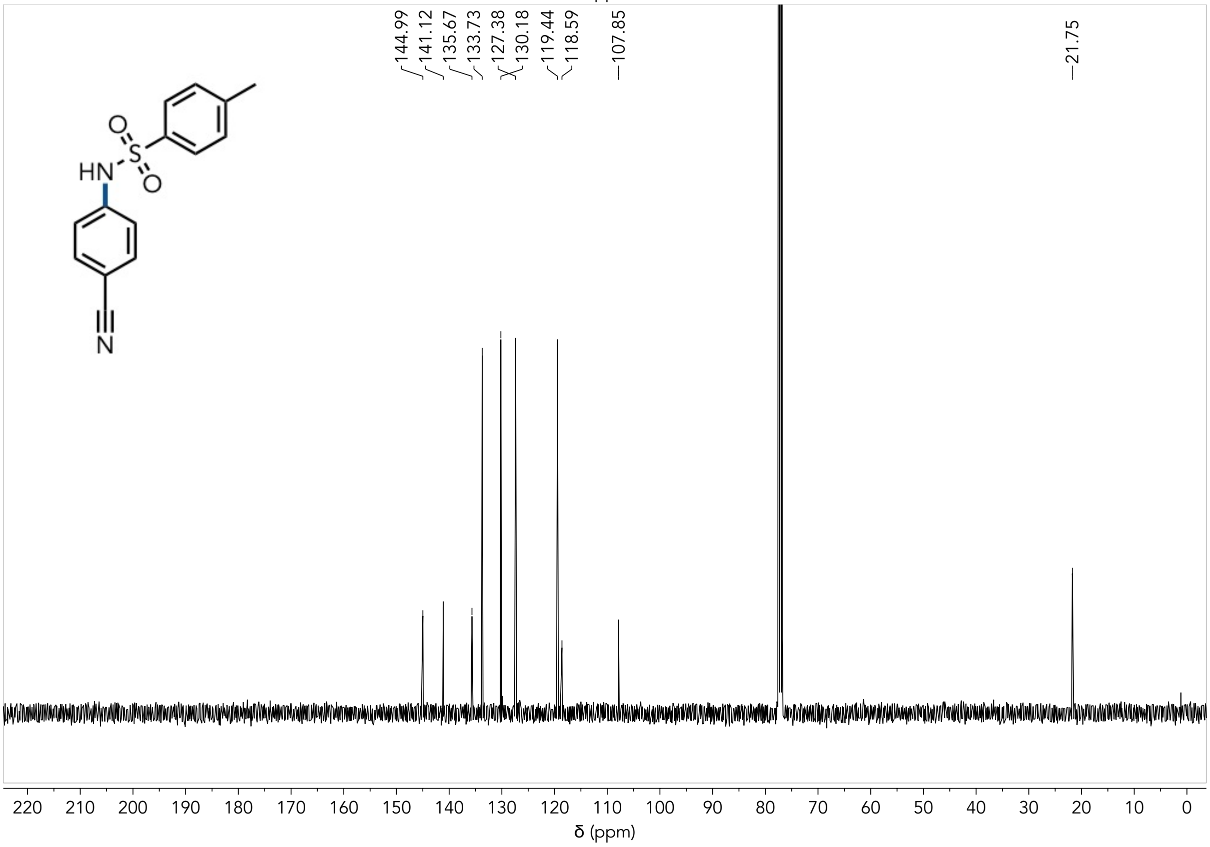
144.99
141.12
135.67
133.73
127.38
130.18
119.44
118.59
107.85
21.75
HN
S
O
O
N
220 210 200 190 180 170 160 150 140 130 120 110 100 90 80 70 60 50 40 30 20 10 0
δ (ppm)

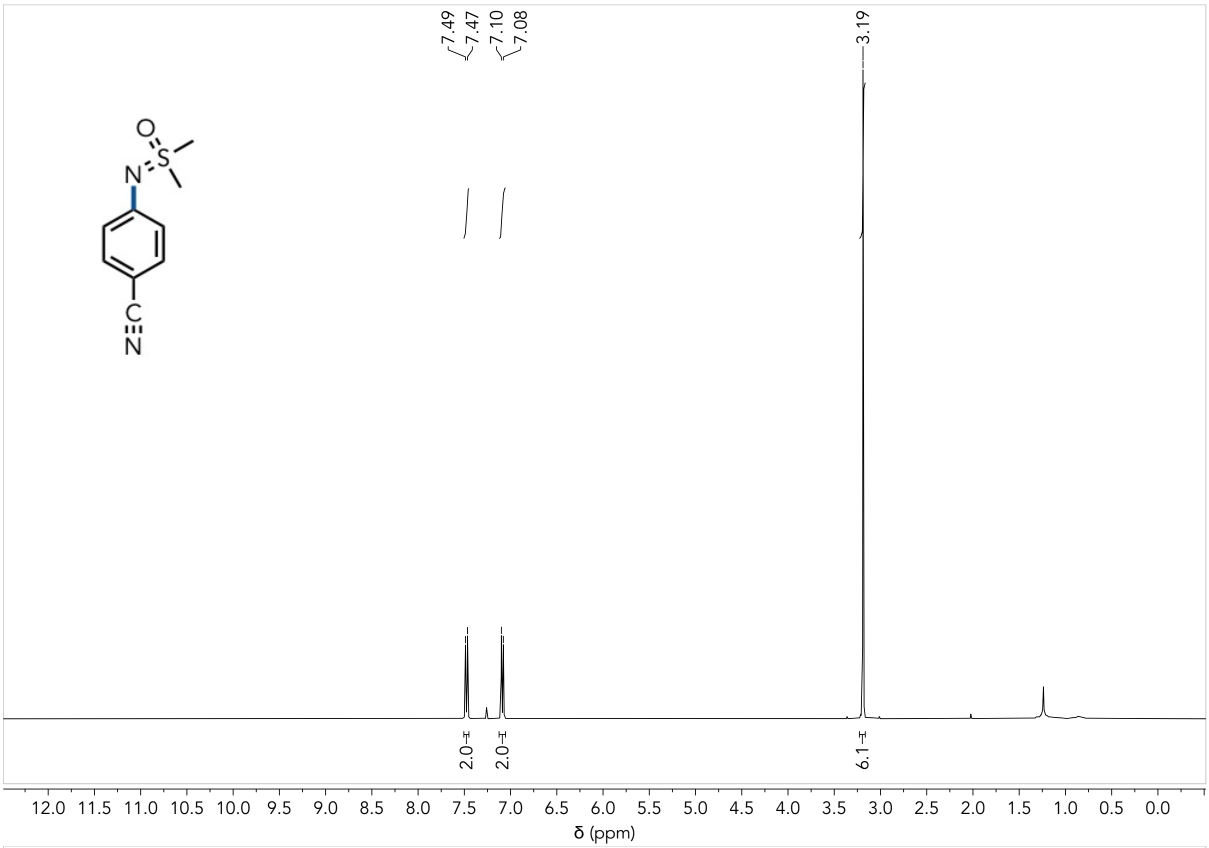
7.49
7.47
7.10
7.08
3.19
2.0
2.0
6.1
12.0 11.5 11.0 10.5 10.0 9.5 9.0 8.5 8.0 7.5 7.0 6.5 6.0 5.5 5.0 4.5 4.0 3.5 3.0 2.5 2.0 1.5 1.0 0.5 0.0
δ (ppm)

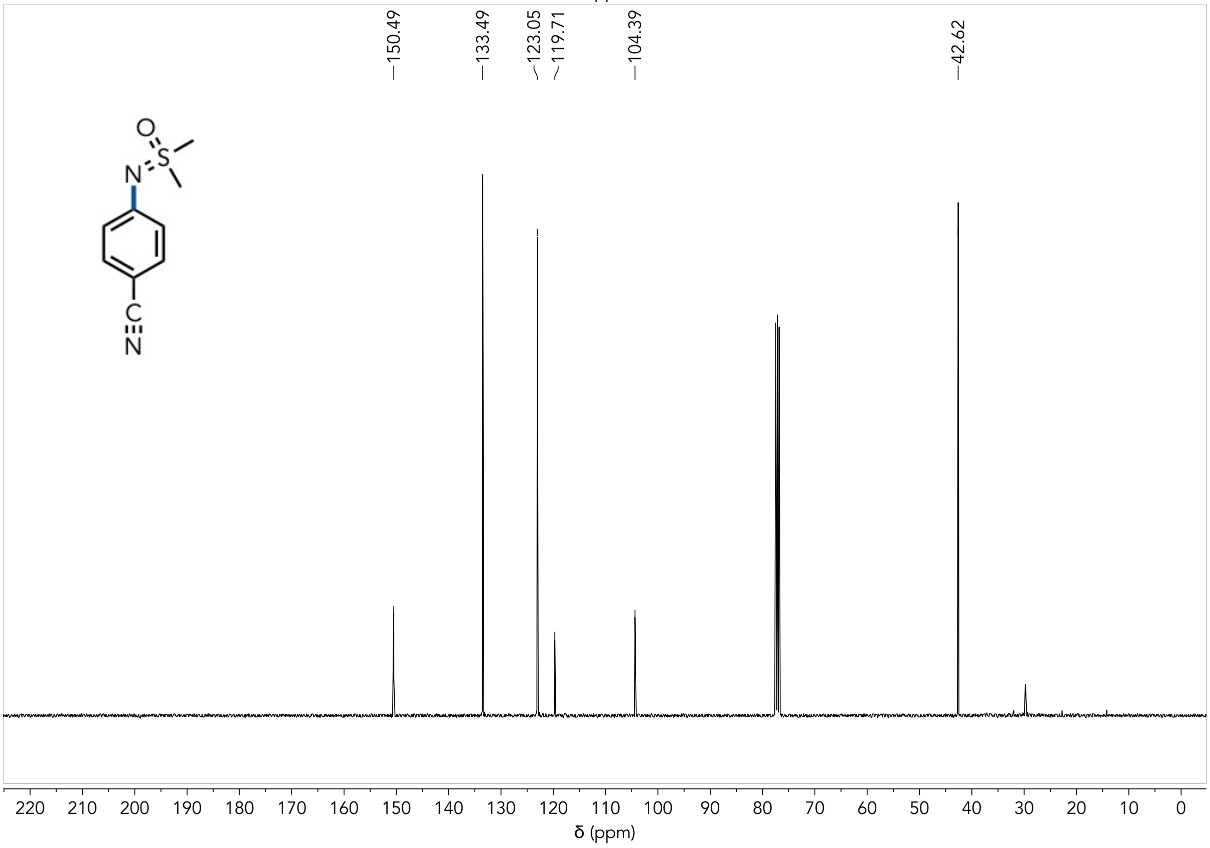
150.49
133.49
123.05
119.71
104.39
42.62
220 210 200 190 180 170 160 150 140 130 120 110 100 90 80 70 60 50 40 30 20 10 0
δ (ppm)

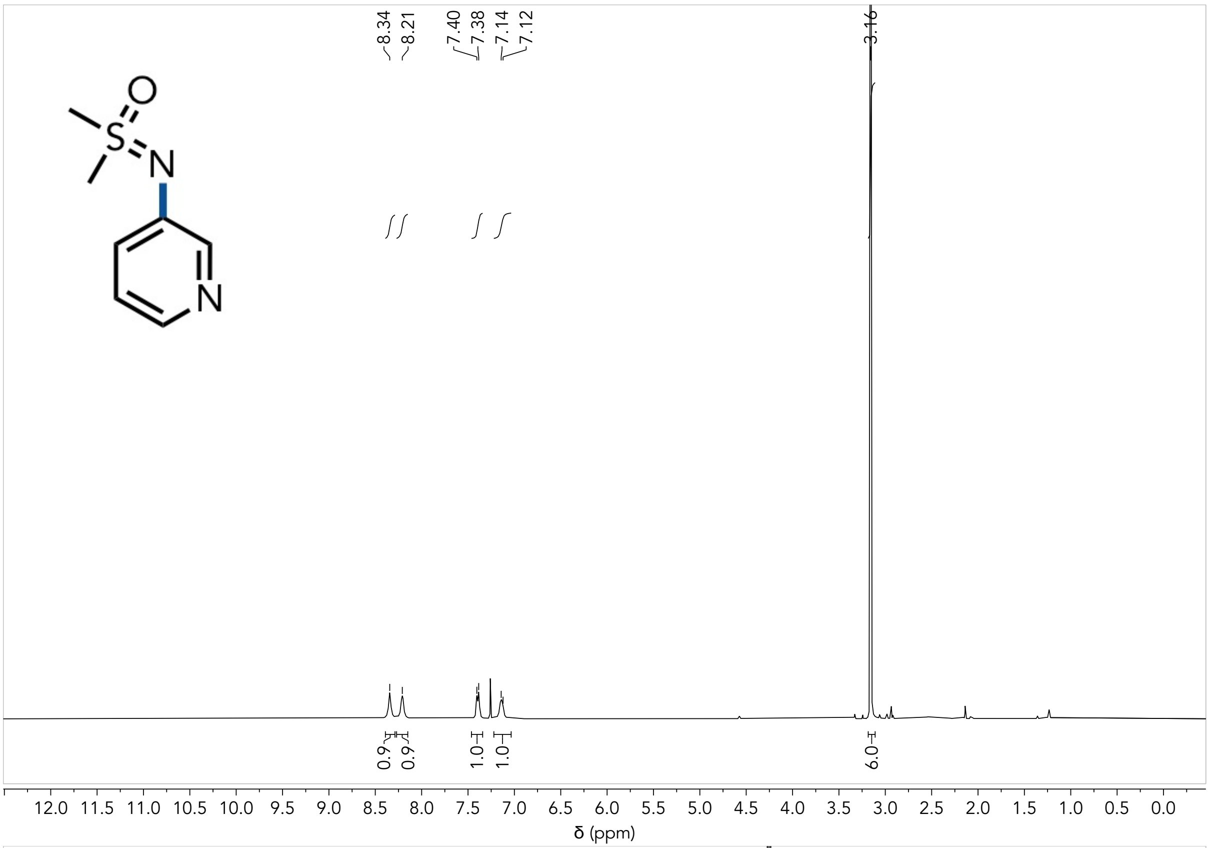

8.34
8.21
7.40
7.38
7.14
7.12
3.16
0.9
0.9
1.0
1.0
6.0
12.0 11.5 11.0 10.5 10.0 9.5 9.0 8.5 8.0 7.5 7.0 6.5 6.0 5.5 5.0 4.5 4.0 3.5 3.0 2.5 2.0 1.5 1.0 0.5 0.0
δ (ppm)

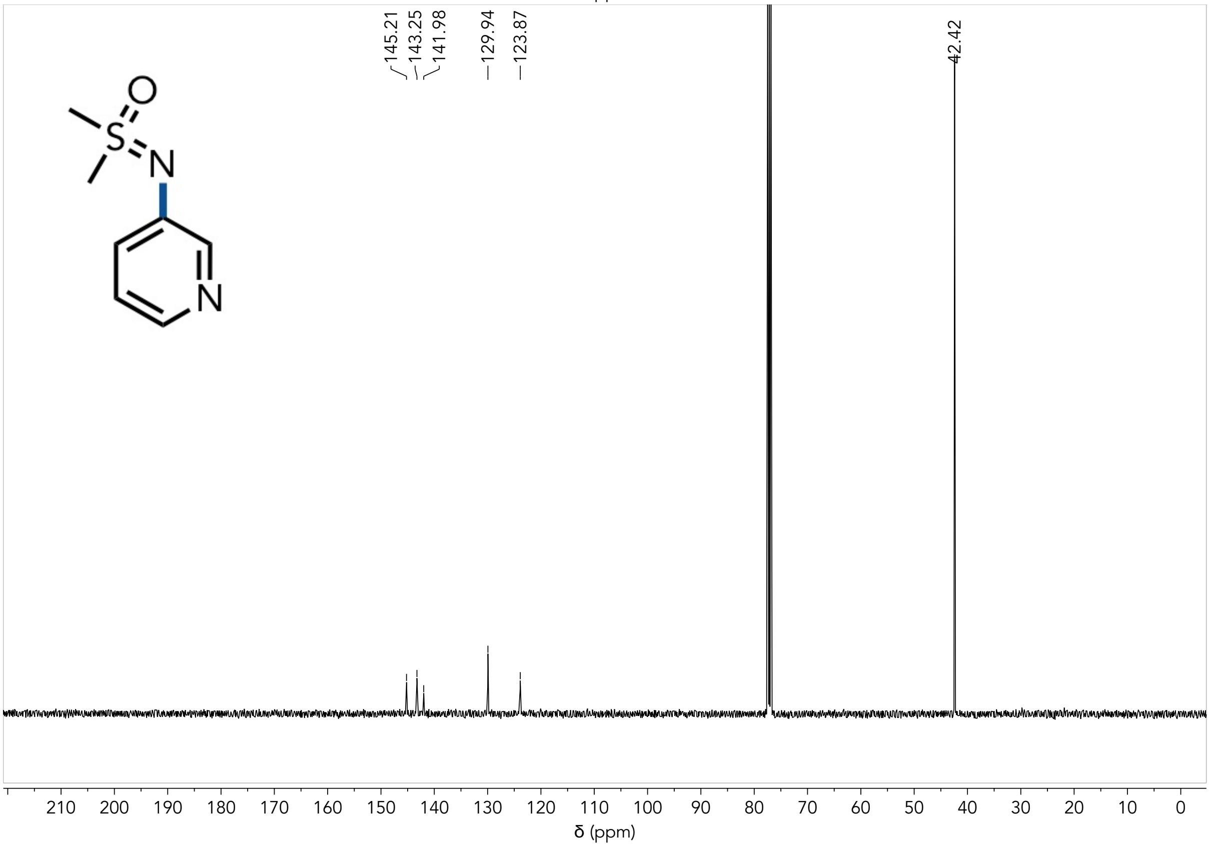

145.21
143.25
141.98
129.94
123.87
42.42
210 200 190 180 170 160 150 140 130 120 110 100 90 80 70 60 50 40 30 20 10 0
δ (ppm)

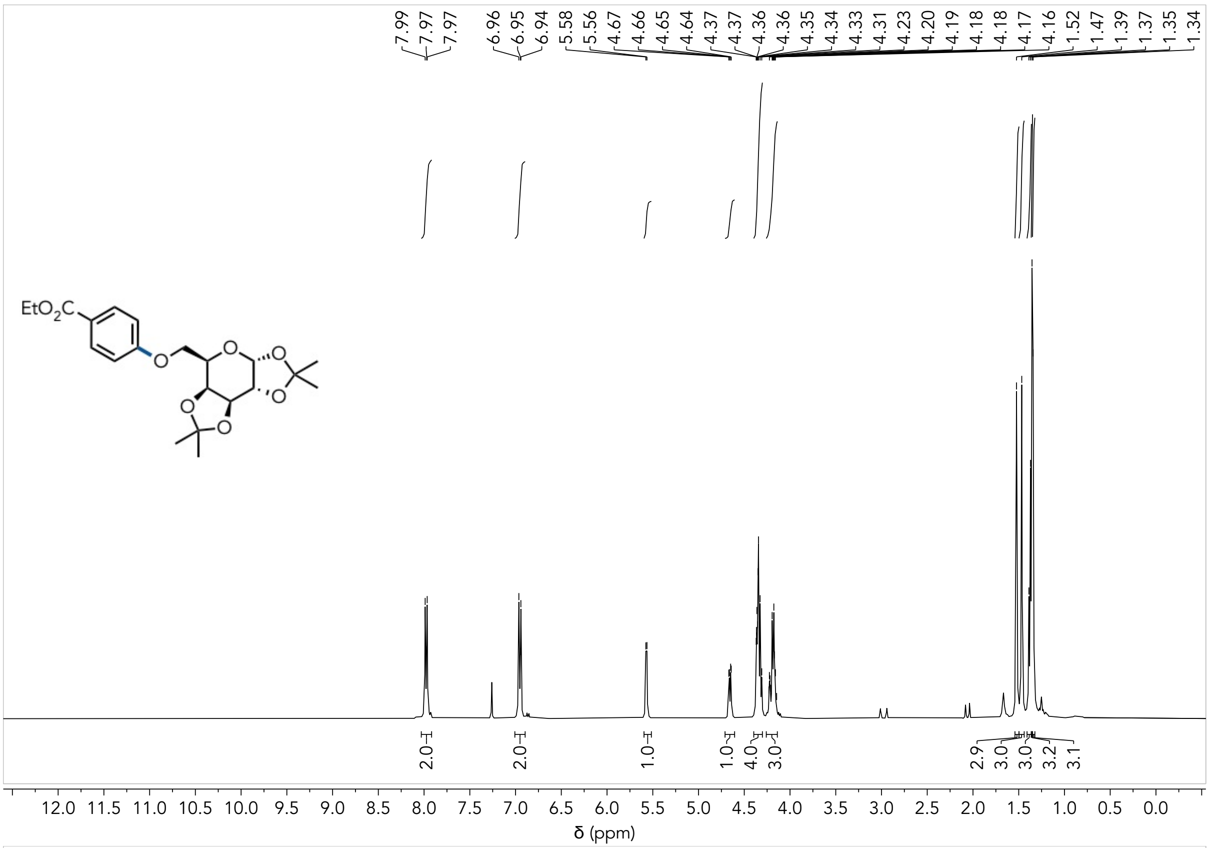
7.99 7.97 7.97 6.96 6.95 6.94 5.58 5.56 4.67 4.66 4.65 4.64 4.37 4.37 4.36 4.36 4.35 4.34 4.33 4.31 4.23 4.20 4.19 4.18 4.18 4.17 4.16 1.52 1.47 1.39 1.37 1.35 1.34
EtO2C
O
2.0 2.0 1.0 1.0 4.0 3.0 2.9 3.0 3.0 3.2 3.1
12.0 11.5 11.0 10.5 10.0 9.5 9.0 8.5 8.0 7.5 7.0 6.5 6.0 5.5 5.0 4.5 4.0 3.5 3.0 2.5 2.0 1.5 1.0 0.5 0.0
δ (ppm)

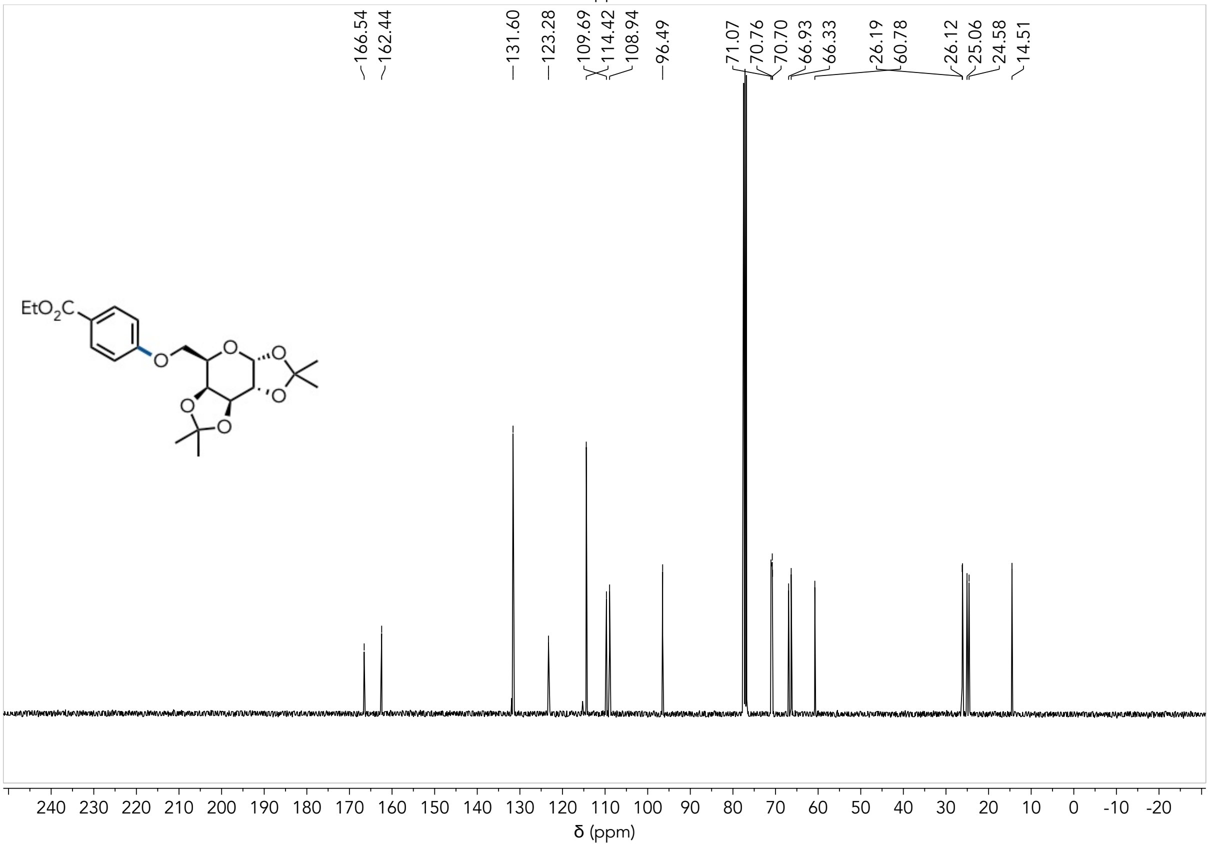
166.54 162.44 131.60 123.28 109.69 114.42 108.94 96.49 71.07 70.76 70.70 66.93 66.33 26.19 60.78 26.12 25.06 24.58 14.51
EtO2C
O
240 230 220 210 200 190 180 170 160 150 140 130 120 110 100 90 80 70 60 50 40 30 20 10 0 -10 -20
δ (ppm)

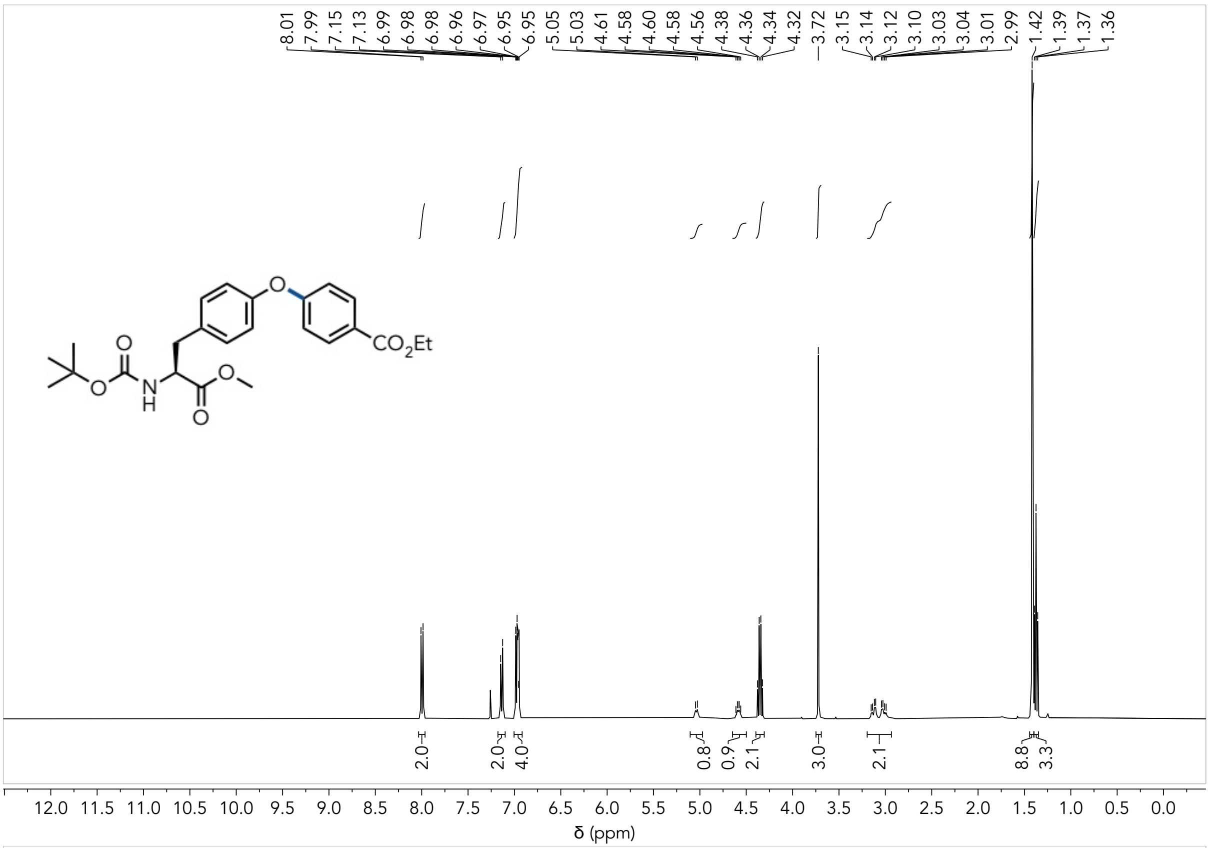
8.01
7.99
7.15
7.13
6.99
6.98
6.96
6.97
6.95
6.95
5.05
5.03
4.61
4.58
4.60
4.58
4.56
4.38
4.36
4.34
4.32
3.72
3.15
3.14
3.12
3.10
3.03
3.04
3.01
2.99
1.42
1.39
1.37
1.36
CO2Et
2.0
2.0
4.0
0.8
0.9
2.1
3.0
2.1
8.8
3.3
12.0 11.5 11.0 10.5 10.0 9.5 9.0 8.5 8.0 7.5 7.0 6.5 6.0 5.5 5.0 4.5 4.0 3.5 3.0 2.5 2.0 1.5 1.0 0.5 0.0
δ (ppm)

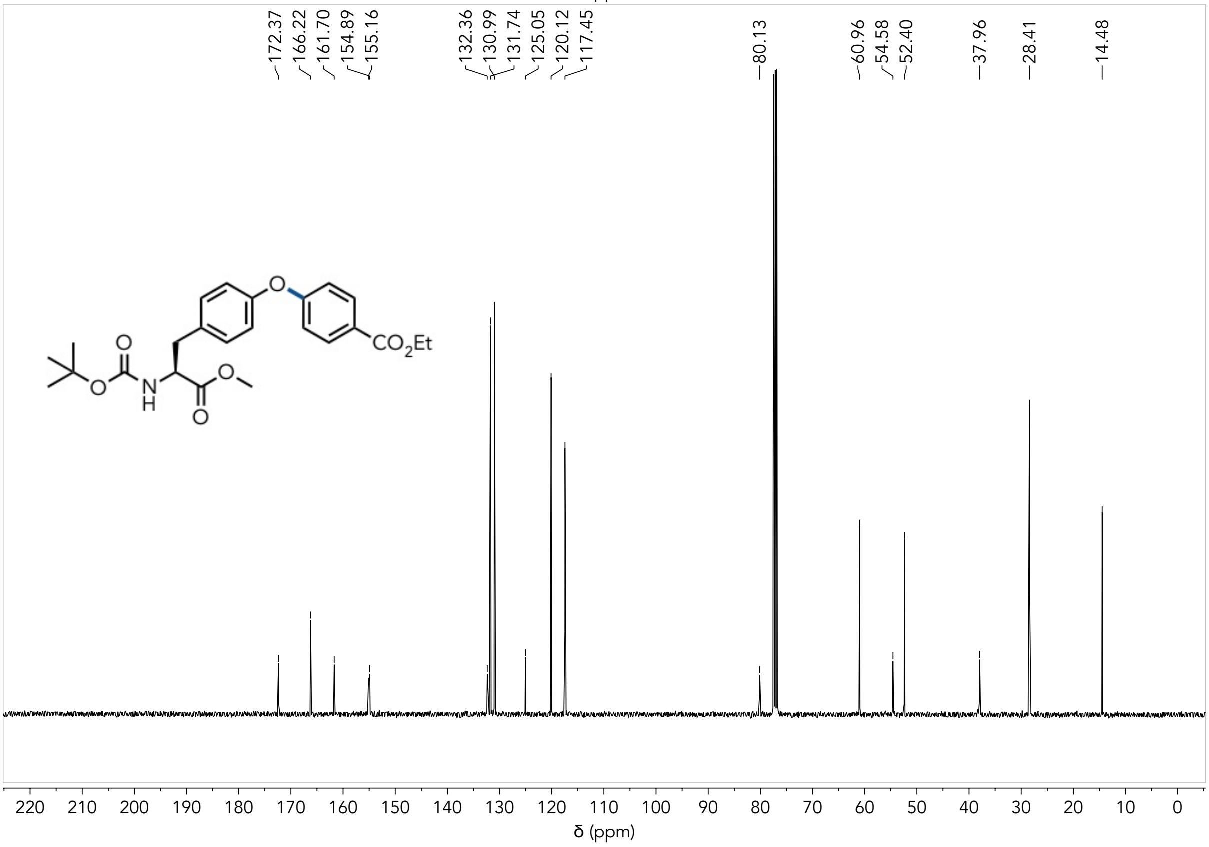
172.37
166.22
161.70
154.89
155.16
132.36
130.99
131.74
125.05
120.12
117.45
80.13
60.96
54.58
52.40
37.96
28.41
14.48
CO2Et
220 210 200 190 180 170 160 150 140 130 120 110 100 90 80 70 60 50 40 30 20 10 0
δ (ppm)

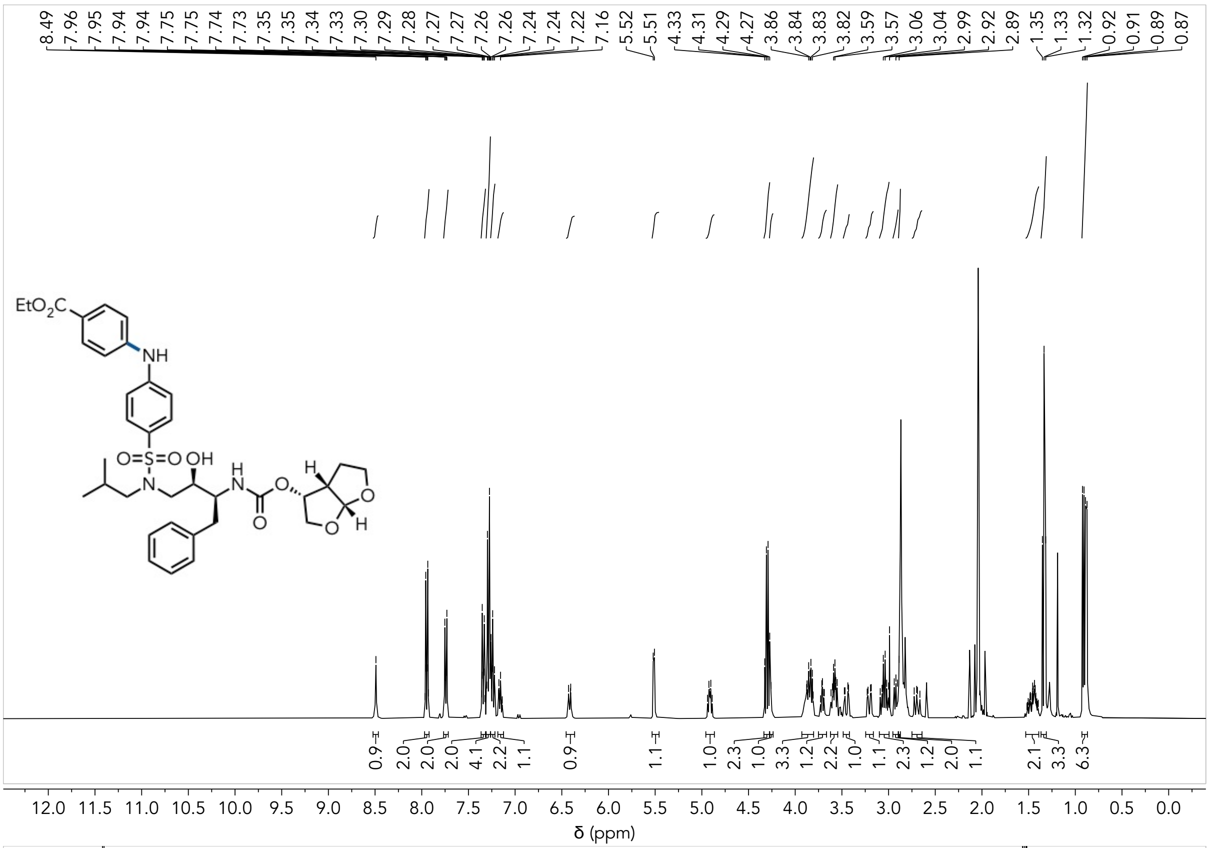
8.49 7.96 7.95 7.94 7.94 7.75 7.75 7.74 7.73 7.35 7.35 7.34 7.33 7.30 7.29 7.28 7.27 7.27 7.26 7.26 7.24 7.24 7.22 7.16 5.52 5.51 4.33 4.31 4.29 4.27 3.86 3.84 3.83 3.82 3.59 3.57 3.06 3.04 2.99 2.92 2.89 1.35 1.33 1.32 0.92 0.91 0.89 0.87
EtO_2C
NH
O=S=O OH
N
H
O
H
0.9 2.0 2.0 2.0 4.1 2.2 1.1 0.9 1.1 1.0 2.3 1.0 3.3 1.2 2.2 1.0 1.1 2.3 1.2 2.0 1.1 2.1 3.3 6.3
12.0 11.5 11.0 10.5 10.0 9.5 9.0 8.5 8.0 7.5 7.0 6.5 6.0 5.5 5.0 4.5 4.0 3.5 3.0 2.5 2.0 1.5 1.0 0.5 0.0
δ (ppm)

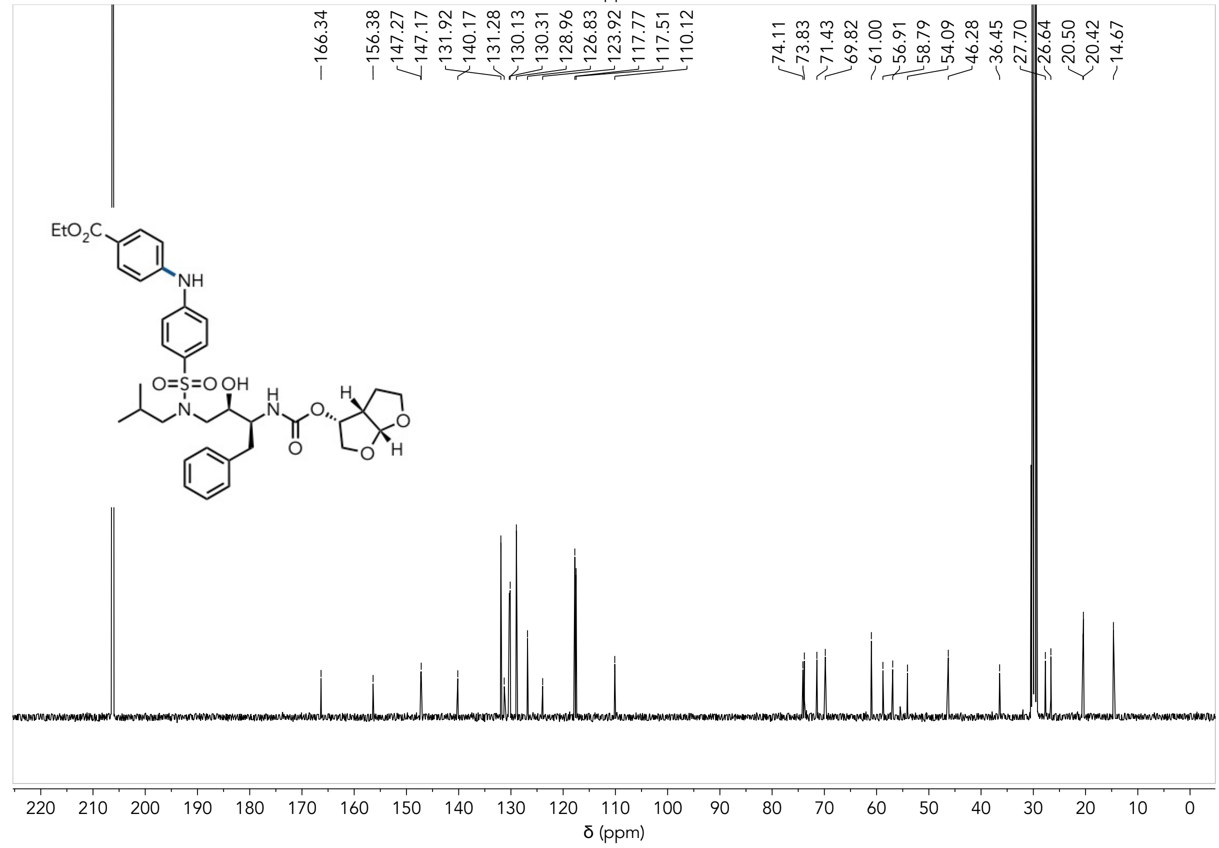
166.34 156.38 147.27 147.17 131.92 140.17 131.28 130.13 130.31 128.96 126.83 123.92 117.77 117.51 110.12 74.11 73.83 71.43 69.82 61.00 56.91 58.79 54.09 46.28 36.45 27.70 26.64 20.50 20.42 14.67
EtO_2C
NH
O=S=O OH
N
H
O
H
220 210 200 190 180 170 160 150 140 130 120 110 100 90 80 70 60 50 40 30 20 10 0
δ (ppm)

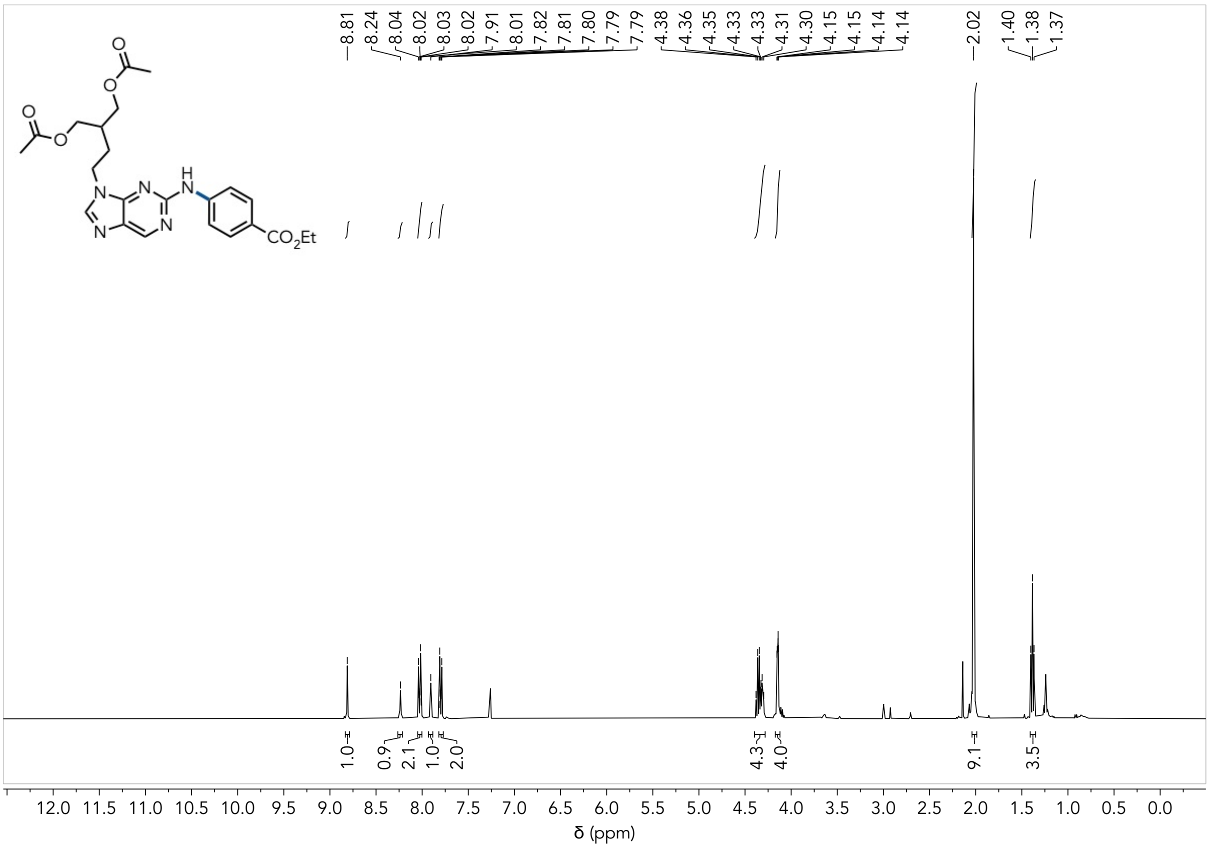

8.81
8.24
8.04
8.02
8.03
8.02
7.91
8.01
7.82
7.81
7.80
7.79
7.79
4.38
4.36
4.35
4.33
4.33
4.31
4.30
4.15
4.15
4.14
4.14
2.02
1.40
1.38
1.37
H
N
CO2Et
1.0
0.9
2.1
1.0
2.0
4.3
4.0
9.1
3.5
12.0 11.5 11.0 10.5 10.0 9.5 9.0 8.5 8.0 7.5 7.0 6.5 6.0 5.5 5.0 4.5 4.0 3.5 3.0 2.5 2.0 1.5 1.0 0.5 0.0
δ (ppm)

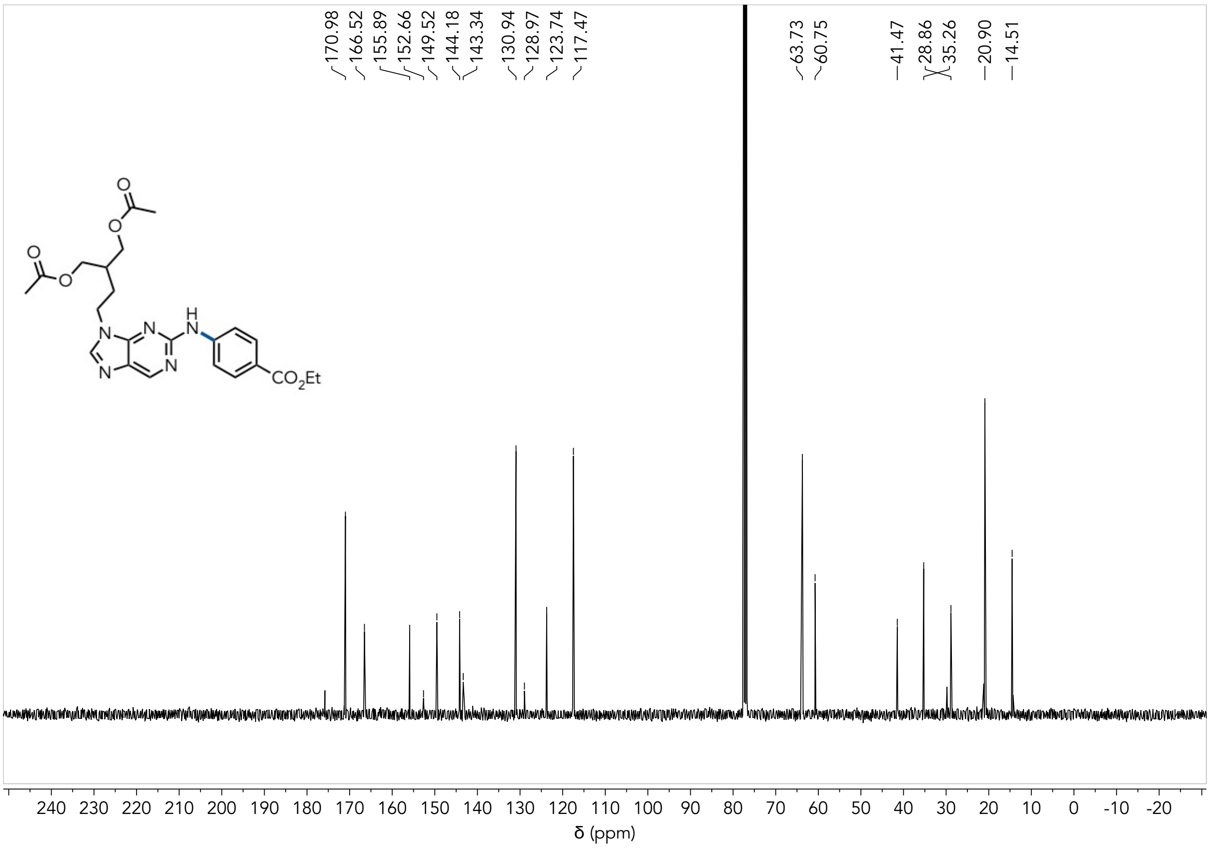

170.98
166.52
155.89
152.66
149.52
144.18
143.34
130.94
128.97
123.74
117.47
63.73
60.75
41.47
28.86
35.26
20.90
14.51
H
N
CO2Et
240 230 220 210 200 190 180 170 160 150 140 130 120 110 100 90 80 70 60 50 40 30 20 10 0 -10 -20
δ (ppm)

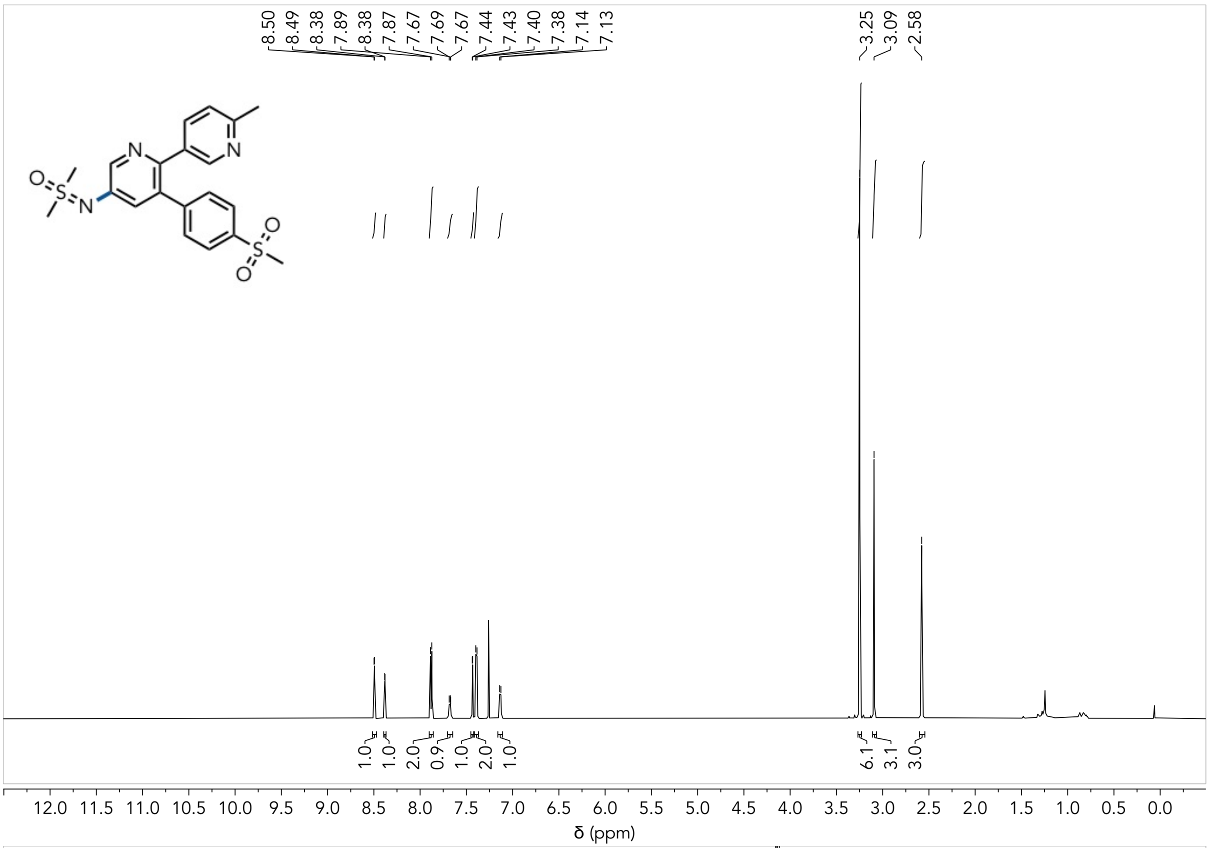
8.50
8.49
8.38
7.89
8.38
7.87
7.67
7.69
7.67
7.44
7.43
7.40
7.38
7.14
7.13
3.25
3.09
2.58
1.0
1.0
2.0
0.9
1.0
2.0
1.0
6.1
3.1
3.0
δ (ppm)

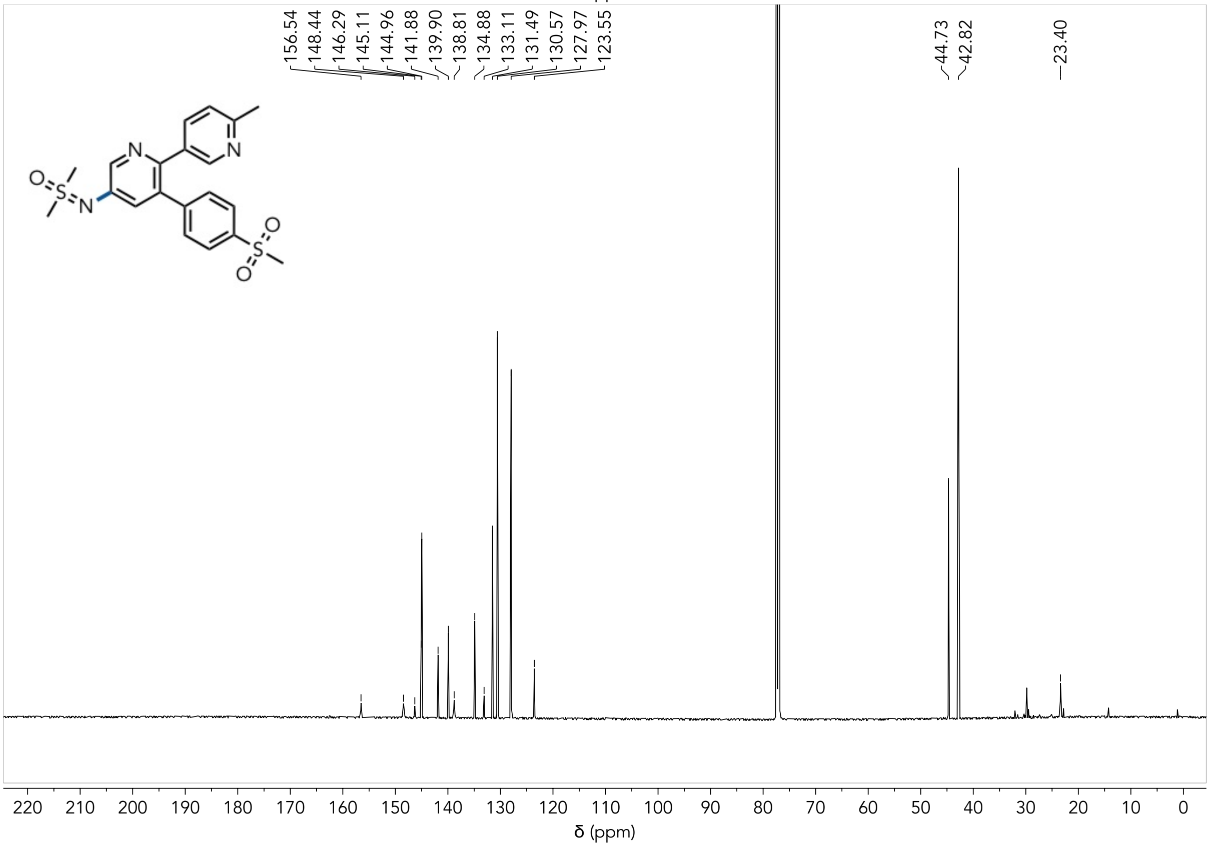
156.54
148.44
146.29
145.11
144.96
141.88
139.90
138.81
134.88
133.11
131.49
130.57
127.97
123.55
44.73
42.82
23.40
δ (ppm)

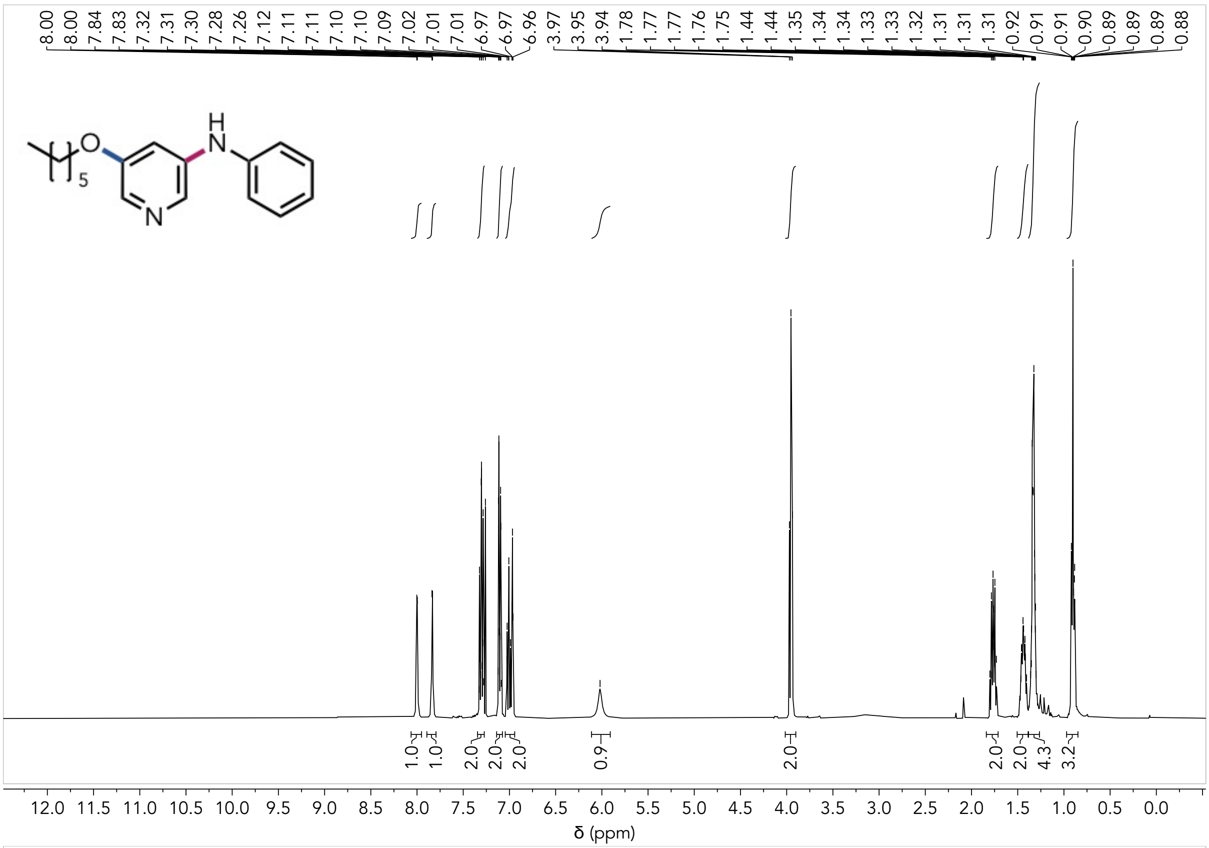
8.00
8.00
7.84
7.83
7.32
7.31
7.30
7.28
7.26
7.12
7.11
7.11
7.10
7.10
7.09
7.02
7.01
7.01
6.97
6.97
6.96
3.97
3.95
3.94
1.78
1.77
1.77
1.76
1.75
1.44
1.44
1.35
1.34
1.34
1.33
1.33
1.32
1.31
1.31
1.31
0.92
0.91
0.91
0.90
0.89
0.89
0.89
0.88
H
N
O
5
1.0
1.0
2.0
2.0
2.0
0.9
2.0
2.0
2.0
4.3
3.2
12.0 11.5 11.0 10.5 10.0 9.5 9.0 8.5 8.0 7.5 7.0 6.5 6.0 5.5 5.0 4.5 4.0 3.5 3.0 2.5 2.0 1.5 1.0 0.5 0.0
δ (ppm)

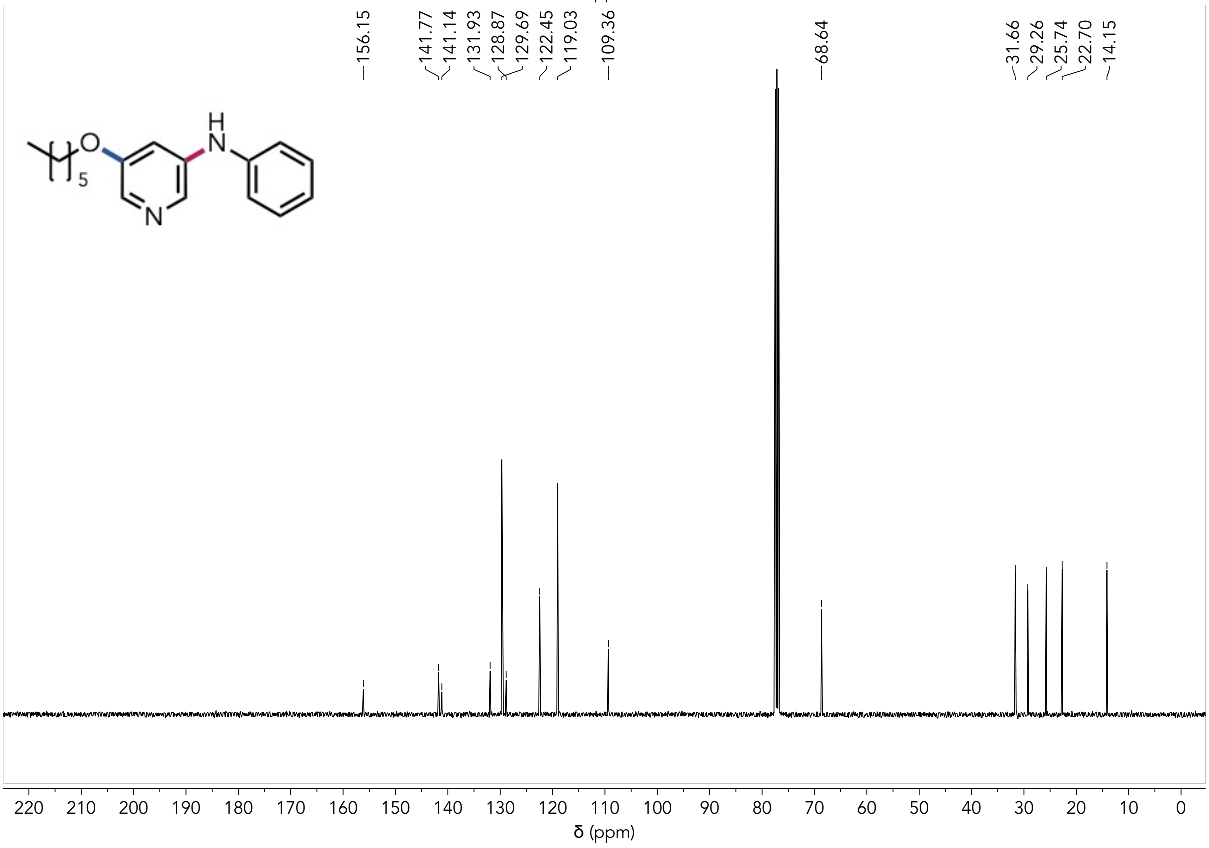
156.15
141.77
141.14
131.93
128.87
129.69
122.45
119.03
109.36
68.64
31.66
29.26
25.74
22.70
14.15
H
N
O
5
220 210 200 190 180 170 160 150 140 130 120 110 100 90 80 70 60 50 40 30 20 10 0
δ (ppm)

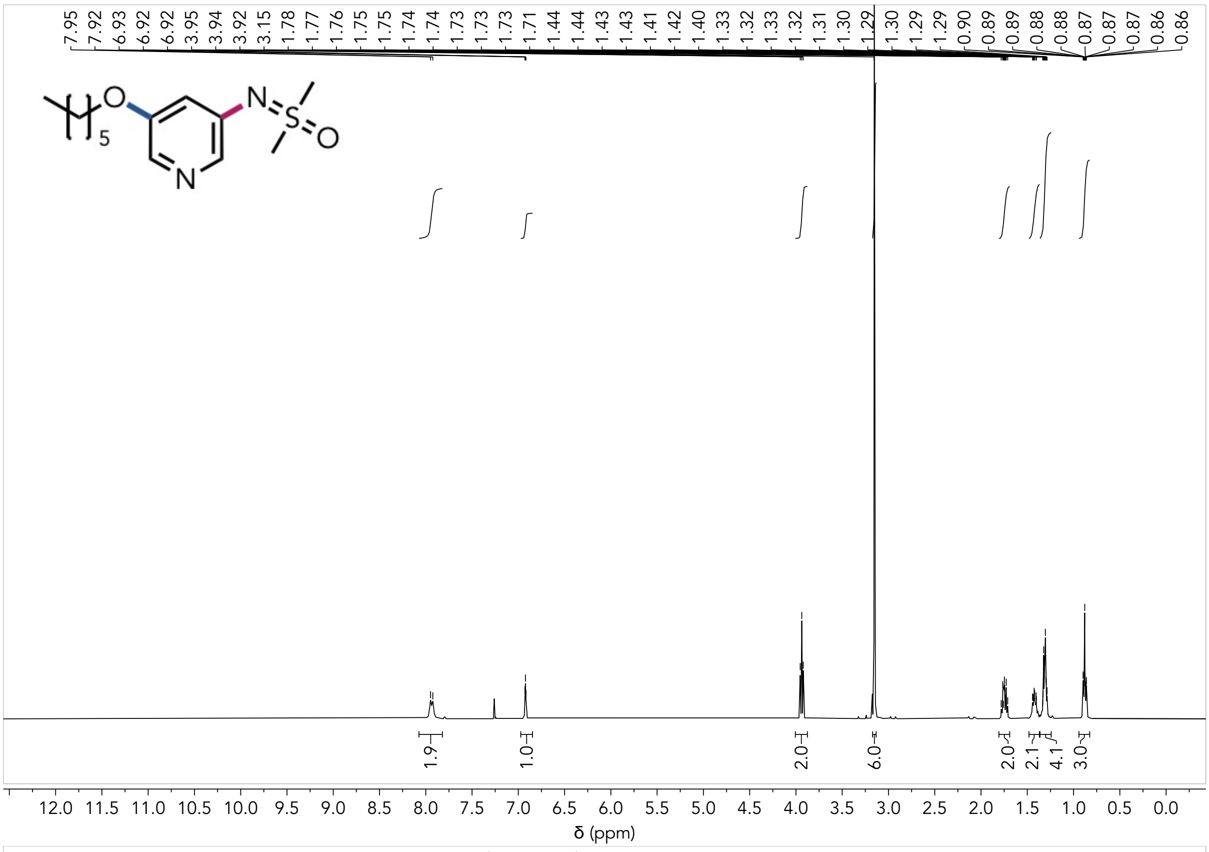

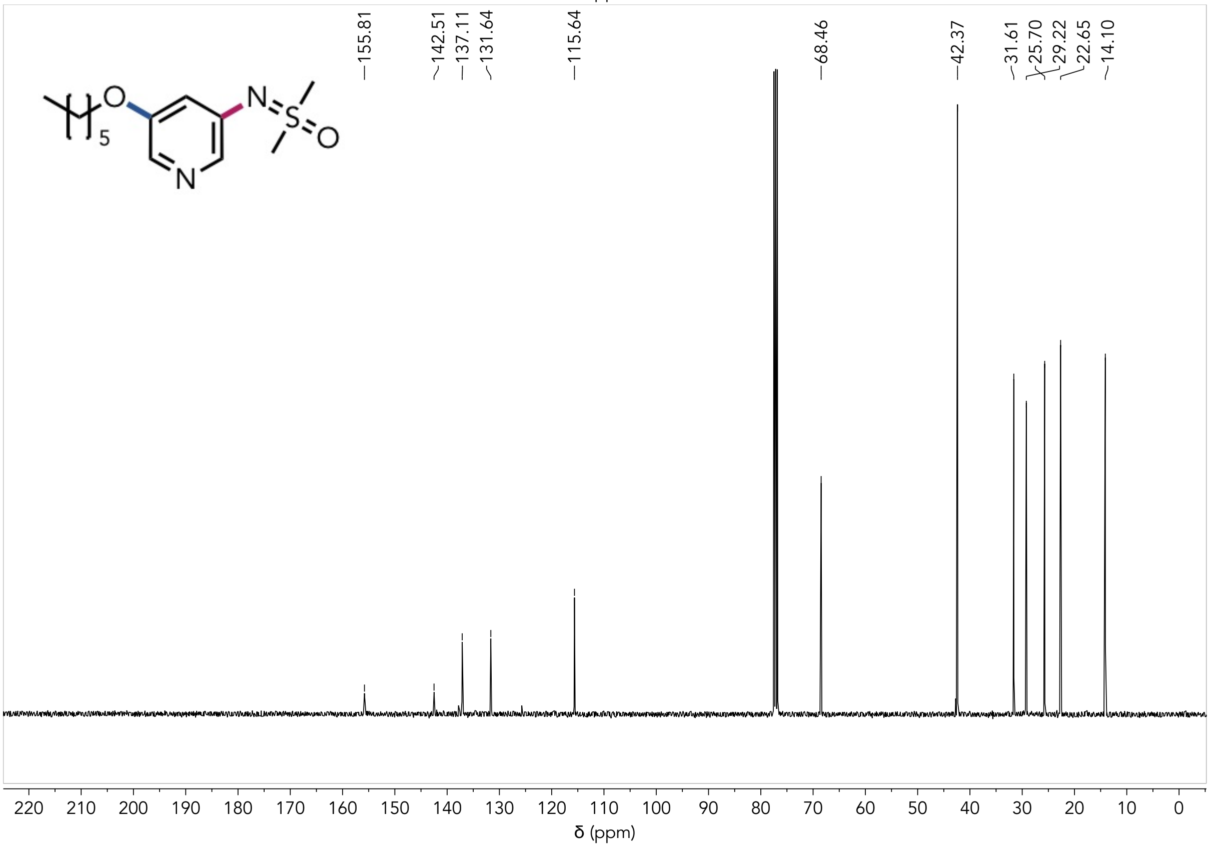

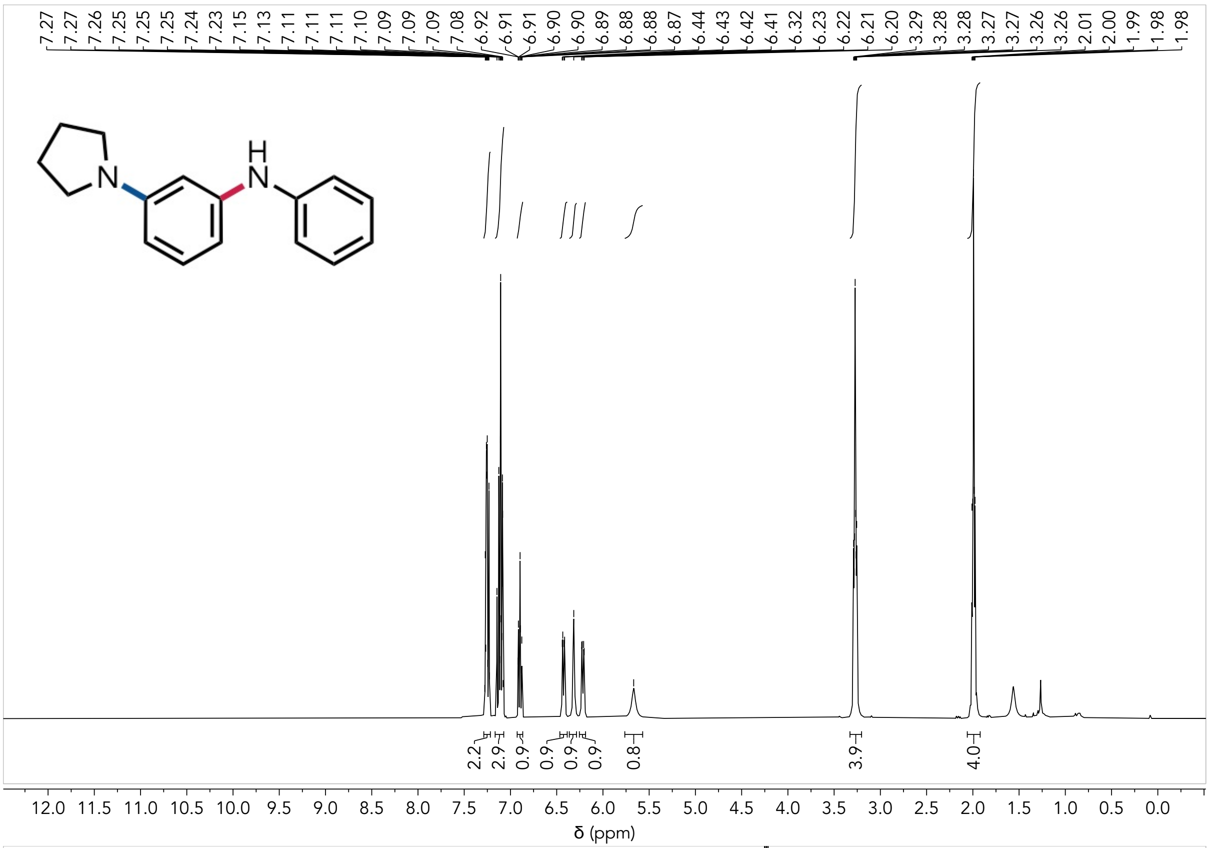
7.27 7.27 7.26 7.25 7.25 7.25 7.24 7.23 7.15 7.13 7.11 7.11 7.11 7.10 7.09 7.09 7.09 7.08 6.92 6.91 6.91 6.90 6.90 6.89 6.88 6.88 6.87 6.44 6.43 6.42 6.41 6.32 6.23 6.22 6.21 6.20 3.29 3.28 3.28 3.27 3.27 3.26 2.01 2.00 1.99 1.98 1.98
H
N
N
2.2 2.9 0.9 0.9 0.9 0.9 0.8 3.9 4.0
12.0 11.5 11.0 10.5 10.0 9.5 9.0 8.5 8.0 7.5 7.0 6.5 6.0 5.5 5.0 4.5 4.0 3.5 3.0 2.5 2.0 1.5 1.0 0.5 0.0
δ (ppm)

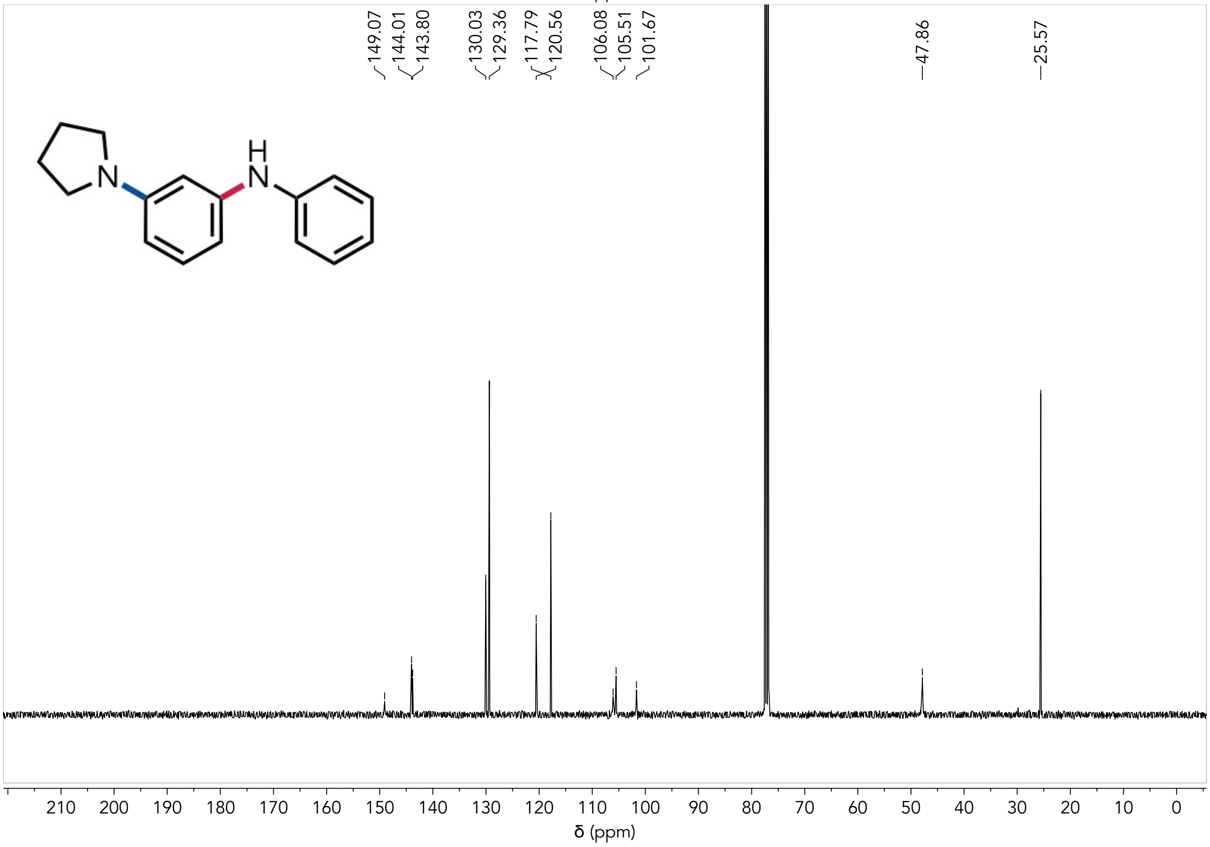
149.07 144.01 143.80 130.03 129.36 117.79 120.56 106.08 105.51 101.67 47.86 25.57
H
N
N
210 200 190 180 170 160 150 140 130 120 110 100 90 80 70 60 50 40 30 20 10 0
δ (ppm)

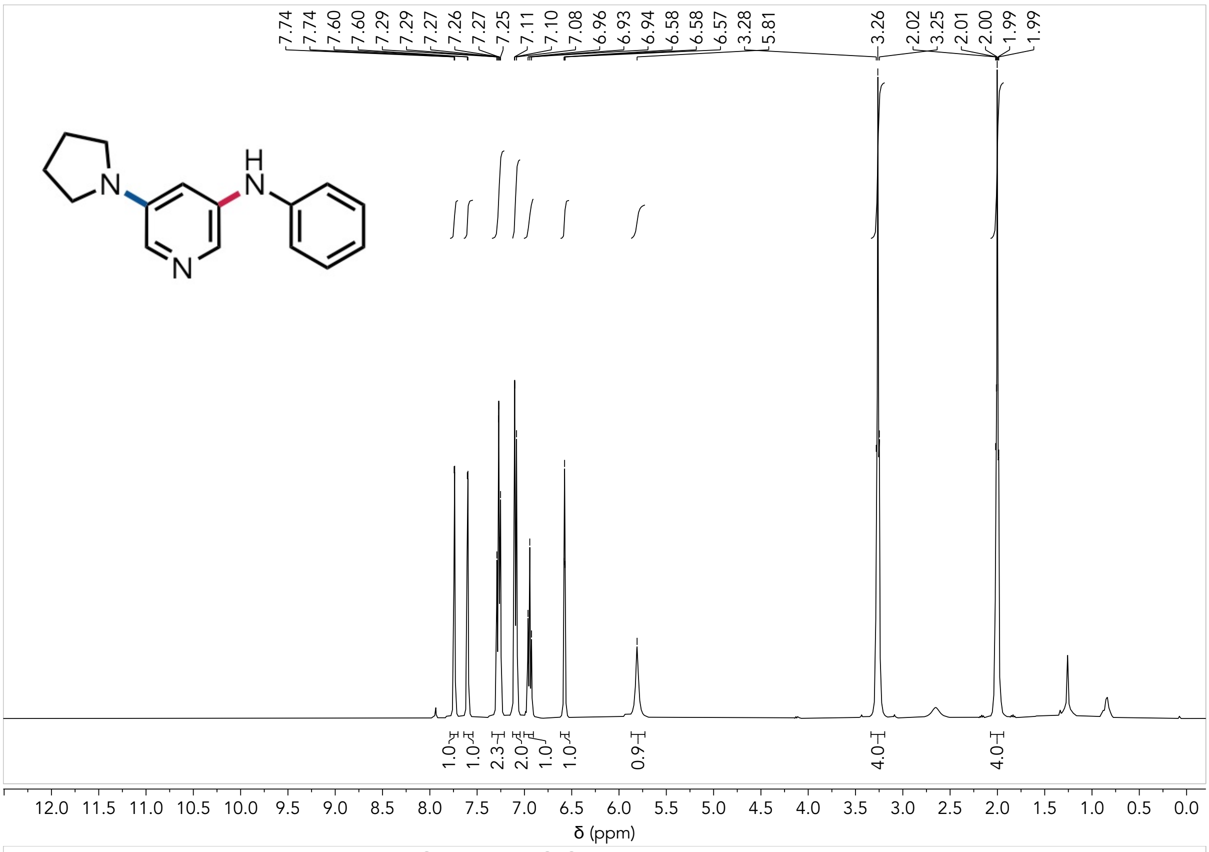
7.74 7.74 7.60 7.60 7.29 7.29 7.27 7.26 7.27 7.25 7.11 7.10 7.08 6.96 6.93 6.94 6.58 6.58 6.57 3.28 5.81 3.26 2.02 3.25 2.01 2.00 1.99 1.99
H
N
N
N
1.0 1.0 2.3 2.0 1.0 1.0 0.9 4.0 4.0
12.0 11.5 11.0 10.5 10.0 9.5 9.0 8.5 8.0 7.5 7.0 6.5 6.0 5.5 5.0 4.5 4.0 3.5 3.0 2.5 2.0 1.5 1.0 0.5 0.0
δ (ppm)

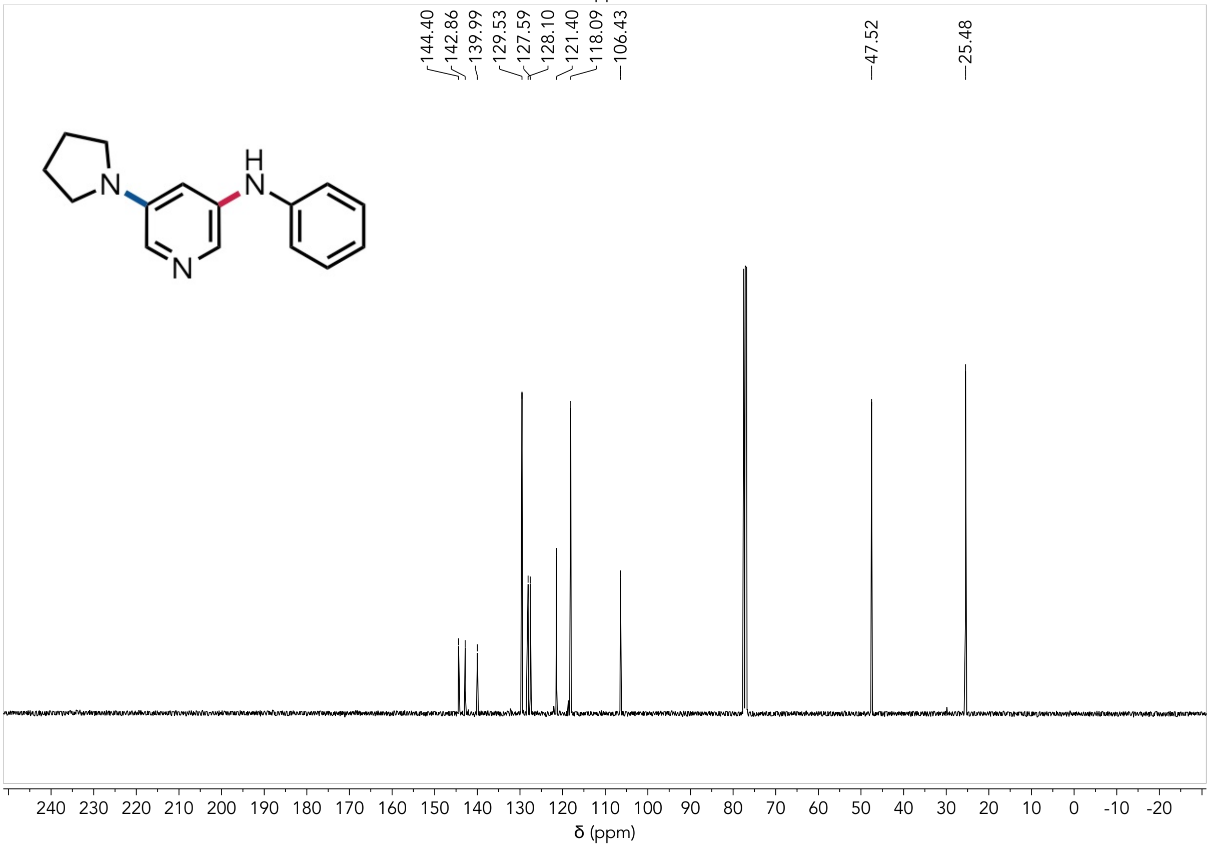
144.40 142.86 139.99 129.53 127.59 128.10 121.40 118.09 106.43 47.52 25.48
H
N
N
N
240 230 220 210 200 190 180 170 160 150 140 130 120 110 100 90 80 70 60 50 40 30 20 10 0 -10 -20
δ (ppm)

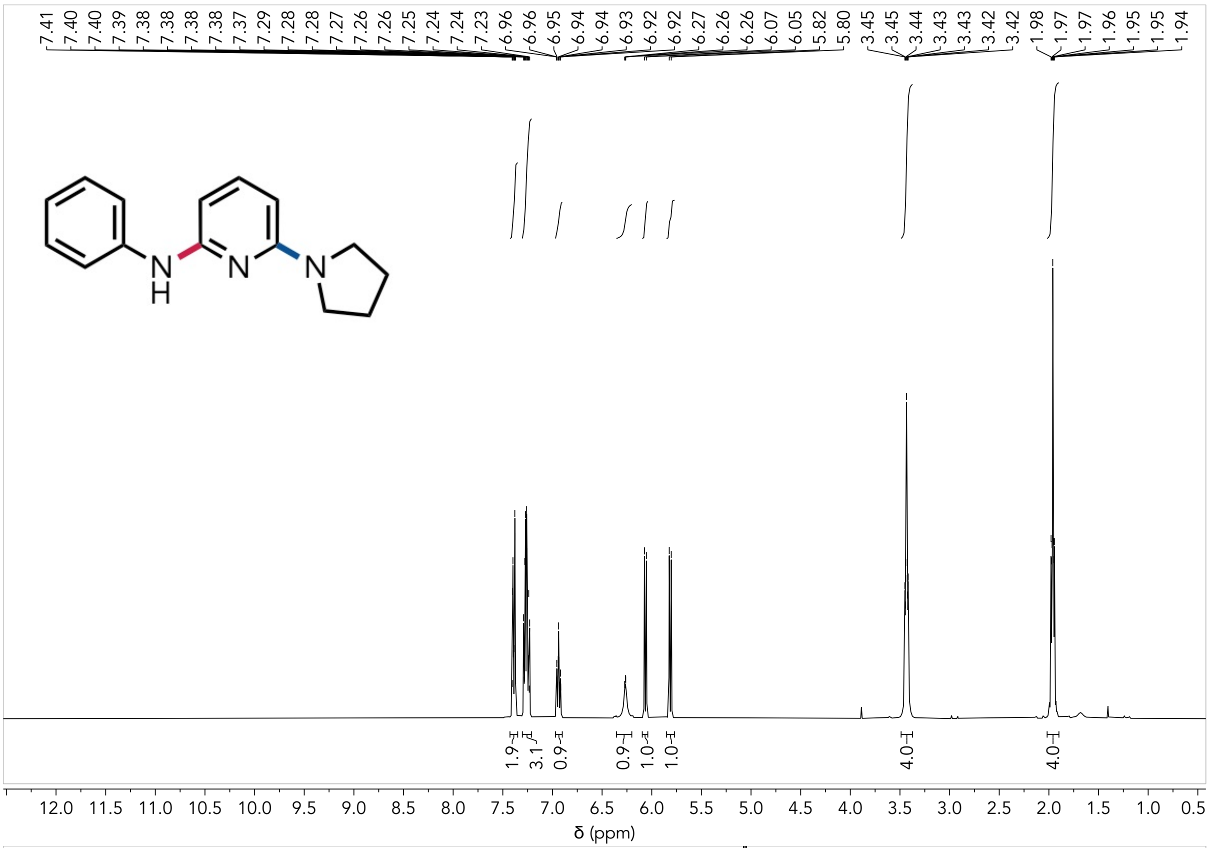
7.41
7.40
7.40
7.39
7.38
7.38
7.38
7.38
7.37
7.29
7.28
7.28
7.27
7.26
7.26
7.25
7.24
7.24
7.23
6.96
6.96
6.95
6.94
6.94
6.93
6.92
6.92
6.92
6.27
6.26
6.26
6.07
6.05
5.82
5.80
3.45
3.45
3.44
3.43
3.43
3.42
3.42
1.98
1.97
1.97
1.96
1.95
1.95
1.94
N
H
N
N
1.9
3.1
0.9
0.9
1.0
1.0
4.0
4.0
12.0 11.5 11.0 10.5 10.0 9.5 9.0 8.5 8.0 7.5 7.0 6.5 6.0 5.5 5.0 4.5 4.0 3.5 3.0 2.5 2.0 1.5 1.0 0.5
δ (ppm)

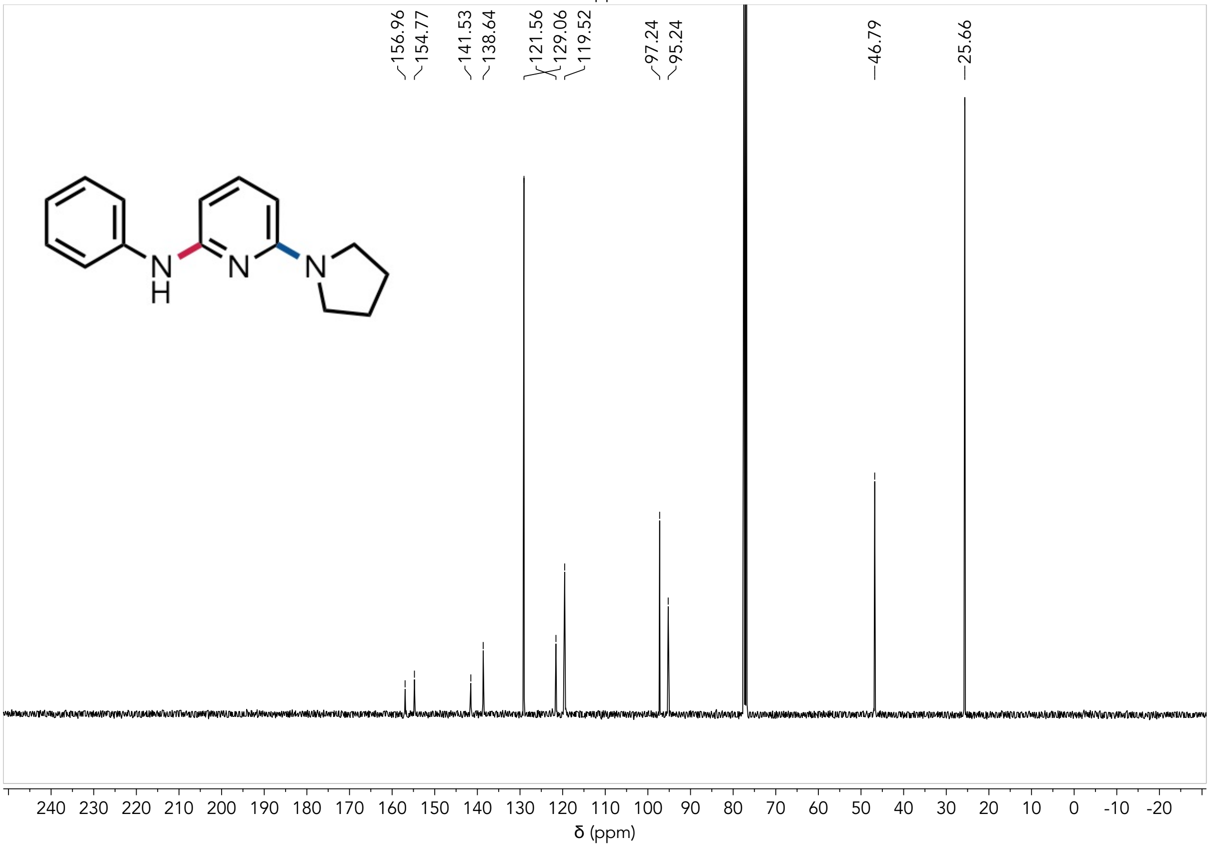
156.96
154.77
141.53
138.64
121.56
129.06
119.52
97.24
95.24
46.79
25.66
N
H
N
N
240 230 220 210 200 190 180 170 160 150 140 130 120 110 100 90 80 70 60 50 40 30 20 10 0 -10 -20
δ (ppm)

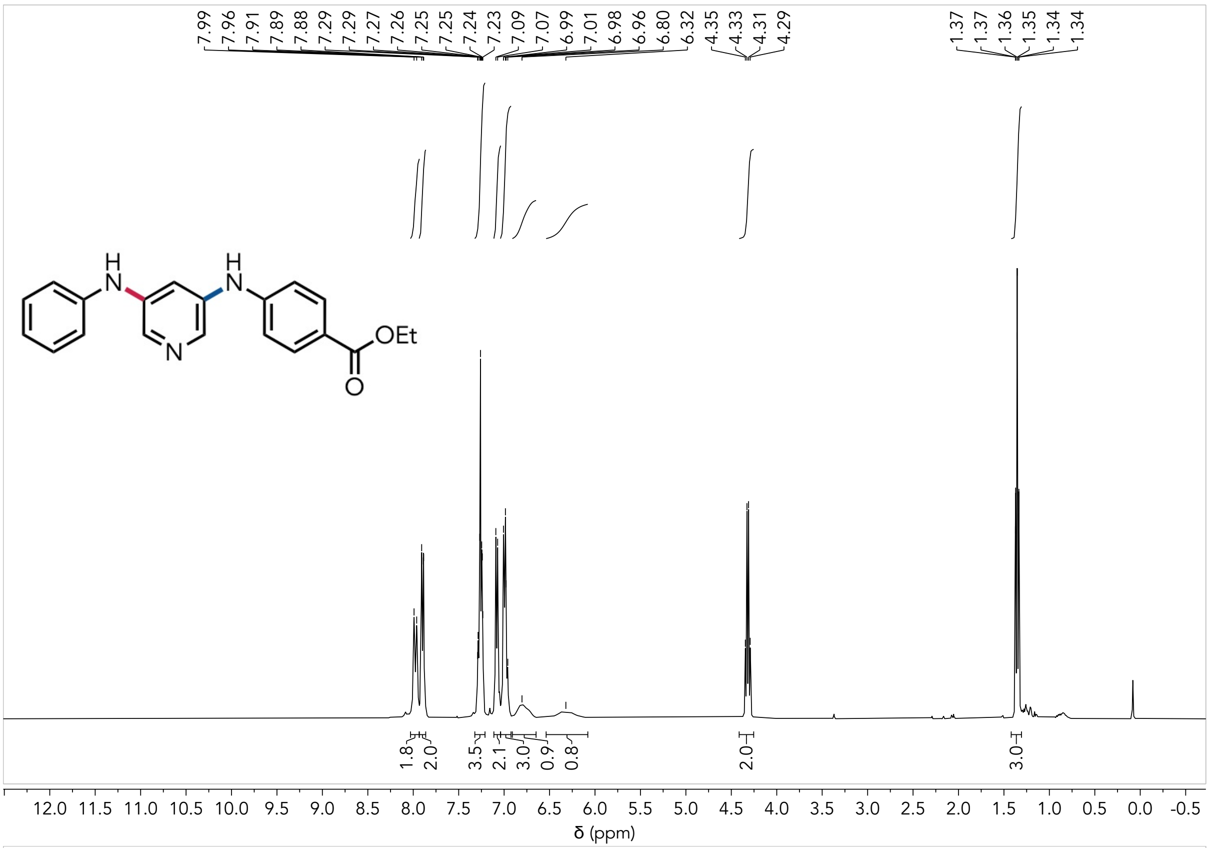
7.99
7.96
7.91
7.89
7.88
7.29
7.29
7.27
7.26
7.25
7.25
7.24
7.23
7.09
7.07
6.99
7.01
6.98
6.96
6.80
6.32
4.35
4.33
4.31
4.29
1.37
1.37
1.36
1.35
1.34
1.34
OEt
1.8
2.0
3.5
2.1
3.0
0.9
0.8
2.0
3.0
12.0 11.5 11.0 10.5 10.0 9.5 9.0 8.5 8.0 7.5 7.0 6.5 6.0 5.5 5.0 4.5 4.0 3.5 3.0 2.5 2.0 1.5 1.0 0.5 0.0 -0.5
δ (ppm)

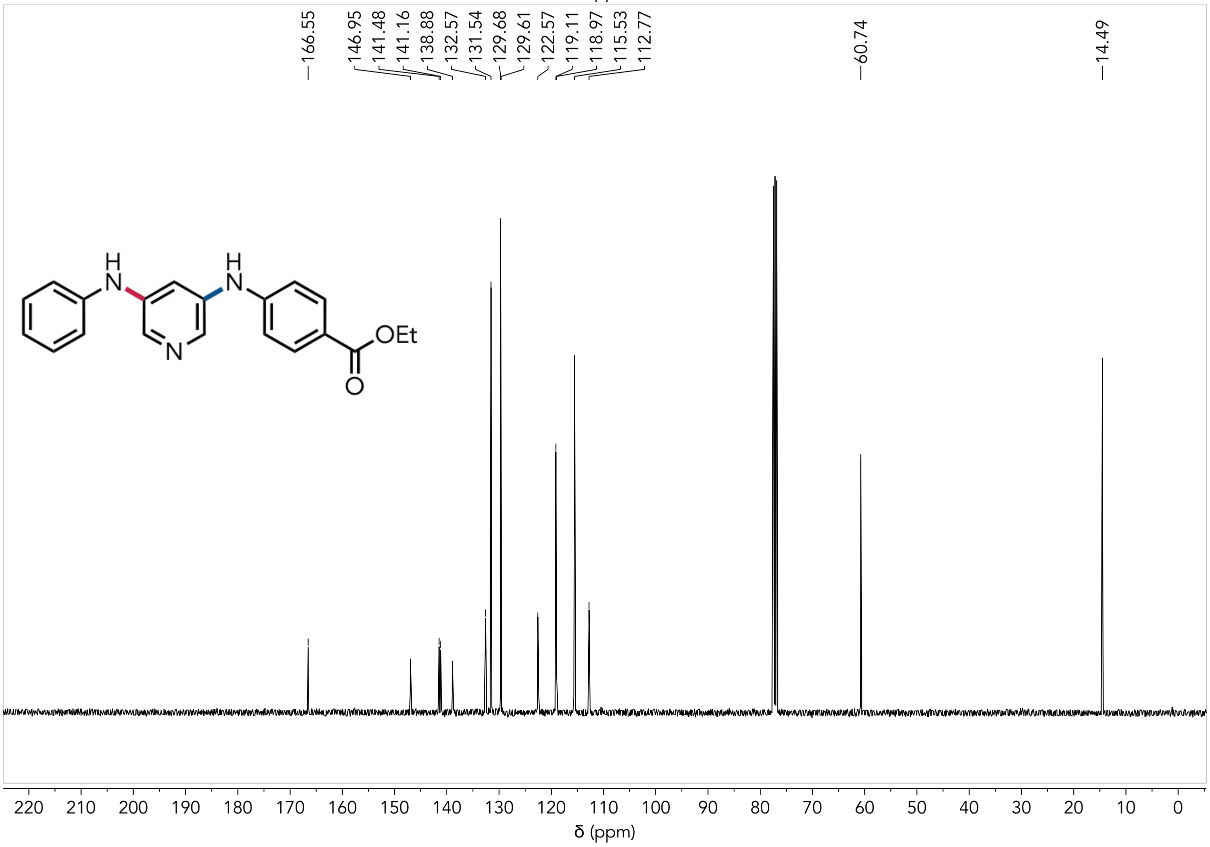
166.55
146.95
141.48
141.16
138.88
132.57
131.54
129.68
129.61
122.57
119.11
118.97
115.53
112.77
60.74
14.49
OEt
220 210 200 190 180 170 160 150 140 130 120 110 100 90 80 70 60 50 40 30 20 10 0
δ (ppm)

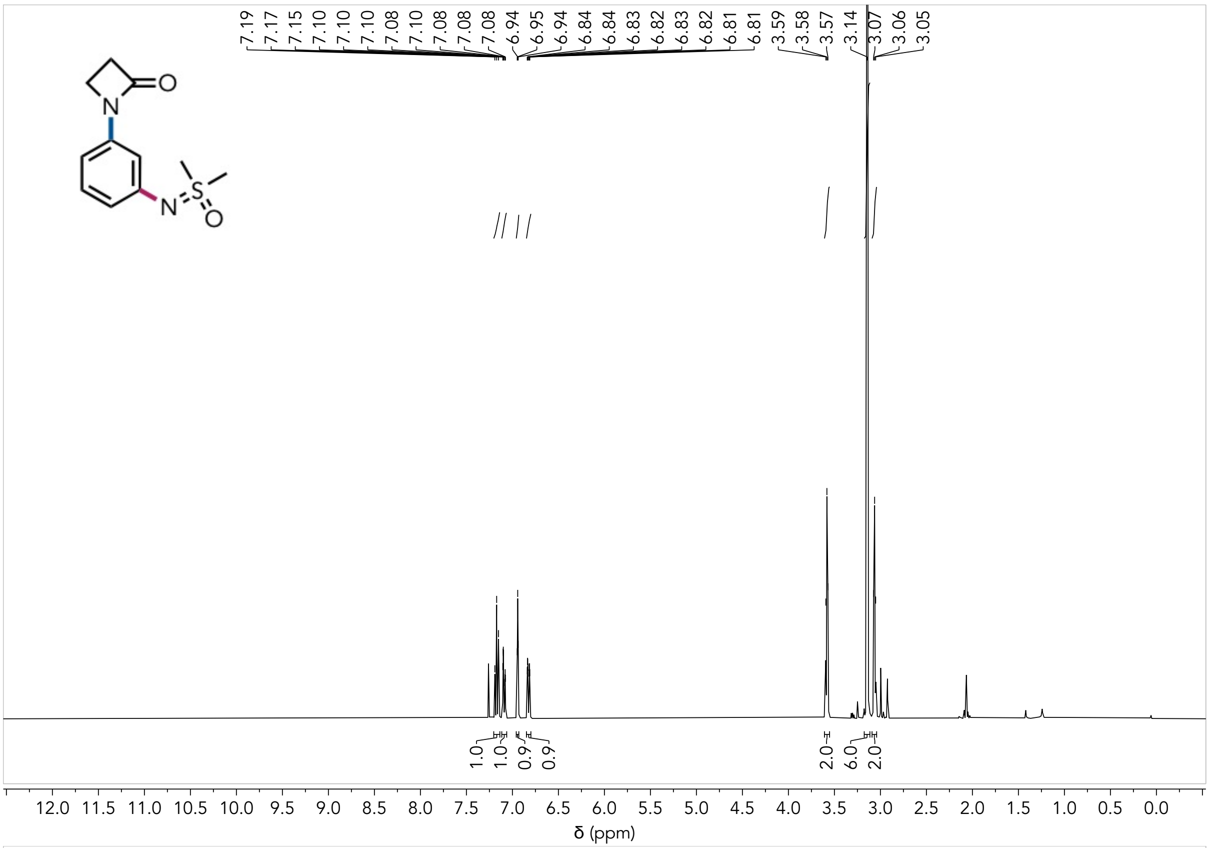
7.19
7.17
7.15
7.10
7.10
7.10
7.08
7.10
7.08
7.08
7.08
6.94
6.95
6.94
6.84
6.84
6.83
6.82
6.83
6.82
6.81
6.81
3.59
3.58
3.57
3.14
3.07
3.06
3.05
1.0
1.0
0.9
0.9
2.0
6.0
2.0
12.0 11.5 11.0 10.5 10.0 9.5 9.0 8.5 8.0 7.5 7.0 6.5 6.0 5.5 5.0 4.5 4.0 3.5 3.0 2.5 2.0 1.5 1.0 0.5 0.0
δ (ppm)

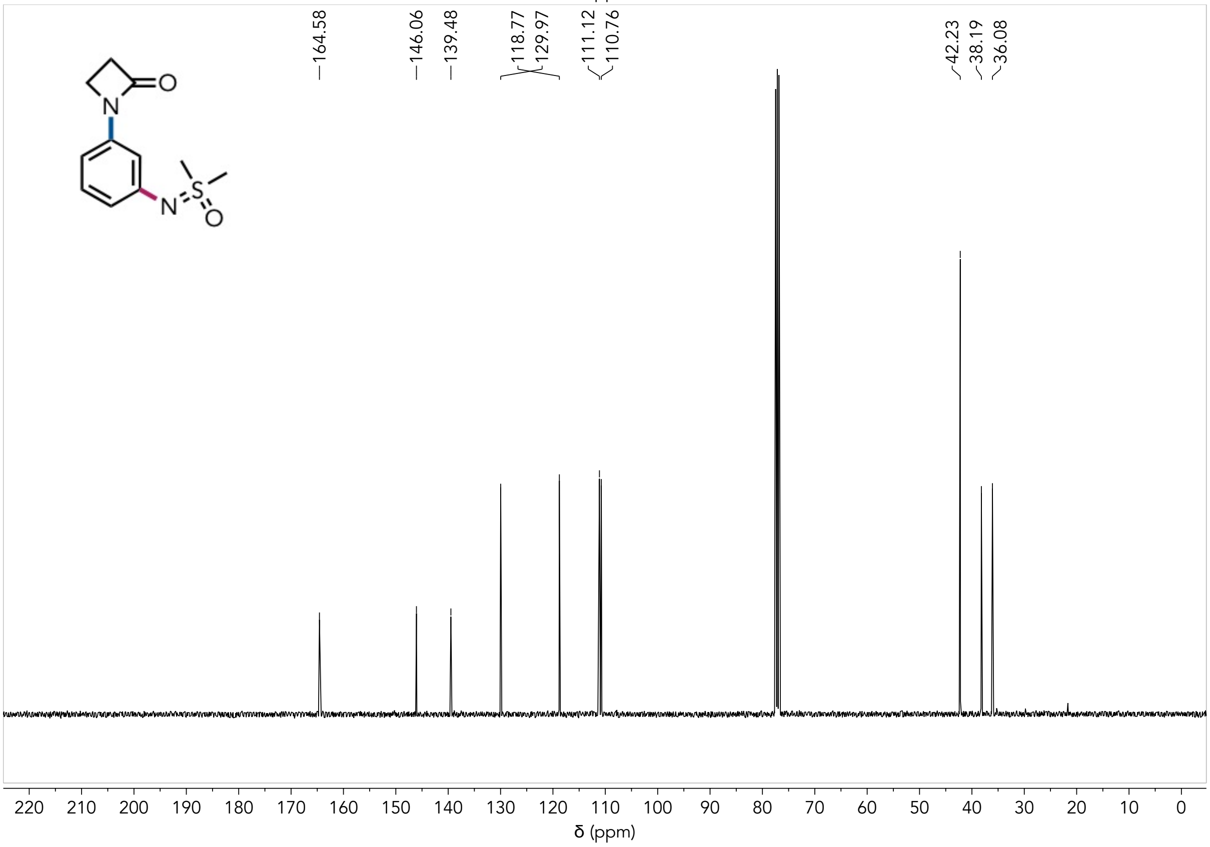
164.58
146.06
139.48
118.77
129.97
111.12
110.76
42.23
38.19
36.08
220 210 200 190 180 170 160 150 140 130 120 110 100 90 80 70 60 50 40 30 20 10 0
δ (ppm)

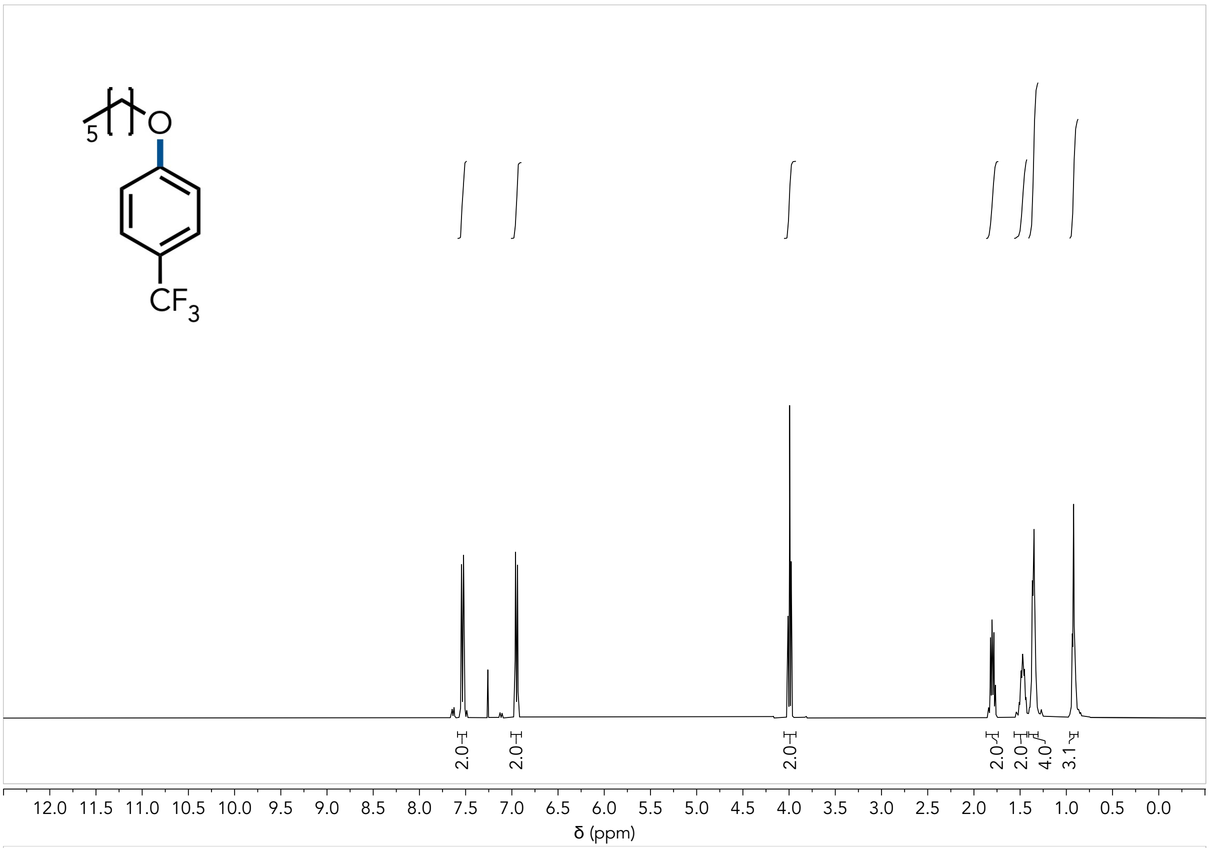
5
O
CF3
2.0
2.0
2.0
2.0
2.0
4.0
3.1
12.0 11.5 11.0 10.5 10.0 9.5 9.0 8.5 8.0 7.5 7.0 6.5 6.0 5.5 5.0 4.5 4.0 3.5 3.0 2.5 2.0 1.5 1.0 0.5 0.0
δ (ppm)

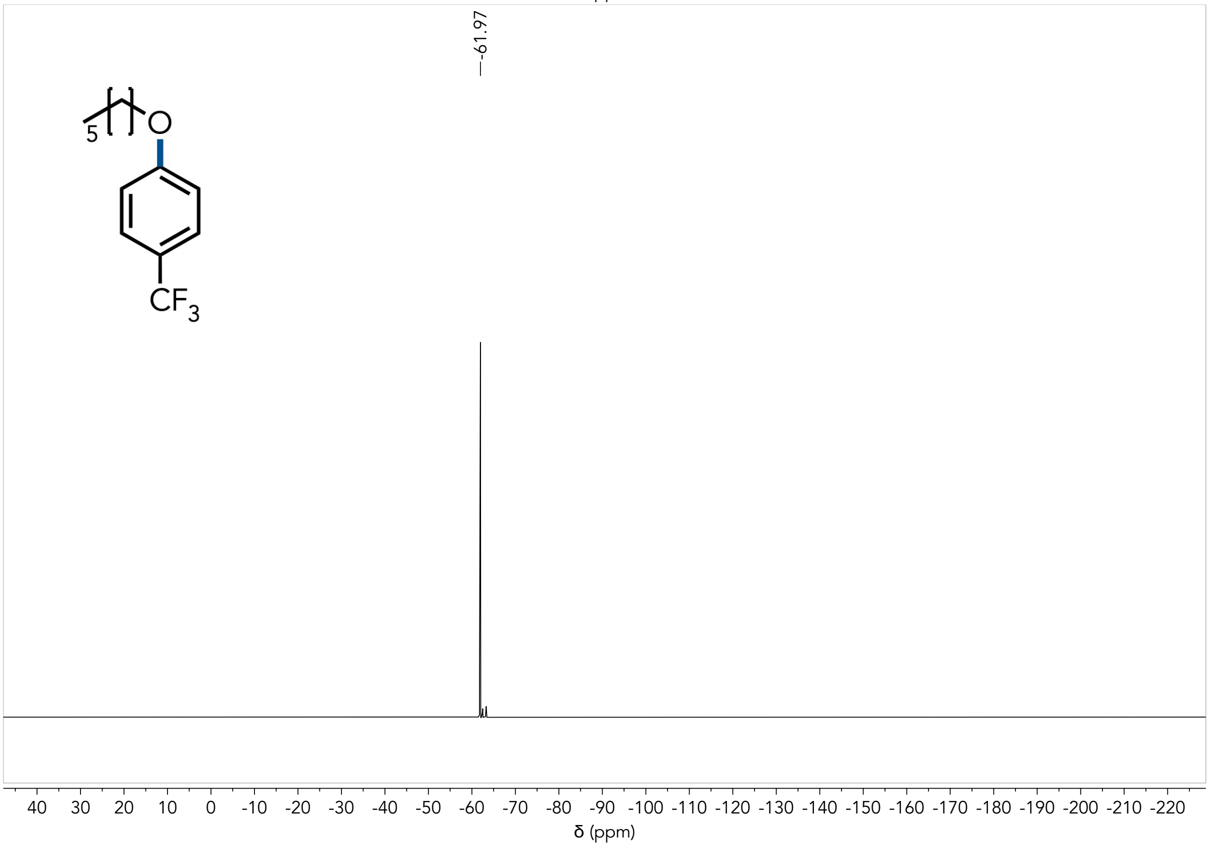
-61.97
5
O
CF3
40 30 20 10 0 -10 -20 -30 -40 -50 -60 -70 -80 -90 -100 -110 -120 -130 -140 -150 -160 -170 -180 -190 -200 -210 -220
δ (ppm)

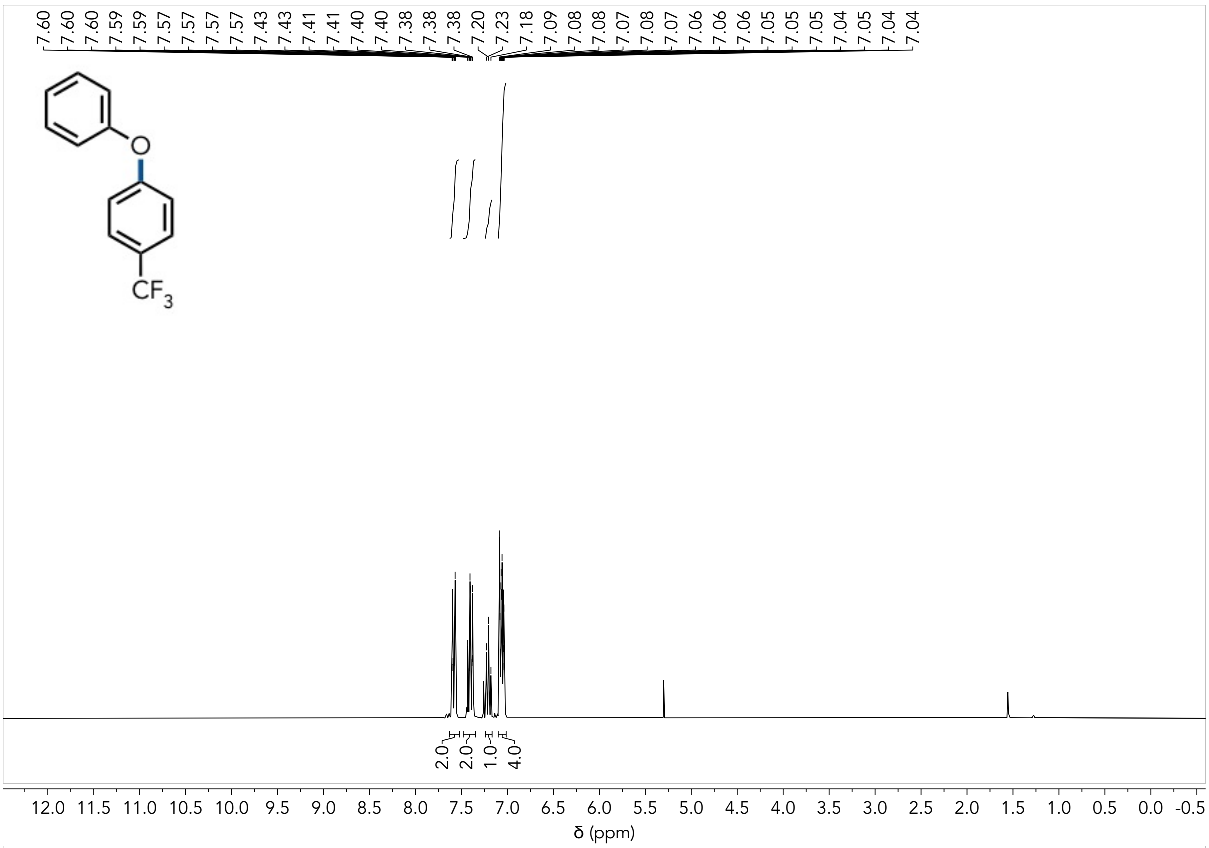

O
CF3
7.60 7.60 7.60 7.59 7.59 7.57 7.57 7.57 7.57 7.43 7.43 7.41 7.41 7.40 7.40 7.38 7.38 7.38 7.20 7.23 7.18 7.09 7.08 7.08 7.07 7.08 7.07 7.06 7.06 7.06 7.05 7.05 7.05 7.04 7.05 7.04 7.04
2.0 2.0 1.0 4.0
12.0 11.5 11.0 10.5 10.0 9.5 9.0 8.5 8.0 7.5 7.0 6.5 6.0 5.5 5.0 4.5 4.0 3.5 3.0 2.5 2.0 1.5 1.0 0.5 0.0 -0.5
δ (ppm)

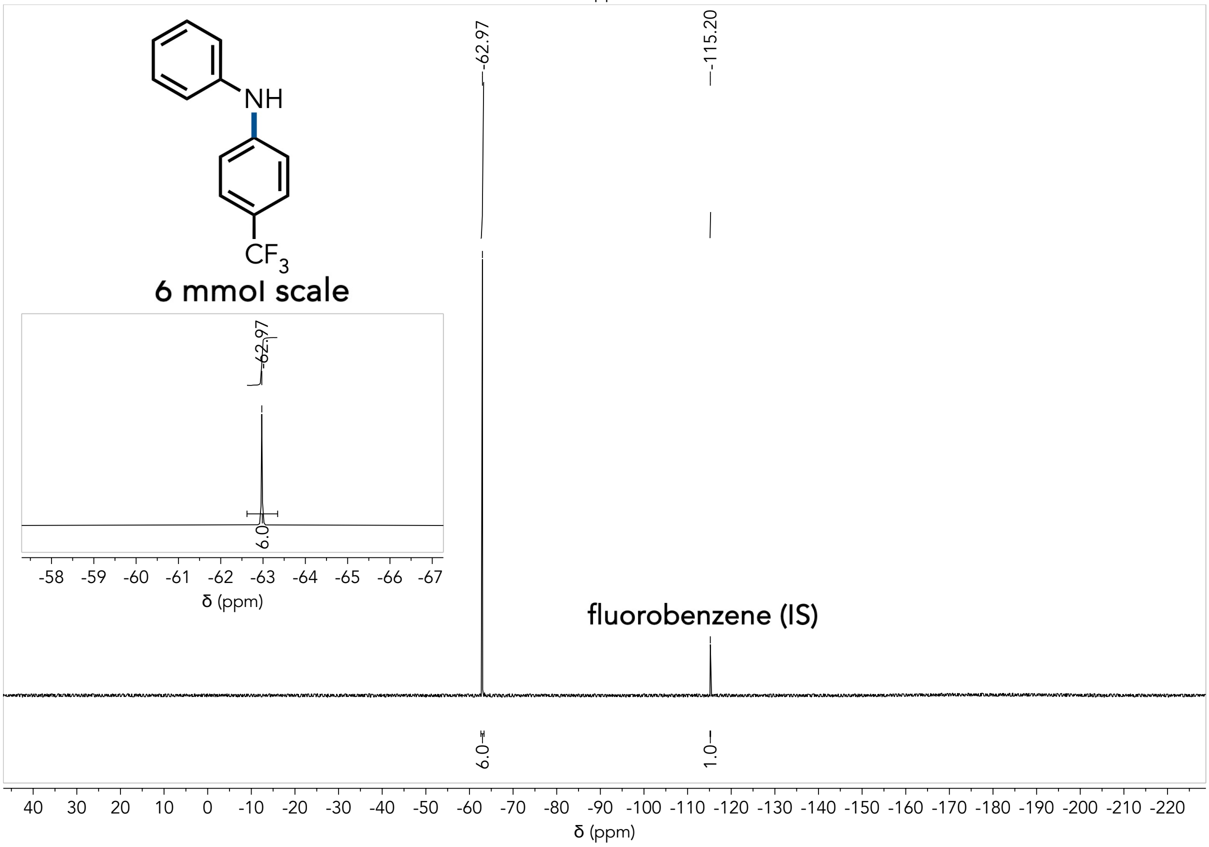

NH
CF3
6 mmol scale
-62.97
-115.20
-62.97
6.0
-58 -59 -60 -61 -62 -63 -64 -65 -66 -67
δ (ppm)
fluorobenzene (IS)
6.0
1.0
40 30 20 10 0 -10 -20 -30 -40 -50 -60 -70 -80 -90 -100 -110 -120 -130 -140 -150 -160 -170 -180 -190 -200 -210 -220
δ (ppm)

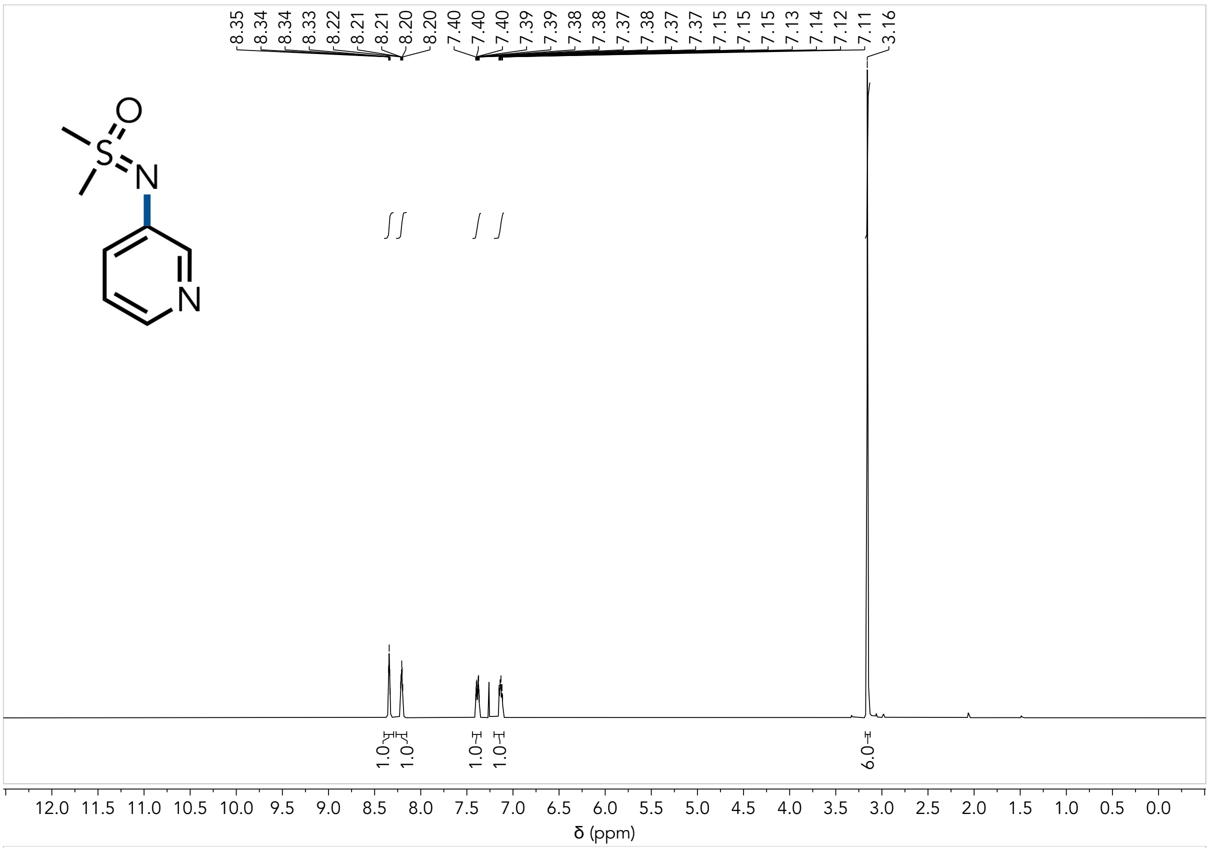
8.35 8.34 8.34 8.33 8.22 8.21 8.21 8.20 8.20 7.40 7.40 7.40 7.39 7.39 7.38 7.38 7.37 7.38 7.37 7.37 7.15 7.15 7.15 7.13 7.14 7.12 7.11 3.16
O
S
N
N
1.0
1.0
1.0
1.0
6.0
12.0 11.5 11.0 10.5 10.0 9.5 9.0 8.5 8.0 7.5 7.0 6.5 6.0 5.5 5.0 4.5 4.0 3.5 3.0 2.5 2.0 1.5 1.0 0.5 0.0
δ (ppm)

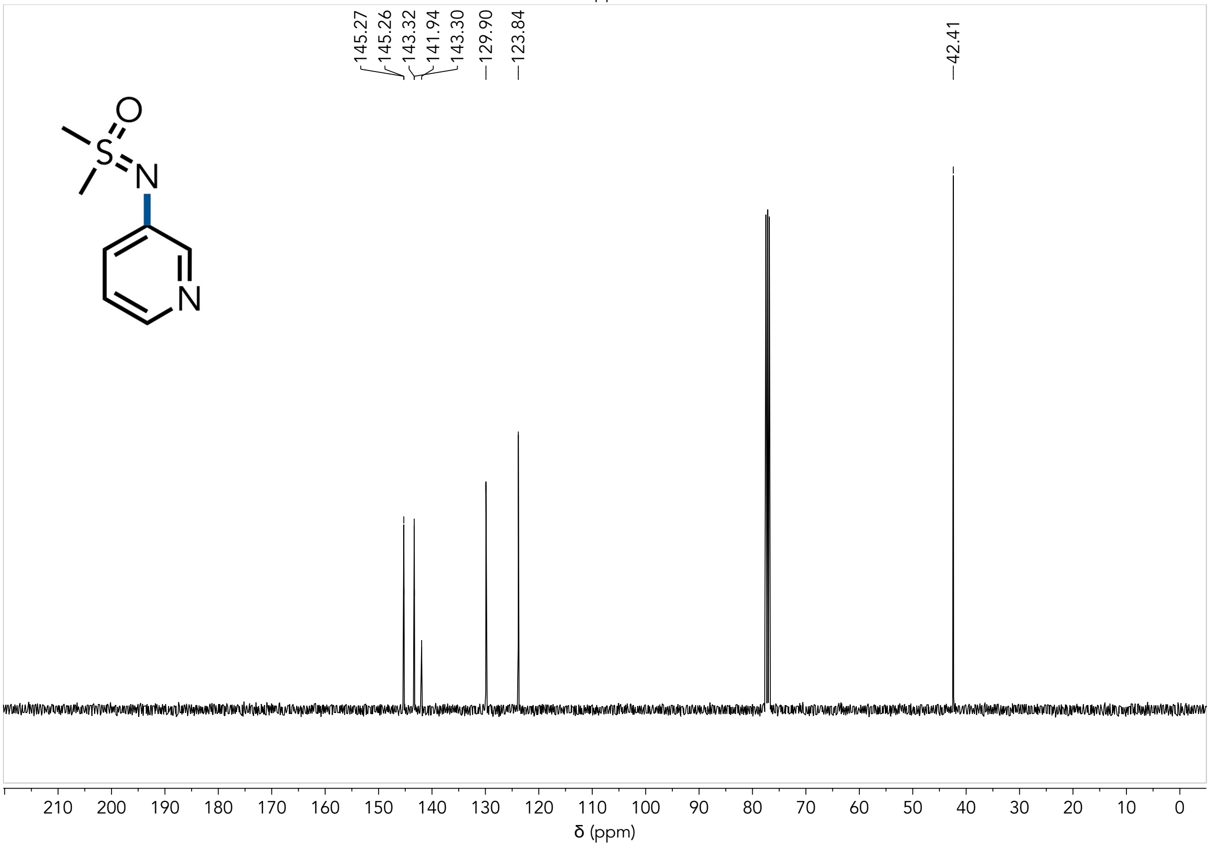
145.27 145.26 143.32 141.94 143.30 129.90 123.84
42.41
O
S
N
N
210 200 190 180 170 160 150 140 130 120 110 100 90 80 70 60 50 40 30 20 10 0
δ (ppm)

11. Computational Details

Conformational search and clustering

First, a conformational search and clustering was performed using MacroModel[28] with the following settings:

- Force Field: OPLS4
- Solvent: None
- Maximum iterations: 10,000
- Convergence threshold: 0.001
- Energy window for saving structures: 21 kJ/mol (5.02 kcal/mol)
- Excluding mirror-image conformations

Atomic root mean square deviation (RMSD) clustering to the minimum Kelley penalty value was performed if more than 20 conformers were present. Centroids of the resultant clusters are taken as the representative conformation.

DFT calculations

The conformers obtained from the conformational search and clustering were carried forward for DFT calculations. DFT calculations were run with the Gaussian16.RevC.01[29] suite of programs. Geometry optimizations were performed employing the 6-31G(d,p) basis set[30,31] and B3LYP[32–35] functional with Grimme's D3 dispersion correction with Becke-Johnson damping.[36,37] The obtained structures were verified to be minimum energy structures by frequency analysis where no imaginary frequency indicated a minimum energy structure. Single point calculations were performed with the Def2TZVP[38,39] basis set and M06-2X[40] functional for property collection.

Descriptor collection

For the automated collection of molecular descriptors and post-processing a Jupyter Notebook by Brittany Haas and Melissa Hardy was used (https://github.com/SigmanGroup/Get_Properties).[41]

Utilized descriptors

- **nbo charges** were obtained with pop=nbo7 in Gaussian.
- **% buried volume**[42–44] was obtained with MORFEUS (hydrogens included in the calculations) (https://digital-chemistry-laboratory.github.io/morfeus/). **% buried volume** corresponds to the volume percent of a sphere originating on a definable atom (the radius can be set) that is occupied by the atoms included in the sphere.
- **Mulliken electronegativity:** $\chi\ (Mulliken) = \frac{IP+EA}{2} \approx -\frac{HOMO+LUMO}{2}$

The Mulliken electronegativity (arithmetic mean of the ionization energy (IP), and electron affinity (EA) can be approximated as the arithmetic mean of the HOMO and LUMO energy using the Koopmans' theorem.[45] The arithmetic mean of the HOMO and LUMO energy was obtained from the respective DFT calculations.

Computational descriptors in **Table 1**: For primary amines listed in **Table 1**, the buried volume (**3.0 Å radius**) at nitrogen of the **lowest energy conformer** was provided.

Computational descriptors in **Figure 3**: For the partial charges (**nbo charges)**, Mulliken electronegativity, and buried volume (**3.5 Å radius**) provided in **Figure 3**, **Boltzmann-weighted descriptors** were utilized: Boltzmann-weighted average of a property from all the conformers in an ensemble (T = 298.15 K):

$$Boltzmann\ average\ property = \sum^{all\ conformers} \frac{\exp\left(\frac{-\Delta G_i}{RT}\right)}{\sum^{all\ conformers} \exp\left(\frac{-\Delta G_i}{RT}\right)} \cdot property_i$$

Geometries

The geometries utilized for property collection are listed below:

Aniline conf 1

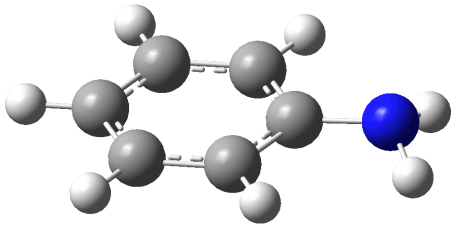

0 1
N 2.33394600 -0.00000100 -0.07718500
C 0.93817300 0.00000100 -0.01020600
C 0.22149200 -1.20784800 -0.00531600
C -1.17060900 -1.20201400 0.00336100
C -1.87995500 0.00000000 0.00825800
C -1.17061000 1.20201300 0.00336200
C 0.22149200 1.20784800 -0.00531600
H 2.77423200 -0.83544600 0.28337500
H 2.77423300 0.83544800 0.28336400
H 0.76372700 -2.14997700 -0.01286100
H -1.70430400 -2.14806100 0.00888800
H -2.96482700 -0.00000100 0.01664500
H -1.70430400 2.14806100 0.00888800
H 0.76372500 2.14997800 -0.01285900

p-Methoxyaniline conf 1

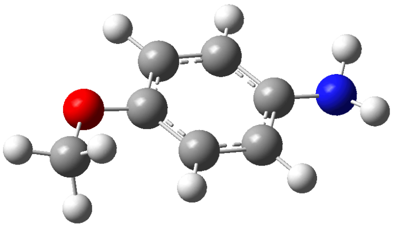

0 1
N 3.23810900 -0.30165700 -0.07773400
C 1.84781300 -0.11096600 -0.00926200
C 0.96697700 -1.19716200 -0.00716500
C -0.41732800 -1.00903800 -0.00292800
C -0.94714900 0.28298900 0.00050300
O -2.28590900 0.58291700 0.00820700
C -3.19594000 -0.49986900 0.00450500
C -0.07368500 1.37712300 -0.00091000
C 1.30022900 1.18359000 -0.00660700
H 3.54734200 -1.18747000 0.30098500
H 3.77357900 0.45884000 0.32061400

H 1.36428100 -2.20885300 -0.01366800
H -1.06385200 -1.87803000 0.00045000
H -3.08070300 -1.12518400 -0.89108500
H -3.07911200 -1.13298100 0.89450200
H -4.19416000 -0.05953200 0.00751700
H -0.49545200 2.37654600 0.00386000
H 1.96308900 2.04493100 -0.01351100

p-Methoxyaniline conf 2

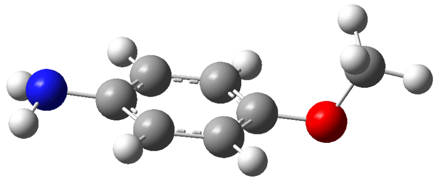

0 1
N -3.20756400 -0.00038400 0.32440300
C -1.82961000 -0.00012400 0.07495700
C -1.11898600 1.20679800 -0.02465700
C 0.26010700 1.20573100 -0.21384000
C 0.95555800 0.00037100 -0.31158700
O 2.32081600 0.00068400 -0.53876100
C 3.09507900 -0.00065100 0.65552400
C 0.26047200 -1.20524400 -0.21427900
C -1.11861200 -1.20679800 -0.02506400
H -3.68711800 0.83300000 0.01112600
H -3.68685900 -0.83380600 0.01083100
H -1.65437100 2.14990700 0.04733900
H 0.80680900 2.13909300 -0.30274300
H 2.89275000 -0.89250000 1.26404500
H 2.89276500 0.88987300 1.26599000
H 4.14468300 -0.00034100 0.35331000
H 0.80742800 -2.13842300 -0.30355200
H -1.65371000 -2.15009500 0.04659800

2,4,6-Trimethylaniline conf 1

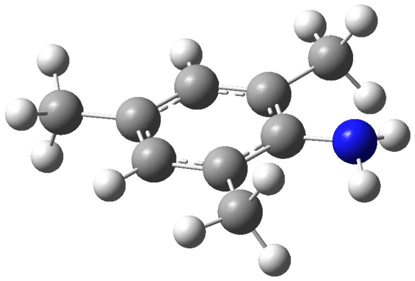

0 1
N 2.49046000 0.00005100 -0.04692100
C 1.08901100 0.00001100 0.00673800
C 0.38331600 -1.21996300 -0.00256100
C 1.13525500 -2.52595400 -0.02209100
C -1.01163700 -1.19453400 -0.00813800

C -1.73608900 -0.00004600 -0.00527300
C -3.24489300 -0.00003600 0.02433300
C -1.01167900 1.19448800 -0.00814000
C 0.38325700 1.21997100 -0.00256200
C 1.13515300 2.52598700 -0.02209000
H 2.92539000 0.83285100 0.32495700
H 2.92543300 -0.83274100 0.32492700
H 1.75318500 -2.65967200 0.87753300
H 1.80862200 -2.59062000 -0.88581600
H 0.44725000 -3.37316900 -0.06861900
H -1.54707800 -2.14151900 -0.01703900
H -3.65397900 -0.88550200 -0.47190700
H -3.62967900 0.00189700 1.05228600
H -3.65407500 0.88353300 -0.47522100
H -1.54715600 2.14145500 -0.01704900
H 0.44711900 3.37317900 -0.06863200
H 1.80852800 2.59066700 -0.88580700
H 1.75306600 2.65972900 0.87754100

2,6-Diethylaniline conf 1

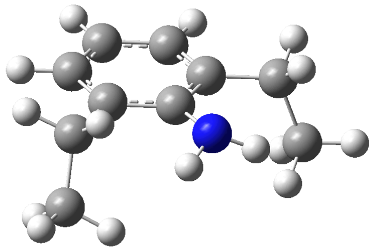

0 1
N 0.00010600 -1.71731800 -0.75072000
C 0.00004200 -0.35925300 -0.39806600
C 1.22344500 0.33234100 -0.25871000
C 2.54433500 -0.39179600 -0.39337600
C 2.95266900 -1.14471400 0.88583200
C 1.19915900 1.69667000 0.03622800
C -0.00007300 2.38808100 0.18918100
C -1.19924600 1.69658400 0.03615800
C -1.22342000 0.33224800 -0.25875700
C -2.54425300 -0.39198900 -0.39345800
C -2.95276400 -1.14463400 0.88585900
H -0.83162300 -2.22569500 -0.49122700
H 0.83186200 -2.22562500 -0.49117600
H 3.32448100 0.33664400 -0.63774600
H 2.50907100 -1.08859200 -1.24072800
H 3.06187900 -0.44538100 1.71990300
H 2.19807600 -1.88131000 1.18337100
H 3.90296000 -1.67166800 0.75206600
H 2.14551400 2.22125400 0.14252500
H -0.00011700 3.44977400 0.41353800
H -2.14564500 2.22109800 0.14241100
H -3.32439600 0.33636300 -0.63810100

H -2.50882800 -1.08896800 -1.24065400
H -3.90299800 -1.67167800 0.75205200
H -3.06216500 -0.44510800 1.71974400
H -2.19818200 -1.88111200 1.18371500

2,6-Diethylaniline conf 2

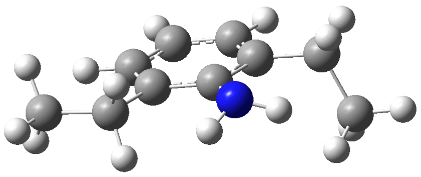

0 1
N -0.34117100 -1.90475300 -0.38210900
C -0.15421000 -0.52638100 -0.20255000
C -1.25283500 0.35437700 -0.28719000
C -2.65812100 -0.17053600 -0.47944400
C -3.29895600 -0.67155100 0.82748900
C -1.02711100 1.72667000 -0.16117300
C 0.25152200 2.23558000 0.04390500
C 1.33022900 1.35520400 0.11601700
C 1.15330100 -0.02333400 -0.00818000
C 2.31004400 -1.00059800 0.04649100
C 3.70218000 -0.38683100 0.19294200
H 0.35840100 -2.48858800 0.05315900
H -1.27094700 -2.23824400 -0.17657100
H -2.66424300 -0.97279600 -1.22872600
H -3.28098900 0.62903000 -0.89386700
H -2.70685700 -1.46820800 1.29139000
H -3.36424500 0.14257700 1.55521400
H -4.30793300 -1.05949200 0.65385600
H -1.87694800 2.40151900 -0.22786800
H 0.41021500 3.30492700 0.13864300
H 2.32820100 1.75123900 0.26766700
H 2.15242500 -1.70158000 0.88268600
H 2.28645300 -1.62184900 -0.86016500
H 3.79391800 0.18583900 1.12099700
H 3.93045000 0.28474900 -0.64034800
H 4.46403400 -1.17144400 0.20884400

2,6-Diethylaniline conf 3

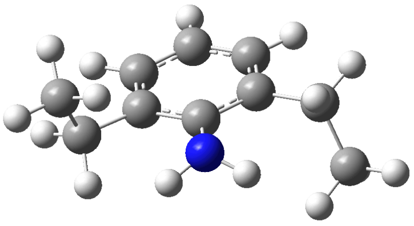

0 1
N -0.04614900 -1.82606100 0.03995500
C -0.01493000 -0.42336600 -0.00903900
C -1.18977500 0.29876200 -0.31593100
C -2.48983200 -0.41613500 -0.60715000
C -3.15497200 -0.99363000 0.65404100
C -1.14535400 1.69438800 -0.30659600
C 0.02375800 2.38495100 0.00295400
C 1.17155400 1.66005600 0.31349600
C 1.17546800 0.26405800 0.31359800
C 2.44693000 -0.49203800 0.62769200
C 3.20228800 -0.95245600 -0.63216300
H -0.78370000 -2.24761700 -0.50694100
H 0.83919900 -2.27488300 -0.14354300
H -2.33246400 -1.22232500 -1.33972100
H -3.17747100 0.28493700 -1.09168100
H -2.48889400 -1.69466100 1.16459300
H -3.39120200 -0.19031500 1.35808300
H -4.08441500 -1.51713300 0.40693000
H -2.05190700 2.24366800 -0.54788800
H 0.03859100 3.46998700 0.00687500
H 2.09200700 2.18155800 0.56428400
H 3.10645800 0.15315400 1.21742600
H 2.22338500 -1.35708600 1.26498400
H 4.10824700 -1.50952200 -0.37229000
H 3.49161700 -0.08937000 -1.23886300
H 2.58278100 -1.59551500 -1.26734000

2,6-Diethylaniline conf 4

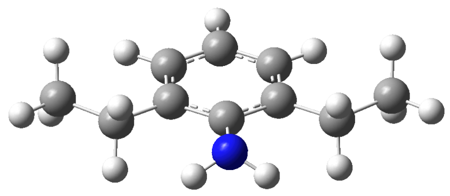

0 1
N 0.00000100 -2.08435300 0.04227700
C 0.00000000 -0.68405600 -0.02009000
C 1.22802200 0.01444200 -0.01772100
C 2.51961600 -0.77774100 0.00620700
C 3.80884200 0.04135600 0.06445600
C 1.20324000 1.40995300 -0.02622200
C -0.00000100 2.11163700 -0.03399700
C -1.20324100 1.40995300 -0.02621800
C -1.22802100 0.01444200 -0.01771600
C -2.51961600 -0.77774200 0.00621500
C -3.80884200 0.04135600 0.06444900
H 0.83285000 -2.52522800 -0.32100000
H -0.83285300 -2.52522900 -0.32098600

H 2.56292900 -1.42625700 -0.88424200
H 2.49434000 -1.46505600 0.86396300
H 3.84094600 0.66949700 0.95993400
H 4.67932900 -0.62044000 0.08859600
H 3.91016300 0.69434600 -0.80786400
H 2.13821800 1.95913200 -0.02544400
H 0.00000000 3.19689000 -0.04003500
H -2.13821900 1.95913000 -0.02543500
H -2.56292500 -1.42626900 -0.88422600
H -2.49434400 -1.46504600 0.86397900
H -3.84094800 0.66951200 0.95991600
H -4.67932900 -0.62044000 0.08859800
H -3.91016200 0.69433100 -0.80788300

2,6-Diisopropylaniline conf 1

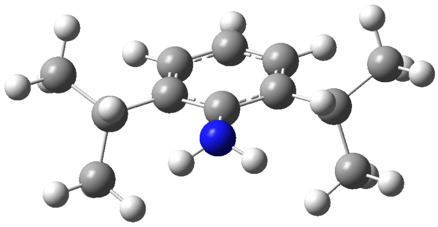

0 1
N 0.00001700 -1.76866700 -0.68189300
C 0.00001300 -0.42568600 -0.26858000
C 1.22822400 0.25555700 -0.09773900
C 2.54283600 -0.48848200 -0.29304200
C 3.71614200 0.41563400 -0.69123900
C 2.90542300 -1.29842700 0.96905300
C 1.20095200 1.59920800 0.28029100
C 0.00000200 2.27485000 0.47712000
C -1.20094400 1.59919300 0.28030700
C -1.22820500 0.25553600 -0.09770500
C -2.54280800 -0.48853500 -0.29296100
C -3.71604800 0.41548200 -0.69157500
C -2.90558300 -1.29810200 0.96932400
H -0.82954200 -2.28774400 -0.43853600
H 0.82957900 -2.28773800 -0.43853700
H 2.40158600 -1.19307100 -1.12408500
H 4.59015700 -0.19297600 -0.94357300
H 4.01069600 1.08085600 0.12691200
H 3.46644000 1.03389800 -1.55811800
H 3.80685600 -1.89988700 0.80884500
H 2.09709500 -1.97087600 1.27500400
H 3.09228100 -0.61882800 1.80682000
H 2.13642500 2.13012100 0.41863300
H -0.00000300 3.31990600 0.76995500
H -2.13642000 2.13009400 0.41866900
H -2.40145600 -1.19336900 -1.12378100
H -4.59003700 -0.19319100 -0.94385000

H -3.46622300 1.03349100 -1.55860000
H -4.01070200 1.08094400 0.12634600
H -3.09257400 -0.61824900 1.80685500
H -3.80699100 -1.89961200 0.80916000
H -2.09730400 -1.97045600 1.27560900

2,6-Diisopropylaniline conf 2

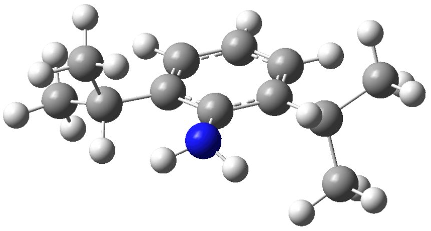

0 1
N -0.00342900 -1.87782700 0.00690700
C -0.00501600 -0.47287200 -0.03147900
C -1.23054000 0.23086700 -0.11839500
C -2.54574500 -0.53446900 -0.18280500
C -2.89837700 -1.12831900 1.19591900
C -3.72252600 0.28495400 -0.72503500
C -1.19936300 1.62709800 -0.10283100
C -0.00084600 2.32768500 -0.00305900
C 1.19509700 1.62214500 0.09222700
C 1.21925800 0.22659000 0.08487100
C 2.52799200 -0.54726300 0.17107700
C 3.64825900 0.20483300 0.90041700
C 3.00579700 -0.97286500 -1.23281600
H -0.83384400 -2.30749100 -0.37489100
H 0.82663400 -2.30890400 -0.37219600
H -2.41401000 -1.37275600 -0.88615700
H -3.80270600 -1.74407800 1.13828500
H -3.08177700 -0.31974700 1.91100000
H -2.08430300 -1.74255200 1.58866700
H -4.59744500 -0.35917000 -0.85591100
H -3.48567300 0.74177000 -1.69042200
H -4.00843900 1.08288400 -0.03207400
H -2.12935200 2.17961800 -0.17377400
H 0.00022200 3.41295200 0.00560700
H 2.12777600 2.16867500 0.17907900
H 2.33201100 -1.45570200 0.75644100
H 4.51087300 -0.45346400 1.04368800
H 3.31832200 0.55662700 1.88197100
H 3.99454500 1.07135100 0.32775800
H 3.24692200 -0.08698000 -1.82927400
H 3.90227400 -1.59954500 -1.17337100
H 2.23802200 -1.52901000 -1.78131700

2,6-Diisopropylaniline conf 3

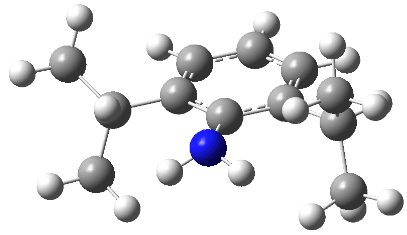

0 1
N 0.29309600 -1.68220700 0.34570800
C 0.12467200 -0.30664300 0.10936600
C -1.18666200 0.22897600 0.08578600
C -2.38485000 -0.69790000 0.24539800
C -3.62611100 -0.01444400 0.83293200
C -2.73943400 -1.37053000 -1.09712300
C -1.35312000 1.60018100 -0.11274200
C -0.26125000 2.44856300 -0.27249100
C 1.02075700 1.91191800 -0.22376300
C 1.24565400 0.54523600 -0.03335900
C 2.69085500 0.05848400 0.01332300
C 3.08174300 -0.82362600 -1.18921200
C 3.07728200 -0.57988700 1.36076000
H -0.46047400 -2.26186000 0.00745400
H 1.18258500 -2.05132200 0.05020200
H -2.09819000 -1.48297400 0.95771300
H -4.39864800 -0.75976300 1.04668700
H -4.05980400 0.70926500 0.13519200
H -3.38909700 0.51045700 1.76263200
H -3.54727200 -2.10001700 -0.97392300
H -1.88150400 -1.88523400 -1.54275900
H -3.06855100 -0.61450300 -1.81740800
H -2.35503200 2.01452300 -0.13665100
H -0.40811000 3.51308700 -0.42380700
H 1.88164100 2.56590900 -0.33676600
H 3.30427800 0.96287000 -0.07868500
H 2.84242200 -0.31851400 -2.12918500
H 2.57088100 -1.79253900 -1.20505400
H 4.15673800 -1.03165900 -1.17388900
H 2.88499200 0.11911200 2.17965900
H 2.51143700 -1.48987100 1.57507100
H 4.14281600 -0.83348100 1.37032100

2,6-Diisopropylaniline conf 4

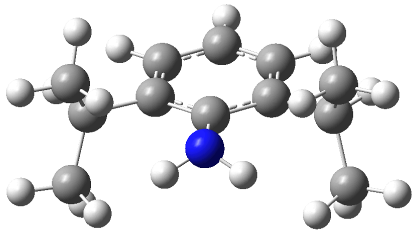

0 1
N 0.00003300 -1.58818000 0.01739600
C 0.00000800 -0.18286200 -0.00757500
C -1.22759600 0.52444100 0.01346600
C -2.59465700 -0.15453600 0.02292100
C -2.93360500 -0.87961700 -1.29496200
C -2.82944400 -1.04210400 1.25950000
C -1.19582000 1.92144900 0.03462200
C -0.00000100 2.63122600 0.04076600
C 1.19582300 1.92146000 0.03466900
C 1.22760200 0.52445300 0.01351300
C 2.59465600 -0.15453100 0.02300500
C 2.93371500 -0.87947000 -1.29493000
C 2.82929200 -1.04226600 1.25949300
H -0.82784000 -2.00959200 -0.37357900
H 0.82793000 -2.00955400 -0.37357300
H -3.32162300 0.66316500 0.09670600
H -3.97515600 -1.21746900 -1.28376100
H -2.31679400 -1.76593100 -1.48045000
H -2.79867600 -0.20889500 -2.14812100
H -3.85290100 -1.43240700 1.25813600
H -2.68712100 -0.46047300 2.17476600
H -2.14211200 -1.88987800 1.30568800
H -2.14040900 2.45909800 0.04754200
H -0.00000600 3.71620200 0.05713300
H 2.14041000 2.45911400 0.04762400
H 3.32162500 0.66315000 0.09695500
H 3.97528200 -1.21727400 -1.28369600
H 2.79880600 -0.20868000 -2.14803900
H 2.31696300 -1.76580400 -1.48052800
H 2.14187900 -1.88998500 1.30551100
H 3.85271800 -1.43265300 1.25815900
H 2.68694700 -0.46074200 2.17482300

p-Trifluoromethylaniline conf 1

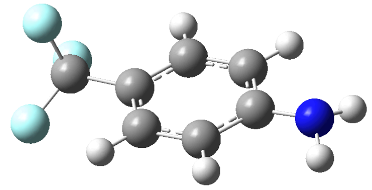

0 1
N 3.81115500 0.00000300 0.08615200
C 2.42557900 0.00000200 0.01170700
C 1.70753400 1.20947100 -0.00146000
C 0.31988200 1.20570600 -0.02374300
C -0.38625000 -0.00000300 -0.03720000
C -1.88164400 0.00000300 -0.00193700
F -2.36639400 0.00000200 1.26562800
F -2.40279100 1.09150400 -0.61080700
F -2.40279200 -1.09150600 -0.61079800
C 0.31988700 -1.20571200 -0.02374600
C 1.70753800 -1.20947100 -0.00146300
H 4.27178200 0.84104700 -0.23001600
H 4.27178000 -0.84104800 -0.23000200
H 2.24736800 2.15211700 0.00623300
H -0.22187800 2.14482900 -0.04232800
H -0.22187400 -2.14483300 -0.04233100
H 2.24737900 -2.15211300 0.00622600

o-Isopropylaniline conf 1

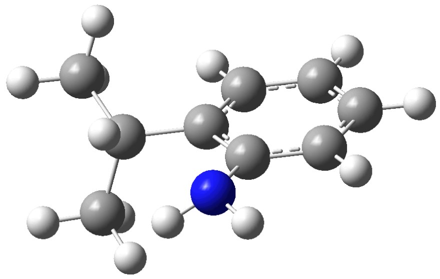

0 1
N 0.24175100 2.24265700 -0.42245000
C 0.72522000 0.95013900 -0.17071400
C -0.15846200 -0.15252500 -0.10037500
C -1.65793500 0.07460300 -0.21907900
C -2.25244500 0.52066000 1.13263100
C -2.42899000 -1.13570100 -0.75981700
C 0.38629800 -1.42260400 0.10315700
C 1.76060700 -1.63062500 0.22256400
C 2.62205500 -0.53909600 0.13571700
C 2.10708800 0.73914300 -0.05578000
H -0.65623300 2.45481700 -0.01226500
H 0.91168900 2.97253200 -0.22059900
H -1.80301500 0.89022700 -0.94044100

H -3.31517400 0.76778600 1.03548500
H -1.73670500 1.39545400 1.54320700
H -2.15445100 -0.28385400 1.86886900
H -1.99791400 -1.49548000 -1.69835800
H -3.47354100 -0.86502200 -0.94314000
H -2.43069200 -1.96635000 -0.04638600
H -0.28037000 -2.27642700 0.16239500
H 2.14969400 -2.63187500 0.37646100
H 3.69578100 -0.67698100 0.22214400
H 2.77806300 1.59260700 -0.12004800

o-Isopropylaniline conf 2

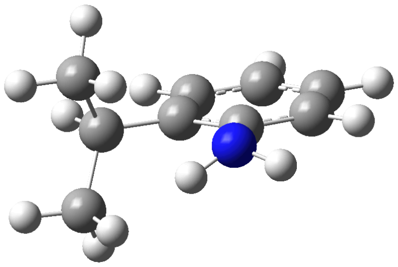

0 1
N 0.37606000 2.04262300 0.02598000
C -0.44909500 0.90896100 -0.01553000
C 0.07663600 -0.40520100 0.01374500
C 1.56769400 -0.71769000 0.03987400
C 2.28239700 -0.14815300 1.27882100
C 2.29007900 -0.34727400 -1.27010600
C -0.82933500 -1.47115900 0.02618300
C -2.21084800 -1.28730000 0.01862200
C -2.71483100 0.01143700 -0.00181700
C -1.84123900 1.09285100 -0.02133200
H 1.27547000 1.93661200 -0.41814500
H -0.08398100 2.88725600 -0.28681000
H 1.63854700 -1.80962500 0.11702600
H 3.33365600 -0.45566600 1.28748300
H 1.81044400 -0.51788400 2.19358400
H 2.24504300 0.94317500 1.31283100
H 1.78448800 -0.79444800 -2.13055500
H 3.32307900 -0.71010200 -1.25034400
H 2.33847600 0.73297200 -1.44736100
H -0.42828400 -2.48156500 0.04550800
H -2.87817200 -2.14275700 0.03063500
H -3.78635700 0.18777200 -0.00760600
H -2.23358200 2.10707300 -0.03886200

2,5-di-*tert*-Butylaniline conf 1

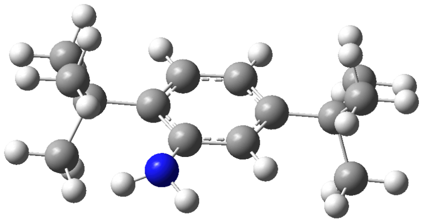

0 1
N 1.24628200 2.27643700 0.06936500
C 0.61688400 1.02197300 0.00017200
C 1.33286400 -0.20569900 -0.00158000
C 2.87191200 -0.27762100 0.00619100
C 3.38135200 -1.72947900 0.09301100
C 3.44717200 0.30105600 -1.30939600
C 3.44533900 0.46417500 1.23650300
C 0.55926600 -1.36999900 -0.01316700
C -0.83554500 -1.36925800 -0.01241400
C -1.53975800 -0.16447700 -0.00195100
C -3.07200300 -0.08521600 0.00181800
C -3.72288900 -1.47774000 -0.00510700
C -3.54340200 0.66243100 1.26750500
C -3.55052900 0.67743100 -1.25225600
C -0.78445900 1.01035500 0.00041700
H 2.14383500 2.33983400 -0.38506800
H 0.64456600 3.03716100 -0.21763000
H 4.47532200 -1.72625400 0.12210300
H 3.02609400 -2.22860700 0.99917600
H 3.07610800 -2.32545500 -0.77228000
H 4.54095600 0.24462800 -1.30180200
H 3.18102800 1.34600000 -1.49116600
H 3.08059900 -0.27328900 -2.16534400
H 4.54043600 0.43940200 1.21381800
H 3.12430400 1.50425400 1.29679900
H 3.11446300 -0.03028000 2.15496000
H 1.05603100 -2.33166500 -0.01998400
H -1.35579900 -2.31871500 -0.01904600
H -3.43922400 -2.05979100 0.87705300
H -3.44477500 -2.04881000 -0.89624200
H -4.81261600 -1.37605800 -0.00109200
H -3.14473100 1.67981400 1.30789000
H -4.63686200 0.72872600 1.28746600
H -3.21494000 0.13939800 2.17090700
H -3.15461700 1.69635500 -1.28119800
H -4.64414700 0.74208000 -1.26632400
H -3.22570200 0.16635700 -2.16391000
H -1.29153000 1.97226200 0.00688600

2,5-di-*tert*-Butylaniline conf 2

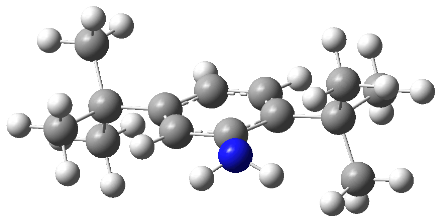

0 1
N 1.28188200 2.29124400 -0.06858700
C 0.63614800 1.04536500 -0.00032800
C 1.33099400 -0.18974800 0.00188200
C 2.86870300 -0.28723400 -0.00618000
C 3.35425300 -1.74732100 -0.09244200
C 3.45384700 0.44420400 -1.23715200
C 3.45396600 0.28283400 1.30871600
C 0.53706900 -1.34477300 0.01378700
C -0.85273400 -1.31758300 0.01326400
C -1.54067700 -0.09814800 0.00211200
C -3.07456900 -0.07647400 -0.00170900
C -3.58825900 -0.80819800 -1.26036400
C -3.59677000 -0.79772700 1.25939100
C -3.64291300 1.35159200 -0.00973900
C -0.77017600 1.06115500 -0.00121900
H 2.18446600 2.34089500 0.37733400
H 0.69307300 3.05940500 0.22504600
H 4.44812700 -1.76215600 -0.12189900
H 3.03962100 -2.33784600 0.77325400
H 2.99063300 -2.24110100 -0.99825700
H 4.54844100 0.40213600 -1.21475700
H 3.14962400 1.48931900 -1.29855800
H 3.11484700 -0.04554800 -2.15515600
H 4.54678900 0.20978800 1.30047200
H 3.20351700 1.33169600 1.49026600
H 3.07922900 -0.28557100 2.16509400
H 1.01878500 -2.31406400 0.02112900
H -1.39628700 -2.25647100 0.02047200
H -3.24816600 -1.84679200 -1.28902800
H -3.23001300 -0.31400900 -2.16875300
H -4.68375200 -0.81150400 -1.28042900
H -3.24501900 -0.29602000 2.16632800
H -4.69234800 -0.80091800 1.27207100
H -3.25720300 -1.83611700 1.29926800
H -3.33420600 1.91468600 0.87673900
H -4.73665900 1.31346100 -0.01397900
H -3.32658200 1.90767700 -0.89778600
H -1.25337900 2.03267100 -0.00888100

2,5-di-*tert*-Butylaniline conf 3

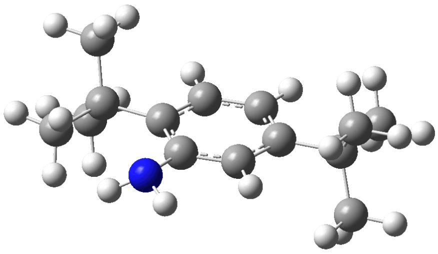

0 1
N 1.17741800 2.37305300 0.05770000
C 0.60128000 1.09105000 -0.02149200
C 1.34273300 -0.11767400 -0.03995500
C 2.88144800 -0.29131500 0.00821800
C 3.71614200 1.00142300 -0.07174200
C 3.24765900 -0.97904900 1.34408700
C 3.33904900 -1.18157400 -1.17034800
C 0.58678500 -1.29874400 -0.05261600
C -0.80324300 -1.33827800 -0.03877100
C -1.53516500 -0.14821600 -0.01213600
C -3.06860700 -0.10265400 0.00571000
C -3.57536100 0.65285200 -1.24153600
C -3.68769300 -1.50969300 0.00016300
C -3.54507200 0.63029200 1.27800200
C -0.80375800 1.03818700 -0.00957300
H 0.50836600 3.10345900 -0.14889000
H 2.01607700 2.50344200 -0.48475500
H 4.77647800 0.73969900 0.00020700
H 3.58903600 1.51173100 -1.03420400
H 3.48848400 1.69374100 0.74048500
H 4.32873700 -1.14881800 1.40423900
H 2.74780000 -1.94485400 1.45168900
H 2.95089000 -0.35353800 2.19153800
H 4.42619000 -1.31436800 -1.14213500
H 2.88515800 -2.17455100 -1.14454100
H 3.07634200 -0.72023200 -2.12767800
H 1.11230600 -2.24770600 -0.06141700
H -1.29958500 -2.30042300 -0.04642500
H -3.24722000 0.15211600 -2.15771200
H -3.20247000 1.68042300 -1.27042900
H -4.67024300 0.69292700 -1.24573700
H -3.38207600 -2.08815800 0.87737700
H -4.77935300 -1.43303400 0.01529000
H -3.40568000 -2.07126300 -0.89578700
H -3.19674400 0.11214400 2.17676500
H -4.63950500 0.67193200 1.30804700
H -3.16898800 1.65624600 1.31793500
H -1.33310600 1.98806400 0.00418300

2,5-di-*tert*-Butylaniline conf 4

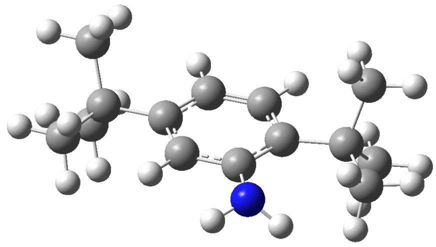

0 1
N -1.21244500 2.39021300 0.05737400
C -0.62090100 1.11553400 -0.02011900
C -1.34169100 -0.10106100 -0.03821600
C -2.87689100 -0.30157500 0.00812600
C -3.73272500 0.97802100 -0.05825500
C -3.31873800 -1.18711000 -1.17986100
C -3.23233000 -1.00852000 1.33682100
C -0.56527100 -1.27337400 -0.05165600
C 0.82058700 -1.28702800 -0.03879700
C 1.53674500 -0.08199700 -0.01175700
C 3.07006700 -0.09398000 0.00549600
C 3.66967600 1.32093800 0.02985500
C 3.55564800 -0.84691800 1.26289900
C 3.58702200 -0.81751900 -1.25647900
C 0.79003100 1.08938100 -0.00788500
H -0.55643500 3.12866900 -0.16139200
H -2.06158900 2.50666700 -0.47131300
H -4.78844500 0.69888000 0.01627200
H -3.51264900 1.66772300 0.75845500
H -3.61834100 1.49774600 -1.01727100
H -4.40328600 -1.34042100 -1.15308200
H -2.84633900 -2.17171800 -1.16478200
H -3.06469400 -0.71048800 -2.13199800
H -4.31036500 -1.19788300 1.39379300
H -2.71626000 -1.96661000 1.43649700
H -2.94764200 -0.38577700 2.19039300
H -1.07607500 -2.23035500 -0.06175600
H 1.33988600 -2.23949200 -0.04734000
H 3.35659700 1.87733400 0.91885900
H 3.38230700 1.89709700 -0.85538900
H 4.76220400 1.25840800 0.04464300
H 3.19163100 -1.87765900 1.28059300
H 4.65056200 -0.87574800 1.29212700
H 3.20107400 -0.35139600 2.17198400
H 3.22368200 -1.84738600 -1.30763000
H 4.68229900 -0.84640300 -1.25896100
H 3.25564100 -0.30070000 -2.16257400
H 1.29596800 2.04928200 0.00721900

p-tert-Butylaniline conf 1

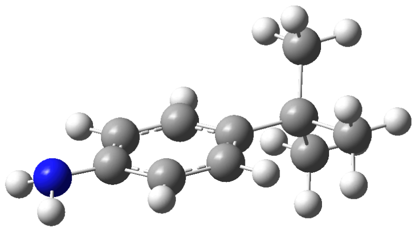

0 1
N -3.88253000 -0.01411800 -0.07176100
C -2.48535900 0.00259900 -0.00928300
C -1.77446900 1.20914100 -0.00691100
C -0.38064300 1.21613700 -0.00416700
C 0.36556500 0.03253600 -0.00239500
C 1.89936900 0.00370900 0.00287000
C 2.40440500 -0.73693500 -1.25424700
C 2.50586600 1.41615100 0.00361600
C 2.39646500 -0.73491100 1.26433200
C -0.36075800 -1.16876300 -0.00430700
C -1.74964400 -1.19325300 -0.00769000
H -4.32797500 0.81790300 0.29106400
H -4.30884100 -0.85129800 0.30202400
H -2.31766300 2.15090000 -0.01158500
H 0.12209100 2.17614200 -0.00071200
H 2.03594200 -1.76598300 -1.29103800
H 2.06810900 -0.22946200 -2.16370400
H 3.49956400 -0.77266600 -1.26555300
H 2.20993200 1.98348100 -0.88441100
H 2.20574000 1.98459800 0.88958300
H 3.59829000 1.34939000 0.00634700
H 2.05501500 -0.22566300 2.17097200
H 2.02755200 -1.76384200 1.30055800
H 3.49150800 -0.77102400 1.28252000
H 0.16853800 -2.11698400 -0.00106600
H -2.27487200 -2.14514200 -0.01358500

p-Chloroaniline conf 1

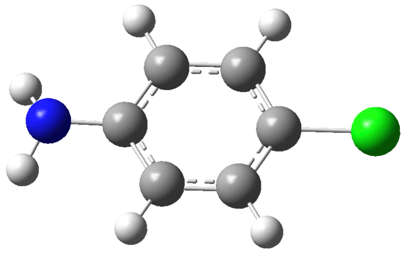

0 1
N -3.22006500 0.00000000 -0.06910400
C -1.82620600 0.00000100 -0.00792000
C -1.10795700 1.20668200 -0.00643700
C 0.28352900 1.20922000 -0.00333300

C 0.97474200 0.00000000 -0.00137500
Cl 2.73550200 0.00000000 0.00684900
C 0.28352900 -1.20921900 -0.00333400
C -1.10795700 -1.20668300 -0.00643700
H -3.66443500 0.83691200 0.28227900
H -3.66443300 -0.83691300 0.28228000
H -1.64515700 2.15089300 -0.01207500
H 0.82900500 2.14589200 -0.00004700
H 0.82900600 -2.14589200 -0.00004700
H -1.64515700 -2.15089300 -0.01207600

2-Aminopyridine conf 1

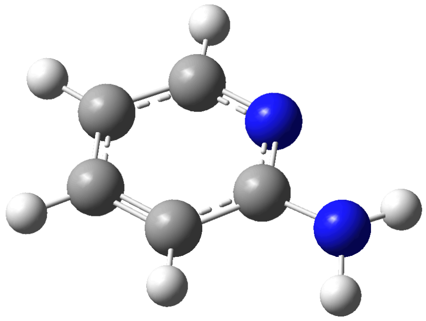

0 1
C 0.18571100 1.22024800 -0.00710000
C -1.19908600 1.16914100 0.00375900
C -1.84323400 -0.07238400 0.00616600
C -1.04430800 -1.21126500 0.00254100
N 0.29412200 -1.19206000 0.00050200
H 0.71540100 2.16771700 -0.02094900
H -1.77503300 2.08995100 0.00789400
H -2.92420000 -0.15250100 0.01381800
H -1.49871400 -2.20049400 0.00682400
C 0.90050700 0.00477800 -0.00898800
N 2.28196000 -0.01388000 -0.06792900
H 2.76811000 0.78309700 0.31475200
H 2.68431900 -0.90929700 0.17138400

3-Aminopyridine conf 1

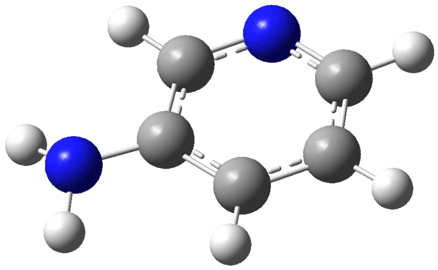

0 1
N -2.31085800 0.01998400 -0.07567300
C -0.92027700 0.02459500 -0.00977500
C -0.18257100 1.21643900 -0.00499400
C 1.20635100 1.14914700 0.00325500

C 1.82717000 -0.09985600 0.00703500
N 1.13843300 -1.24832200 0.00442800
C -0.19172800 -1.17971500 -0.00343500
H -2.75174600 -0.82975100 0.24877500
H -2.76379100 0.84475400 0.29289300
H -0.69490500 2.17500100 -0.01182300
H 1.80135000 2.05695900 0.00862600
H 2.91107400 -0.18348300 0.01442100
H -0.72867700 -2.12876900 -0.00669200

4-Bromobenzotrifluoride conf 1

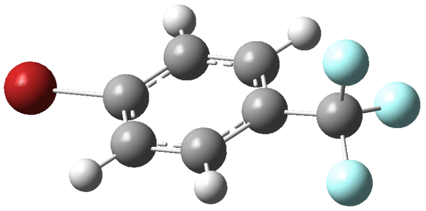

0 1
Br -3.23019100 -0.00001100 0.00998000
C -1.32359100 0.00001000 -0.00891500
C -0.64204700 -1.21550700 -0.01768100
C 0.75038300 -1.21015200 -0.03242800
C 1.44634700 0.00004200 -0.04064000
C 2.94850100 0.00000600 0.00352600
F 3.40966800 -0.00059100 1.27595600
F 3.46866600 -1.09039000 -0.60218900
F 3.46872300 1.09091100 -0.60121200
C 0.75036000 1.21021600 -0.03242500
C -0.64207400 1.21553800 -0.01768100
H -1.19250300 -2.14844800 -0.01569800
H 1.29549000 -2.14693300 -0.04673100
H 1.29545000 2.14700400 -0.04671700
H -1.19255100 2.14846600 -0.01569700

Bromobenzene conf 1

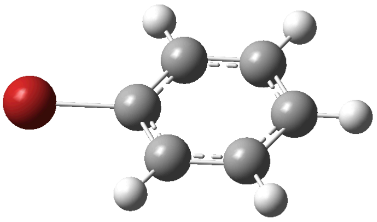

0 1
C 2.87837600 0.00000100 0.00000100
C 2.17866500 1.20724200 0.00000000
C 0.78371500 1.21549300 -0.00000200
C 0.10338200 -0.00000300 0.00000200
Br -1.80849800 0.00000000 0.00000000
C 0.78371600 -1.21549300 0.00000200
C 2.17867100 -1.20723900 -0.00000200

H 3.96367000 0.00000700 0.00000100
H 2.71633500 2.15041500 0.00000100
H 0.23096200 2.14773800 -0.00000100
H 0.23097300 -2.14774500 0.00000100
H 2.71633700 -2.15041600 -0.00000400

1-Bromo-4-chlorobenzene conf 1

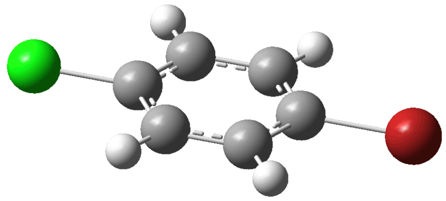

0 1
Cl -3.80824600 0.00000000 0.00000000
C -2.05326500 0.00000000 -0.00000100
C -1.36843500 1.21395000 0.00000000
C 0.02541200 1.21347100 0.00000000
C 0.70940700 0.00000000 0.00000000
Br 2.61737700 0.00000000 0.00000000
C 0.02541200 -1.21347200 0.00000000
C -1.36843500 -1.21395000 -0.00000100
H -1.91716100 2.14853300 0.00000000
H 0.57286300 2.14845300 -0.00000100
H 0.57286400 -2.14845300 0.00000100
H -1.91716300 -2.14853100 -0.00000100

4-Bromoanisole conf 1

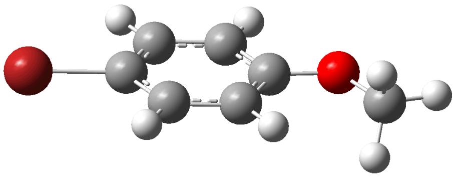

0 1
Br -2.63393800 -0.11525400 0.00000400
C -0.72878000 0.04484900 -0.00000300
C 0.05760000 -1.09997700 -0.00001200
C 1.44993400 -0.98680600 -0.00001800
C 2.04509600 0.27947000 -0.00001200
O 3.38955600 0.50226600 -0.00001800
C 4.25005200 -0.62574200 0.00002900
C 1.23870000 1.42655600 -0.00000300
C -0.14490300 1.31253100 0.00000200
H -0.40737100 -2.07886300 -0.00001600
H 2.05019200 -1.88774500 -0.00002700
H 4.10271600 -1.24524500 0.89367500
H 5.26594200 -0.22989400 0.00006000
H 4.10278000 -1.24527000 -0.89361000
H 1.71922300 2.39861200 0.00000200
H -0.76828700 2.19888200 0.00001100

1-Bromo-4-propylbenzene conf 1

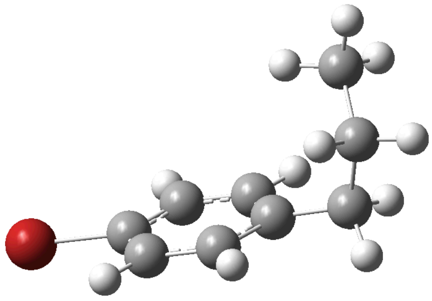

0 1
Br 2.95996200 -0.10974100 0.20504100
C 1.08364200 0.06187700 -0.12181700
C 0.38377000 -1.00745900 -0.67172600
C -0.98409900 -0.86990200 -0.90767000
C -1.66093000 0.31626300 -0.60346600
C -3.15027600 0.44967000 -0.81739900
C -3.96353400 0.29507100 0.48398400
C -3.83046300 -1.08769700 1.12330300
C -0.92529100 1.37325600 -0.05179200
C 0.44128700 1.25753000 0.19170100
H 0.89885100 -1.93004400 -0.91243700
H -1.53364000 -1.70257900 -1.33742300
H -3.48512700 -0.30290700 -1.54108500
H -3.36869500 1.42944100 -1.25833800
H -5.01770300 0.50093000 0.26205900
H -3.64046400 1.06339400 1.19756600
H -4.43194400 -1.16114000 2.03440800
H -2.79107200 -1.30376500 1.38765900
H -4.16789600 -1.87226300 0.43625500
H -1.42835500 2.30595000 0.18935200
H 1.00274900 2.08225200 0.61485300

1-Bromo-4-propylbenzene conf 2

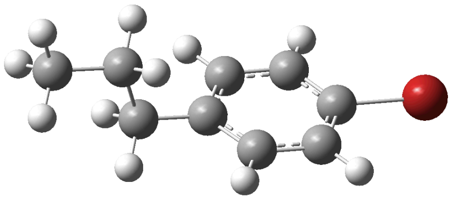

0 1
Br 3.11950700 0.00001300 0.19396200
C 1.22713200 -0.00000700 -0.08333100
C 0.55060600 1.21255200 -0.18473200
C -0.82795300 1.20096200 -0.39000500
C -1.53929200 -0.00003000 -0.49637200
C -3.03934200 -0.00003700 -0.66295700
C -3.78158000 0.00003500 0.68540100
C -5.30201600 0.00002900 0.52225000
C -0.82794100 -1.20100800 -0.38996300
C 0.55062100 -1.21257500 -0.18468900
H 1.09362900 2.14722300 -0.10786500

H -1.35925800 2.14547900 -0.47210400
H -3.34804100 -0.87931200 -1.24153200
H -3.34803800 0.87917900 -1.24162500
H -3.46499500 -0.87637100 1.26437200
H -3.46499300 0.87650200 1.26428000
H -5.80712200 0.00008100 1.49294300
H -5.63988100 -0.88371900 -0.03041100
H -5.63987800 0.88372100 -0.03050400
H -1.35923200 -2.14553600 -0.47202800
H 1.09365100 -2.14723900 -0.10779100

1-Bromo-4-propylbenzene 3

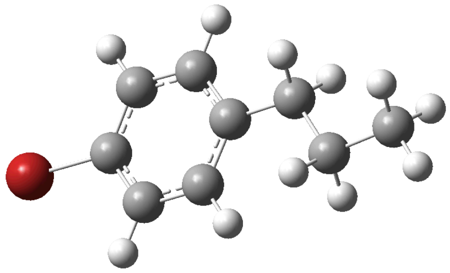

0 1
Br -3.15195600 -0.17129600 0.00000700
C -1.25255300 0.05205100 -0.00000300
C -0.42988900 -1.06788000 -0.00002100
C 0.95578800 -0.89537100 -0.00002900
C 1.53007300 0.37963300 -0.00001800
C 3.02858200 0.61016000 -0.00003200
C 3.91003100 -0.63997200 0.00003500
C 5.40045000 -0.29484100 0.00001800
C 0.66936400 1.48705100 -0.00000100
C -0.71385000 1.33736700 0.00000700
H -0.86265300 -2.06147400 -0.00002700
H 1.58661400 -1.77708600 -0.00004400
H 3.28613300 1.22574800 0.87316700
H 3.28613200 1.22565400 -0.87329800
H 3.67639700 -1.25445700 -0.87839200
H 3.67639400 -1.25436400 0.87852600
H 6.01844300 -1.19761100 0.00006300
H 5.66928200 0.29423600 0.88380100
H 5.66928200 0.29414800 -0.88382300
H 1.09122800 2.48903400 0.00000900
H -1.36675700 2.20233500 0.00002100

3-Bromopyridine conf 1

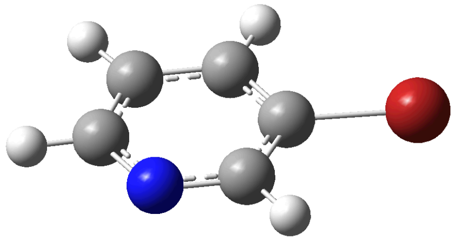

0 1
Br 1.79409600 0.00462300 0.00000000
C -0.11192500 0.01502100 0.00000100
C -0.80970700 1.21854400 0.00000100
C -2.20174000 1.15901900 0.00000100
C -2.81988900 -0.09152100 -0.00000600
N -2.14537600 -1.24784800 0.00000000
C -0.81056800 -1.19304200 0.00000300
H -0.28069500 2.16456500 0.00000200
H -2.79478000 2.06760500 0.00000500
H -3.90450700 -0.16994800 0.00000500
H -0.27277900 -2.13721800 0.00000100

phenol conf 1

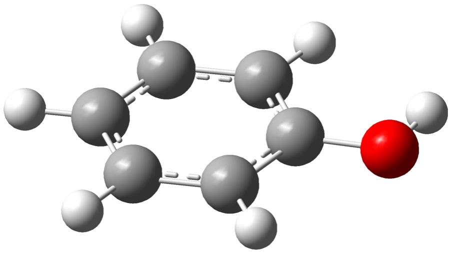

0 1
O 2.30511000 -0.11141900 0.00000300
C 0.94046700 -0.02502400 0.00000000
C 0.22055800 -1.22378600 0.00000000
C -1.17114200 -1.18945900 -0.00000100
C -1.85601100 0.02808000 0.00000000
C -1.13037300 1.21890300 0.00000000
C 0.26402700 1.19879900 0.00000100
H 2.67521100 0.78098100 -0.00002100
H 0.76515200 -2.16162300 -0.00000100
H -1.72477100 -2.12365900 -0.00000100
H -2.94069500 0.04761600 0.00000000
H -1.64814300 2.17336900 0.00000000
H 0.82721200 2.12958900 0.00000200

4-*tert*-butylphenol conf 1

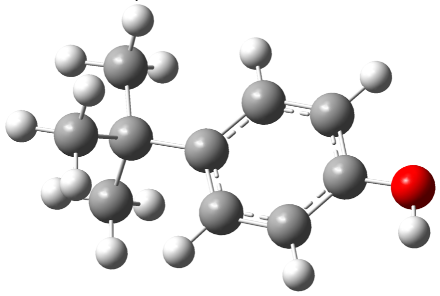

0 1
O 3.84641600 0.07064900 0.00000200
C 2.47922900 0.01899400 0.00000000
C 1.76677200 -1.18365000 0.00000000
C 0.37524400 -1.16571600 -0.00000100
C -0.35153100 0.03374800 -0.00000100

C -1.88592200 -0.00102500 0.00000100
C -2.38267300 -0.74268200 -1.25964200
C -2.38266900 -0.74274300 1.25960800
C -2.49818500 1.40908600 0.00003500
C 0.38769300 1.22369000 0.00000100
C 1.78122000 1.22633300 0.00000000
H 4.19235200 -0.83123900 -0.00001300
H 2.29965900 -2.13226300 0.00000100
H -0.15075500 -2.11534000 0.00000000
H -2.01186800 -1.77094400 -1.29558700
H -2.04471800 -0.23378000 -2.16767300
H -3.47750500 -0.78125400 -1.27511800
H -3.47750000 -0.78132500 1.27508200
H -2.04471900 -0.23388100 2.16766300
H -2.01185600 -1.77100400 1.29550600
H -2.20333700 1.97839400 -0.88702500
H -2.20335800 1.97834300 0.88713500
H -3.59014200 1.33685100 0.00002000
H -0.12197900 2.17966800 0.00000100
H 2.33932800 2.15637100 0.00000000

4-*tert*-butylphenol conf 2

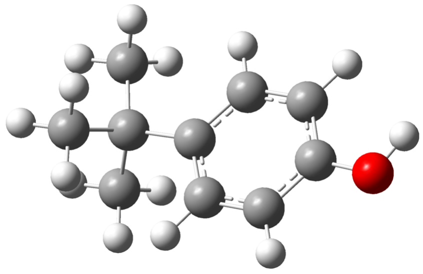

0 1
O 3.84422800 -0.09734600 -0.00000200
C 2.47870700 -0.01429000 0.00000000
C 1.79146200 1.19889300 -0.00000100
C 0.39536200 1.21267000 0.00000000
C -0.35153700 0.03080900 0.00000100
C -1.88574200 0.00875300 0.00000000
C -2.38812600 -0.72917200 1.25969500
C -2.48653700 1.42371900 -0.00000300
C -2.38812300 -0.72917600 -1.25969400
C 0.36739200 -1.17672000 0.00000100
C 1.75586300 -1.21110400 0.00000100
H 4.21029900 0.79667000 0.00002000
H 2.34294600 2.13681200 -0.00000200
H -0.10412700 2.17400800 0.00000100
H -2.04689100 -0.22241700 2.16770500
H -2.02448500 -1.75985800 1.29598300
H -3.48319400 -0.75987000 1.27458100
H -3.57905500 1.36073600 0.00000000
H -2.18710700 1.99052100 -0.88720800
H -2.18710400 1.99052500 0.88720000

H -2.02448100 -1.75986100 -1.29597800
H -2.04688700 -0.22242200 -2.16770400
H -3.48319100 -0.75987500 -1.27458100
H -0.16877500 -2.12074100 0.00000100
H 2.29590700 -2.15173900 0.00000100

Methyl 4-hydroxybenzoate conf 1

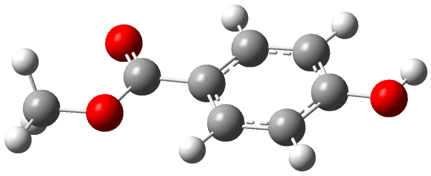

0 1
O 3.90106600 -0.35095900 -0.00005700
C 2.55598200 -0.14380300 -0.00001900
C 1.99131700 1.13824200 -0.00002100
C 0.61049800 1.28188200 0.00001900
C -0.22275700 0.15659700 0.00006400
C -1.68745300 0.37638600 0.00010900
O -2.38499500 -0.78593900 0.00001100
C -3.81082300 -0.63477600 -0.00009800
O -2.22586600 1.46761900 -0.00000800
C 0.35303700 -1.12312600 0.00006200
C 1.73189100 -1.27638100 0.00002200
H 4.35434000 0.50264800 -0.00009500
H 2.63474300 2.01490400 -0.00005200
H 0.15216000 2.26440800 0.00001900
H -4.14038500 -0.08907600 -0.88762400
H -4.21441000 -1.64691800 -0.00021200
H -4.14053300 -0.08921900 0.88746100
H -0.29016300 -1.99477900 0.00009500
H 2.19245800 -2.25786500 0.00002200

Methyl 4-hydroxybenzoate conf 2

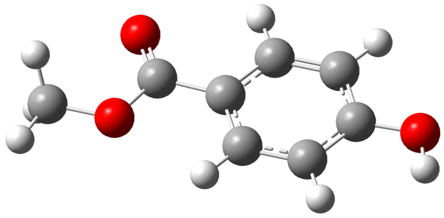

0 1
O 3.91680500 -0.19052800 0.00000000
C 2.55793300 -0.11487600 0.00000000
C 1.98570600 1.16400500 0.00000000
C 0.60600000 1.29510700 0.00000000
C -0.22311200 0.16323700 0.00000000
C -1.68924400 0.37176600 0.00000000
O -2.37829900 -0.79634700 0.00000000
C -3.80518700 -0.65527300 0.00000000
O -2.23579800 1.45864600 0.00000000

C 0.35784900 -1.11088300 0.00000000
C 1.74058200 -1.25211000 0.00000000
H 4.18419100 -1.11919500 0.00000200
H 2.63846000 2.02963300 0.00000000
H 0.14061300 2.27431300 0.00000000
H -4.13873700 -0.11215400 -0.88758600
H -4.20166100 -1.67031500 0.00000300
H -4.13873600 -0.11214800 0.88758300
H -0.27841500 -1.98755000 0.00000000
H 2.18945800 -2.24259100 0.00000000

4-(Trifluormethyl)phenol conf 1

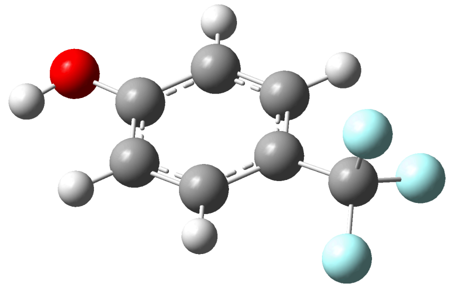

0 1
C -2.41818500 0.01575500 0.00437800
C -1.72573800 -1.20002900 -0.00762100
C -0.33457700 -1.20448200 -0.02797300
C 0.37176900 -0.00134900 -0.03935800
C 1.86962300 -0.00543600 0.00040800
F 2.34460200 0.04807800 1.26913300
F 2.38739800 -1.12276200 -0.55997000
F 2.39370700 1.05757400 -0.65218600
C -0.32502600 1.21144000 -0.02771500
C -1.71290600 1.22508200 -0.00708700
H -2.27393700 -2.13881300 -0.00466900
H 0.20376700 -2.14517400 -0.04362900
H 0.22462900 2.14584800 -0.04385700
H -2.26842300 2.15588100 -0.00333900
O -3.77778100 0.08713700 0.02307300
H -4.14490400 -0.80672600 0.02791900

4-Methoxyphenol conf 1

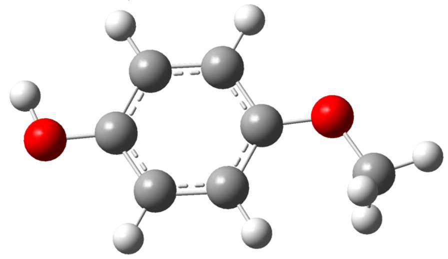

0 1
O -3.18991700 -0.39497200 0.00000100
C -1.84397000 -0.13195600 0.00000100
C -1.32542600 1.16782800 0.00000100

C 0.04933400 1.37375400 -0.00000100
C 0.92838500 0.28541100 -0.00000400
O 2.26318600 0.59492200 -0.00000700
C 3.18467900 -0.47957000 0.00000900
C 0.41086800 -1.01422700 -0.00000500
C -0.96981300 -1.21751400 -0.00000200
H -3.67035200 0.44261100 0.00002600
H -1.99819200 2.02254300 0.00000100
H 0.46310300 2.37617300 0.00000100
H 4.17783000 -0.02834400 0.00001700
H 3.07498100 -1.10908300 -0.89301400
H 3.07496100 -1.10907500 0.89303500
H 1.06735700 -1.87541100 -0.00000900
H -1.38018700 -2.22136900 -0.00000200

4-Methoxyphenol conf 2

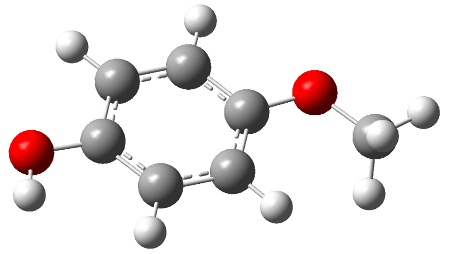

0 1
O -3.21250400 -0.22212700 0.00001000
C -1.84662500 -0.10053800 0.00000200
C -1.31387700 1.19467900 0.00000400
C 0.06009600 1.38140200 -0.00000100
C 0.93025300 0.28198700 -0.00000900
O 2.26742800 0.57938500 -0.00001700
C 3.17851100 -0.50355500 0.00002100
C 0.40154300 -1.01001600 -0.00001200
C -0.98524000 -1.19493400 -0.00000600
H -3.44114700 -1.16043000 -0.00000200
H -1.99197600 2.04102200 0.00001000
H 0.48714800 2.37833900 0.00000100
H 4.17604200 -0.06233800 0.00003700
H 3.06226100 -1.13221300 -0.89294200
H 3.06221700 -1.13218800 0.89299400
H 1.04699900 -1.87937100 -0.00002000
H -1.38890800 -2.20503000 -0.00000700

Hexylamine low energy conformer

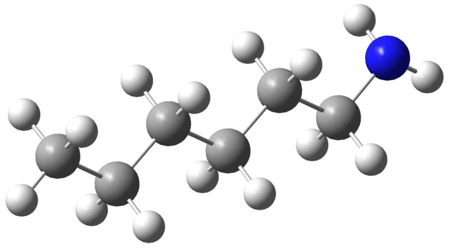

0 1
N 3.75362700 -0.39600500 -0.08733600
C 2.57311400 0.46889500 0.01135200
C 1.29250300 -0.36237500 -0.03364300
C 0.02021700 0.48647000 0.01538700
C -1.26441000 -0.34535000 -0.01849500
C -2.53984500 0.50077400 0.02587500
C -3.81796900 -0.33953300 -0.00775000
H 4.59889300 0.17000400 -0.07757400
H 3.80553200 -0.98945600 0.73857100
H 2.58716600 1.15291500 -0.84663200
H 2.55520500 1.10538500 0.91539100
H 1.29134200 -1.06682800 0.81125400
H 1.30678000 -0.97416100 -0.94353000
H 0.02650000 1.10590900 0.92334500
H 0.01900500 1.18859400 -0.83016600
H -1.26548900 -1.04729400 0.82737200
H -1.27174000 -0.96559400 -0.92565100
H -2.53045000 1.12056500 0.93253700
H -2.53754000 1.20123800 -0.81997500
H -4.71394600 0.28842500 0.02472600
H -3.86759100 -0.94477400 -0.91983800
H -3.86070900 -1.02617300 0.84517000

Cyclohexylamine low energy conformer

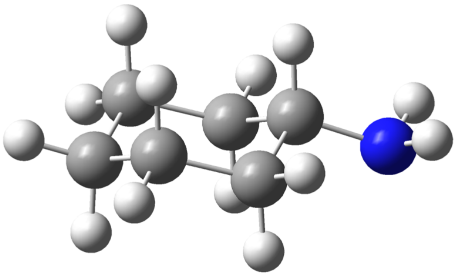

0 1
N 2.41178100 -0.00000100 -0.17217400
C 1.02869700 -0.00000100 0.31603400
C 0.31068700 1.25836700 -0.18520500
C -1.16960200 1.26577100 0.21550300
C -1.88454000 0.00000100 -0.27535400
C -1.16960300 -1.26577100 0.21550300
C 0.31068600 -1.25836800 -0.18520500
H 2.90300800 0.81362300 0.19310400
H 2.90301100 -0.81361900 0.19311300
H 0.96483500 -0.00000100 1.42218600
H 0.81547000 2.15304100 0.20248300
H 0.40691900 1.28945700 -1.27813500
H -1.66233600 2.16099800 -0.18032100
H -1.25034500 1.32340400 1.31006400
H -2.92969800 0.00000200 0.05500100
H -1.90098900 0.00000000 -1.37402500
H -1.25034700 -1.32340400 1.31006400

H -1.66233800 -2.16099700 -0.18032200
H 0.81546800 -2.15304200 0.20248100
H 0.40691700 -1.28945700 -1.27813500

Isopropylamine low energy conformer

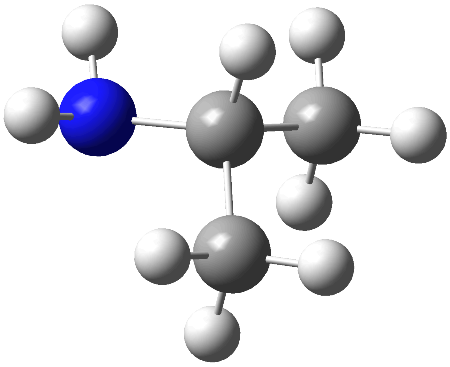

0 1
N -0.00001100 1.38165100 -0.21024100
C 0.00000000 0.02894900 0.36474300
C -1.26293800 -0.69909400 -0.09567500
C 1.26295200 -0.69907300 -0.09567700
H -0.81372300 1.89510700 0.12190500
H 0.81367000 1.89513400 0.12193900
H 0.00000100 0.03932600 1.47120100
H -2.16560500 -0.17659000 0.24118900
H -1.28955200 -0.74959500 -1.18870200
H -1.29682400 -1.71676600 0.30566000
H 1.29686900 -1.71673400 0.30568200
H 2.16560700 -0.17653500 0.24116300
H 1.28955300 -0.74959500 -1.18870300

1-Adamantylamine low energy conformer

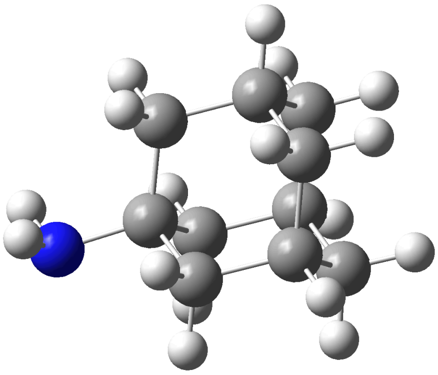

0 1
N -2.72190400 0.00000100 -0.11062400
C -1.25821800 0.00000100 -0.01554200
C -0.72924600 -0.00004000 1.43825900
C 0.81084300 -0.00004100 1.45875800
C 1.32569400 -1.25737000 0.73361400
C 0.82055400 -1.25801500 -0.72140400
C -0.71894300 -1.25150900 -0.73308900
C 1.33910400 0.00004000 -1.44286900

C 0.82055200 1.25805500 -0.72133400
C -0.71894500 1.25155000 -0.73301900
C 1.32569300 1.25733000 0.73368200
H -3.08927200 -0.81308500 0.38241500
H -3.08927400 0.81305800 0.38246100
H -1.11685400 0.88334800 1.96399600
H -1.11685500 -0.88345800 1.96394600
H 1.16603900 -0.00006700 2.49669500
H 0.97834800 -2.15948600 1.25314700
H 2.42260500 -1.27863100 0.75057200
H 1.18425200 -2.15499500 -1.23732100
H -1.10242600 -1.26366400 -1.75947800
H -1.10358200 -2.15177800 -0.23373300
H 1.00230700 0.00006800 -2.48695000
H 2.43632500 0.00004400 -1.45740100
H 1.18424900 2.15506300 -1.23720400
H -1.10358400 2.15179300 -0.23361800
H -1.10242700 1.26376100 -1.75940900
H 0.97834300 2.15941600 1.25326600
H 2.42260400 1.27859300 0.75064200

tert-Butylamine low energy conformer

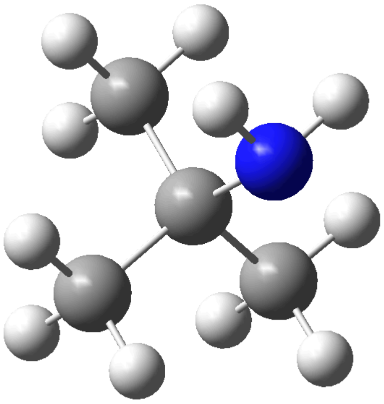

0 1
N 0.00000500 0.16216200 1.48339900
C -0.00000200 -0.00540300 0.01636800
C -1.25673700 -0.80380600 -0.35241400
C 0.00006600 1.34375500 -0.72762900
C 1.25666300 -0.80392500 -0.35240800
H -0.81253300 0.71333900 1.75614600
H 0.81258600 0.71327500 1.75614700
H -2.16344500 -0.25049700 -0.08088800
H -1.29572000 -0.99944400 -1.42860300
H -1.26943200 -1.75822000 0.18056600
H -0.88648600 1.93156800 -0.46334000
H 0.00006200 1.20824600 -1.81509200
H 0.88667400 1.93148000 -0.46333500
H 2.16341800 -0.25070000 -0.08087300
H 1.26926000 -1.75834100 0.18056900
H 1.29563200 -0.99956100 -1.42859800

12. References

(1) Williams, D. B. G.; Lawton, M. Drying of Organic Solvents: Quantitative Evaluation of the Efficiency of Several Desiccants. *J. Org. Chem.* **2010**, *75* (24), 8351–8354. https://doi.org/10.1021/jo101589h.

(2) Engle, S. Preparation of 2,4,5,6-Tetra(9H-Carbazol-9-Yl)Isophthalonitrile. *Org. Synth.* **2019**, *96*, 455–473. https://doi.org/10.15227/orgsyn.096.0455.

(3) Fulmer, G. R.; Miller, A. J. M.; Sherden, N. H.; Gottlieb, H. E.; Nudelman, A.; Stoltz, B. M.; Bercaw, J. E.; Goldberg, K. I. NMR Chemical Shifts of Trace Impurities: Common Laboratory Solvents, Organics, and Gases in Deuterated Solvents Relevant to the Organometallic Chemist. *Organometallics* **2010**, *29* (9), 2176–2179. https://doi.org/10.1021/om100106e.

(4) Ji, Y.; DiRocco, D. A.; Kind, J.; Thiele, C. M.; Gschwind, R. M.; Reibarkh, M. LED-Illuminated NMR Spectroscopy: A Practical Tool for Mechanistic Studies of Photochemical Reactions. *ChemPhotoChem* **2019**, *3* (10), 984–992. https://doi.org/10.1002/cptc.201900109.

(5) Hansch, Corwin.; Leo, A.; Taft, R. W. A Survey of Hammett Substituent Constants and Resonance and Field Parameters. *Chem. Rev.* **1991**, *91* (2), 165–195. https://doi.org/10.1021/cr00002a004.

(6) Ghosh, I.; Shlapakov, N.; Karl, T. A.; Düker, J.; Nikitin, M.; Burykina, J. V.; Ananikov, V. P.; König, B. General Cross-Coupling Reactions with Adaptive Dynamic Homogeneous Catalysis. *Nature* **2023**, *619* (7968), 87–93. https://doi.org/10.1038/s41586-023-06087-4.

(7) Senecal, T. D.; Parsons, A. T.; Buchwald, S. L. Room Temperature Aryl Trifluoromethylation via Copper-Mediated Oxidative Cross-Coupling. *J. Org. Chem.* **2011**, *76* (4), 1174–1176. https://doi.org/10.1021/jo1023377.

(8) Sergeev, A. G.; Hartwig, J. F. Selective, Nickel-Catalyzed Hydrogenolysis of Aryl Ethers. *Science* **2011**, *332* (6028), 439–443. https://doi.org/10.1126/science.1200437.

(9) Zhang, H.-J.; Chen, L.; Oderinde, M. S.; Edwards, J. T.; Kawamata, Y.; Baran, P. S. Chemoselective, Scalable Nickel-Electrocatalytic O-Arylation of Alcohols. *Angew. Chem., Int. Ed.* **2021**, *60* (38), 20700–20705. https://doi.org/10.1002/anie.202107820.

(10) Terrett, J. A.; Cuthbertson, J. D.; Shurtleff, V. W.; MacMillan, D. W. C. Switching on Elusive Organometallic Mechanisms with Photoredox Catalysis. *Nature* **2015**, *524* (7565), 330–334. https://doi.org/10.1038/nature14875.

(11) Song, G.; Li, Q.; Nong, D.-Z.; Song, J.; Li, G.; Wang, C.; Xiao, J.; Xue, D. Ni-Catalyzed Photochemical C−N Coupling of Amides with (Hetero)Aryl Chlorides. *Chem. Eur. J.* **2023**, *29* (37), e202300458. https://doi.org/10.1002/chem.202300458.

(12) Corcoran, E. B.; Pirnot, M. T.; Lin, S.; Dreher, S. D.; DiRocco, D. A.; Davies, I. W.; Buchwald, S. L.; MacMillan, D. W. C. Aryl Amination Using Ligand-Free Ni(II) Salts and Photoredox Catalysis. *Science* **2016**, *353* (6296), 279–283. https://doi.org/10.1126/science.aag0209.

(13) Moon, S.-Y.; Nam, J.; Rathwell, K.; Kim, W.-S. Copper-Catalyzed Chan–Lam Coupling between Sulfonyl Azides and Boronic Acids at Room Temperature. *Org. Lett.* **2014**, *16* (2), 338–341. https://doi.org/10.1021/ol403717f.

(14) Koizumi, Y.; Taniguchi, K.; Jin, X.; Yamaguchi, K.; Nozaki, K.; Mizuno, N. Formal Arylation of NH3 to Produce Diphenylamines over Supported Pd Catalysts. *Chem. Commun.* **2017**, *53* (78), 10827–10830. https://doi.org/10.1039/C7CC06737B.

(15) Ruos, M. E.; Kinney, R. G.; Ring, O. T.; Doyle, A. G. A General Photocatalytic Strategy for Nucleophilic Amination of Primary and Secondary Benzylic C–H Bonds. *J. Am. Chem. Soc.* **2023**, *145* (33), 18487–18496. https://doi.org/10.1021/jacs.3c04912.

(16) Xu, S.; Guo, H.; Liu, Y.; Chang, W.; Feng, J.; He, X.; Zhang, Z. Rh(I)-Catalyzed Coupling of Azides with Boronic Acids Under Neutral Conditions. *Org. Lett.* **2022**, *24* (30), 5546–5551. https://doi.org/10.1021/acs.orglett.2c02053.

(17) Rao, D. N.; Rasheed, S.; Aravinda, S.; Vishwakarma, R. A.; Das, P. Base and Ligand Free Copper-Catalyzed N-Arylation of 2-Amino-N-Heterocycles with Boronic Acids in Air. *RSC Adv.* **2013**, *3* (29), 11472–11475. https://doi.org/10.1039/C3RA40735G.

(18) Lim, C.-H.; Kudisch, M.; Liu, B.; Miyake, G. M. C–N Cross-Coupling via Photoexcitation of Nickel–Amine Complexes. *J. Am. Chem. Soc.* **2018**, *140* (24), 7667–7673. https://doi.org/10.1021/jacs.8b03744.

(19) Kore, N.; Pazdera, P. New Stable Cu(I) Catalyst Supported on Weakly Acidic Polyacrylate Resin for Green C-N Coupling: Synthesis of N-(Pyridin-4-Yl)Benzene Amines and N,N-Bis(Pyridine-4-Yl)Benzene Amines. *Molecules* **2017**, *22* (1), 2. https://doi.org/10.3390/molecules22010002.

(20) Pierce, J. K.; Hiatt, L. D.; Howard, J. R.; Hu, H.; Qu, F.; Shaughnessy, K. H. Amines as Activating Ligands for Phosphine Palladium(II) Precatalysts: Effect of Amine Ligand Identity on the Catalyst Efficiency. *Organometallics* **2022**, *41* (24), 3861–3871. https://doi.org/10.1021/acs.organomet.2c00518.

(21) Zhou, Y.; Verkade, J. G. Highly Efficient Ligands for the Palladium-Assisted Double N-Arylation of Primary Amines for One-Sep Construction of Carbazoles. *Adv. Synth. Catal.* **2010**, *352* (4), 616–620. https://doi.org/10.1002/adsc.200900846.
(22) Taillefer, M.; Xia, N.; Ouali, A. Efficient Iron/Copper Co-Catalyzed Arylation of Nitrogen Nucleophiles. *Angew. Chem., Int. Ed.* **2007**, *46* (6), 934–936. https://doi.org/10.1002/anie.200603173.
(23) Vaddula, B.; Leazer, J.; Varma, R. S. Copper-Catalyzed Ultrasound-Expedited N-Arylation of Sulfoximines Using Diaryliodonium Salts. *Adv. Synth. Catal.* **2012**, *354* (6), 986–990. https://doi.org/10.1002/adsc.201100808.
(24) Biscoe, M. R.; Fors, B. P.; Buchwald, S. L. A New Class of Easily Activated Palladium Precatalysts for Facile C−N Cross-Coupling Reactions and the Low Temperature Oxidative Addition of Aryl Chlorides. *J. Am. Chem. Soc.* **2008**, *130* (21), 6686–6687. https://doi.org/10.1021/ja801137k.
(25) Sapountzis, I.; Knochel, P. A New General Preparation of Polyfunctional Diarylamines by the Addition of Functionalized Arylmagnesium Compounds to Nitroarenes. *J. Am. Chem. Soc.* **2002**, *124* (32), 9390–9391. https://doi.org/10.1021/ja026718r.
(26) Zhang, W.; Xie, J.; Rao, B.; Luo, M. Iron-Catalyzed N-Arylsulfonamide Formation through Directly Using Nitroarenes as Nitrogen Sources. *J. Org. Chem.* **2015**, *80* (7), 3504–3511. https://doi.org/10.1021/acs.joc.5b00130.
(27) Zhu, C.; Kale, A. P.; Yue, H.; Rueping, M. Redox-Neutral Cross-Coupling Amination with Weak N-Nucleophiles: Arylation of Anilines, Sulfonamides, Sulfoximines, Carbamates, and Imines via Nickelaelectrocatalysis. *JACS Au.* **2021**, *1* (7), 1057–1065. https://doi.org/10.1021/jacsau.1c00148.
(28) Schrödinger Release 2021-4: MacroModel, Schrödinger, LLC, New York, NY, 2021.
(29) Gaussian 16, Revision C.01. Frisch, M. J., Trucks, G. W., Schlegel, H. B., Scuseria, G. E., Robb, M. A., Cheeseman, J. R., Scalmani, G., Barone, V., Petersson, G. A., Nakatsuji, H., Li, X., Caricato, M., Marenich, A. V., Bloino, J., Janesko, B. G., Gomperts, R., Mennucci, B., Hratchian, H. P., Ortiz, J. V., Izmaylov, A. F., Sonnenberg, J. L., Williams-Young, D., Ding, F., Lipparini, F., Egidi, F., Goings, J., Peng, B., Petrone, A., Henderson, T., Ranasinghe, D., Zakrzewski, V. G., Gao, J., Rega, N., Zheng, G., Liang, W., Hada, M., Ehara, M., Toyota, K., Fukuda, R., Hasegawa, J., Ishida, M., Nakajima, T., Honda, Y., Kitao, O., Nakai, H., Vreven, T., Throssell, K., Montgomery, J. A. J., Peralta, J. E., Ogliaro, F., Bearpark, M. J., Heyd, J. J., Brothers, E. N., Kudin, K. N., Staroverov, V. N., Keith, T. A., Kobayashi, R., Normand, J., Raghavachari, K., Rendell, A. P., Burant, J. C., Iyengar, S. S., Tomasi, J., Cossi, M., Millam, J. M., Klene, M., Adamo, C., Cammi, R., Ochterski, J. W., Martin, R. L., Morokuma, K., Farkas, O., Foresman, J. B., Fox, D. J.; Gaussian, Inc., Wallingford, CT, 2016.
(30) Godbout, N.; Salahub, D. R.; Andzelm, J.; Wimmer, E. Optimization of Gaussian-Type Basis Sets for Local Spin Density Functional Calculations. Part I. Boron through Neon, Optimization Technique and Validation. *Can. J. Chem.* **1992**, *70* (2), 560–571. https://doi.org/10.1139/v92-079.
(31) Sosa, C.; Andzelm, J.; Elkin, B. C.; Wimmer, E.; Dobbs, K. D.; Dixon, D. A. A Local Density Functional Study of the Structure and Vibrational Frequencies of Molecular Transition-Metal Compounds. *J. Phys. Chem.* **1992**, *96* (16), 6630–6636. https://doi.org/10.1021/j100195a022.
(32) Becke, A. D. Density-functional Thermochemistry. III. The Role of Exact Exchange. *J. Chem. Phys.* **1993**, *98* (7), 5648–5652. https://doi.org/10.1063/1.464913.
(33) Lee, C.; Yang, W.; Parr, R. G. Development of the Colle-Salvetti Correlation-Energy Formula into a Functional of the Electron Density. *Phys. Rev. B* **1988**, *37* (2), 785–789. https://doi.org/10.1103/PhysRevB.37.785.
(34) Stephens, P. J.; Devlin, F. J.; Chabalowski, C. F.; Frisch, M. J. Ab Initio Calculation of Vibrational Absorption and Circular Dichroism Spectra Using Density Functional Force Fields. *J. Phys. Chem.* **1994**, *98* (45), 11623–11627. https://doi.org/10.1021/j100096a001.
(35) Vosko, S. H.; Wilk, L.; Nusair, M. Accurate Spin-Dependent Electron Liquid Correlation Energies for Local Spin Density Calculations: A Critical Analysis. *Can. J. Phys.* **1980**, *58* (8), 1200–1211. https://doi.org/10.1139/p80-159.
(36) Grimme, S.; Ehrlich, S.; Goerigk, L. Effect of the Damping Function in Dispersion Corrected Density Functional Theory. *J. Comput. Chem.* **2011**, *32* (7), 1456–1465. https://doi.org/10.1002/jcc.21759.
(37) Grimme, S.; Antony, J.; Ehrlich, S.; Krieg, H. A Consistent and Accurate Ab Initio Parametrization of Density Functional Dispersion Correction (DFT-D) for the 94 Elements H-Pu. *J. Chem. Phys.* **2010**, *132* (15), 154104. https://doi.org/10.1063/1.3382344.
(38) Weigend, F. Accurate Coulomb-Fitting Basis Sets for H to Rn. *Phys. Chem. Chem. Phys.* **2006**, *8* (9), 1057–1065. https://doi.org/10.1039/B515623H.
(39) Weigend, F.; Ahlrichs, R. Balanced Basis Sets of Split Valence, Triple Zeta Valence and Quadruple Zeta Valence Quality for H to Rn: Design and Assessment of Accuracy. *Phys. Chem. Chem. Phys.* **2005**, *7* (18), 3297–3305. https://doi.org/10.1039/B508541A.

(40) Zhao, Y.; Truhlar, D. G. The M06 Suite of Density Functionals for Main Group Thermochemistry, Thermochemical Kinetics, Noncovalent Interactions, Excited States, and Transition Elements: Two New Functionals and Systematic Testing of Four M06-Class Functionals and 12 Other Functionals. *Theor. Chem. Acc.* **2008**, *120* (1), 215–241. https://doi.org/10.1007/s00214-007-0310-x.
(41) Haas, B.; Hardy, M.; V, S. S. S.; Adams, K.; Coley, C.; Paton, R.; Sigman, M. Rapid Prediction of Conformationally-Dependent DFT-Level Descriptors Using Graph Neural Networks for Carboxylic Acids and Alkyl Amines. ChemRxiv February 23, 2024. https://doi.org/10.26434/chemrxiv-2024-m5bpn.
(42) Clavier, H.; Nolan, S. P. Percent Buried Volume for Phosphine and N-Heterocyclic Carbene Ligands: Steric Properties in Organometallic Chemistry. *Chem. Commun.* **2010**, *46* (6), 841–861. https://doi.org/10.1039/B922984A.
(43) Falivene, L.; Credendino, R.; Poater, A.; Petta, A.; Serra, L.; Oliva, R.; Scarano, V.; Cavallo, L. SambVca 2. A Web Tool for Analyzing Catalytic Pockets with Topographic Steric Maps. *Organometallics* **2016**, *35* (13), 2286–2293. https://doi.org/10.1021/acs.organomet.6b00371.
(44) Poater, A.; Cosenza, B.; Correa, A.; Giudice, S.; Ragone, F.; Scarano, V.; Cavallo, L. SambVca: A Web Application for the Calculation of the Buried Volume of N-Heterocyclic Carbene Ligands. *Eur. J. Org. Chem.* **2009**, *2009* (13), 1759–1766. https://doi.org/10.1002/ejic.200801160.
(45) Pearson, R. G. Absolute Electronegativity and Hardness Correlated with Molecular Orbital Theory. *Proc. Natl. Acad. Sci. USA* **1986**, *83* (22), 8440–8441.
